# Resident Interventional Spine Course with Didactics and Hands-On Skills Lab

**DOI:** 10.15766/mep_2374-8265.11551

**Published:** 2025-10-07

**Authors:** William J. Naber, Andrew Coryea, Jared Gershowitz, Michael Haupt, Andi Garcia, SriKrishna Chandran, John Yarjanian, Sandra Hearn, David J. Kohns

**Affiliations:** 1 Resident, Department of Physical Medicine and Rehabilitation, Michigan Medicine; 2 Assistant Professor, Department of Physical Medicine and Rehabilitation, Michigan Medicine; 3 Associate Professor, Department of Physical Medicine and Rehabilitation, Michigan Medicine

**Keywords:** Spine Pain, Interventional Spine Procedures, Spine Complication, Simulation, Case-Based Learning

## Abstract

**Introduction:**

Fluoroscopically guided spine procedures are a common and integral treatment option for a comprehensive spine care plan. Unfortunately, interventional spine education and training can vary greatly between residency programs.

**Methods:**

A two-session comprehensive workshop was developed to educate residents on interventional spine procedures using a mix of interactive lectures and hands-on education. This course includes an overview of (1) national guidelines, (2) case discussions, (3) systematic chart review, (4) procedural kit preparation, (5) fluoroscopic spine procedure simulation, and (6) management of postprocedure complications. Residents were asked to complete a pre- and postcourse evaluation to report their self-perceived confidence in several domains of interventional spine care, as well as their perceived benefits of the course content.

**Results:**

Since 2018, 26 different residents have participated in the course. Participants reported a significant increase in confidence across several interventional spine care domains, particularly in addressing procedure complications and understanding procedure selection. Resident feedback provided high praise for how this course offered didactics, case discussion, and hands-on learning opportunities in a low-stress environment different from traditional lectures or clinical rotations.

**Discussion:**

This interventional spine course provides resident-level education and active experiences essential to better understand interventional spine care. This course can serve as a framework for other residency programs to develop their own interventional spine course and raise the standards for interventional spine education across all residency programs. Future efforts include the development of a competency-based tool to assess resident skill acquisition longitudinally across their training and once in independent practice.

## Educational Objectives

Upon completion of this interventional spine course, learners will be able to:

1. Analyze patient data via systematic chart review to screen patients for risks and contraindications to common interventional spine procedures.
2. Demonstrate the ability to perform the informed consent and preprocedural timeout for an interventional spine procedure.
3. Summarize how to assemble a procedure kit using sterile technique for an interventional spine procedure.
4. Remember and execute how to perform a simulated lumbosacral fluoroscopically guided procedure.
5. Identify, devise, and discuss management of common postspine procedure complications.
6. Produce documentation detailing appropriate aspects of history, informed consent, procedure performed, and fluoroscopic spine procedure time.

## Introduction

Chronic pain impacts millions of adults in the United States and costs the health care system hundreds of billions of dollars annually, of which more than 40% of US adults report chronic spine pain.^1^ As with most painful conditions, spine pain has a heterogeneous etiology that often requires a multimodal treatment approach.^2^ A comprehensive spine care treatment plan often includes a combination of physical therapy, medications, interventional spine procedures, surgeries, and psychological intervention.

The use of fluoroscopic X-rays for needle guidance in percutaneous spinal injection procedures began in the late 1900s. Advances in these procedures have allowed for more accurate delivery of the medication to the intended target while lowering the risk of procedure-related complications as compared to unguided procedures.^3,4^ The desire for nonopioid treatment options and other factors have led to a 177% increase in the incidence of interventional spine procedures between 2000 and 2014.^5^

The expanded use of fluoroscopically guided spine procedures has increased the demand for high-quality training in graduate medical education. To better prepare residents, some programs have utilized training simulators to allow hands-on, needle-driving skill practice.^5,6^ Such simulators are helpful; however, these models have inherent limitations and can be cost-prohibitive for many training programs that do not accept commercially sponsored funding for such expenses. For example, gel-based commercially manufactured spine models can cost as much as $5,000. Beyond the access to a simulator model, there is great variability in exposure to education on the foundations of interventional spine procedures across different residency programs.

From a resident's perspective, learning spine procedures by practicing skills on live patients, who are often not sedated, can prove challenging, as the ability to ask honest and broad questions is often limited. From an attending physician's perspective, it is difficult to progress a resident's procedural training if they do not have a foundation of knowledge or are unfamiliar with the procedural equipment. From a pain or spine fellowship perspective, when interviewing fellowship candidates from across the country, it is apparent that there is great variability between residency programs in the opportunity for hands-on interventional spine procedure training.^7^

Starting in 2018, our institution developed and has offered a yearly elective resident interventional spine course to improve resident understanding of the risks, benefits, and roles of interventional spine procedures within the broader scope of spine care. This course has enhanced our resident's overall confidence in various aspects of interventional spine care.^7^ Key elements of the course include selecting appropriate patients for spine procedures, conducting a preprocedure chart review based on national safety guidelines, obtaining informed consent, preparing a procedure kit, and managing potential procedure-related complications. Residents also learn the basics of fluoroscopic radiation safety, needle-driving techniques, and the fundamentals of performing many spine procedures using a cost-effective spine model. By combining didactic education, case discussions, and simulated spine procedures, the resident interventional spine course satisfies one of the most desired traits in a residency program, that of interactive hands-on training.

Elective interventional spine courses with lecture and model-based applications have been shown to benefit residents and fellows who typically perform these procedures.^7,8^ This style of training can also benefit residents and fellows across subspecialties that manage patients with spine pain, including physical medicine and rehabilitation (PM&R), anesthesiology, interventional radiology, sports medicine, and pain medicine. With spine pain being the fourth most common reason for a primary care visit, this course will also benefit other trainees who seek to learn how to better screen and refer patients for spine procedures, and may include family medicine, internal medicine, and emergency medicine.^9^

The following is a framework on how to plan, conduct, and assess residents in a structured educational and practical course to introduce the fundamental principles of interventional spine procedures with the goal of improving trainee's knowledge, skills, and confidence.

## Methods

### Personnel

This course was approved as exempt by the University of Michigan's Institutional Review Board (HUM00217164). It has been led by board-certified pain medicine or fellowship-trained interventional spine physicians who are actively involved in teaching residents and fellows. Current interventional spine fellows have also been invited to be course instructors to enhance their own teaching experience. For the rotating stations, it is recommended that there be up to a 1:3 instructor-to-resident ratio. These small groups allow for individualized instruction tailored to individual residents’ knowledge and skill levels. These small groups also allow for ample time with the spine simulator.

### Equipment and Environment

The precourse material, a brief PowerPoint presentation with suggested readings (Appendix A), can be completed on demand at the resident's convenience. The in-person portions of the course will require a lecture room with a video screen and a fluoroscopy suite supplied with informed consent paperwork, spine procedure kits (Appendix B), and fluoroscopic safety equipment (lead aprons and radiation glasses). The spine simulator is a relatively easy-to-construct, low-cost model that is based around a standard flexible plastic human spine model placed between layers of foam (Appendix C). A total spine model that spans from the occiput to the proximal femur offers the option to perform procedures throughout the spine and pelvis.

### Implementation

This course has generally been offered over two evening sessions lasting about 3 hours each and coinciding with the resident's annual spine and pain didactic block. Facilitator guidance for course organization and implementation can be found in Appendix D. Residents are notified via email approximately 1 month in advance to register for the course. A confirmation email includes precourse materials, course agenda, and additional information (Appendices A and D). For the didactic lecture and spine case review sessions, the physicians and fellows serve as a panel to contribute to the discussion.

In 2023, a spine procedure guidelines lecture (Appendix E) was offered as an on-demand precourse video lecture (Appendix F) to allow more time for case-based discussion while in-person. Based on prior resident feedback, in 2024, a new rotating station focusing on spine magnetic resonance imaging and fluoroscopic images was introduced. Appendix D provides a checklist to help instructors prepare and organize the course, with the expectation that modifications should be made to meet the needs of the individual residency program. A sample timeline of the course can be seen in the provided Figure.

**Figure.**
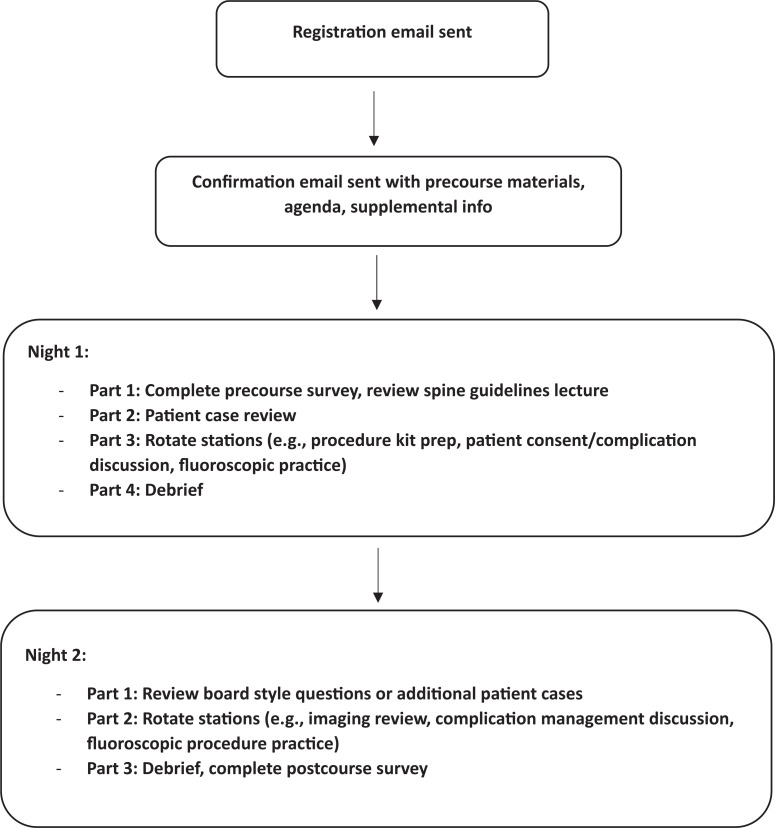
A sample time line of the course.

### Course Content

#### Interventional spine procedure guidelines lecture

Instructors can utilize and modify the spine procedure guidelines lecture slides (Appendix E) as a framework to record their own video presentation and offer this as part of the precourse materials or utilize the provided lecture video (Appendix F). Appendix G provides additional commentary that lecturers may consider using in their own presentation. If not prerecorded, the interventional spine guidelines lecture starts the course with a focus on safety and reducing risks for interventional spine procedures. This lecture is structured around a chart review focused on four broad categories of potential risk factors that any provider should be aware of before performing an interventional spine procedure. The acronym BIAS is used to represent the risk factors of bleeding, infection, allergy, and sedation. Additional topics in the lecture include emergency protocols and sedation guidelines.^10^

#### Case review

Building off the spine procedure guidelines lecture, the instructors will lead the residents through an interactive discussion of patient cases to offer various real-world applications to this content (Appendix H). Five cases have been developed to simulate a medical record that asks the residents to navigate through the requested procedure, patient clinical information, relevant provider notes, laboratory findings, and radiology reports. Additional radiology images may be available when pertinent to the case. Residents are asked to use the BIAS method of reviewing the chart and identifying any potential risks that are specific to the patient or other considerations for the procedure.^7^ There are lecture notes on each slide about how the physician managed each of these cases in real life. Instructors lead the discussion using probing phrases such as, “What would you want to know next?”; “How does this change your plans?”; and “Is there anything else that you should consider?”. At the end of each case, the instructors should offer any further insights based on additional national guidelines or their own personal experience. Instructors should also consider developing their own cases using the framework of the slides.

#### Rotating stations

After completion of the guidelines lecture and case review, residents are divided equally into groups of 2–3 residents per instructor and rotated through three 30-minute stations: (1) patient selection for various spine procedures; (2) obtaining informed consent and preparing procedure kits; and (3) needle-driving skills, radiation safety, fluoroscopic C-arm operation, and an introduction to fluoroscopically guided spine procedures using the spine simulator model. For rate-limiting each station, the instructor for the procedure simulation should keep track of time and notify the groups when it is time to rotate. Skills checklists, procedure descriptions, and spine procedure images can help guide each station (Appendices I–M).

The skills checklist in Appendices I and J includes entrustment scales modified from those used in the Ottawa Surgical Competency Operating Room Evaluation (O-Score). This score uses behavioral anchors rating one's ability to perform the task with or without supervision on a 5-point scale (1 = *low independence*, 5 = *high independence*), which provides an assessment of the learner's technical competence. Data on these checklists is currently being collected for the resident spine course.^11^ Additional learner assessments are provided in Appendices N and O. Appendix N is an updated version of Appendix O and consolidates the number of questions and improves the clarity of response options. The content and manner of presentation for each station is generally at the discretion of the instructor. This course offers flexibility to modify the content and adapt to the needs and interests of the residents.

#### Electives

If the interventional spine guidelines lecture is prerecorded and included in the precourse material, then the first in-person session could start with the case review before moving on to rotating stations. The second session could then start with board review questions, again followed by rotating stations. Several commercially available board review books on pain medicine could be used to guide the discussion, with the instructors offering further insights into high-yield topics, historical perspectives, controversies, and real-world examples.

Additional rotating stations could include understanding spine radiology imaging; management of spine procedure complications; insurance criteria for procedures; billing and coding; and pathways to a pain medicine or interventional spine fellowship. Programs may want to rotate the course agenda on a yearly basis to cover more topics as residents progress through their training. Again, this course is designed to be a framework by which local interventional spine physicians can develop their own educational program to meet the needs and interests of their own residents.

#### Debrief

Instructors provide constant feedback during and after each session of the course. A postcourse survey is administered, asking residents to rate the degree of helpfulness of various segments of the course and to share their comments and suggestions about the course (Appendix O).

#### Evaluations

To evaluate the efficacy of the course, residents are asked to complete both a precourse and postcourse survey to assess changes in their perceived confidence, knowledge, and skills in various interventional spine domains (Appendix O). The rating scales for these measures range from 1 = *low* to 5 = *high*. Additionally, in postcourse surveys, residents are asked to comment on strengths, weaknesses, and further suggestions for the course. These comments help shape the direction of future courses to meet the needs and interests of the residents.

#### Course assessment

The change in resident-perceived confidence from pre- to postcourse evaluation was analyzed for significant differences (*p* < .001) using matched-pairs *t* tests. Percent change in confidence in response to each question was computed by subtracting precourse ratings from postcourse ratings, dividing this result by the precourse rating, and then multiplying by 100. A positive change in an item reflects an increase in self-perceived confidence. In addition, to analyze whether the change in self-perceived confidence varied as a function of the experience of the resident, independent *t* tests were conducted to determine the percent change scores in residents grouped as less experienced (second-year resident) compared to those grouped as more experienced (third- and fourth-year residents).

## Results

A total of 26 different PM&R residents participated in this elective interventional spine course between 2018 and 2023. Three residents opted to repeat the course for a second time, and one took the course for all 3 years during their training. Missing data were due to the following reasons: two residents failed to attend the second date of the course, and thus there was no postcourse data; two residents failed to complete one item on both of their surveys; and one resident failed to complete three items on both of their surveys. To analyze the data without attrition, missing values were replaced with the group mean on a particular item. In total, 31 resident encounters had both baseline and follow-up survey data.

Table 1 presents pre- and postcourse mean ratings of confidence for each course item evaluated, mean percent change in ratings, and the significance of the change from pre- to postcourse. Ratings on all the items (*N* = 31 respondents) significantly increased from pre- to postcourse (all *p* < .001). The largest mean percent increase in resident self-perceived confidence was observed in the residents’ ability to perform basic skills (86.1%), address procedure complications (81.9%), and identify injection imaging targets (71.4%).

**Table 1.**
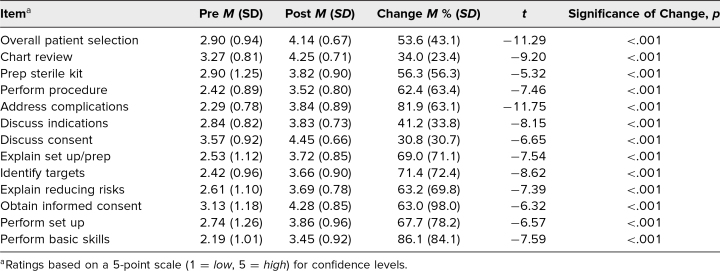
Pre- and Postcourse Resident Confidence Ratings on Each Course Item Evaluated (*N* = 31)

In 2023, three additional items were added to the precourse survey, and five additional items were added to the postcourse survey to assess resident ratings of the perceived helpfulness of preprovided and in-course materials and content (Table 2). Seven residents responded to these questions. One resident failed to respond to one item, which was replaced with the group mean. For all course materials and content, the mean resident ratings were ≥4, with the precourse materials, case reviews, and procedure simulations receiving the highest ratings.

**Table 2.**
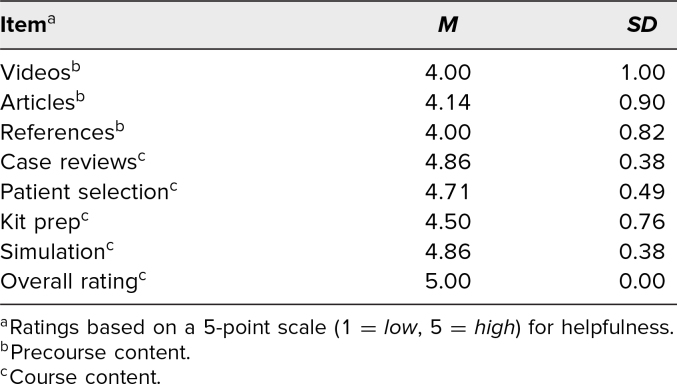
Residents’ Perceived Value of Course Content (*N* = 7)

Table 3 presents the mean percent change in resident ratings of course evaluation items as a function of resident experience (*N* = 31 respondents). While there are trends in the data favoring a particular group based on the item rated, less-experienced residents showed a significantly greater percent change in ratings from pre- to postcourse in terms of their self-perceived ability to perform a chart review (*t* = 2.09, *p* = .045) compared to more experienced residents.

**Table 3.**
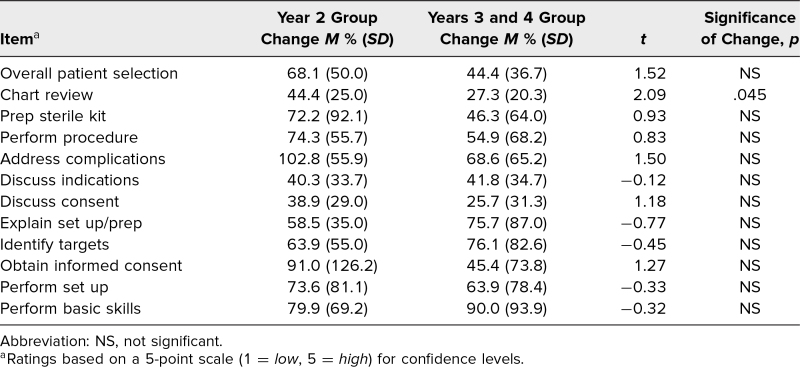
Change in Resident Ratings of Confidence on Each Course Evaluation Item, by Resident Experience (*N* = 31)

### Resident Feedback

As part of the precourse survey, residents were asked to comment on their preferred learning style. Residents consistently reported hands-on learning with many opportunities to practice procedures as one of the best ways to acquire knowledge. Formal lectures, discussions, and self-studying were other preferred learning styles mentioned. Residents also commented that they hoped to improve hands-on procedure skills and increase knowledge of patient selection for procedures, risk identification, and complication management.

Over the 5 years of this interventional spine course offering, residents consistently highlighted that hands-on practice in a low-stress learning environment was a distinct strength of the course. Additional strengths included: informative, easy-to-follow course materials; interactive quizzing; and contiguous sessions allowing information to consolidate. Many residents requested more practice time with the fluoroscopic simulator. The suggestion was often made to expand additional days to the course and/or offer sessions throughout the academic year.

## Discussion

To our knowledge, this is the first shared framework for an interventional spine course with both resident-level didactics and instructions on how to build a low-cost simulation model for hands-on training. The format of this course offers ample time and space for in-depth discussions that cater to residents at different stages of their training. This course provides residents with the foundational knowledge and skills to succeed in any spine procedure rotation.

The initial challenge of learning any procedure begins with becoming familiar with the equipment. Simulated procedures have become commonplace in medical education. Unfortunately, cadavers are increasingly difficult to acquire and costly to maintain. The use of this low-cost spine simulator offers residents an opportunity to handle kits and experience a surprisingly realistic tactile feel while guiding needles to an intended target. Being able to discuss C-arm positions and optimize fluoroscopic images in a patient-free and low-stress manner offers an environment that is seldom available during most residency or fellowship training.

Residents were highly satisfied with the course and expressed that it was helpful in expanding their overall spine care education. We suspect the hands-on kinesthetic feedback in a low-stress setting greatly contributed to these self-reported improvements regarding both kit preparation and procedure performance (see Table 1). Additionally, less-experienced residents perceived chart review education to be more helpful than more-experienced residents (see Table 3). We suspect this reflects how the BIAS method utilized for chart review is a key content point for less-experienced residents, since more-advanced residents may have already been taught and/or already implemented a similar practice in their chart review at the time of course participation.

Based on postcourse surveys and qualitative resident feedback, the course has evolved to offer precourse on-demand video lectures, case review, and a spine image station, which have all been well received. With this newfound confidence, the hope is that attending physicians will be more confident in guiding residents from simulated to real patient procedures.

The course has several areas for improvement. The resident interventional spine course has evolved since its origin in 2018. The initial focus was on providing residents with hands-on skills in a low-stress environment to improve their confidence both in their clinical rotations and in their pursuit of interventional spine/pain fellowships. In its currently packaged form, there is a misalignment between updated learning objectives and prior outcome measures. As the scope of the course and educational materials have expanded, the learning objectives have shifted toward advanced clinical knowledge and procedural skills. There has been a lag in adjusting the outcome measures to truly reflect these new objectives. While measuring learner self-perceived confidence remains important, the next step in this course would be to develop objective measures to quantify improvement as a direct result of this training. For assessment of simulated skills learned in this course, entrustment scales may be appropriate for informed consent, kit preparation, and simulated procedures (Appendices I and J). Crossing over to the clinical setting, entrustment scales may be less helpful, as the potential risks of interventional spine procedures would always require direct supervision, even at a fellowship level. The development of more specific procedural competency assessment tools remains necessary.

Currently, there are no validated and reliable competency assessment tools available for interventional spine/pain procedures at the resident level. With enough participants, an interventional spine course offered across multiple training centers could serve as the basis for developing a valid and reliable competency measure. Furthermore, future longitudinal studies could assess the performance of residents who participate in these courses and monitor their transition to fellowship training and beyond.

The framework and materials in this interventional spine course are meant to be a launching point for local instructors to modify and develop their own educational program that meets the needs of their residents, including continuing to add course content as needed. Instructors could consider alternating the stations within the course in different years to keep the material fresh for residents who are repeating the course.

The supplemental materials do not fully encompass the field of interventional spine procedures. Most of the materials are focused on universal safety concerns that can be identified prior to conducting most spine procedures. The full depth and breadth of knowledge and skills required for interventional spine procedures is beyond the scope of this course. There are several well-known interventional spine textbooks that detail the specifics of performing spine procedures. Local, qualified instructors will obviously bring their own experience and preferences when presenting this course. Some of the supplemental materials might conflict with local departmental policies, and instructors may want to review and edit the slides to best reflect the current standards of practice at their own institution. Instructors should also be aware that national guidelines change regularly, and this lecture may need to be updated periodically. Consider reviewing the International Pain and Spine Interventional Society, American Society of Regional Anesthesia Pain Medicine, and North American Spine Society websites for the latest national guidelines.^12–14^

## Appendices


Overview - Spine.pptxPrep Kit Materials.docxBuilding a Low-Cost Spine Simulator.pptxFacilitators Guide.docxSpine Procedure - Guidelines Lecture.pptxSpine Procedure Guidelines Lecture Video.mp4Course Chart Review Guidelines.docxSpine Course - Cases.pptxChart Review Preprocedures Checklist.docxInformed Consent and Procedure Timeout Checklist.docxLumbar Procedure Table Checklist.docxProcedure Descriptions.docxFluoroscopic Spine Procedure Images.pptxSpine Course Pre-Post Survey - Updated.docxSpine Course Pre-Post Survey - Original.docx
*All appendices are peer reviewed as integral parts of the Original Publication.*
