# Supplementary material for: Resident Interventional Spine Course with Didactics and Hands-On Skills Lab
Source: MedEdPORTAL. 2025 Oct 7;21:11551. doi: 10.15766/mep_2374-8265.11551 (PMC12502988; doi:10.15766/mep_2374-8265.11551)
Supplement: Supplementary file 1 — Overview - Spine.pptxPrep Kit Materials.docxBuilding a Low-Cost Spine Simulator.pptxFacilitators Guide.docxSpine Procedure - Guidelines Lecture.pptxSpine Procedure Guidelines Lecture Video.mp4Course Chart Review Guidelines.docxSpine Course - Cases.pptxChart Review Preprocedures Checklist.docxInformed Consent and Procedure Timeout Checklist.docxLumbar Procedure Table Checklist.docxProcedure Descriptions.docxFluoroscopic Spine Procedure Images.pptxSpine Course Pre-Post Survey - Updated.docxSpine Course Pre-Post Survey - Original.docx [file mep_2374-8265.11551-s001.zip › A. Overview - Spine.pptx]

## Slide 1
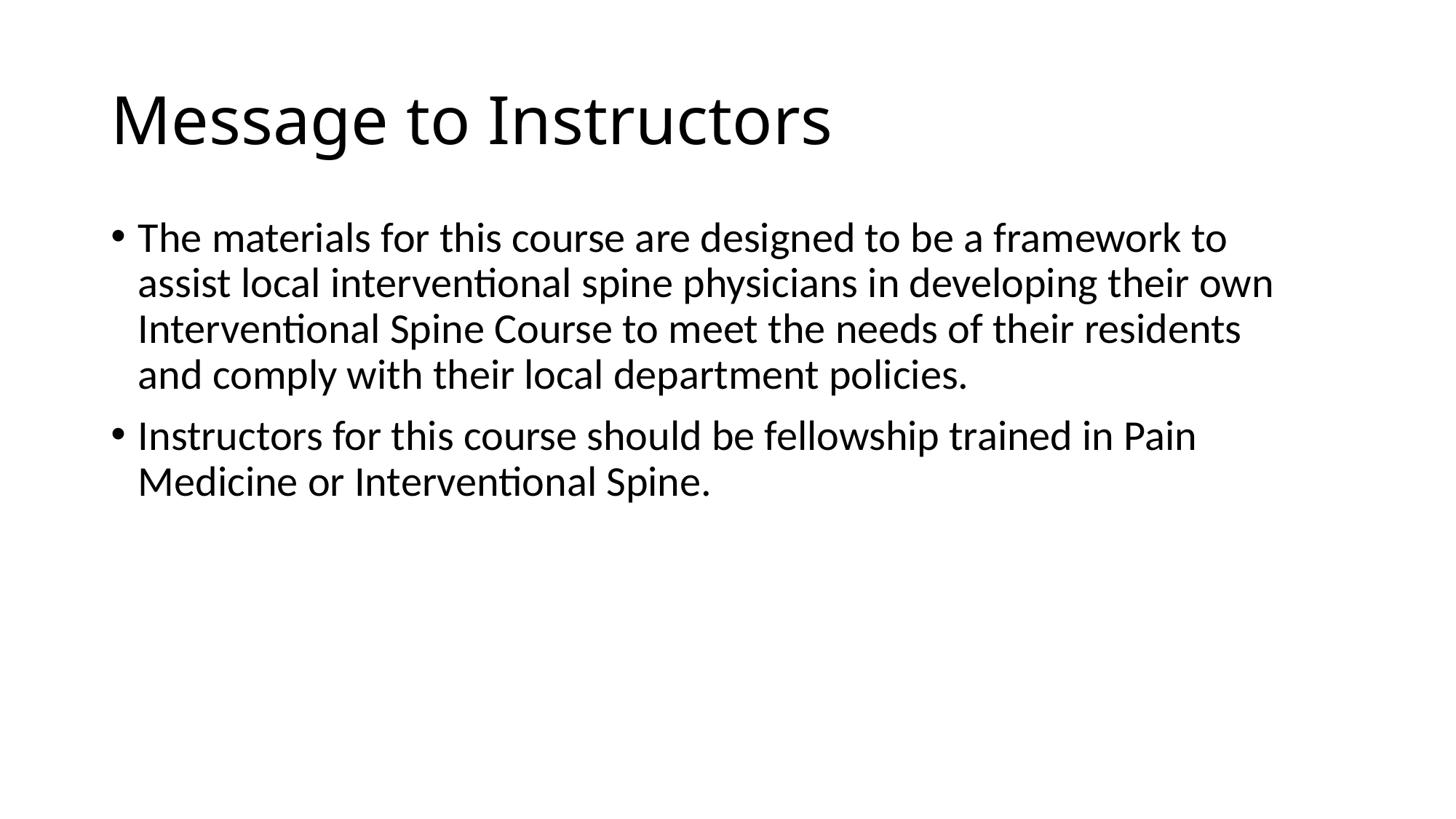

# Message to Instructors
The materials for this course are designed to be a framework to assist local interventional spine physicians in developing their own Interventional Spine Course to meet the needs of their residents and comply with their local department policies.
Instructors for this course should be fellowship trained in Pain Medicine or Interventional Spine.

## Slide 2
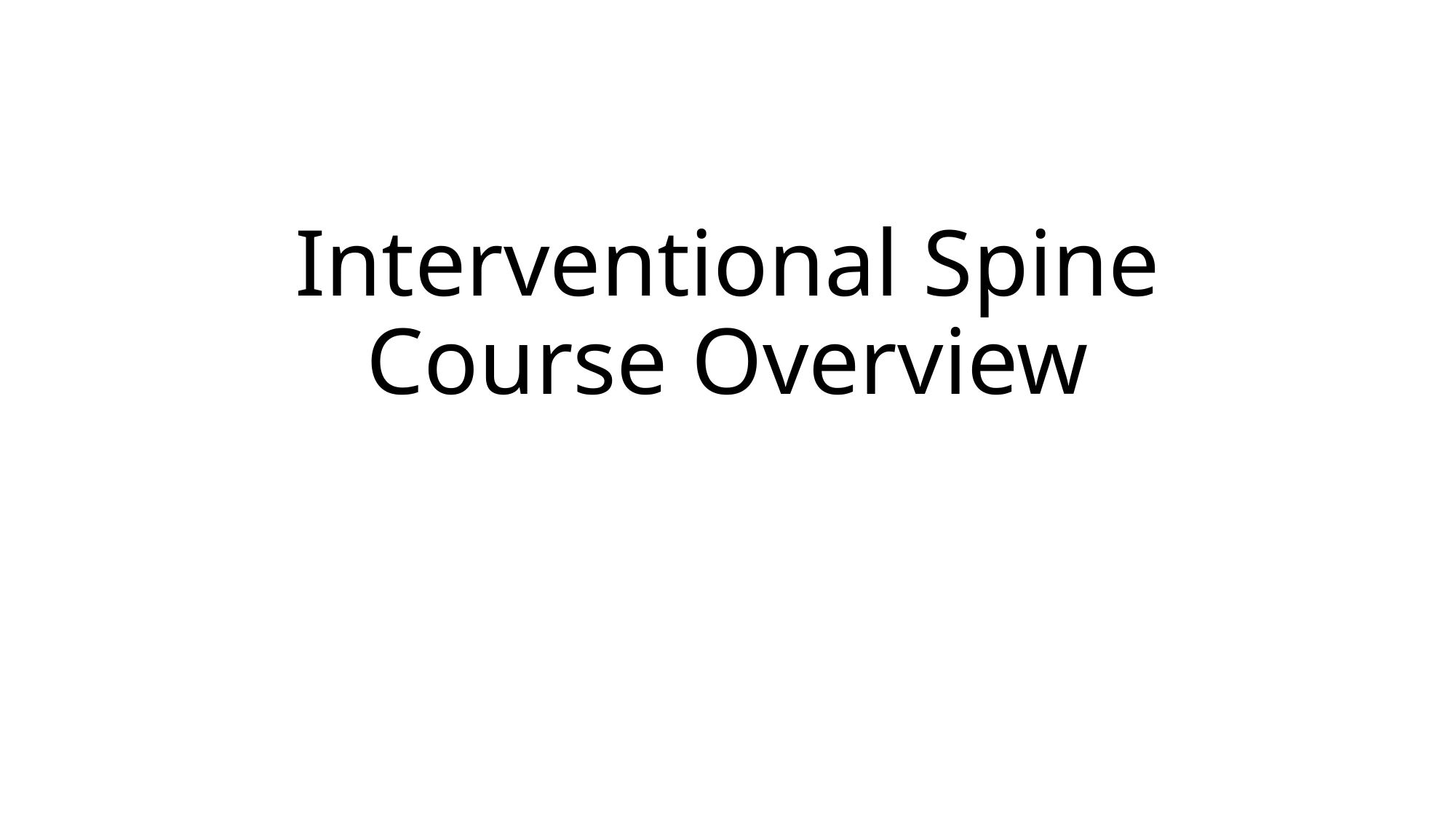

# Interventional Spine Course Overview

## Slide 3
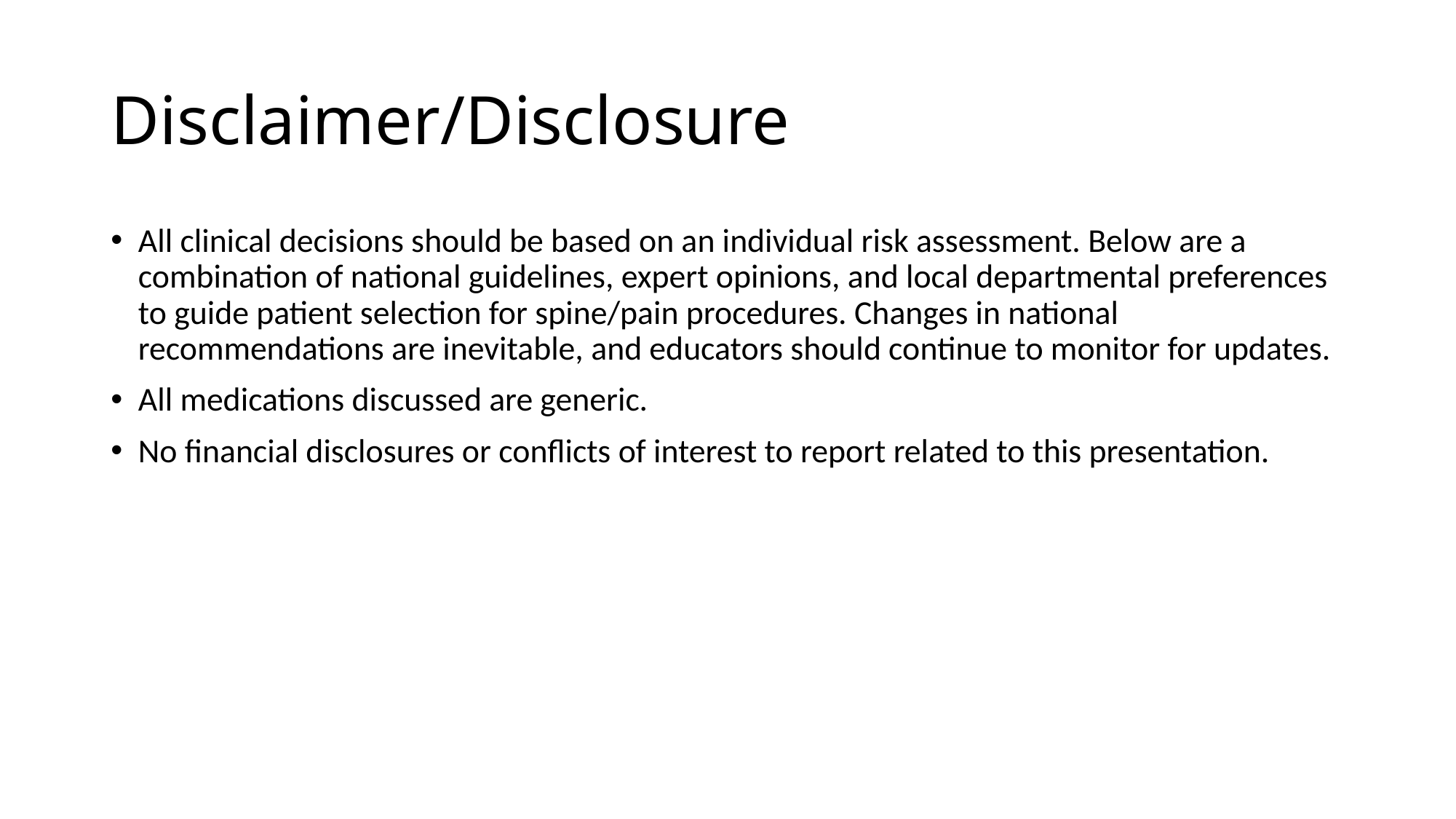

# Disclaimer/Disclosure
All clinical decisions should be based on an individual risk assessment. Below are a combination of national guidelines, expert opinions, and local departmental preferences to guide patient selection for spine/pain procedures. Changes in national recommendations are inevitable, and educators should continue to monitor for updates.
All medications discussed are generic.
No financial disclosures or conflicts of interest to report related to this presentation.

## Slide 4
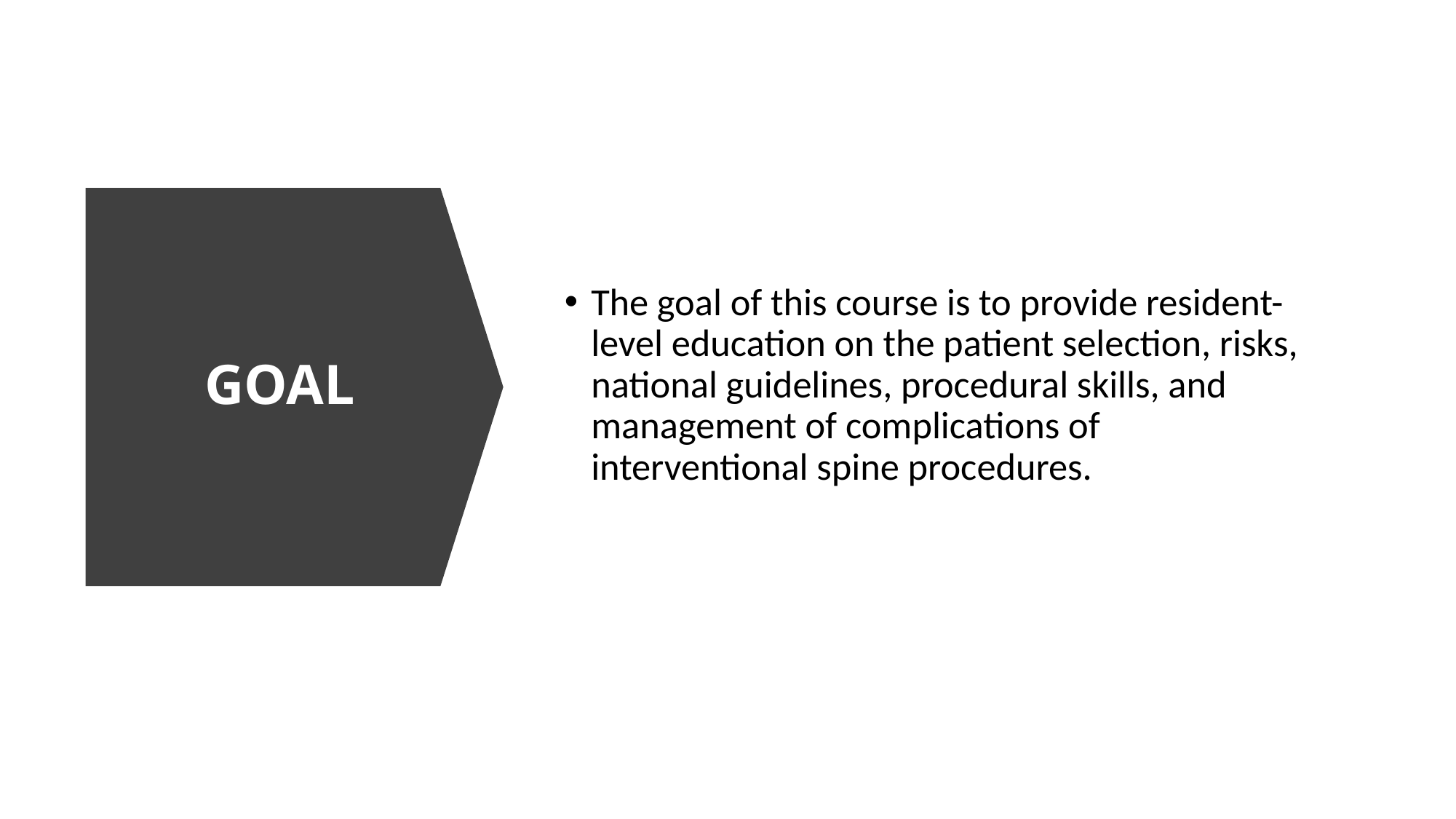

GOAL
The goal of this course is to provide resident-level education on the patient selection, risks, national guidelines, procedural skills, and management of complications of interventional spine procedures.

## Slide 5
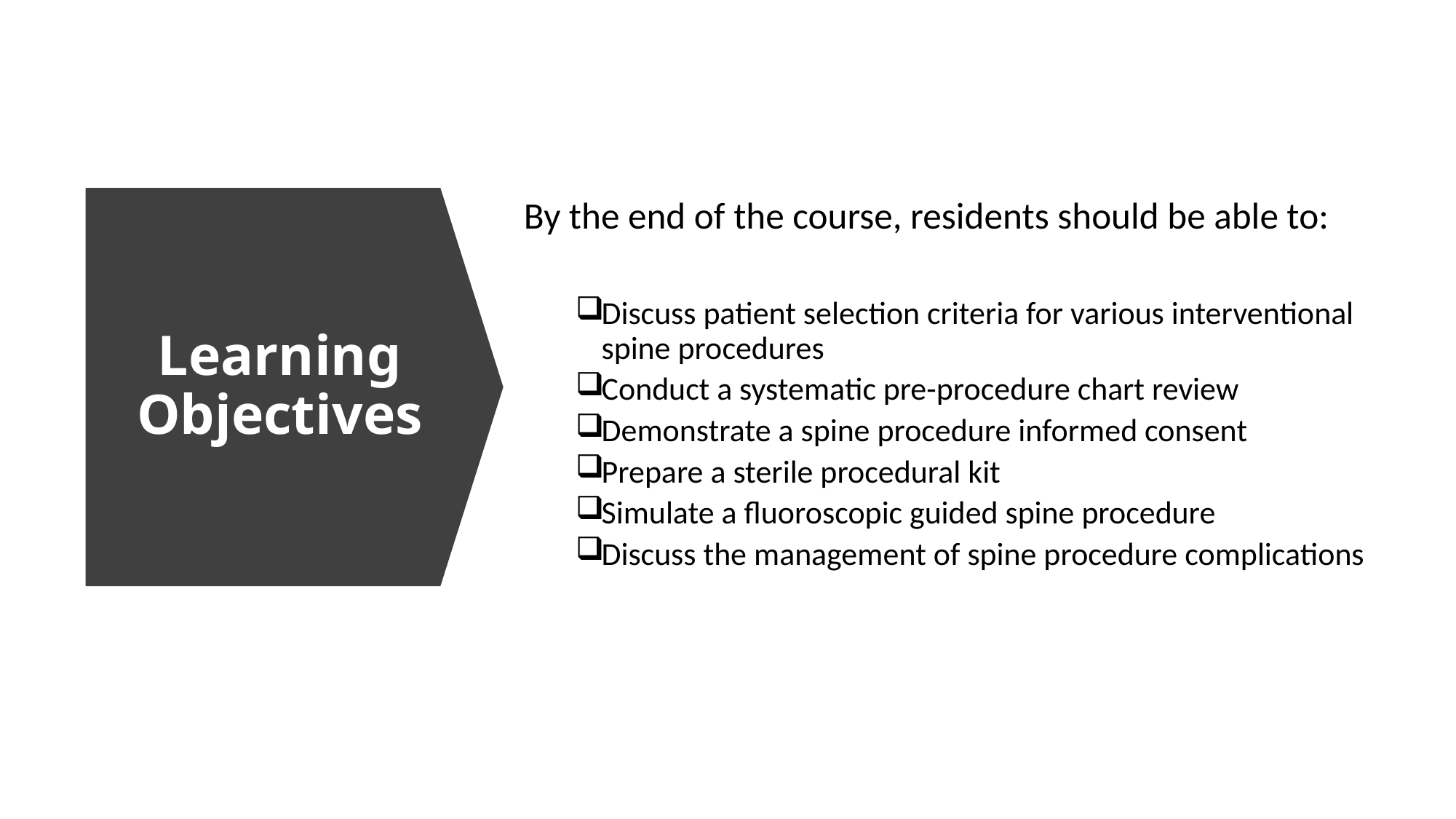

By the end of the course, residents should be able to:
Discuss patient selection criteria for various interventional spine procedures
Conduct a systematic pre-procedure chart review
Demonstrate a spine procedure informed consent
Prepare a sterile procedural kit
Simulate a fluoroscopic guided spine procedure
Discuss the management of spine procedure complications
Learning Objectives

## Slide 6
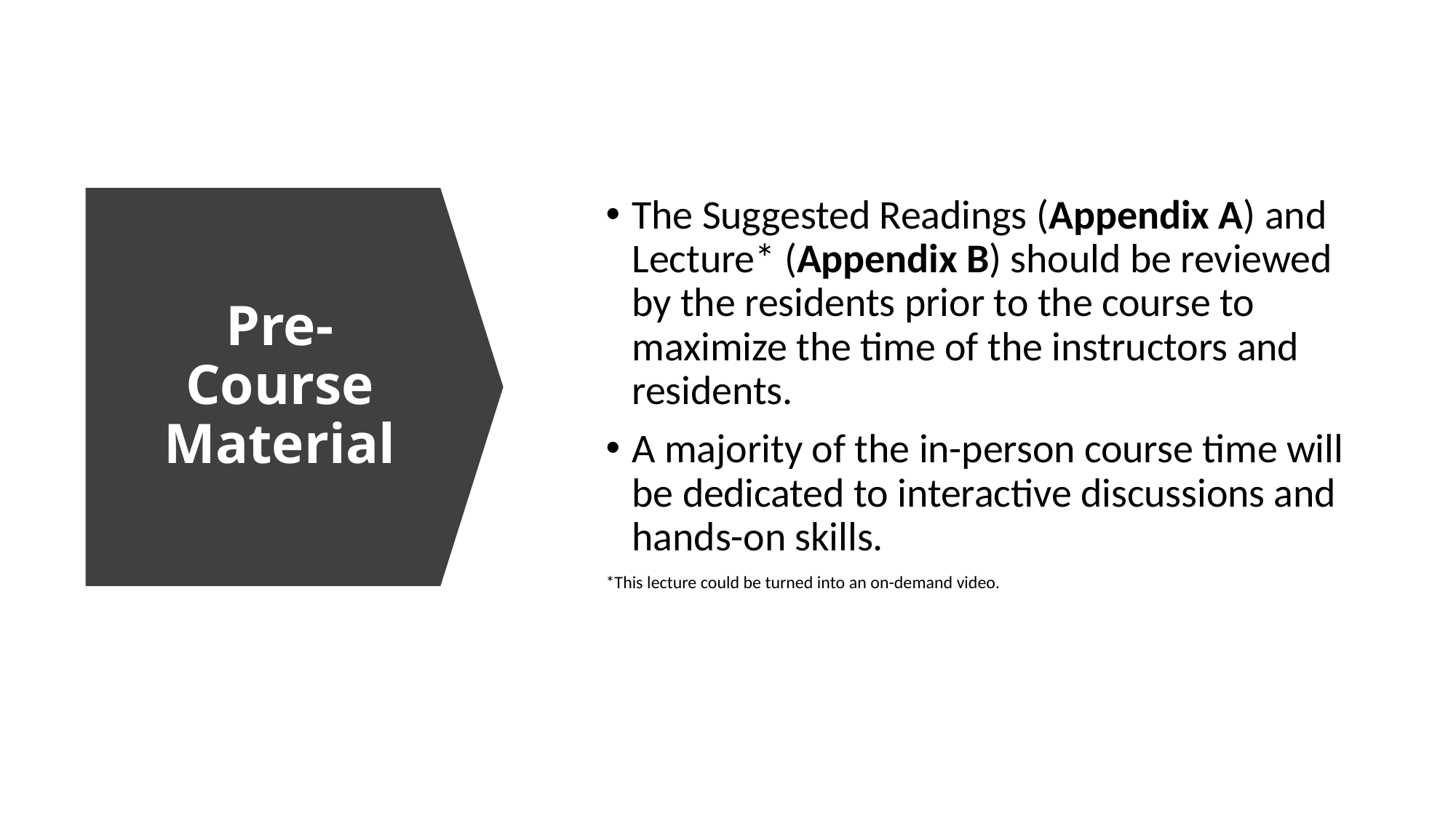

The Suggested Readings (Appendix A) and Lecture* (Appendix B) should be reviewed by the residents prior to the course to maximize the time of the instructors and residents.
A majority of the in-person course time will be dedicated to interactive discussions and hands-on skills.
*This lecture could be turned into an on-demand video.
Pre-Course Material

## Slide 7
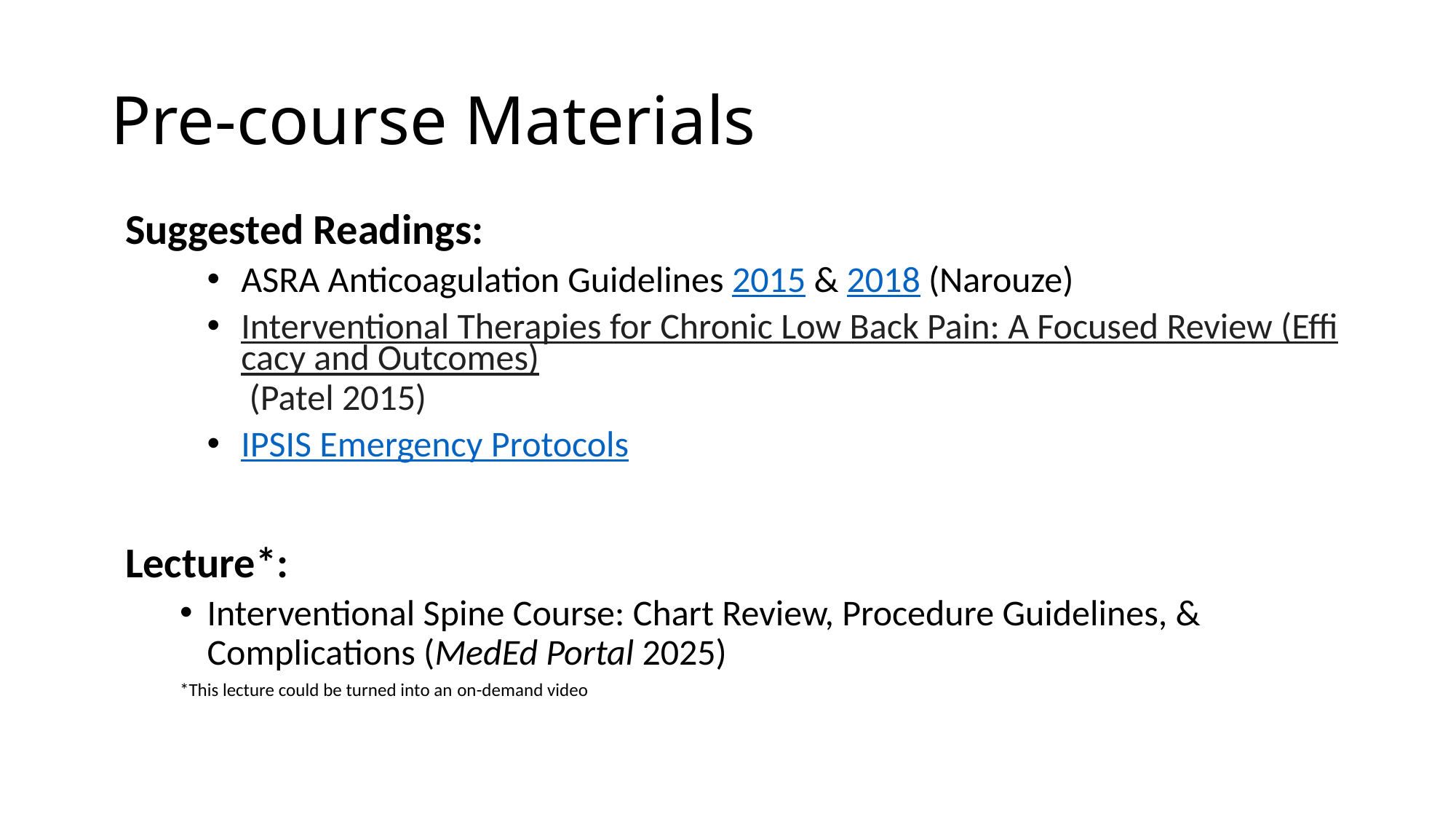

# Pre-course Materials
Suggested Readings:
ASRA Anticoagulation Guidelines 2015 & 2018 (Narouze)
Interventional Therapies for Chronic Low Back Pain: A Focused Review (Efficacy and Outcomes) (Patel 2015)
IPSIS Emergency Protocols
Lecture*:
Interventional Spine Course: Chart Review, Procedure Guidelines, & Complications (MedEd Portal 2025)
*This lecture could be turned into an on-demand video
