# Supplementary material for: Resident Interventional Spine Course with Didactics and Hands-On Skills Lab
Source: MedEdPORTAL. 2025 Oct 7;21:11551. doi: 10.15766/mep_2374-8265.11551 (PMC12502988; doi:10.15766/mep_2374-8265.11551)
Supplement: Supplementary file 1 — Overview - Spine.pptxPrep Kit Materials.docxBuilding a Low-Cost Spine Simulator.pptxFacilitators Guide.docxSpine Procedure - Guidelines Lecture.pptxSpine Procedure Guidelines Lecture Video.mp4Course Chart Review Guidelines.docxSpine Course - Cases.pptxChart Review Preprocedures Checklist.docxInformed Consent and Procedure Timeout Checklist.docxLumbar Procedure Table Checklist.docxProcedure Descriptions.docxFluoroscopic Spine Procedure Images.pptxSpine Course Pre-Post Survey - Updated.docxSpine Course Pre-Post Survey - Original.docx [file mep_2374-8265.11551-s001.zip › B. Prep Kit Materials.docx]

**Appendix B. Sample Procedure Kit Material List:**

- 1- Tray styrene procedural
- 2- Lidocaine 1% 5ml vials
- 1- Hypodermic needle 27Gx1.25
- 1- 18Gx1.5 needle
- 1- 19gx1.5 filter needle
- 1- 12 minibore extension line
- 1- 3cc plastic syringe luer lock
- 1- 5cc plastic syringe luer lock
- 1- 10cc plastic syringe luer lock
- 1- Needle stick pad
- 1- 22”x28”, 4”x3” oval, tissue- poly, blue, no tape
- 1- 17x19 white towel
- 4- Gauze pads 4x4
- 1- Chlorhexidine stick
