# Supplementary material for: Resident Interventional Spine Course with Didactics and Hands-On Skills Lab
Source: MedEdPORTAL. 2025 Oct 7;21:11551. doi: 10.15766/mep_2374-8265.11551 (PMC12502988; doi:10.15766/mep_2374-8265.11551)
Supplement: Supplementary file 1 — Overview - Spine.pptxPrep Kit Materials.docxBuilding a Low-Cost Spine Simulator.pptxFacilitators Guide.docxSpine Procedure - Guidelines Lecture.pptxSpine Procedure Guidelines Lecture Video.mp4Course Chart Review Guidelines.docxSpine Course - Cases.pptxChart Review Preprocedures Checklist.docxInformed Consent and Procedure Timeout Checklist.docxLumbar Procedure Table Checklist.docxProcedure Descriptions.docxFluoroscopic Spine Procedure Images.pptxSpine Course Pre-Post Survey - Updated.docxSpine Course Pre-Post Survey - Original.docx [file mep_2374-8265.11551-s001.zip › C. Building a Low-Cost Spine Simulator.pptx]

## Slide 1
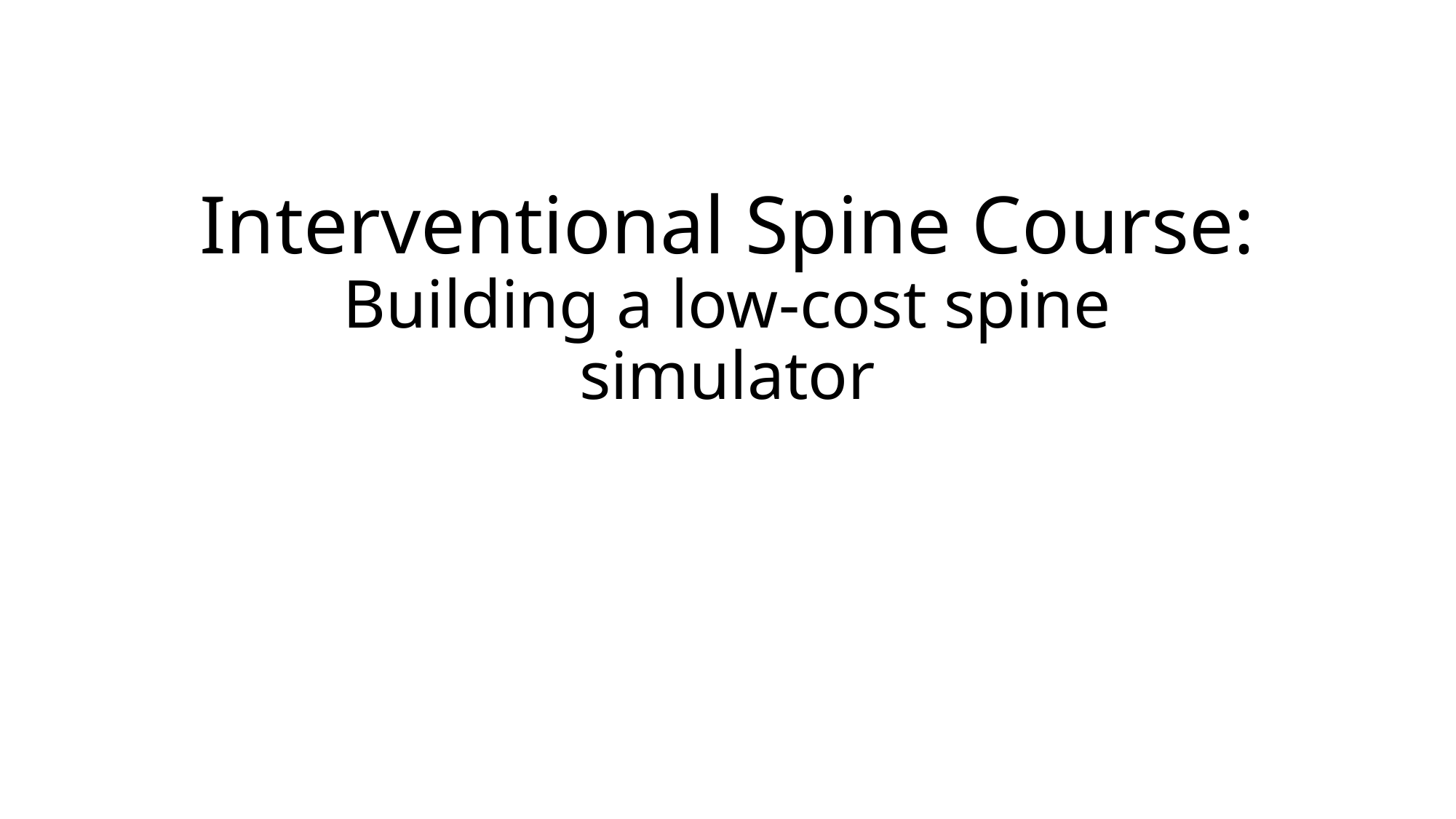

# Interventional Spine Course: Building a low-cost spine simulator

## Slide 2
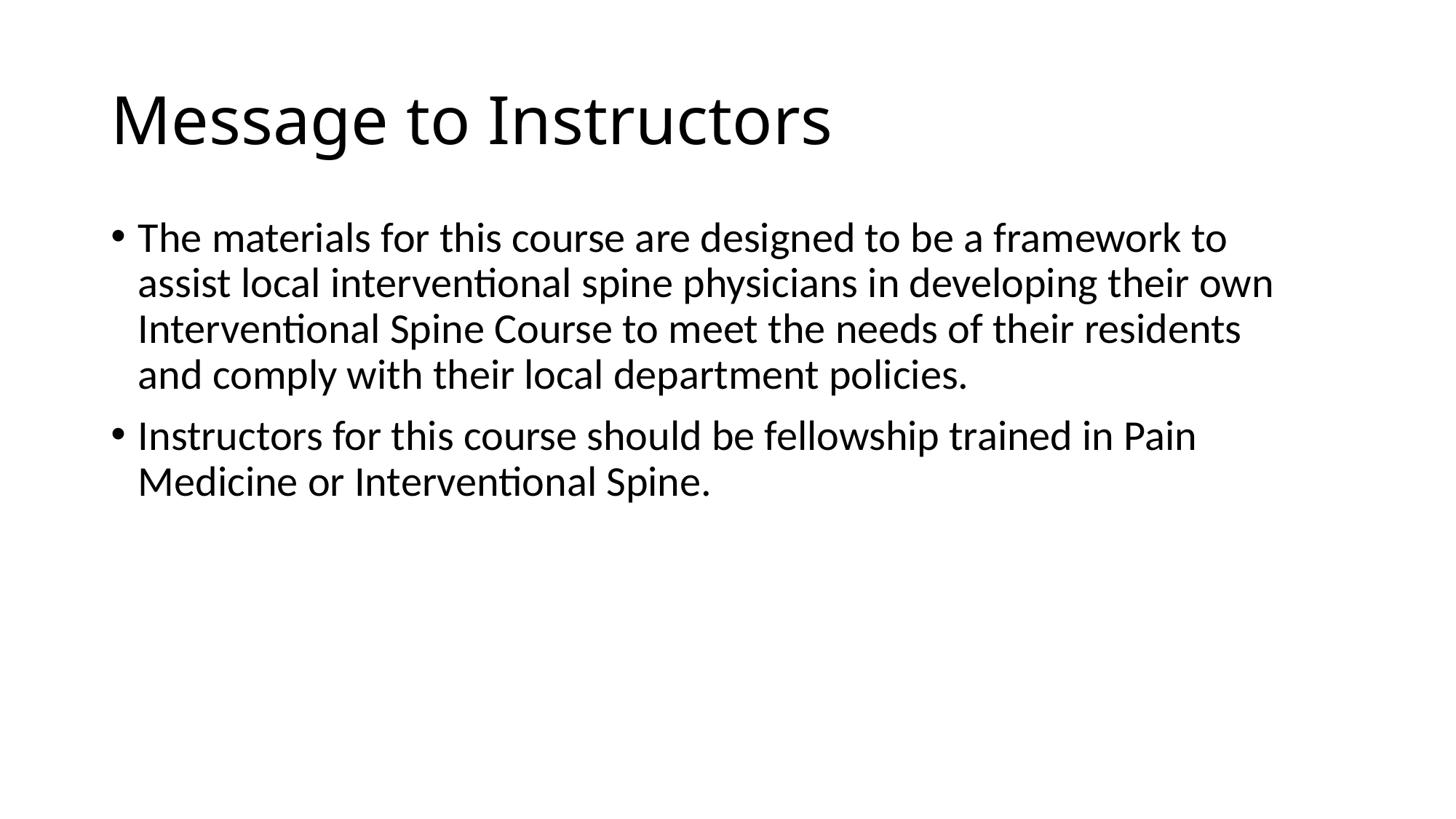

# Message to Instructors
The materials for this course are designed to be a framework to assist local interventional spine physicians in developing their own Interventional Spine Course to meet the needs of their residents and comply with their local department policies.
Instructors for this course should be fellowship trained in Pain Medicine or Interventional Spine.

## Slide 3
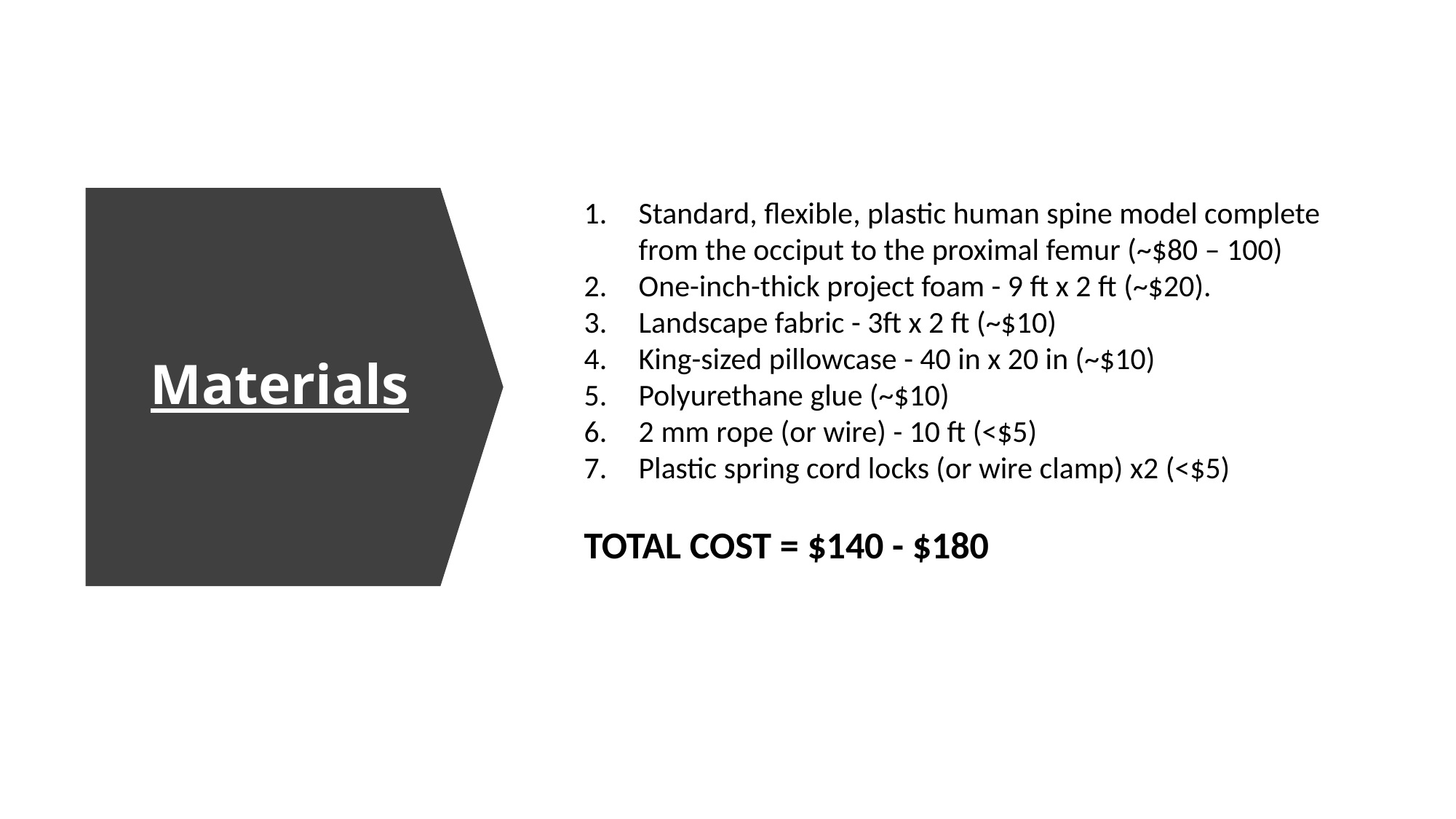

Standard, flexible, plastic human spine model complete from the occiput to the proximal femur (~$80 – 100)
One-inch-thick project foam - 9 ft x 2 ft (~$20).
Landscape fabric - 3ft x 2 ft (~$10)
King-sized pillowcase - 40 in x 20 in (~$10)
Polyurethane glue (~$10)
2 mm rope (or wire) - 10 ft (<$5)
Plastic spring cord locks (or wire clamp) x2 (<$5)
TOTAL COST = $140 - $180
# Materials

## Slide 4
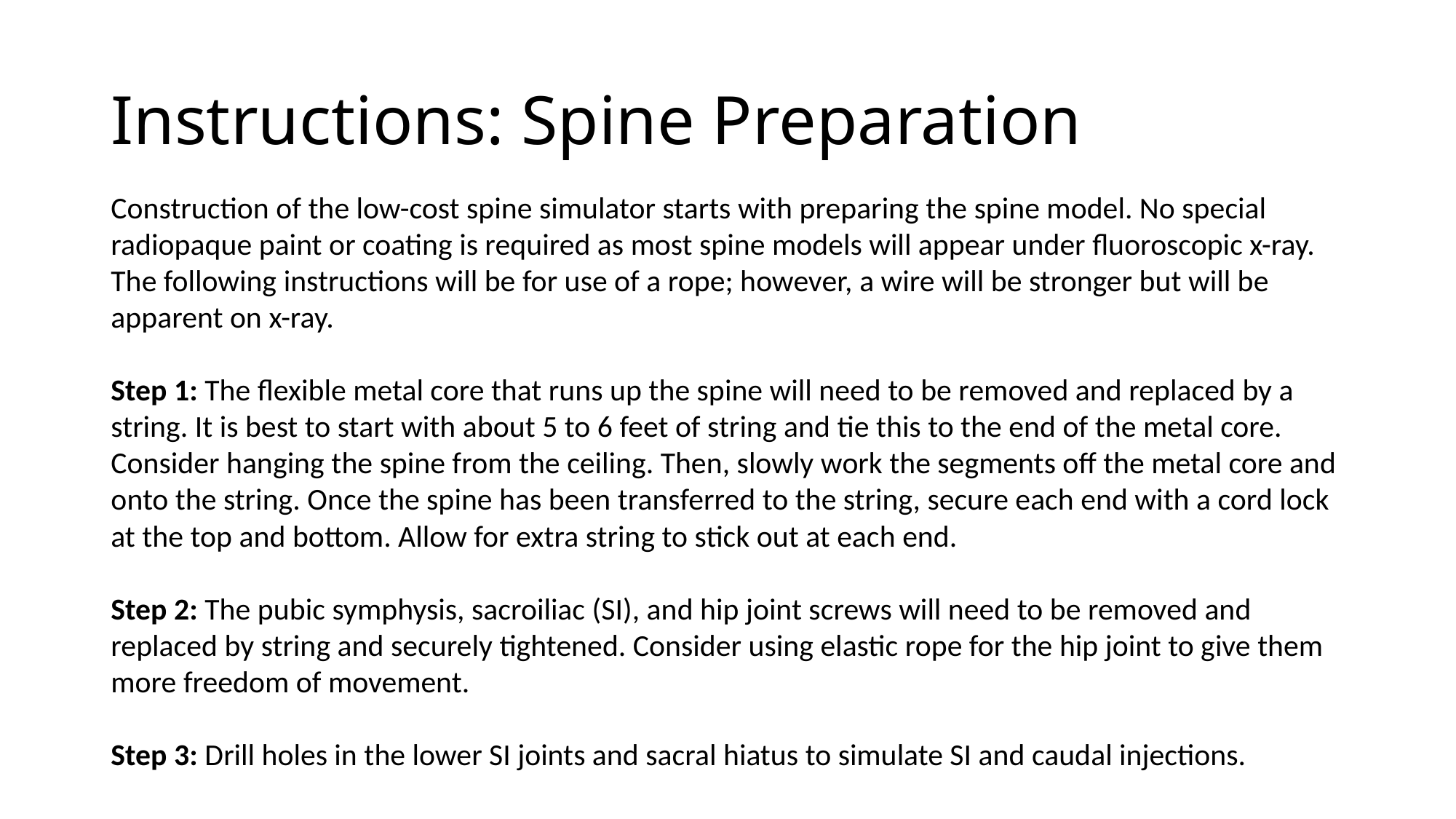

# Instructions: Spine Preparation
Construction of the low-cost spine simulator starts with preparing the spine model. No special radiopaque paint or coating is required as most spine models will appear under fluoroscopic x-ray. The following instructions will be for use of a rope; however, a wire will be stronger but will be apparent on x-ray.
Step 1: The flexible metal core that runs up the spine will need to be removed and replaced by a string. It is best to start with about 5 to 6 feet of string and tie this to the end of the metal core. Consider hanging the spine from the ceiling. Then, slowly work the segments off the metal core and onto the string. Once the spine has been transferred to the string, secure each end with a cord lock at the top and bottom. Allow for extra string to stick out at each end.
Step 2: The pubic symphysis, sacroiliac (SI), and hip joint screws will need to be removed and replaced by string and securely tightened. Consider using elastic rope for the hip joint to give them more freedom of movement.
Step 3: Drill holes in the lower SI joints and sacral hiatus to simulate SI and caudal injections.

## Slide 5
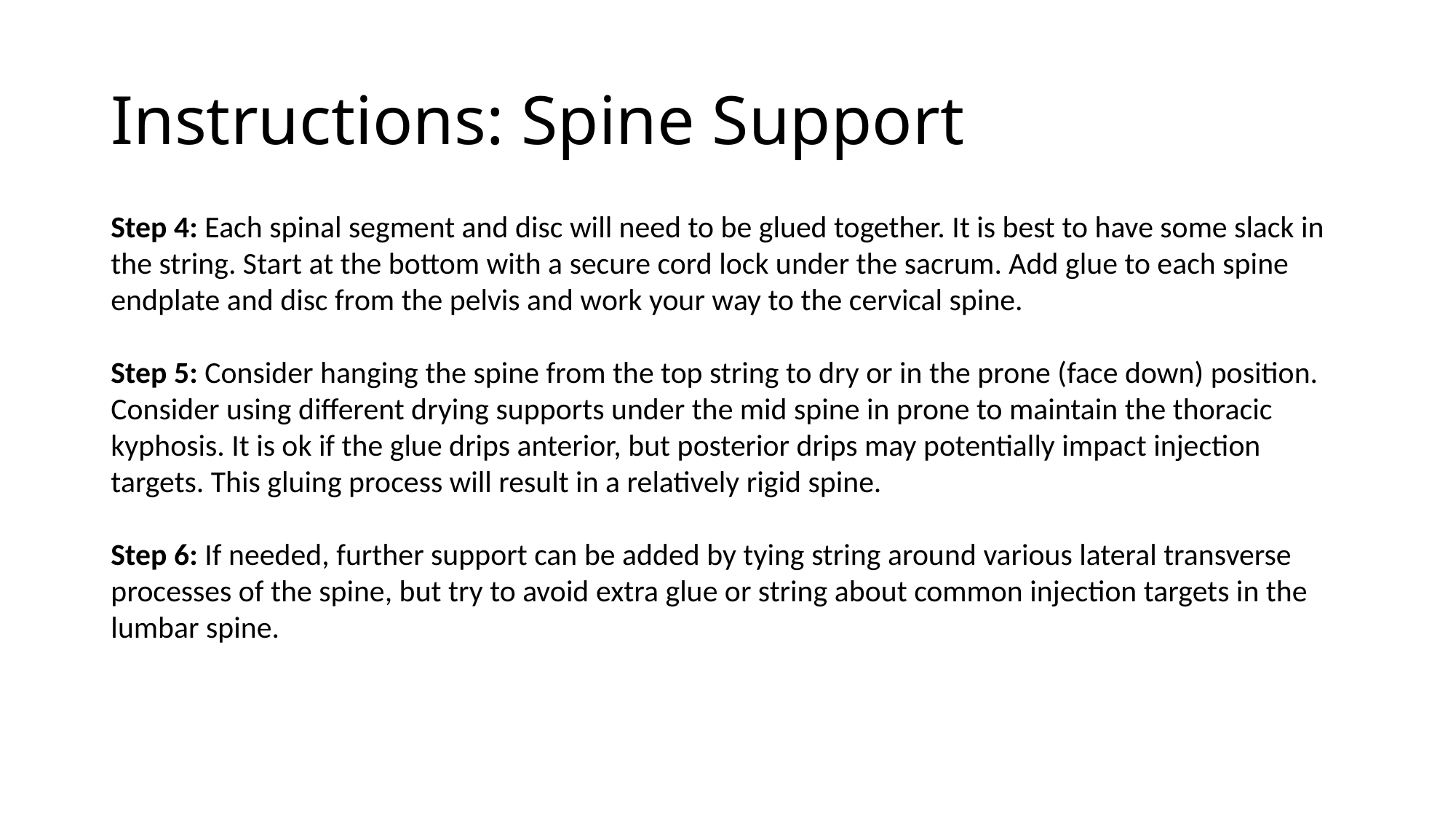

# Instructions: Spine Support
Step 4: Each spinal segment and disc will need to be glued together. It is best to have some slack in the string. Start at the bottom with a secure cord lock under the sacrum. Add glue to each spine endplate and disc from the pelvis and work your way to the cervical spine.
Step 5: Consider hanging the spine from the top string to dry or in the prone (face down) position. Consider using different drying supports under the mid spine in prone to maintain the thoracic kyphosis. It is ok if the glue drips anterior, but posterior drips may potentially impact injection targets. This gluing process will result in a relatively rigid spine.
Step 6: If needed, further support can be added by tying string around various lateral transverse processes of the spine, but try to avoid extra glue or string about common injection targets in the lumbar spine.

## Slide 6
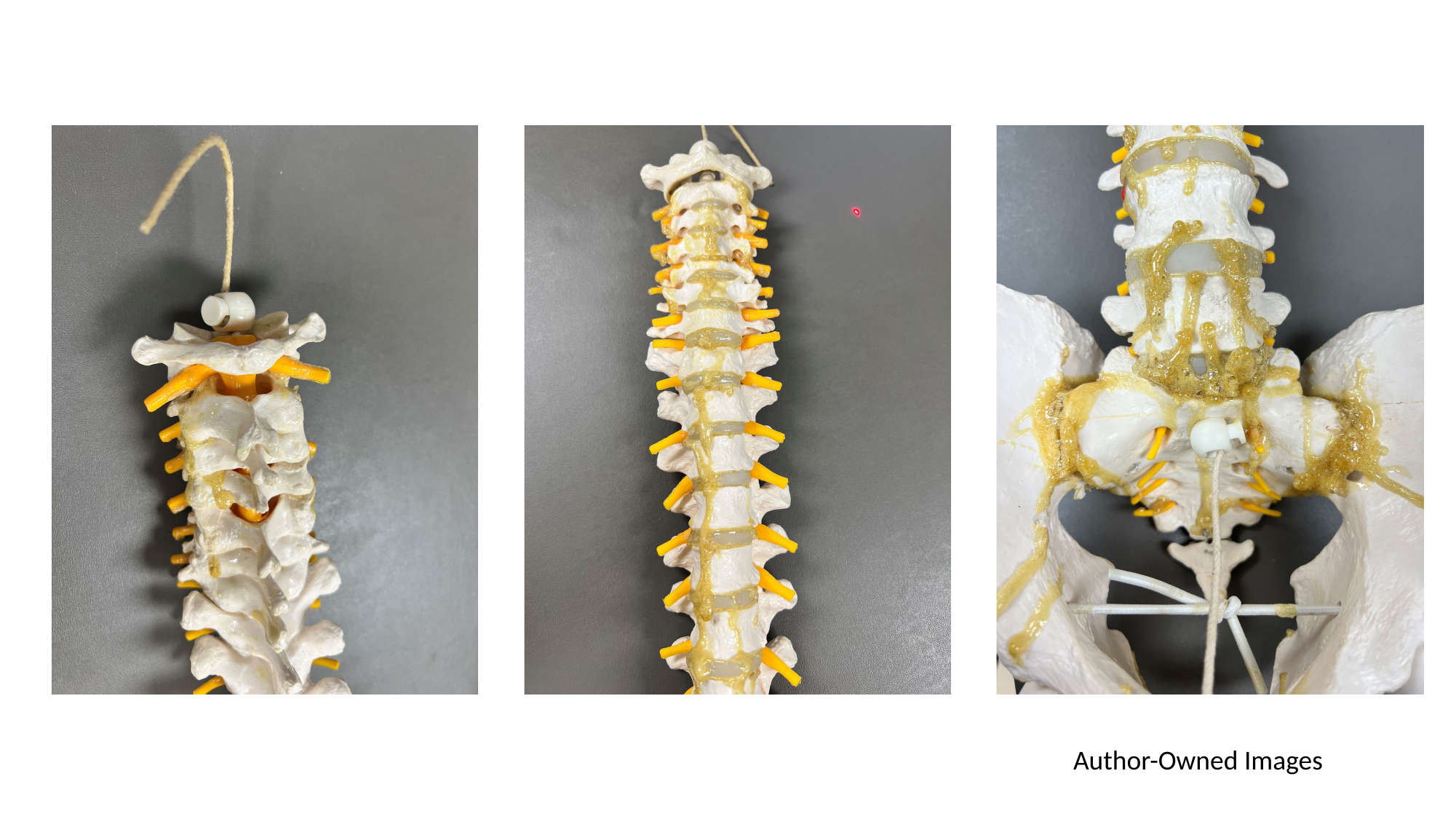

Author-Owned Images

## Slide 7
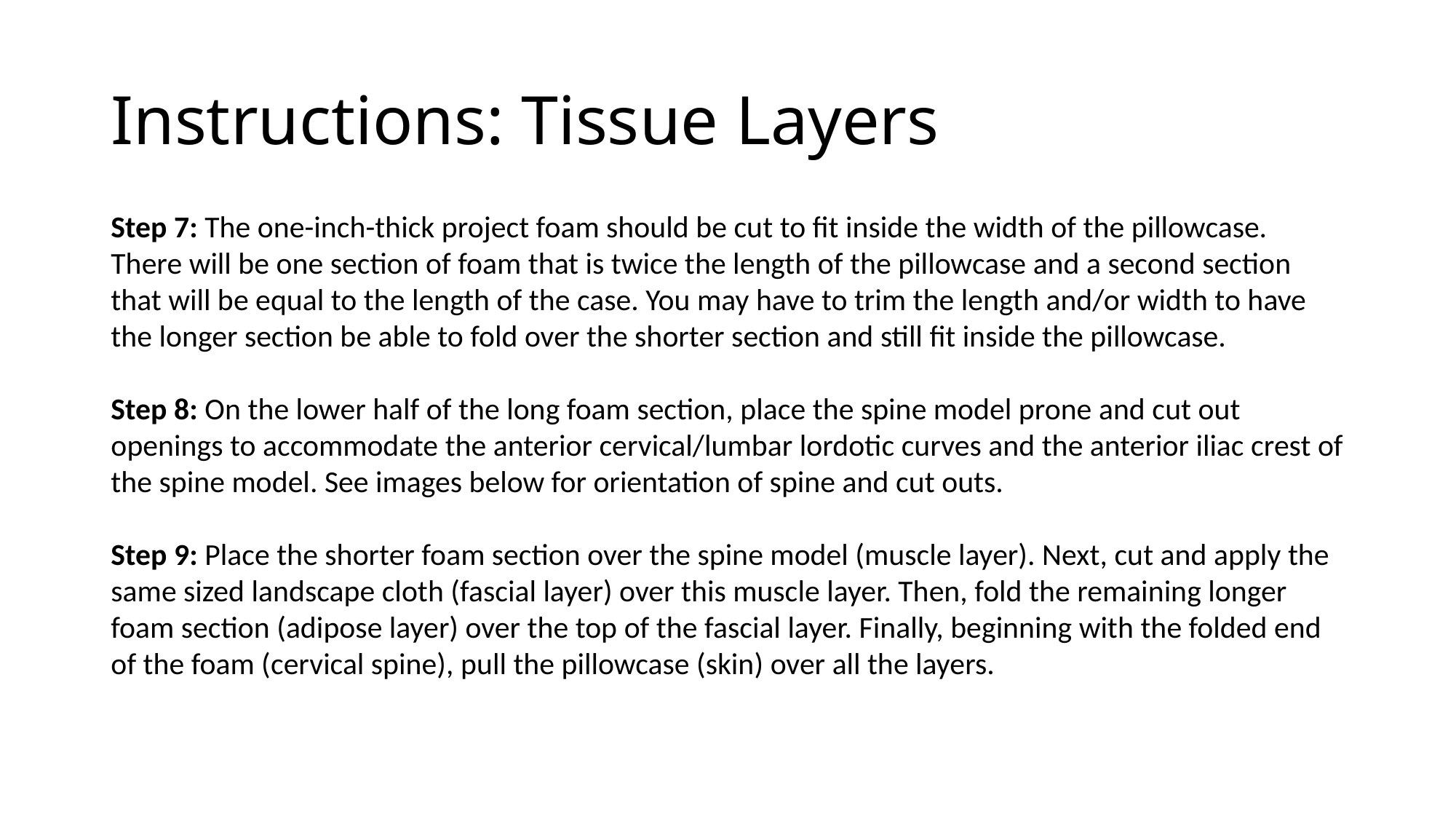

# Instructions: Tissue Layers
Step 7: The one-inch-thick project foam should be cut to fit inside the width of the pillowcase. There will be one section of foam that is twice the length of the pillowcase and a second section that will be equal to the length of the case. You may have to trim the length and/or width to have the longer section be able to fold over the shorter section and still fit inside the pillowcase.
Step 8: On the lower half of the long foam section, place the spine model prone and cut out openings to accommodate the anterior cervical/lumbar lordotic curves and the anterior iliac crest of the spine model. See images below for orientation of spine and cut outs.
Step 9: Place the shorter foam section over the spine model (muscle layer). Next, cut and apply the same sized landscape cloth (fascial layer) over this muscle layer. Then, fold the remaining longer foam section (adipose layer) over the top of the fascial layer. Finally, beginning with the folded end of the foam (cervical spine), pull the pillowcase (skin) over all the layers.

## Slide 8
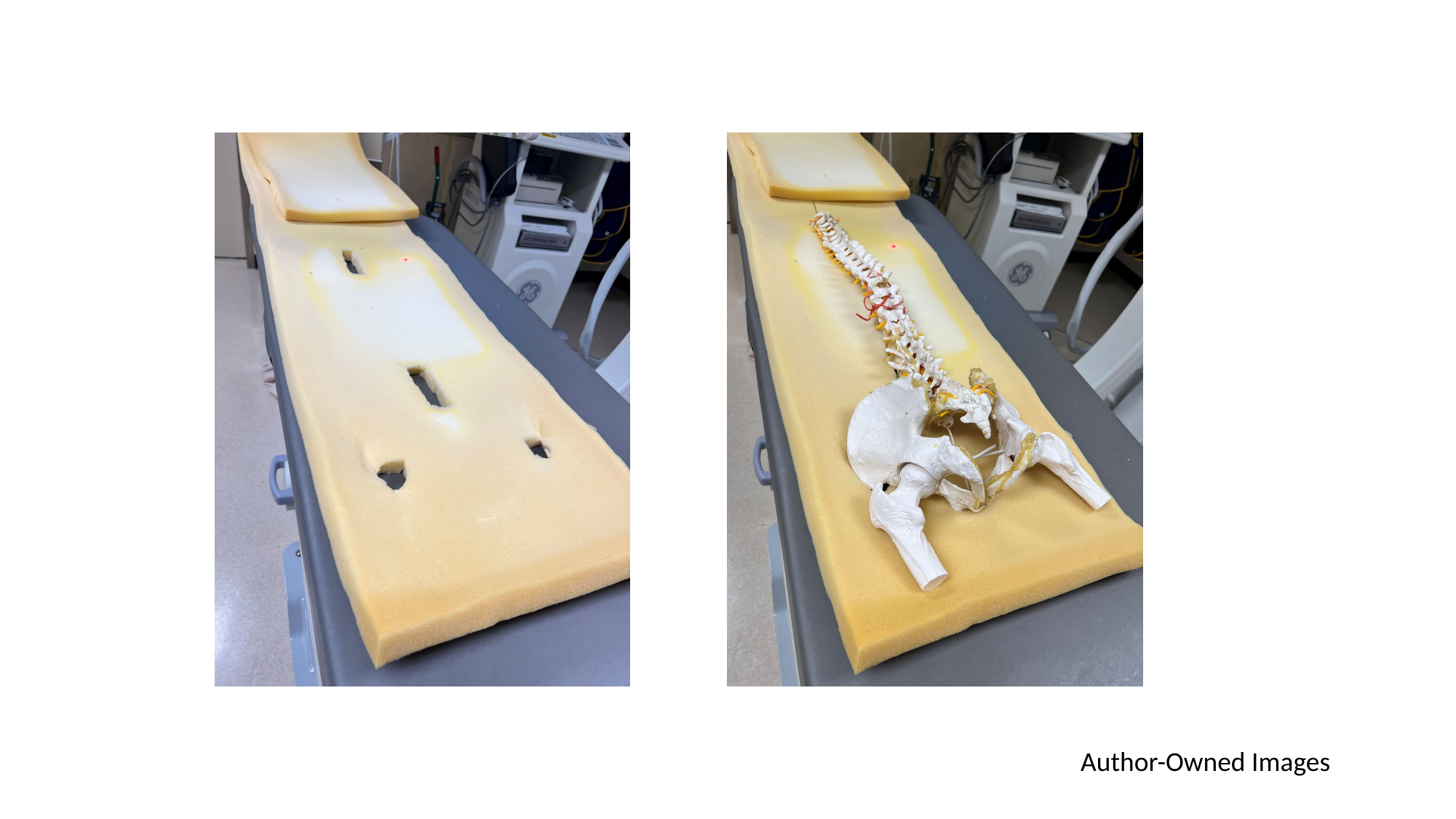

Author-Owned Images

## Slide 9
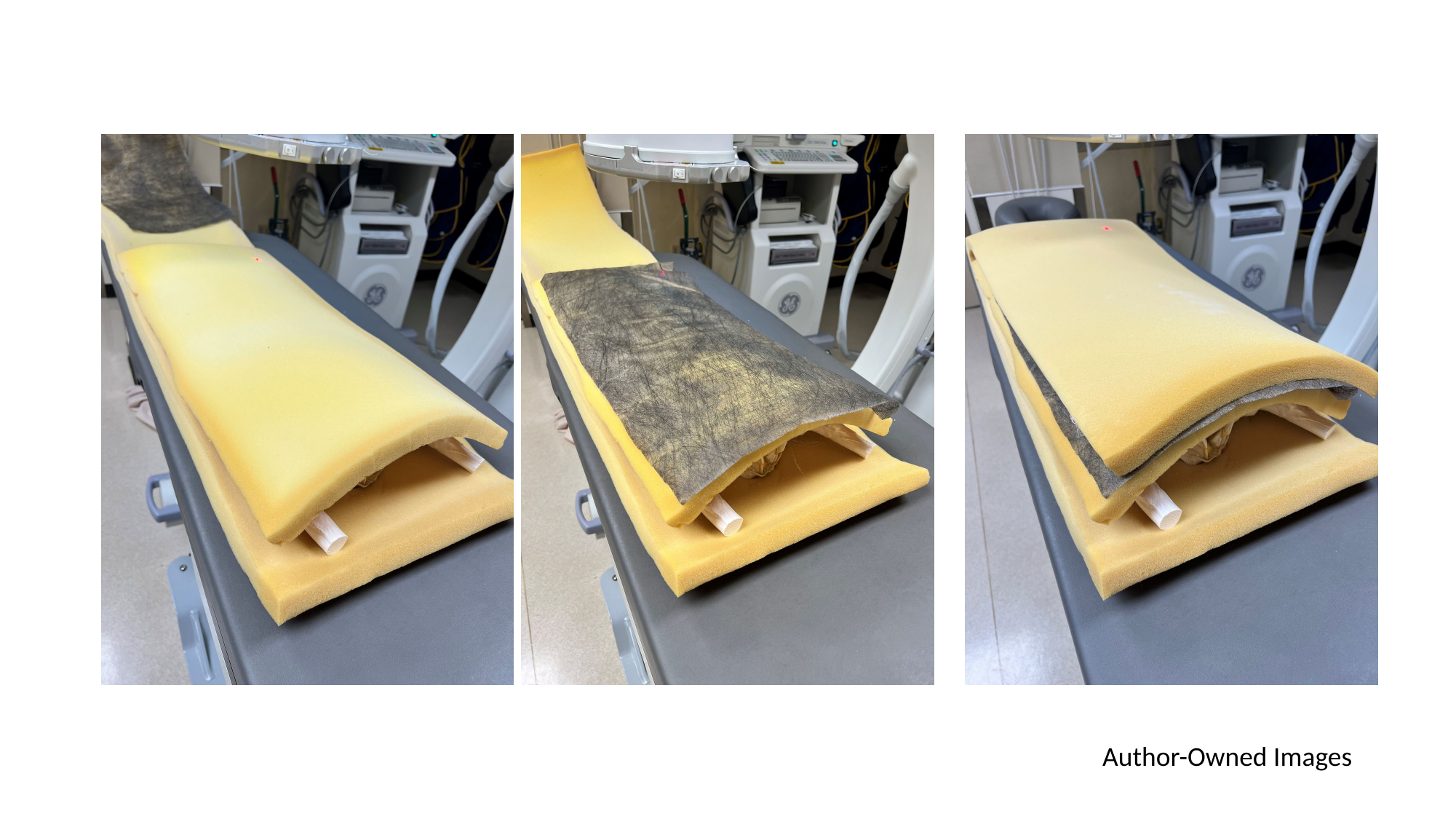

Author-Owned Images

## Slide 10
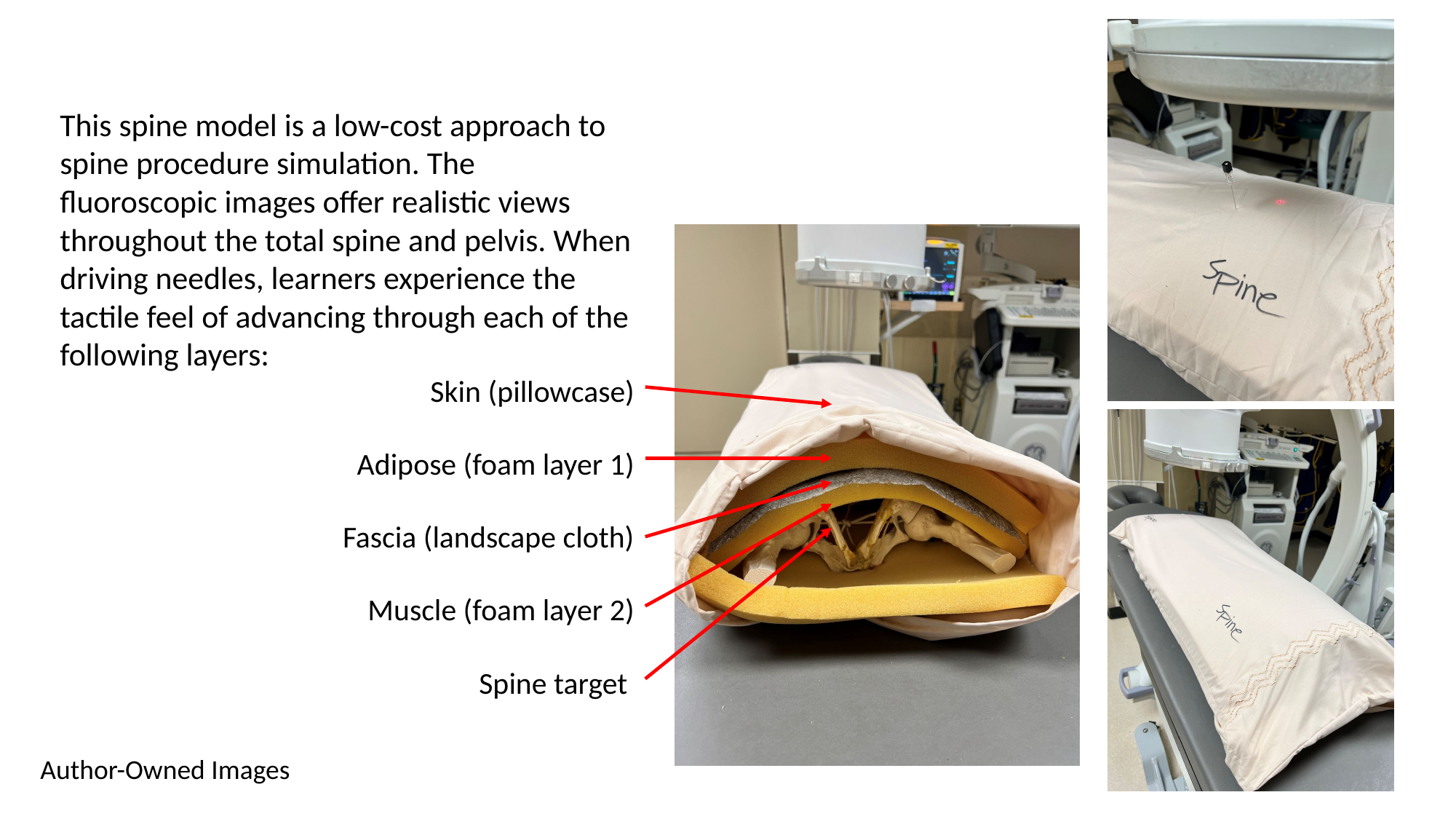

This spine model is a low-cost approach to spine procedure simulation. The fluoroscopic images offer realistic views throughout the total spine and pelvis. When driving needles, learners experience the tactile feel of advancing through each of the following layers:
	Skin (pillowcase)
	Adipose (foam layer 1)
	Fascia (landscape cloth)
	Muscle (foam layer 2)
	Spine target
Author-Owned Images

## Slide 11
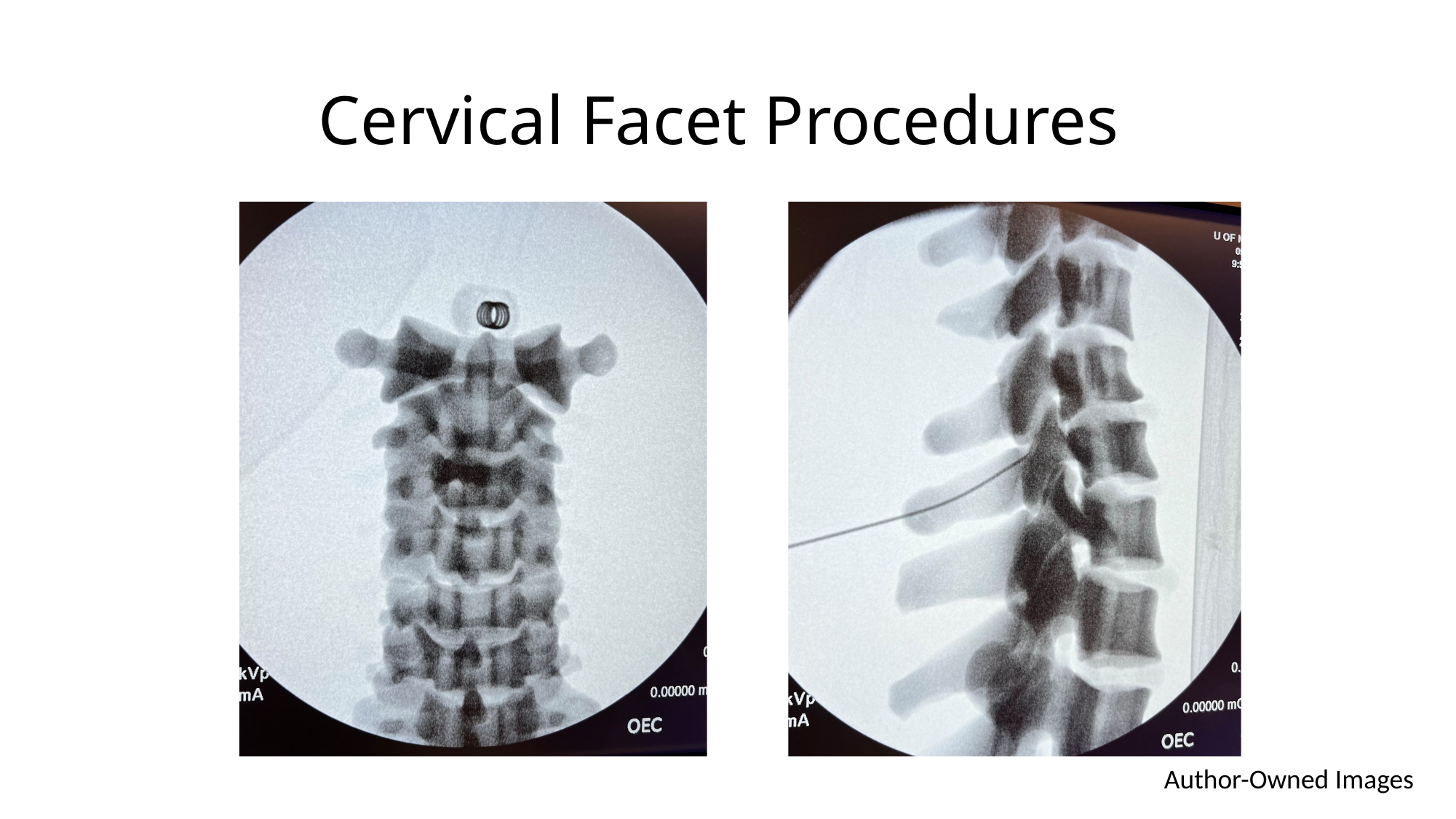

# Cervical Facet Procedures
Author-Owned Images

## Slide 12
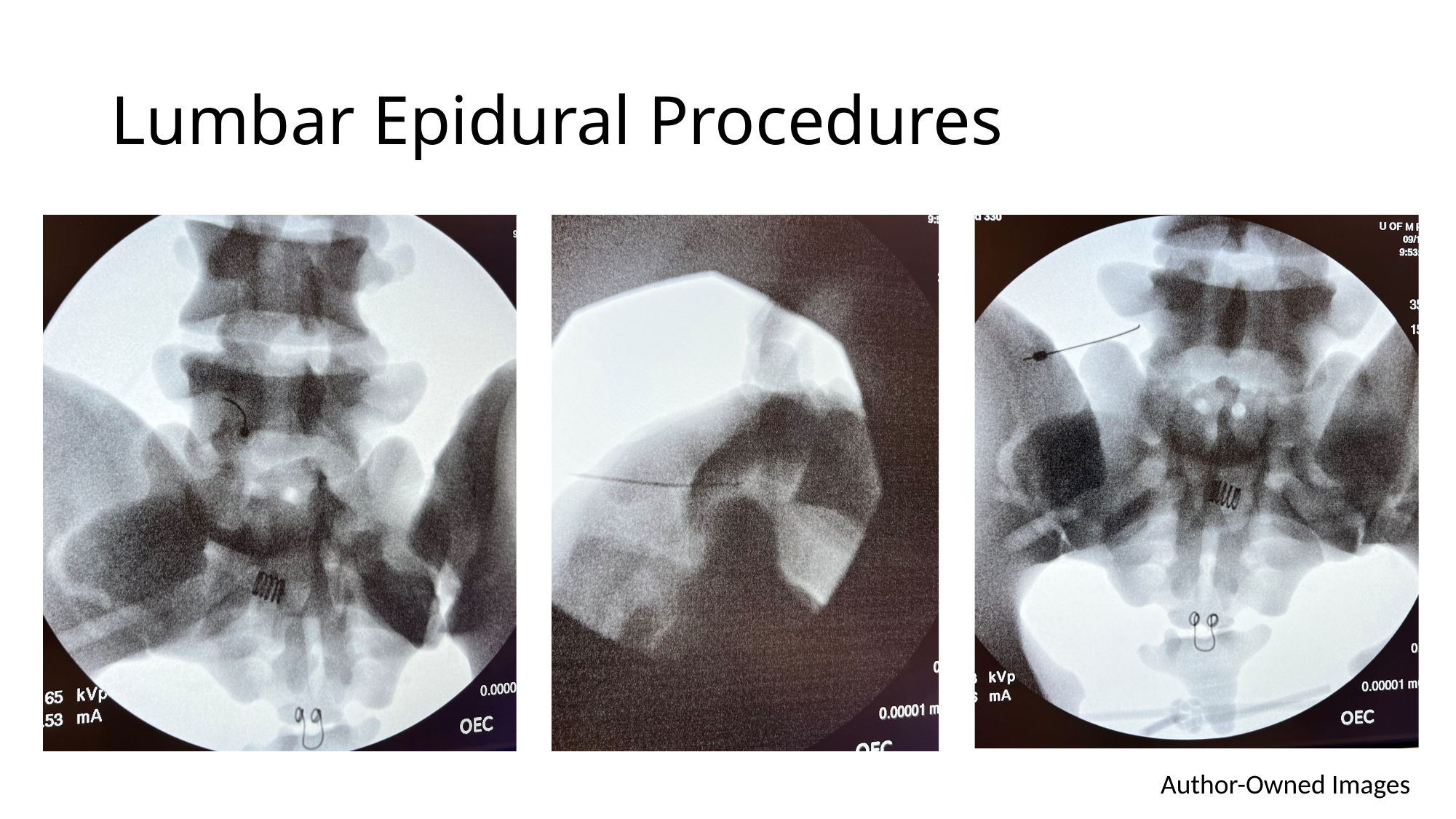

# Lumbar Epidural Procedures
Author-Owned Images
