# Supplementary material for: Resident Interventional Spine Course with Didactics and Hands-On Skills Lab
Source: MedEdPORTAL. 2025 Oct 7;21:11551. doi: 10.15766/mep_2374-8265.11551 (PMC12502988; doi:10.15766/mep_2374-8265.11551)
Supplement: Supplementary file 1 — Overview - Spine.pptxPrep Kit Materials.docxBuilding a Low-Cost Spine Simulator.pptxFacilitators Guide.docxSpine Procedure - Guidelines Lecture.pptxSpine Procedure Guidelines Lecture Video.mp4Course Chart Review Guidelines.docxSpine Course - Cases.pptxChart Review Preprocedures Checklist.docxInformed Consent and Procedure Timeout Checklist.docxLumbar Procedure Table Checklist.docxProcedure Descriptions.docxFluoroscopic Spine Procedure Images.pptxSpine Course Pre-Post Survey - Updated.docxSpine Course Pre-Post Survey - Original.docx [file mep_2374-8265.11551-s001.zip › D. Facilitators Guide.docx]

**Appendix D. Facilitator’s Guide for Course Instruction**

**Summary of Tips:**

Preparing for the course:

- The lead instructor’s role is to prepare for the course and guide the residents/instructors through two sessions lasting about three hours each in length. This can be adjusted to meet the needs of your program.
  - Lead instructor can provide lecture content; however, they will need support from the other instructors to conduct the various stations each session.
- The lead instructor can use Table 1 as a checklist to prepare for the course. Key action items include:
  - **Notifying Residents:** Send out email to the residents around one month prior to the course. This email should include deadlines to sign up, course agenda, pre-course materials, and any additional information (Appendix A, J, K, L, M).
  - **Recruiting Instructors:** Email spine/pain fellows and physicians to serve as station instructors and contribute to panel discussion. It is recommended to target a 1:3 instructor-to-resident ratio.
  - **Reserve Room and Prepare Materials:**
    - Reserve a lecture room with a projector or video screen to present lecture materials.
    - Reserve at least two rooms for rotating stations. This may be the pre-procedure area and an adjacent clinic room.
    - Reserve fluoroscopy suite that is equipped with C-arm, radiation safety equipment (lead aprons, glasses), and spine procedure kits (Appendix F).
  - **Coordinate Dinner/Refreshments:**
    - Consider reaching out to your department to sponsor food for this course and drop them off in advance; make sure to coordinate any food allergies.

Spine Procedure Simulation:

- The low-cost spine model can be assembled using the instructions in Appendix G.
- The simulated fluoroscopic spine procedure station should allow time for the following: instruction on basic needle driving technique, radiation safety, basic C-arm functions, and time for each resident to perform a self-selected spine procedure (5-10 min per procedure).
- Residents not actively performing procedures can operate the C-arm or prepare their procedure using Appendix L and M.
- As access to the spine simulator is a rate limiting factor for the course, the simulation instructor will determine when it is time for all residents to rotate stations.

Assessment:

- Residents should complete the pre-course survey when they first arrive to the course and the post-course survey before they leave (Appendix H). Attempts should be made to collect both pre- and post-course survey responses even if the resident is not able to attend all parts of the course.

**Additional Guidance:**

- Materials for this course are designed to be a framework to assist local interventional spine/pain physicians in customizing their own Resident Interventional Spine Course to meet the needs of their residents and comply with their local department policies.
- The lead instructor can turn the presentation in Appendix D into an on-demand pre-course video lecture to allow more time for in-person case-based discussion. A sample video is included in Appendix E; however, local instructors may want to edit this material and make their own video to meet the needs of their own program.
- Instructors should review course content and prepare their own stations.
  - Course references materials are listed in Appendix A.
- The lead instructor should arrive 20-30 minutes before each session to prepare the audiovisual equipment and set up the stations. All equipment should be inspected beforehand to ensure that everything is in working order.

**Session Organization timeline:**

- We recommend two sessions of approximately three hours each (Figure 1).
  - Sessions: 1-Part 1 and 2; Session 2-Part 1 (60 minutes for each session)
  - Stations: 3 rotating stations for 30 minutes each (90 minutes total)

**Preparing for Course Curriculum:**

- Pre-course material: Residents and instructors should be familiar with all course materials (Appendix D, I, J, K, L, and M).
- Course sessions:
  1. Familiarize yourself with the lectures and case materials.
  2. Budget time for discussion throughout and after the introduction of lecture and case review.
  3. Debrief students at the end of each session, as well as briefly at the end of each station.
  4. Allow time for feedback in person, as well as the post-course survey as a means to improve future courses.

**Facilitator’s Example Checklist:**

| **Checklist to prepare for the resident spine course** |
| --- |
| [ ] Recruit interventional spine instructors  [ ] Send invitation to residents with request for RSVP  [ ] Reserve a lecture room  [ ] Notify the fluoroscopic suite staff of the course schedule  [ ] Gather reference materials, handouts, and surveys  [ ] Build the spine simulator model (or) Ensure the spine simulator model is in good condition  [ ] Collect sterile procedure kits and needles from excess or damaged stock  [ ] Arrange for food/snacks |

**Additional Resources:**

1. Emergency Protocols. Accessed October 22, 2024. https://www.ipsismed.org/page/EmergencyProtocols

2. Patel VB, Wasserman R, Imani F. Interventional Therapies for Chronic Low Back Pain: A Focused Review (Efficacy and Outcomes). *Anesthesiol Pain Med*. 2015;5(4):e29716. doi:10.5812/aapm.29716

3. Narouze S, Benzon HT, Provenzano DA, et al. Interventional spine and pain procedures in patients on antiplatelet and anticoagulant medications: guidelines from the American Society of Regional Anesthesia and Pain Medicine, the European Society of Regional Anaesthesia and Pain Therapy, the American Academy of Pain Medicine, the International Neuromodulation Society, the North American Neuromodulation Society, and the World Institute of Pain. *Reg Anesth Pain Med*. 2015;40(3):182-212. doi:10.1097/AAP.0000000000000223

4. Narouze S, Benzon HT, Provenzano D, et al. Interventional Spine and Pain Procedures in Patients on Antiplatelet and Anticoagulant Medications (Second Edition): Guidelines From the American Society of Regional Anesthesia and Pain Medicine, the European Society of Regional Anaesthesia and Pain Therapy, the American Academy of Pain Medicine, the International Neuromodulation Society, the North American Neuromodulation Society, and the World Institute of Pain. *Reg Anesth Pain Med*. 2018;43(3):225-262. doi:10.1097/AAP.0000000000000700
