# Supplementary material for: Resident Interventional Spine Course with Didactics and Hands-On Skills Lab
Source: MedEdPORTAL. 2025 Oct 7;21:11551. doi: 10.15766/mep_2374-8265.11551 (PMC12502988; doi:10.15766/mep_2374-8265.11551)
Supplement: Supplementary file 1 — Overview - Spine.pptxPrep Kit Materials.docxBuilding a Low-Cost Spine Simulator.pptxFacilitators Guide.docxSpine Procedure - Guidelines Lecture.pptxSpine Procedure Guidelines Lecture Video.mp4Course Chart Review Guidelines.docxSpine Course - Cases.pptxChart Review Preprocedures Checklist.docxInformed Consent and Procedure Timeout Checklist.docxLumbar Procedure Table Checklist.docxProcedure Descriptions.docxFluoroscopic Spine Procedure Images.pptxSpine Course Pre-Post Survey - Updated.docxSpine Course Pre-Post Survey - Original.docx [file mep_2374-8265.11551-s001.zip › E. Spine Procedure - Guidelines Lecture.pptx]

## Slide 1
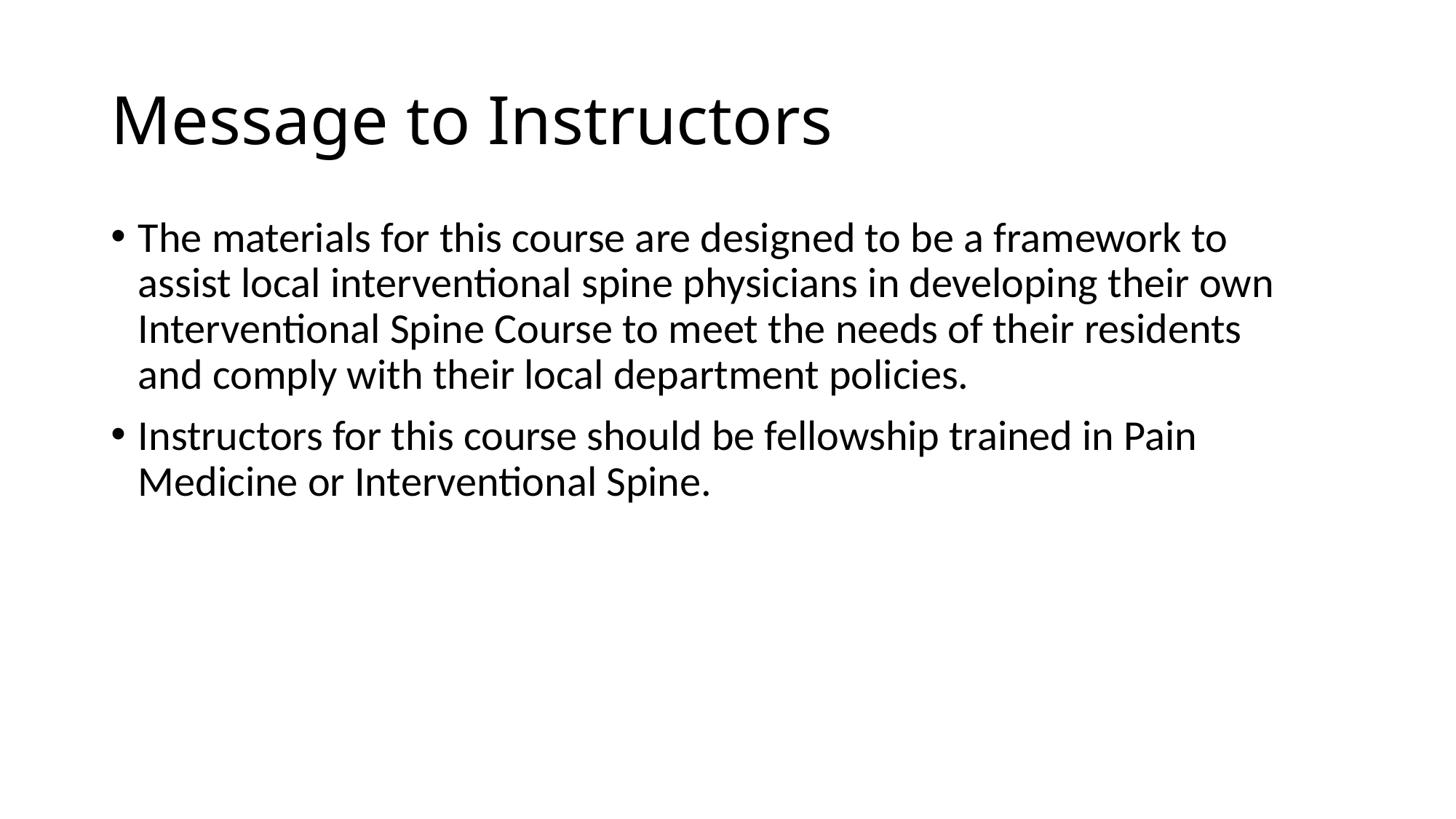

# Message to Instructors
The materials for this course are designed to be a framework to assist local interventional spine physicians in developing their own Interventional Spine Course to meet the needs of their residents and comply with their local department policies.
Instructors for this course should be fellowship trained in Pain Medicine or Interventional Spine.

## Slide 2
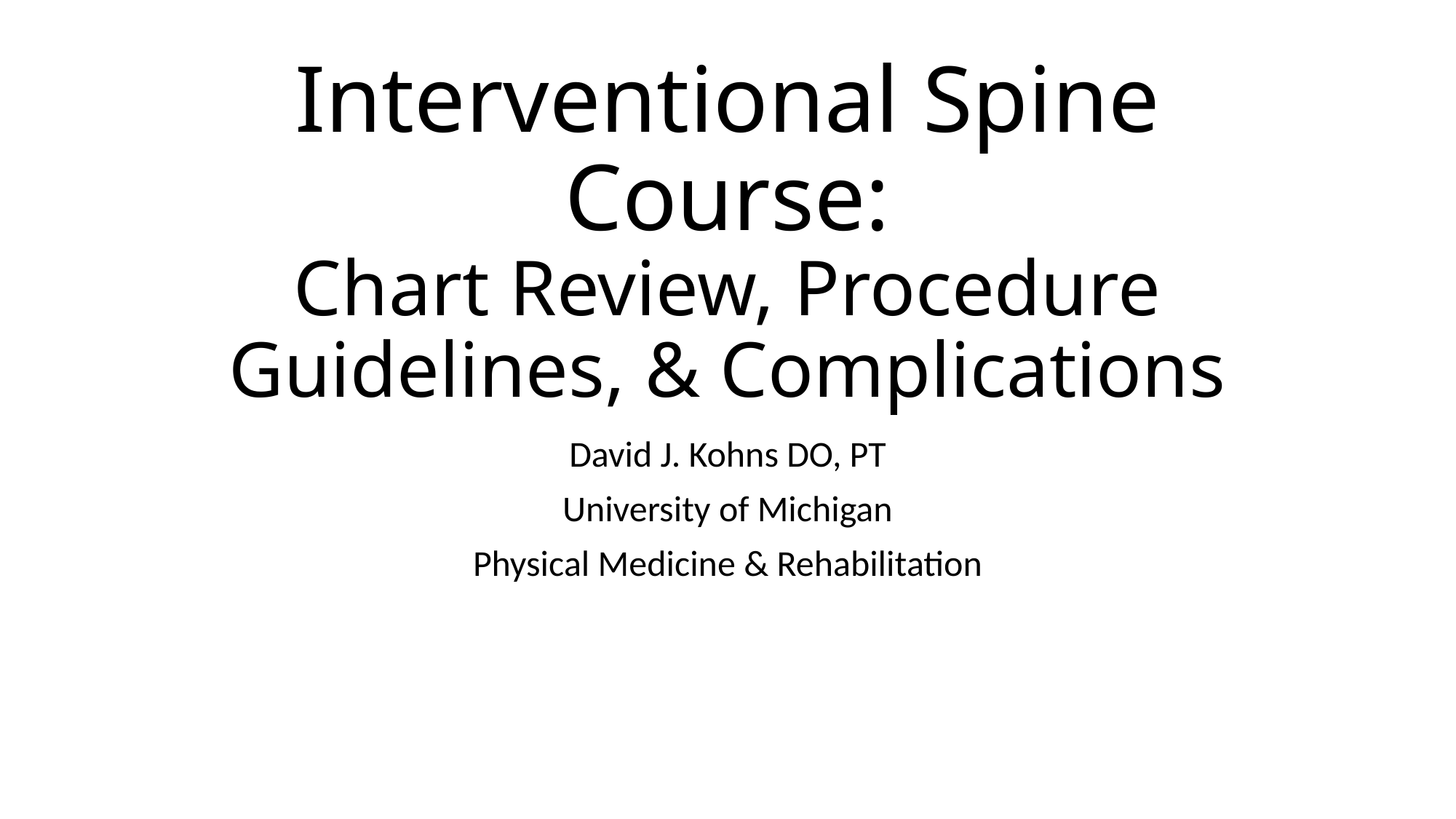

# Interventional Spine Course: Chart Review, Procedure Guidelines, & Complications
David J. Kohns DO, PT
University of Michigan
Physical Medicine & Rehabilitation

## Slide 3
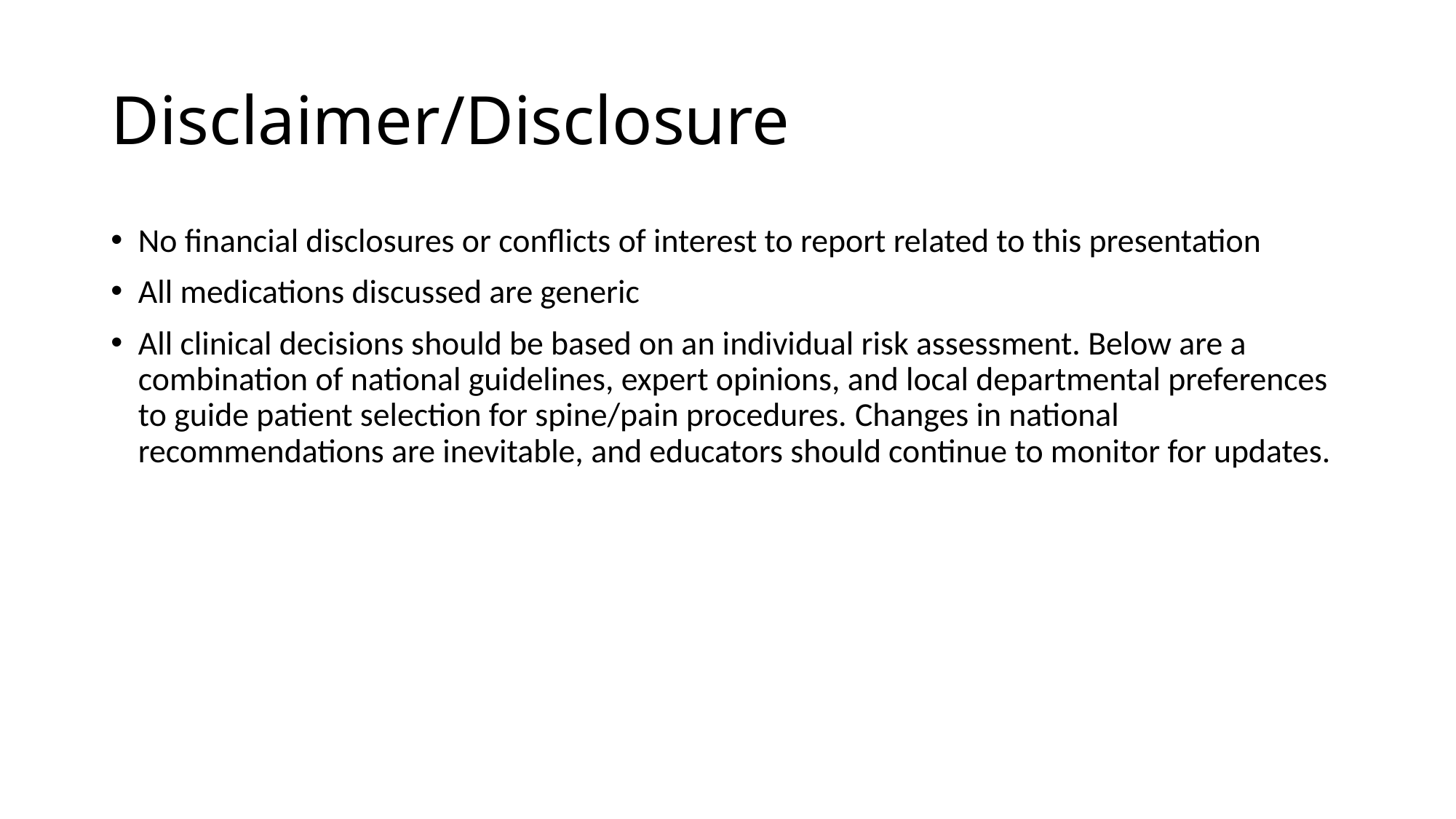

# Disclaimer/Disclosure
No financial disclosures or conflicts of interest to report related to this presentation
All medications discussed are generic
All clinical decisions should be based on an individual risk assessment. Below are a combination of national guidelines, expert opinions, and local departmental preferences to guide patient selection for spine/pain procedures. Changes in national recommendations are inevitable, and educators should continue to monitor for updates.

## Slide 4
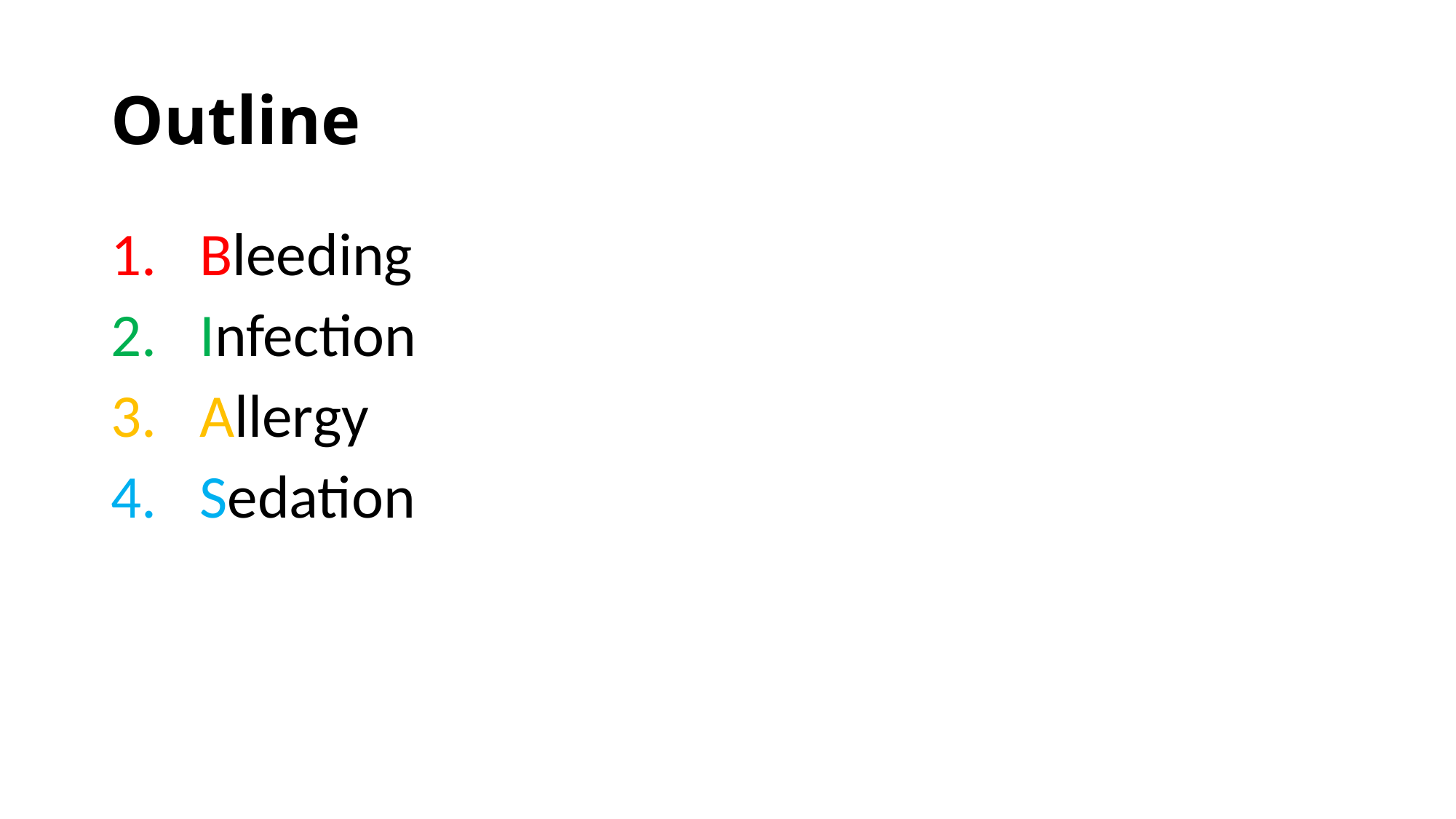

# Outline
Bleeding
Infection
Allergy
Sedation

## Slide 5
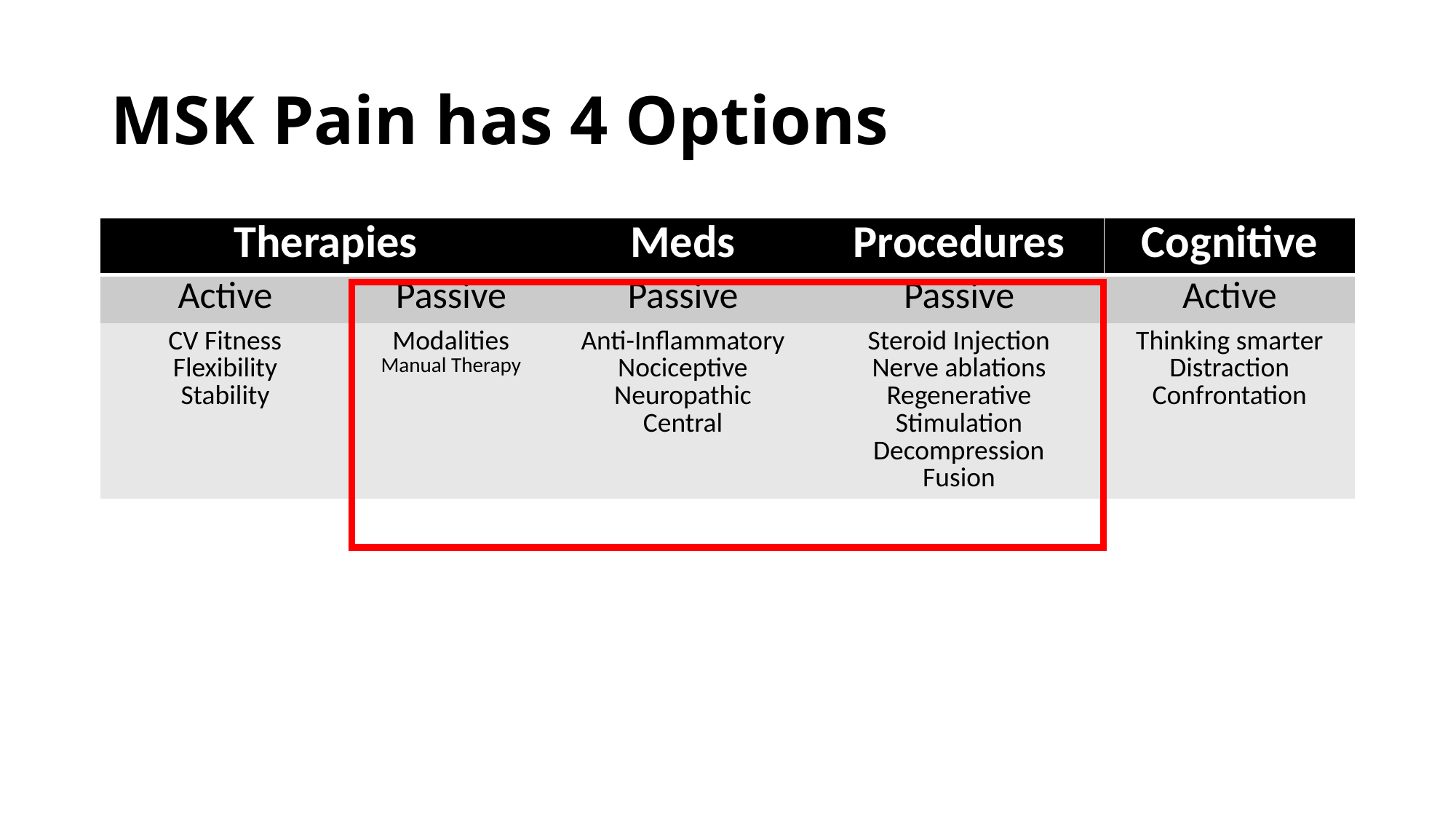

# MSK Pain has 4 Options
| Therapies | | Meds | Procedures | Cognitive |
| --- | --- | --- | --- | --- |
| Active | Passive | Passive | Passive | Active |
| CV Fitness Flexibility Stability | Modalities Manual Therapy | Anti-Inflammatory Nociceptive Neuropathic Central | Steroid Injection Nerve ablations Regenerative Stimulation Decompression Fusion | Thinking smarter Distraction Confrontation |

## Slide 6
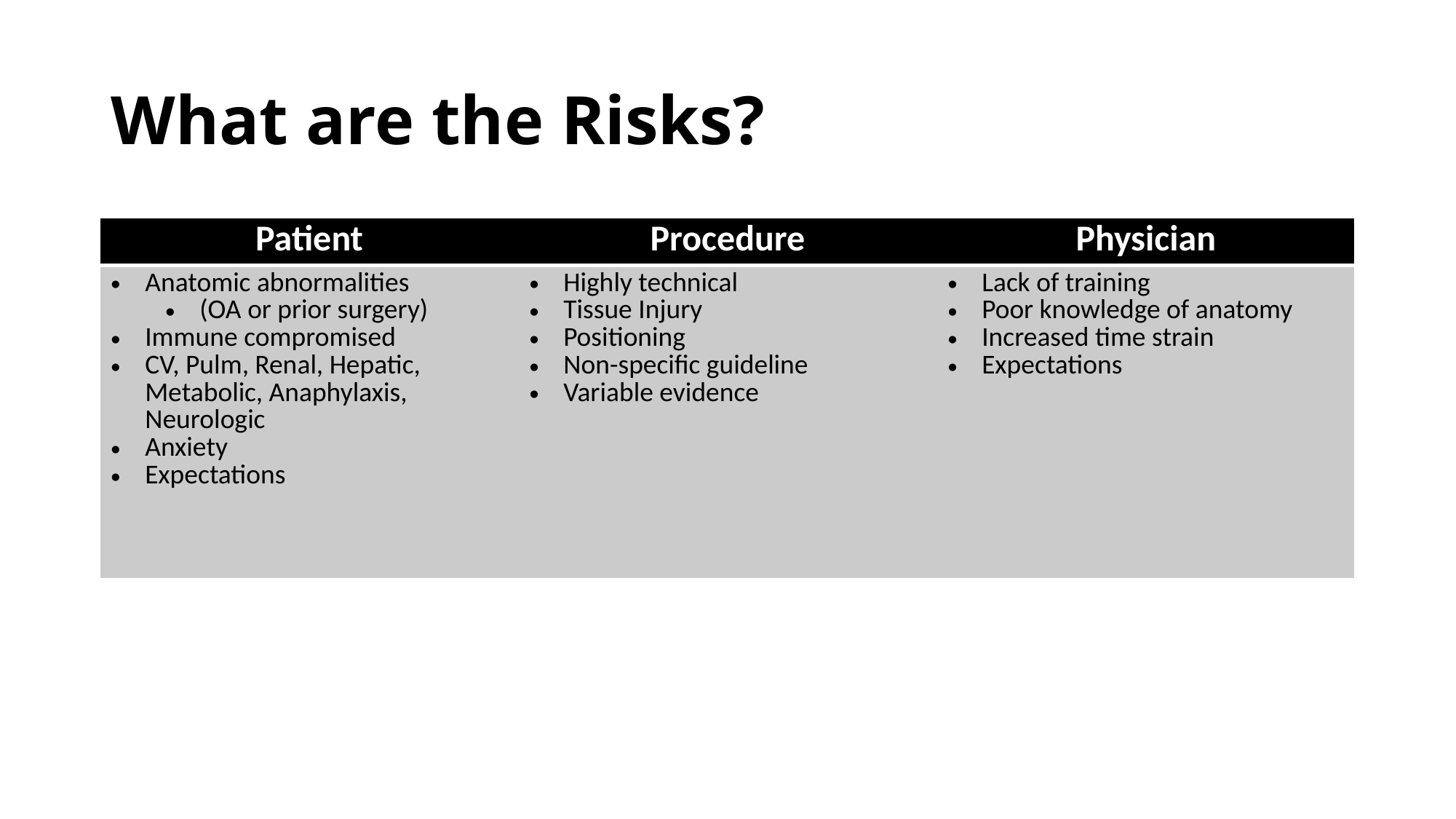

# What are the Risks?
| Patient | Procedure | Physician |
| --- | --- | --- |
| Anatomic abnormalities (OA or prior surgery) Immune compromised CV, Pulm, Renal, Hepatic, Metabolic, Anaphylaxis, Neurologic Anxiety Expectations | Highly technical Tissue Injury Positioning Non-specific guideline Variable evidence | Lack of training Poor knowledge of anatomy Increased time strain Expectations |

## Slide 7
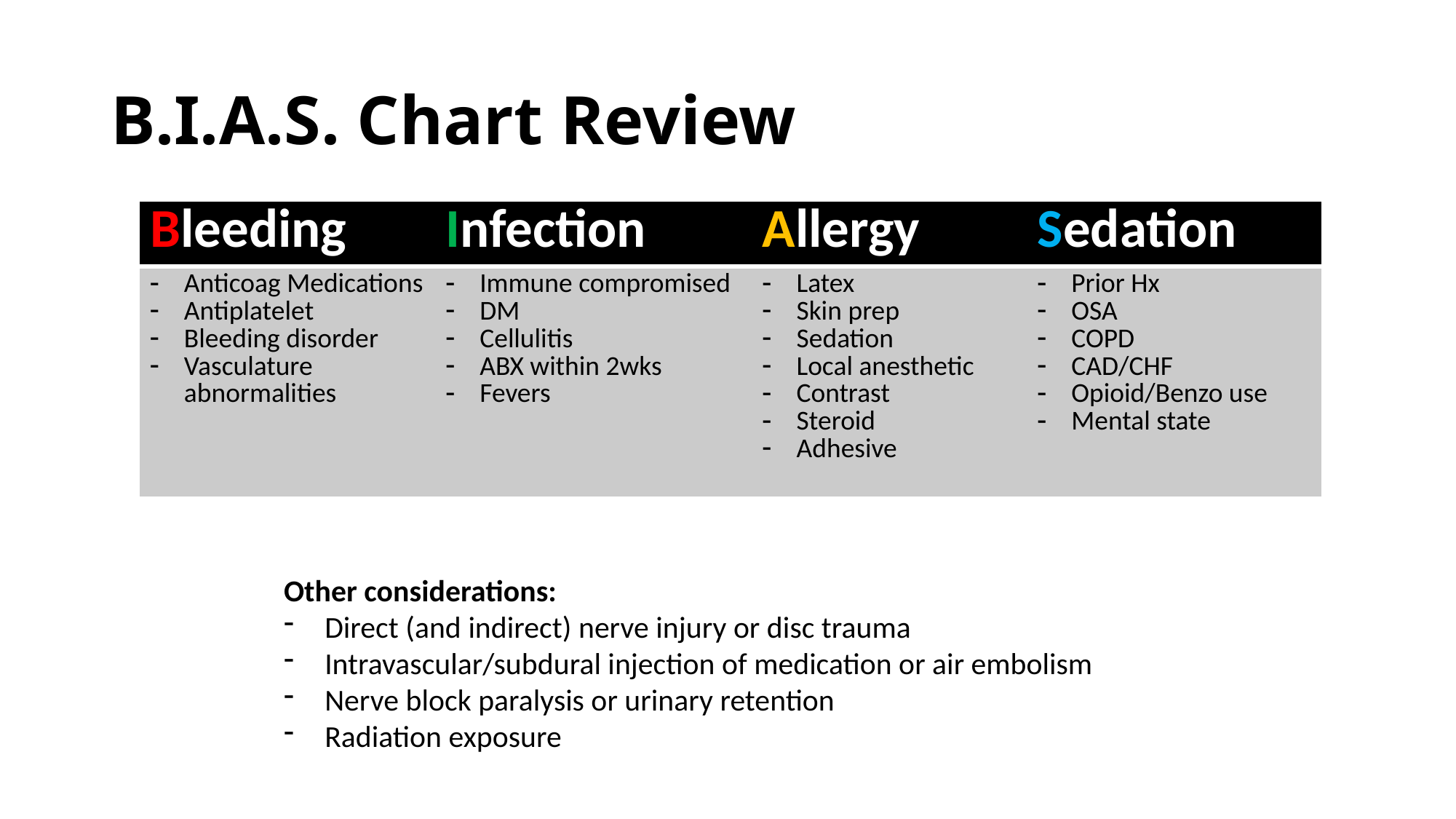

# B.I.A.S. Chart Review
| Bleeding | Infection | Allergy | Sedation |
| --- | --- | --- | --- |
| Anticoag Medications Antiplatelet Bleeding disorder Vasculature abnormalities | Immune compromised DM Cellulitis ABX within 2wks Fevers | Latex Skin prep Sedation Local anesthetic Contrast Steroid Adhesive | Prior Hx OSA COPD CAD/CHF Opioid/Benzo use Mental state |
Other considerations:
Direct (and indirect) nerve injury or disc trauma
Intravascular/subdural injection of medication or air embolism
Nerve block paralysis or urinary retention
Radiation exposure

## Slide 8
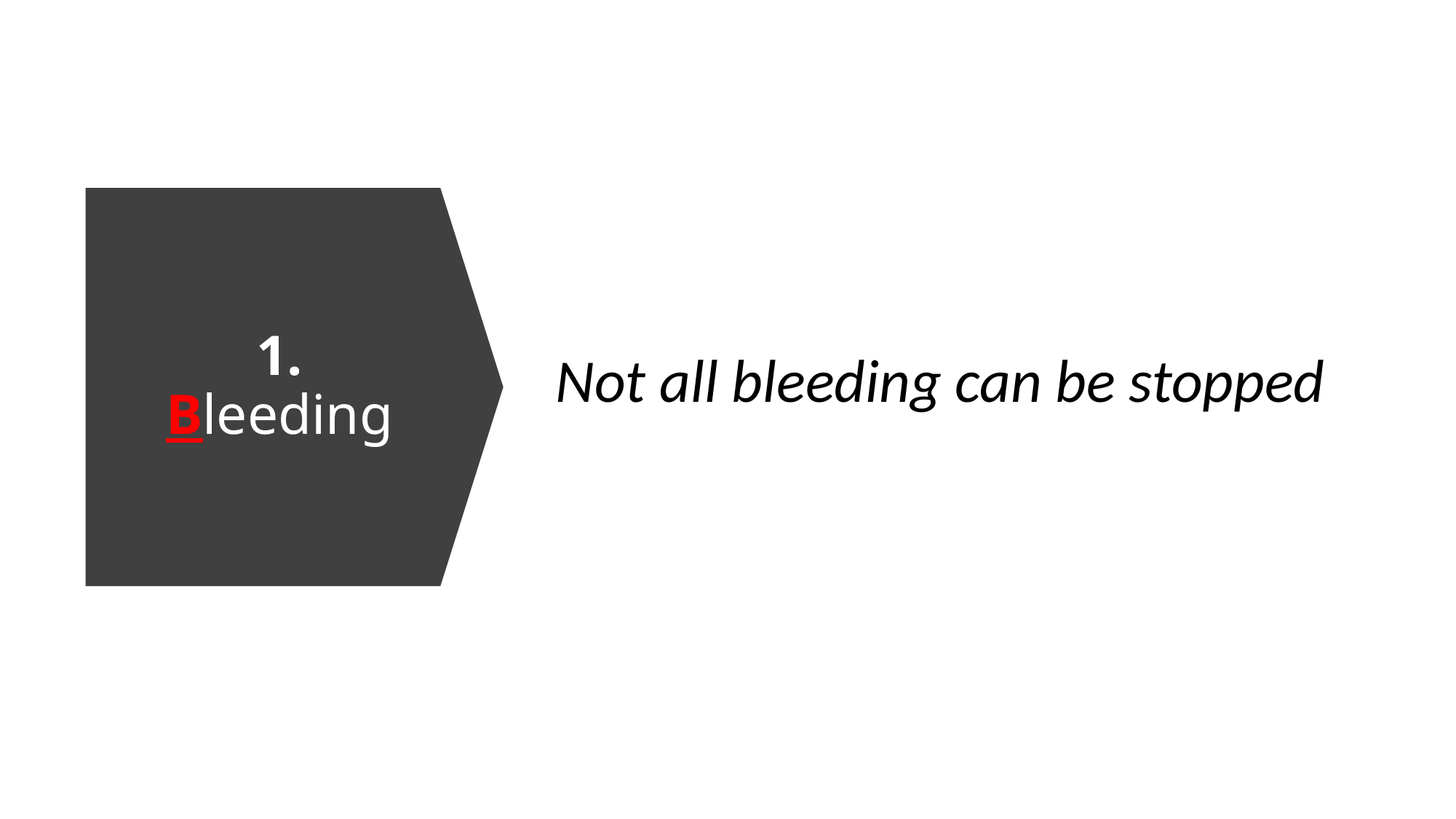

# 1. Bleeding
Not all bleeding can be stopped

## Slide 9
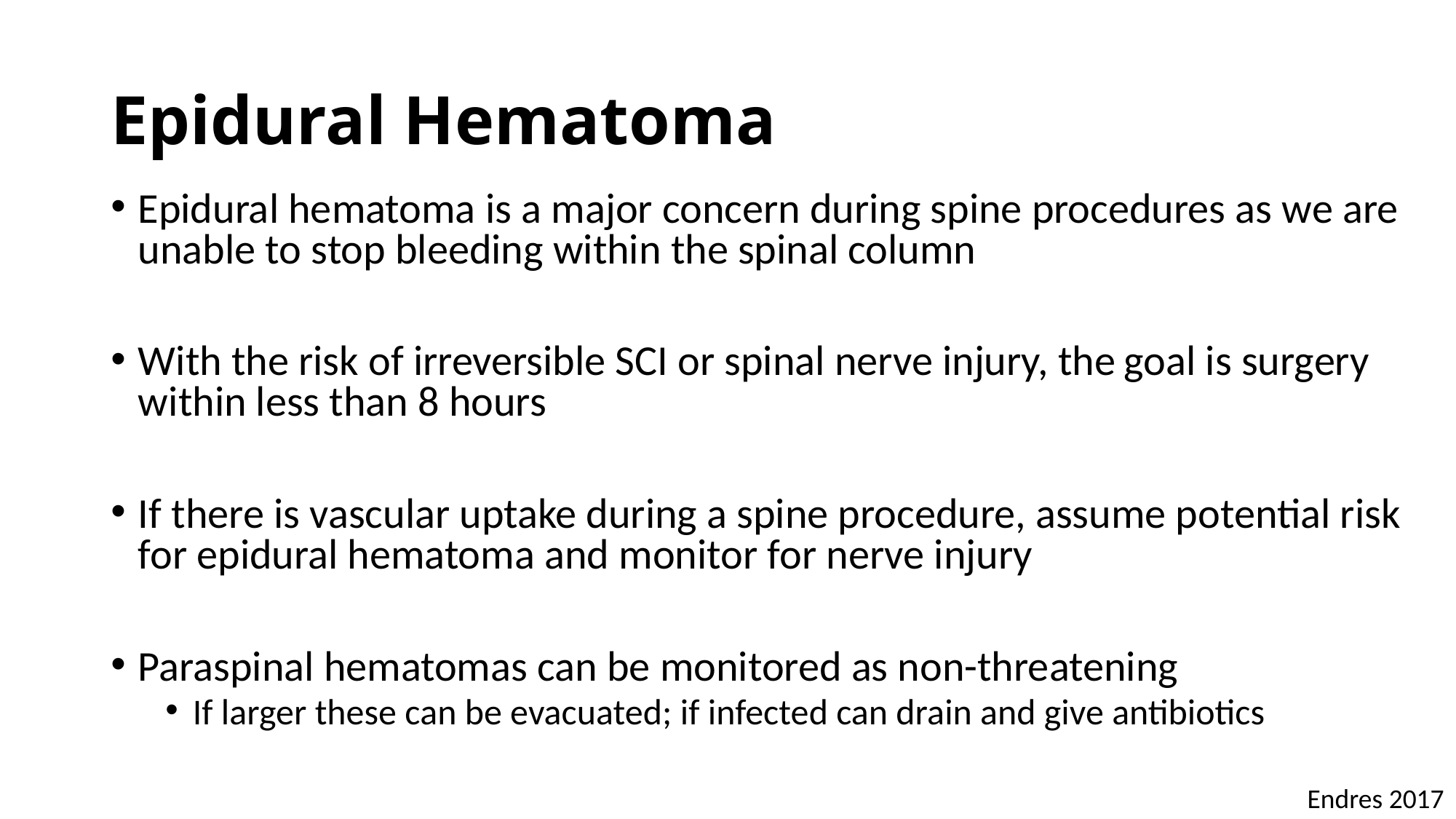

# Epidural Hematoma
Epidural hematoma is a major concern during spine procedures as we are unable to stop bleeding within the spinal column
With the risk of irreversible SCI or spinal nerve injury, the goal is surgery within less than 8 hours
If there is vascular uptake during a spine procedure, assume potential risk for epidural hematoma and monitor for nerve injury
Paraspinal hematomas can be monitored as non-threatening
If larger these can be evacuated; if infected can drain and give antibiotics
Endres 2017

## Slide 10
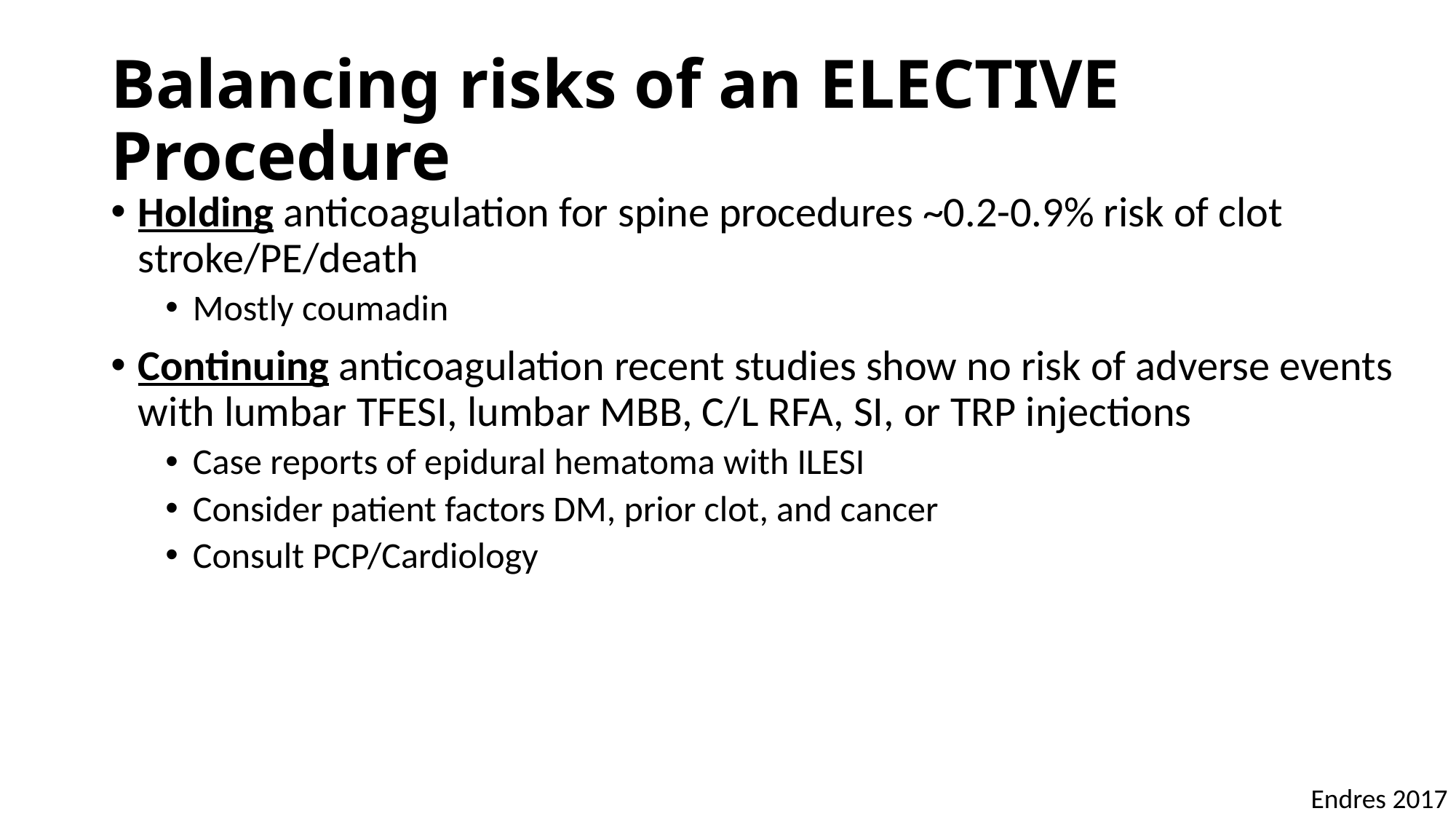

# Balancing risks of an ELECTIVE Procedure
Holding anticoagulation for spine procedures ~0.2-0.9% risk of clot stroke/PE/death
Mostly coumadin
Continuing anticoagulation recent studies show no risk of adverse events with lumbar TFESI, lumbar MBB, C/L RFA, SI, or TRP injections
Case reports of epidural hematoma with ILESI
Consider patient factors DM, prior clot, and cancer
Consult PCP/Cardiology
Endres 2017

## Slide 11
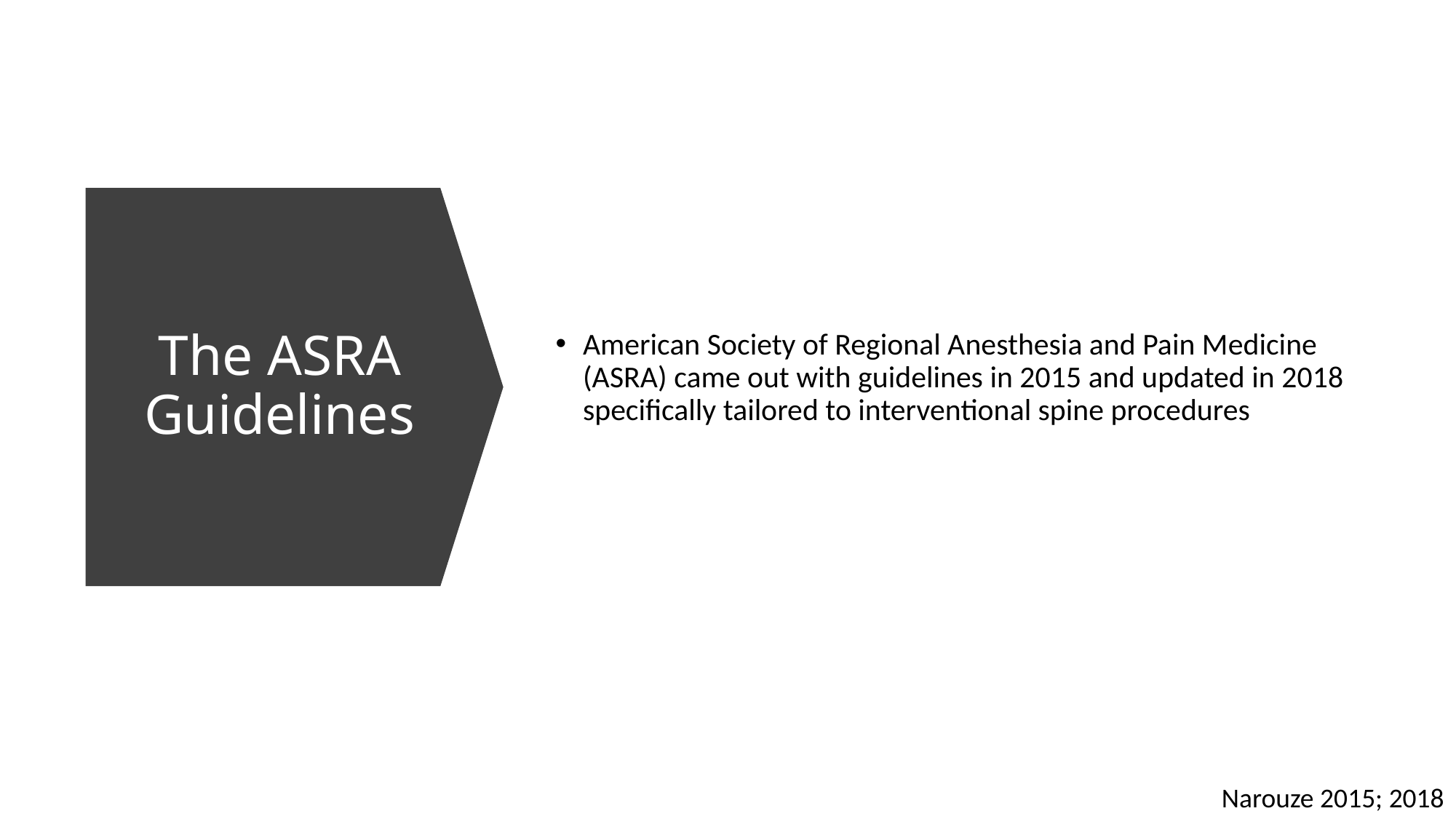

# The ASRA Guidelines
American Society of Regional Anesthesia and Pain Medicine (ASRA) came out with guidelines in 2015 and updated in 2018 specifically tailored to interventional spine procedures
Narouze 2015; 2018

## Slide 12
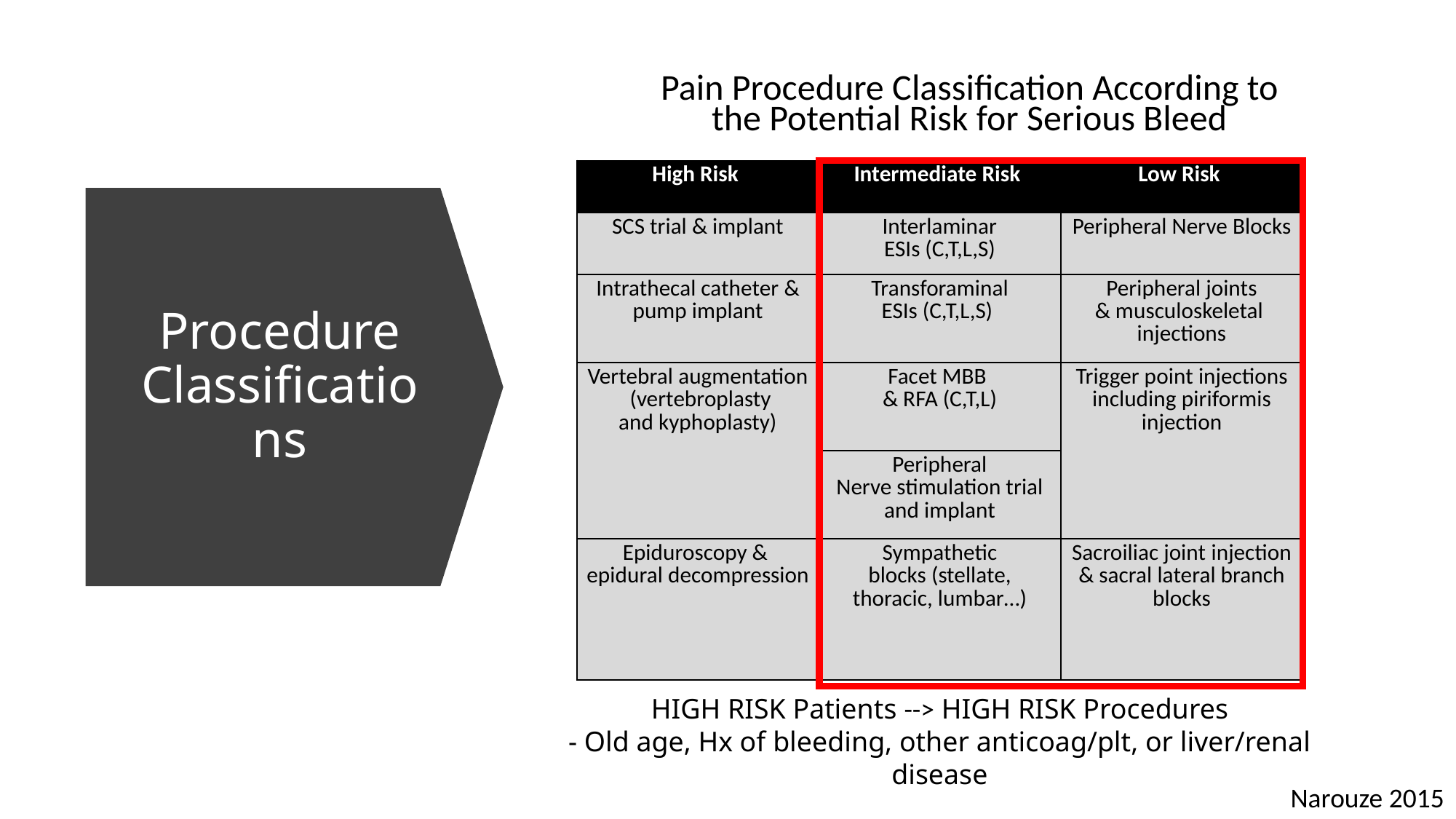

Pain Procedure Classification According to the Potential Risk for Serious Bleed
| High Risk | Intermediate Risk | Low Risk |
| --- | --- | --- |
| SCS trial & implant | Interlaminar ESIs (C,T,L,S) | Peripheral Nerve Blocks |
| Intrathecal catheter & pump implant | Transforaminal ESIs (C,T,L,S) | Peripheral joints & musculoskeletal  injections |
| Vertebral augmentation (vertebroplasty and kyphoplasty) | Facet MBB & RFA (C,T,L) | Trigger point injections including piriformis injection |
| | Peripheral Nerve stimulation trial and implant | |
| Epiduroscopy &  epidural decompression | Sympathetic blocks (stellate, thoracic, lumbar…) | Sacroiliac joint injection & sacral lateral branch blocks |
# Procedure Classifications
HIGH RISK Patients --> HIGH RISK Procedures
- Old age, Hx of bleeding, other anticoag/plt, or liver/renal disease
Narouze 2015

## Slide 13
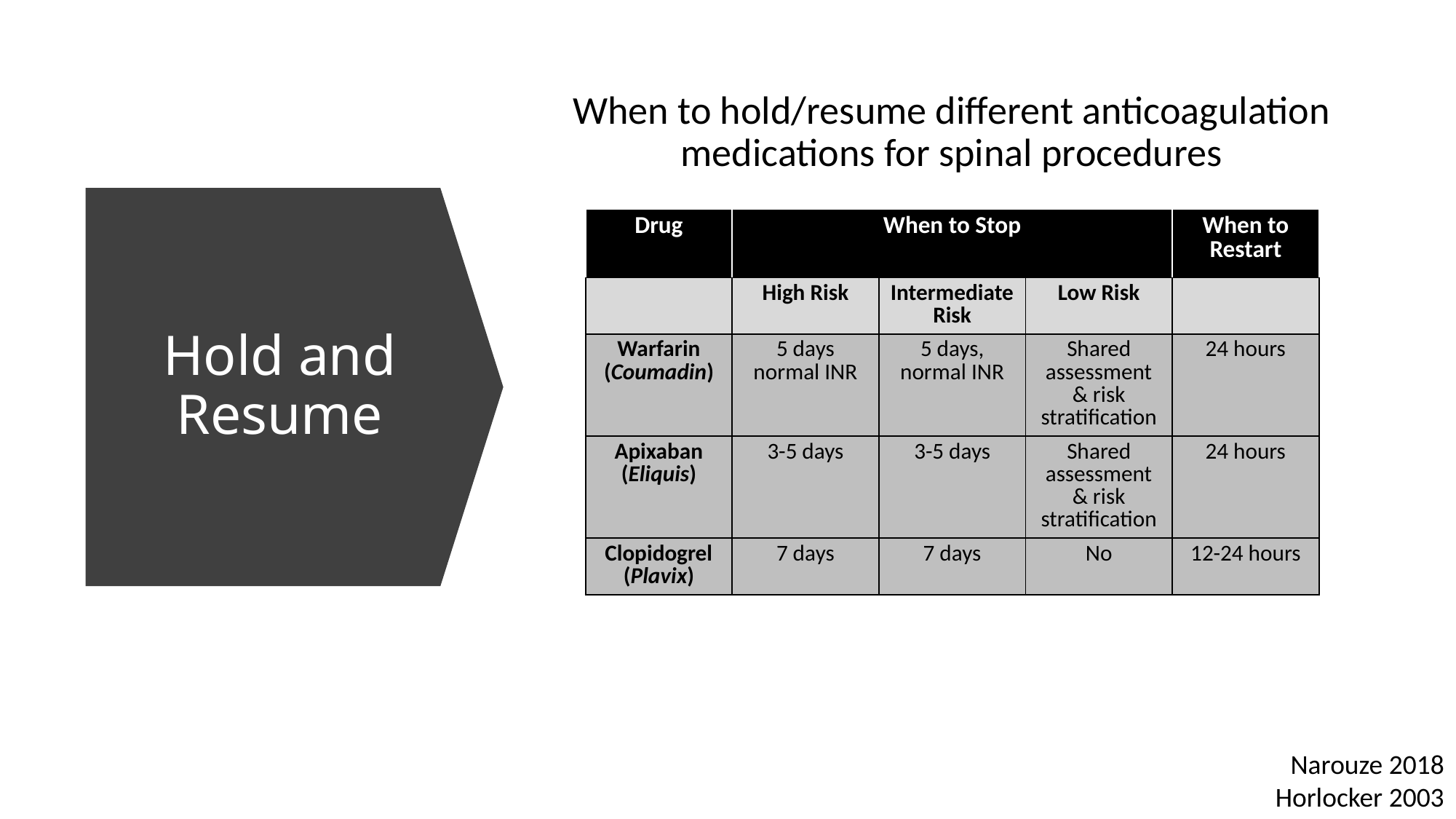

When to hold/resume different anticoagulation medications for spinal procedures
| Drug | When to Stop | | | When to Restart |
| --- | --- | --- | --- | --- |
| | High Risk | Intermediate Risk | Low Risk | |
| Warfarin (Coumadin) | 5 days normal INR | 5 days, normal INR | Shared assessment & risk stratification | 24 hours |
| Apixaban (Eliquis) | 3-5 days | 3-5 days | Shared assessment & risk stratification | 24 hours |
| Clopidogrel (Plavix) | 7 days | 7 days | No | 12-24 hours |
# Hold and Resume
Narouze 2018
Horlocker 2003

## Slide 14
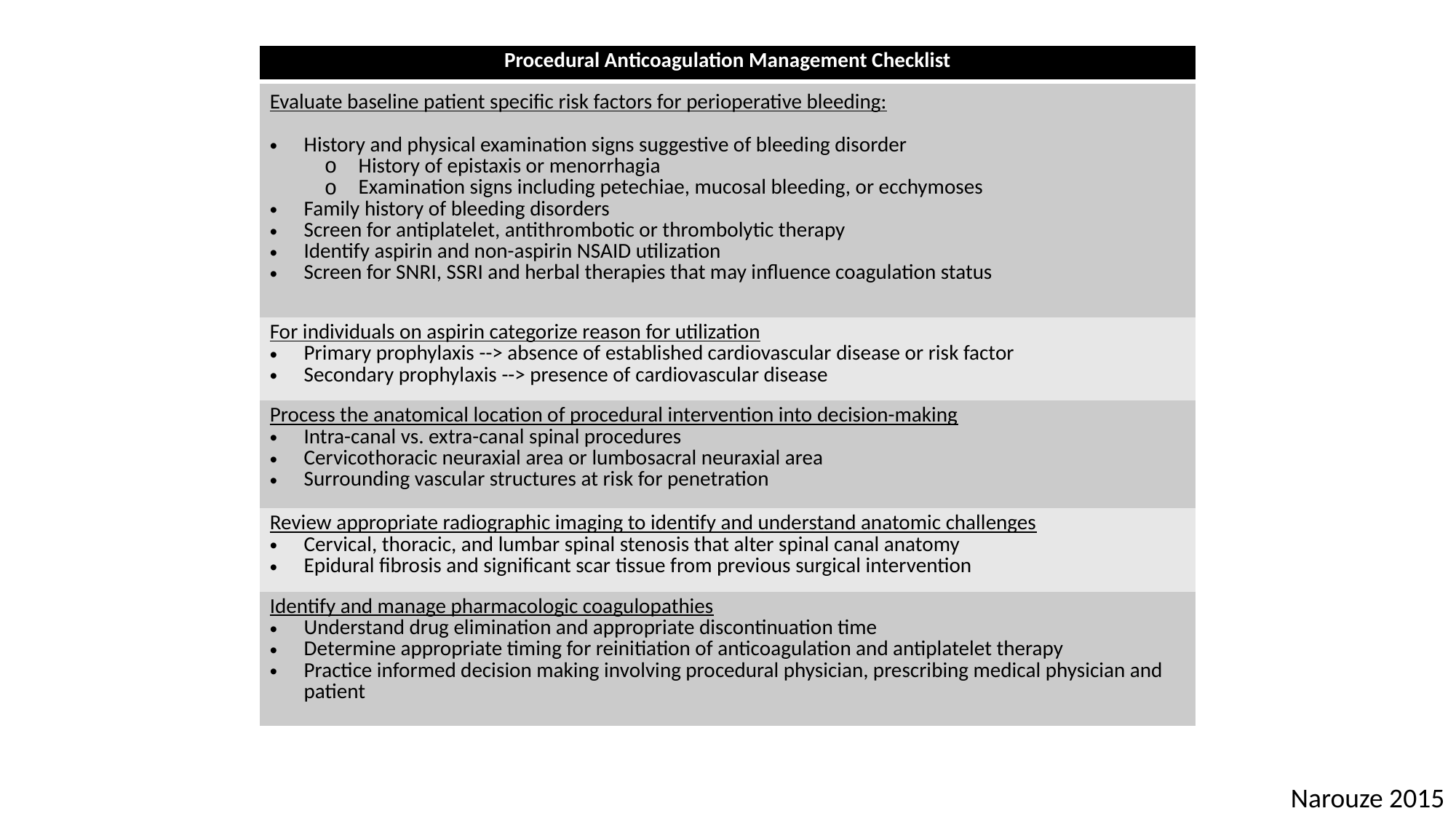

| Procedural Anticoagulation Management Checklist |
| --- |
| Evaluate baseline patient specific risk factors for perioperative bleeding: History and physical examination signs suggestive of bleeding disorder History of epistaxis or menorrhagia Examination signs including petechiae, mucosal bleeding, or ecchymoses Family history of bleeding disorders Screen for antiplatelet, antithrombotic or thrombolytic therapy Identify aspirin and non-aspirin NSAID utilization Screen for SNRI, SSRI and herbal therapies that may influence coagulation status |
| For individuals on aspirin categorize reason for utilization Primary prophylaxis --> absence of established cardiovascular disease or risk factor Secondary prophylaxis --> presence of cardiovascular disease |
| Process the anatomical location of procedural intervention into decision-making Intra-canal vs. extra-canal spinal procedures Cervicothoracic neuraxial area or lumbosacral neuraxial area Surrounding vascular structures at risk for penetration |
| Review appropriate radiographic imaging to identify and understand anatomic challenges Cervical, thoracic, and lumbar spinal stenosis that alter spinal canal anatomy Epidural fibrosis and significant scar tissue from previous surgical intervention |
| Identify and manage pharmacologic coagulopathies Understand drug elimination and appropriate discontinuation time Determine appropriate timing for reinitiation of anticoagulation and antiplatelet therapy Practice informed decision making involving procedural physician, prescribing medical physician and patient |
Narouze 2015

## Slide 15
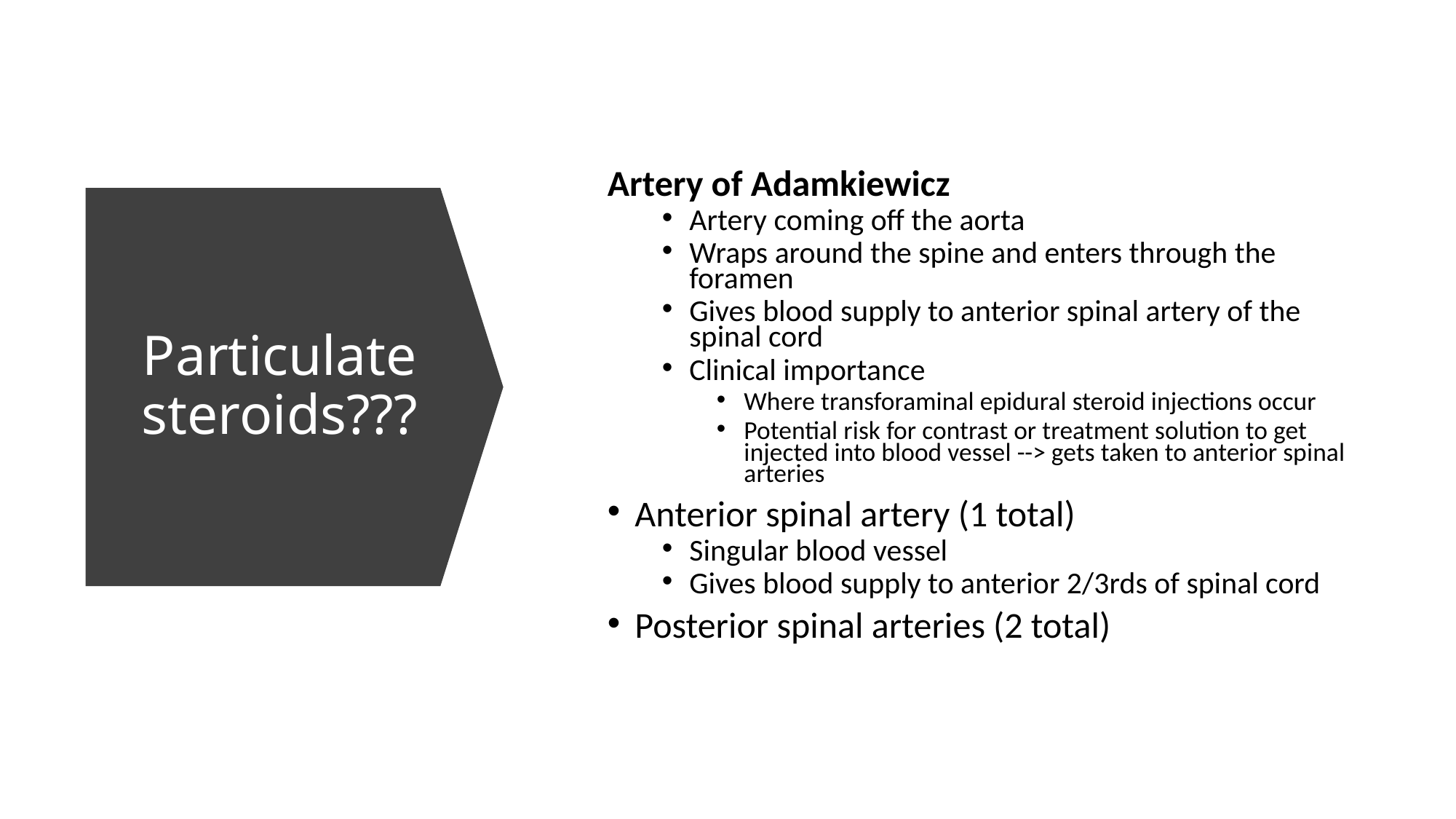

Artery of Adamkiewicz
Artery coming off the aorta
Wraps around the spine and enters through the foramen
Gives blood supply to anterior spinal artery of the spinal cord
Clinical importance
Where transforaminal epidural steroid injections occur
Potential risk for contrast or treatment solution to get injected into blood vessel --> gets taken to anterior spinal arteries
Anterior spinal artery (1 total)
Singular blood vessel
Gives blood supply to anterior 2/3rds of spinal cord
Posterior spinal arteries (2 total)
# Particulate steroids???

## Slide 16
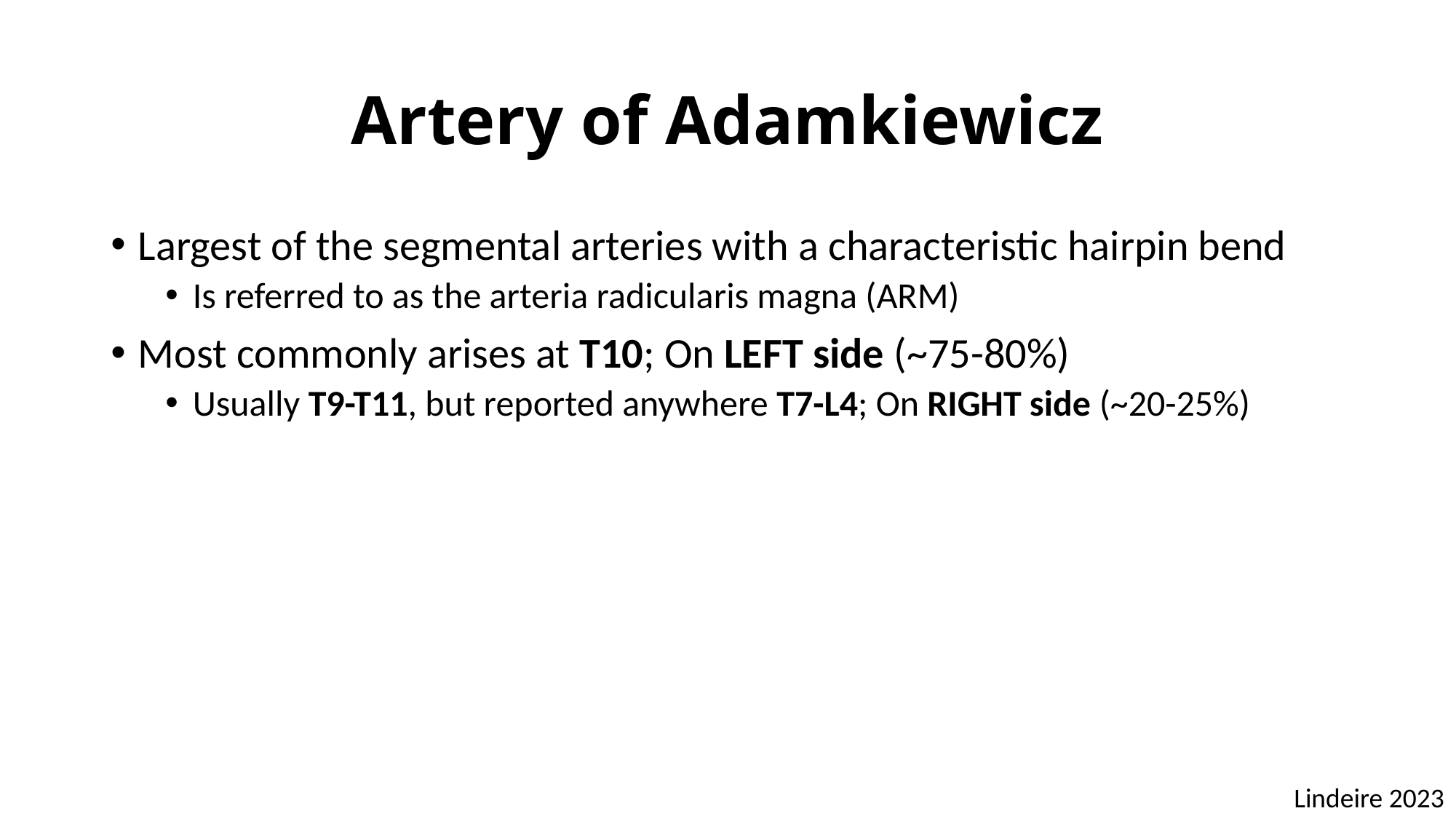

# Artery of Adamkiewicz
Largest of the segmental arteries with a characteristic hairpin bend
Is referred to as the arteria radicularis magna (ARM)
Most commonly arises at T10; On LEFT side (~75-80%)
Usually T9-T11, but reported anywhere T7-L4; On RIGHT side (~20-25%)
 Lindeire 2023

## Slide 17
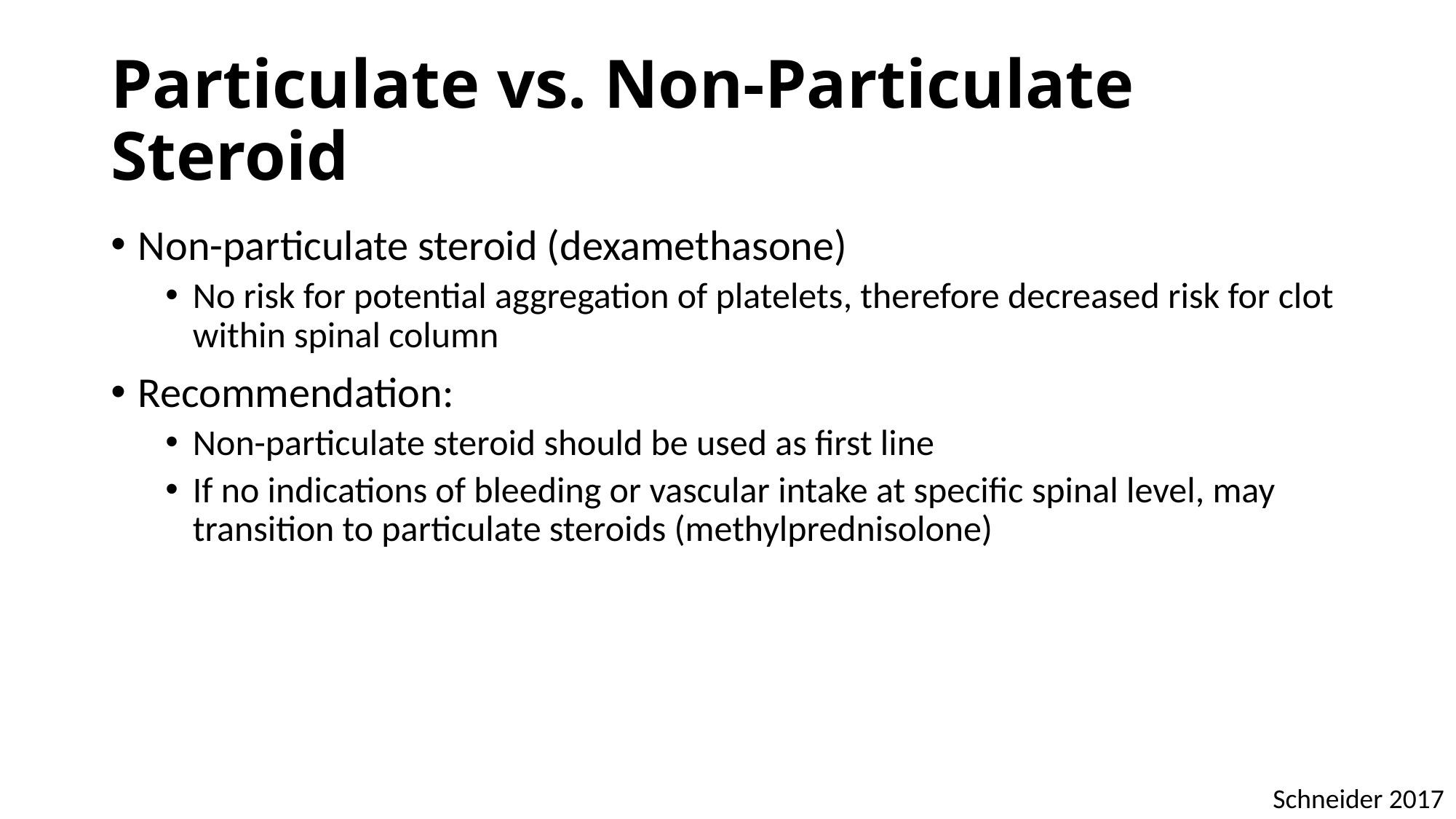

# Particulate vs. Non-Particulate Steroid
Non-particulate steroid (dexamethasone)
No risk for potential aggregation of platelets, therefore decreased risk for clot within spinal column
Recommendation:
Non-particulate steroid should be used as first line
If no indications of bleeding or vascular intake at specific spinal level, may transition to particulate steroids (methylprednisolone)
 Schneider 2017

## Slide 18
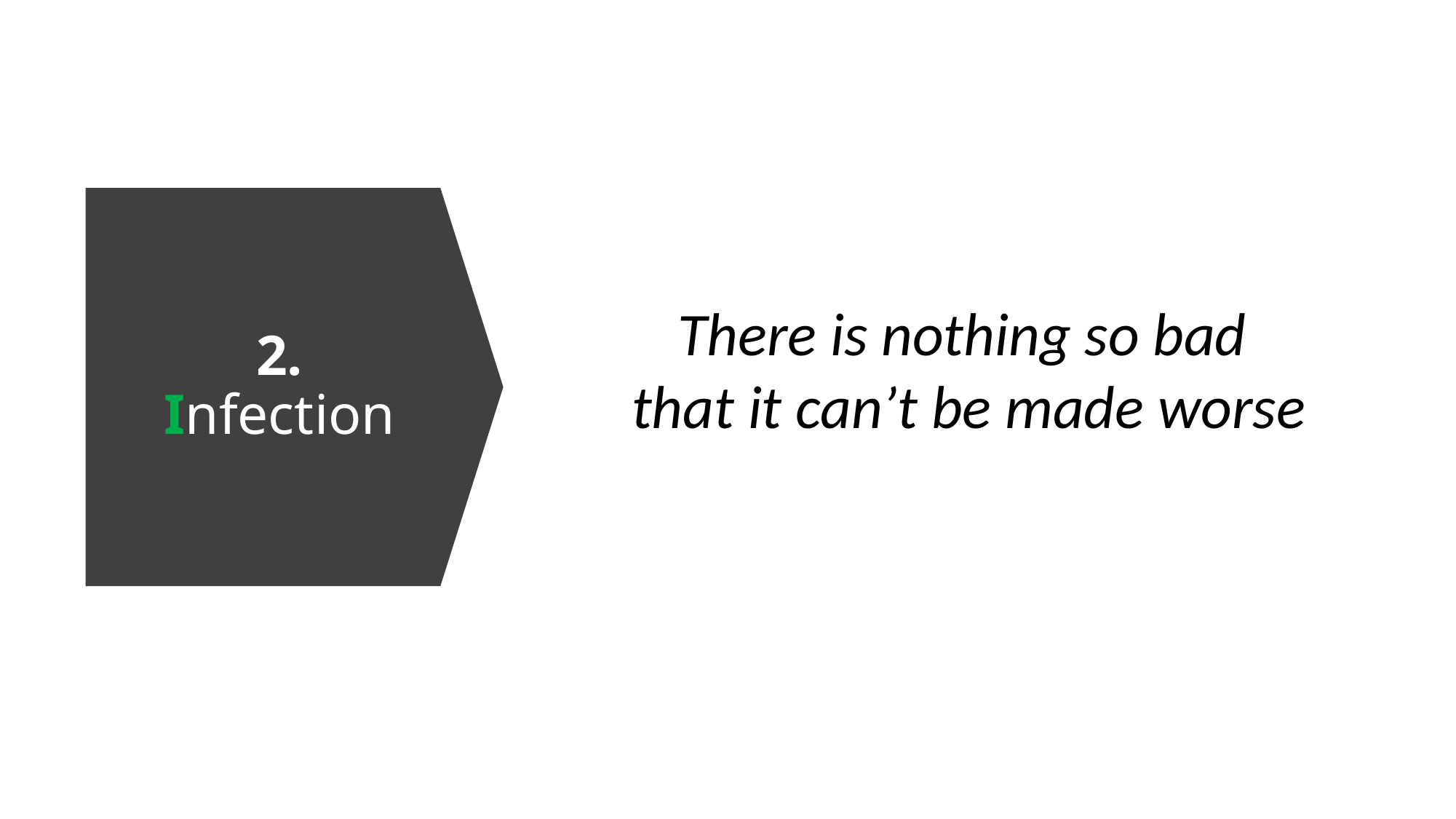

# 2. Infection
There is nothing so bad
that it can’t be made worse

## Slide 19
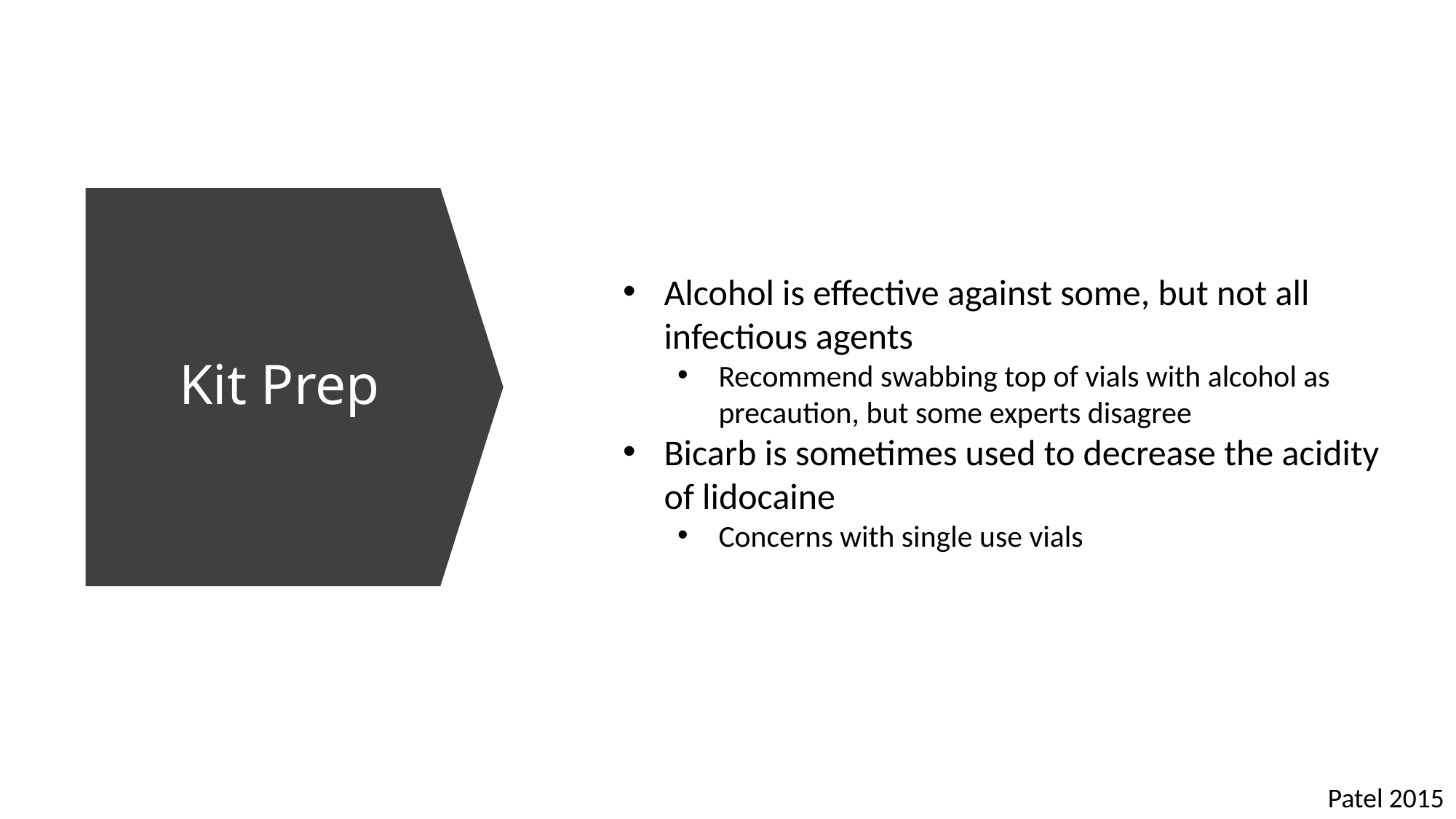

Alcohol is effective against some, but not all infectious agents
Recommend swabbing top of vials with alcohol as precaution, but some experts disagree
Bicarb is sometimes used to decrease the acidity of lidocaine
Concerns with single use vials
# Kit Prep
 Patel 2015

## Slide 20
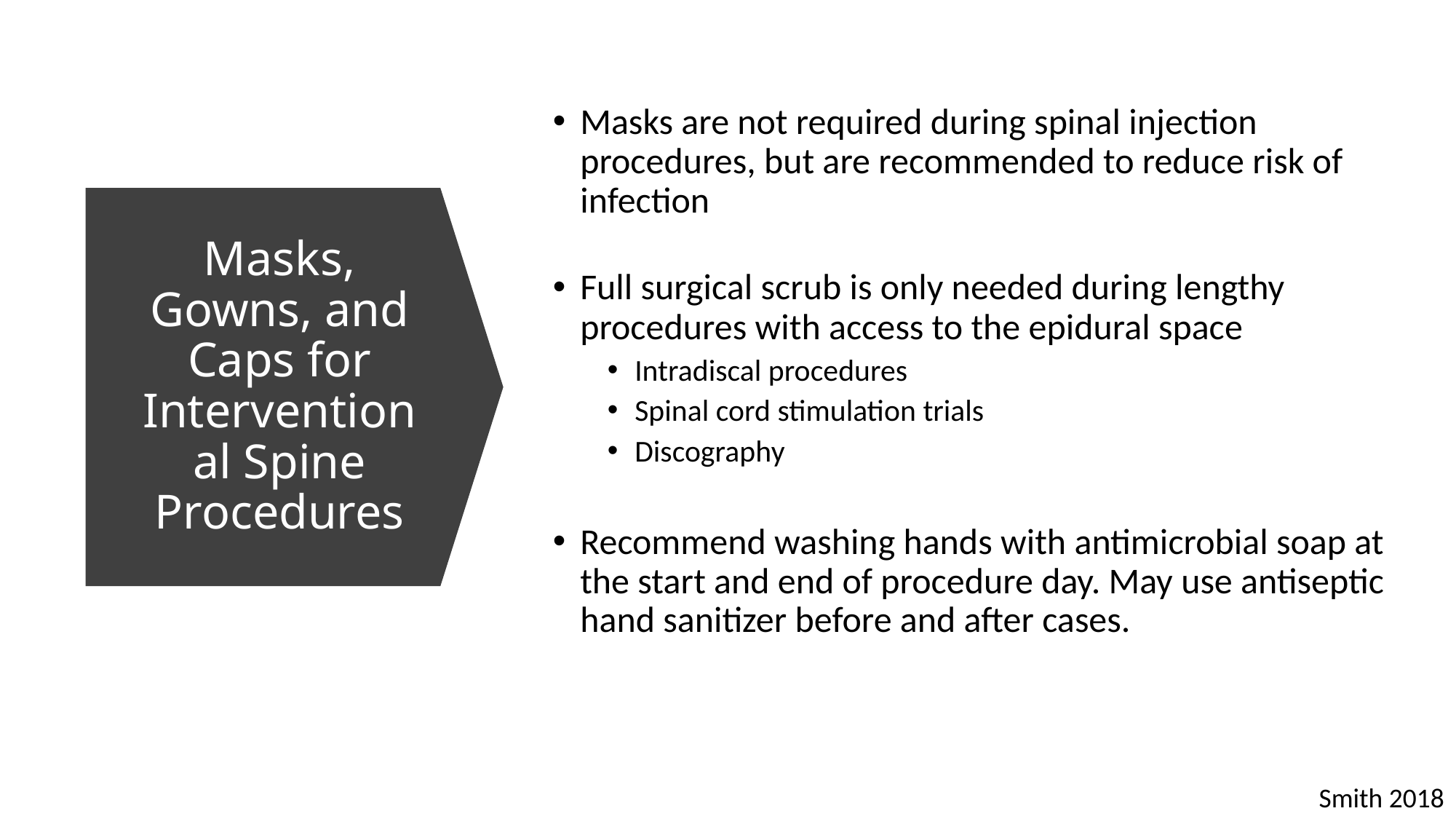

Masks are not required during spinal injection procedures, but are recommended to reduce risk of infection
Full surgical scrub is only needed during lengthy procedures with access to the epidural space
Intradiscal procedures
Spinal cord stimulation trials
Discography
Recommend washing hands with antimicrobial soap at the start and end of procedure day. May use antiseptic hand sanitizer before and after cases.
# Masks, Gowns, and Caps for Interventional Spine Procedures
Smith 2018

## Slide 21
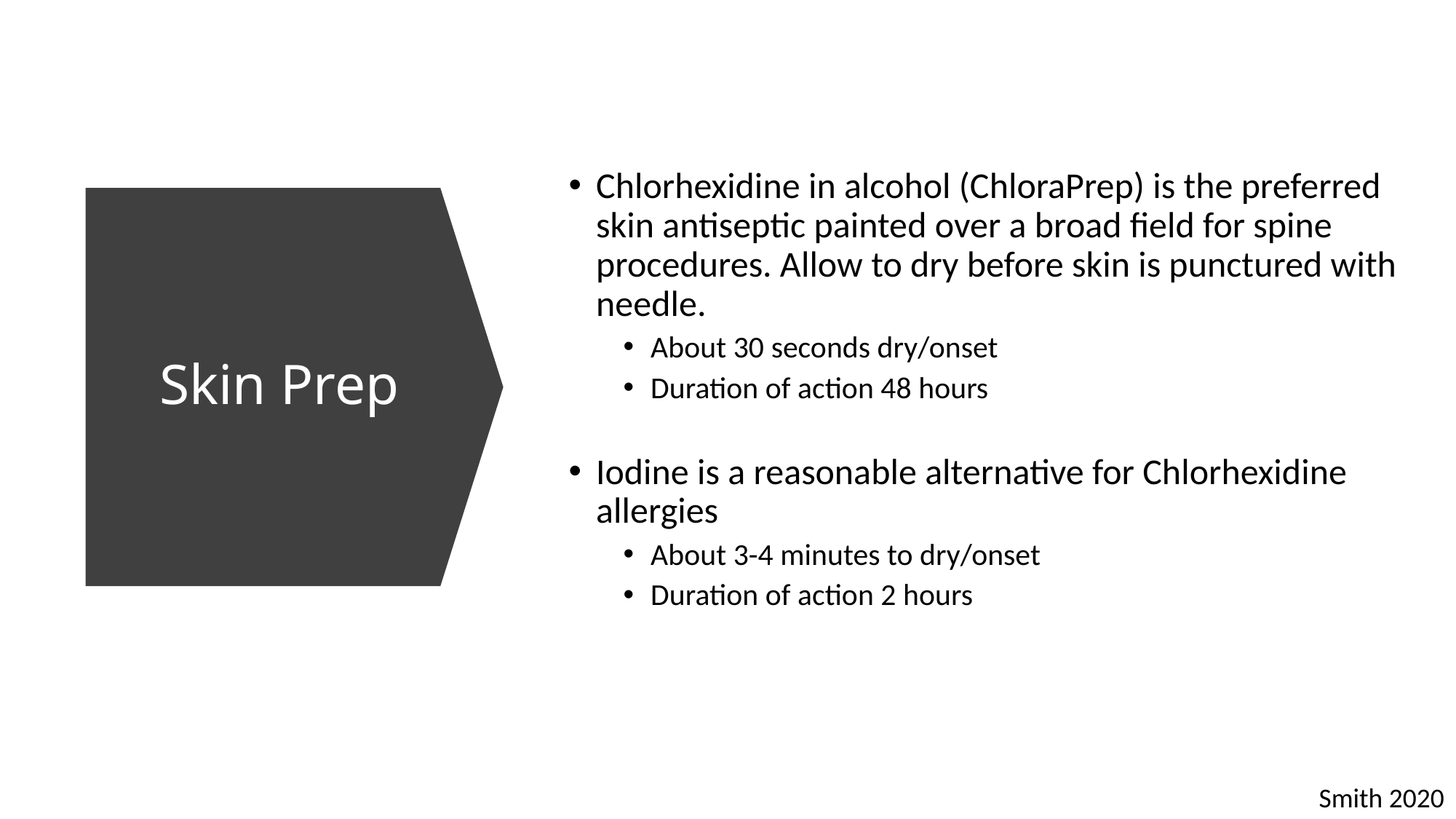

Chlorhexidine in alcohol (ChloraPrep) is the preferred skin antiseptic painted over a broad field for spine procedures. Allow to dry before skin is punctured with needle.
About 30 seconds dry/onset
Duration of action 48 hours
Iodine is a reasonable alternative for Chlorhexidine allergies
About 3-4 minutes to dry/onset
Duration of action 2 hours
# Skin Prep
Smith 2020

## Slide 22
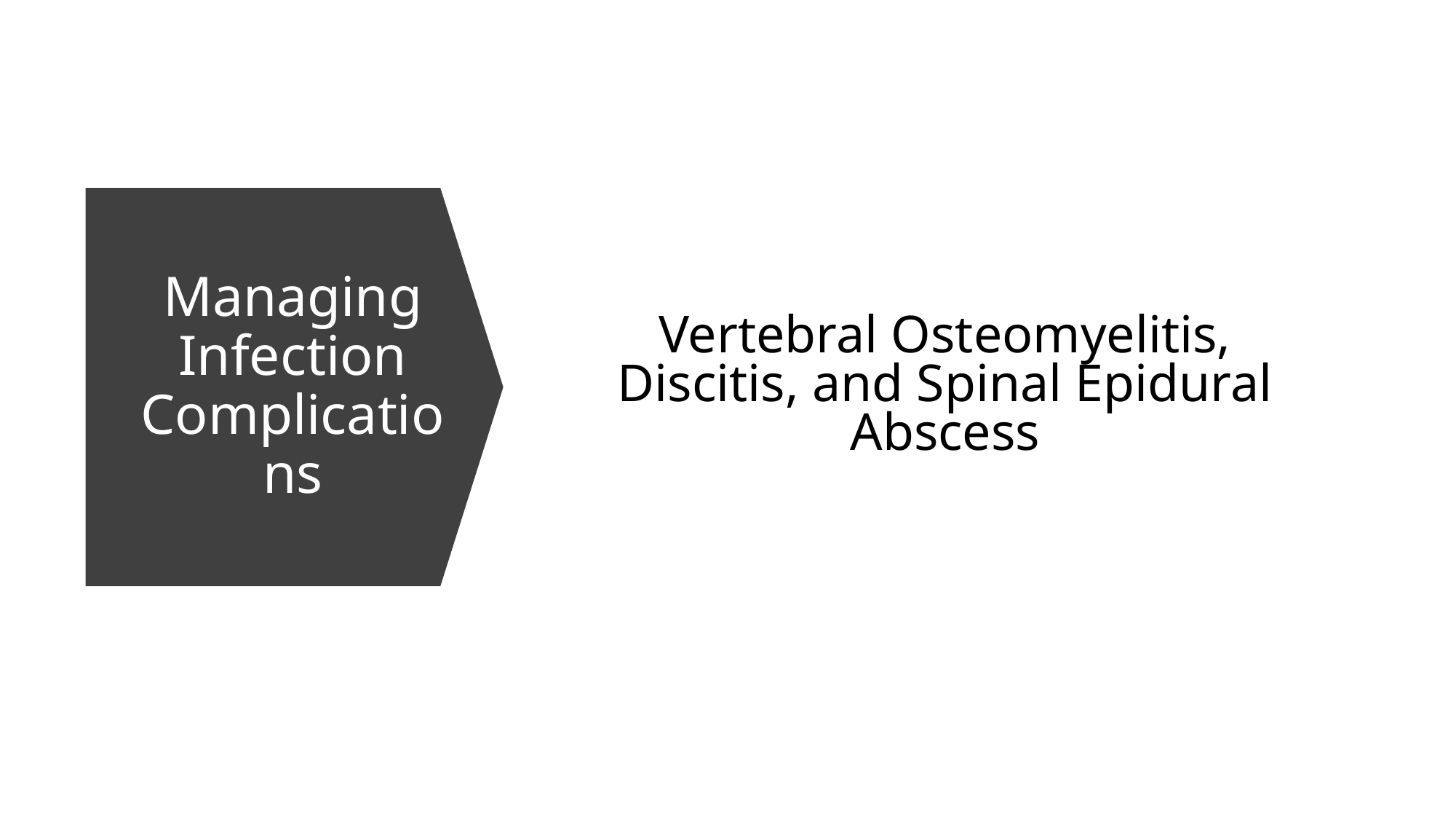

# Managing Infection Complications
Vertebral Osteomyelitis, Discitis, and Spinal Epidural Abscess

## Slide 23
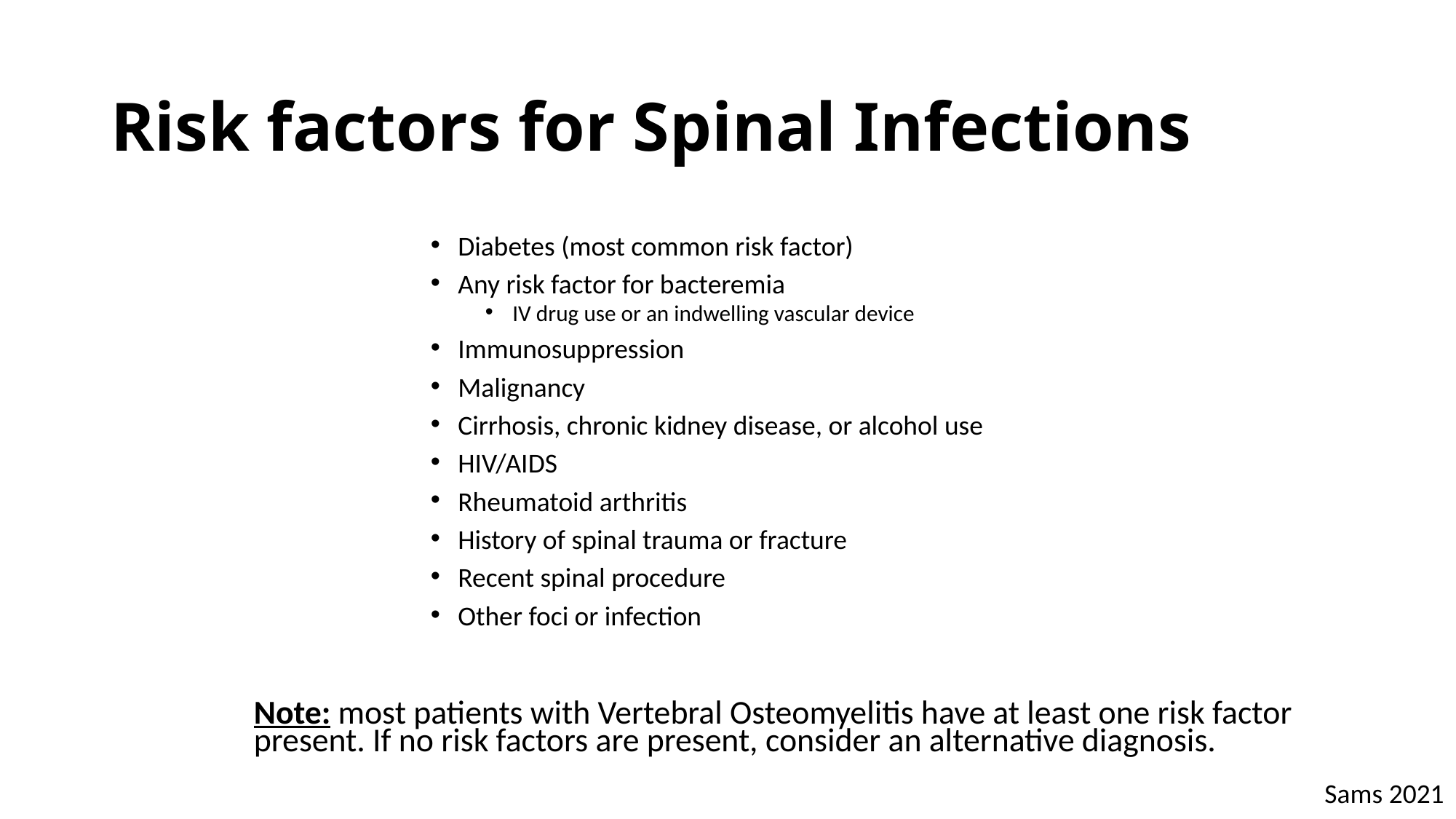

# Risk factors for Spinal Infections
Diabetes (most common risk factor)
Any risk factor for bacteremia
IV drug use or an indwelling vascular device
Immunosuppression
Malignancy
Cirrhosis, chronic kidney disease, or alcohol use
HIV/AIDS
Rheumatoid arthritis
History of spinal trauma or fracture
Recent spinal procedure
Other foci or infection
Note: most patients with Vertebral Osteomyelitis have at least one risk factor present. If no risk factors are present, consider an alternative diagnosis.
Sams 2021

## Slide 24
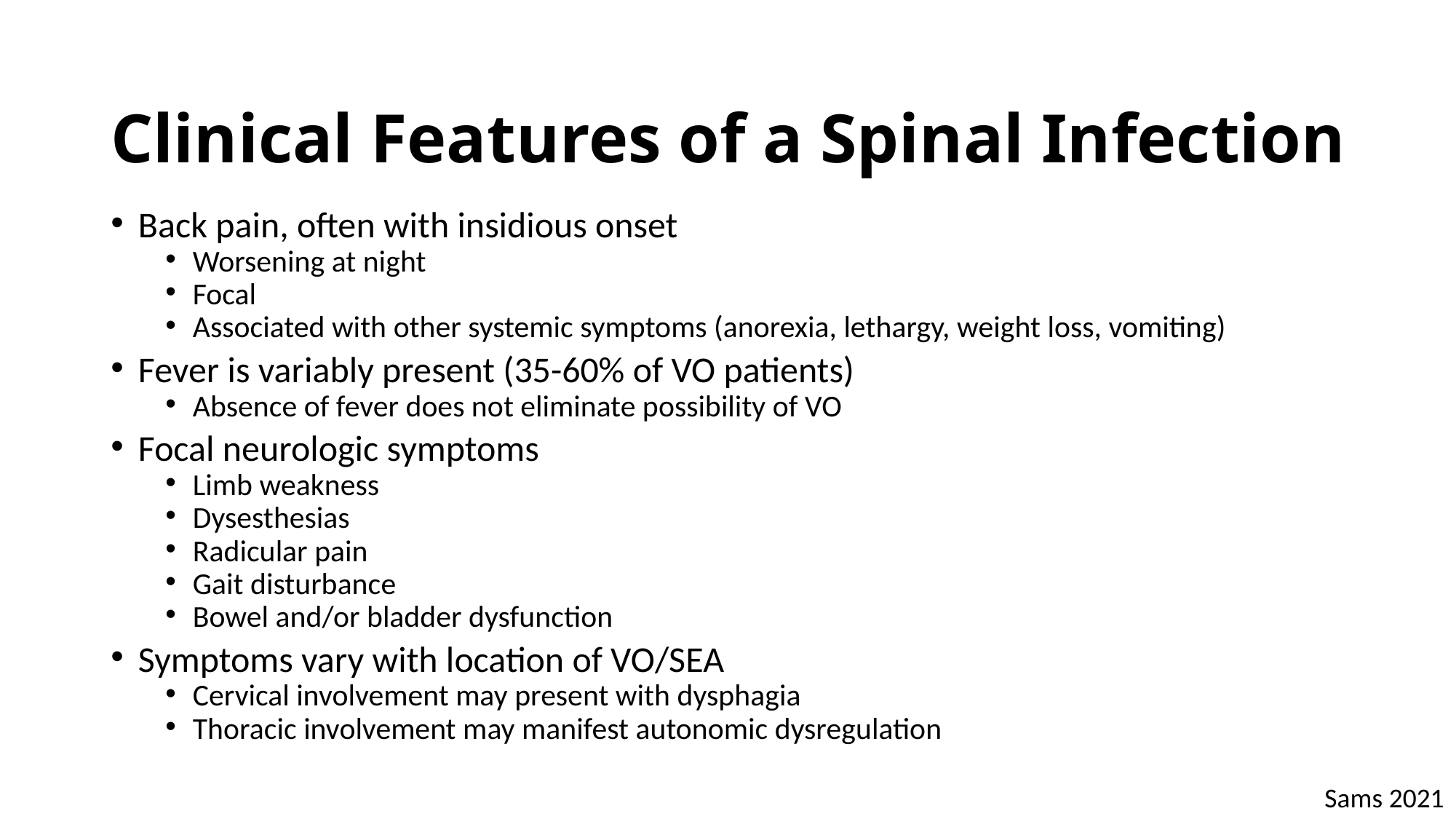

# Clinical Features of a Spinal Infection
Back pain, often with insidious onset
Worsening at night
Focal
Associated with other systemic symptoms (anorexia, lethargy, weight loss, vomiting)
Fever is variably present (35-60% of VO patients)
Absence of fever does not eliminate possibility of VO
Focal neurologic symptoms
Limb weakness
Dysesthesias
Radicular pain
Gait disturbance
Bowel and/or bladder dysfunction
Symptoms vary with location of VO/SEA
Cervical involvement may present with dysphagia
Thoracic involvement may manifest autonomic dysregulation
Sams 2021

## Slide 25
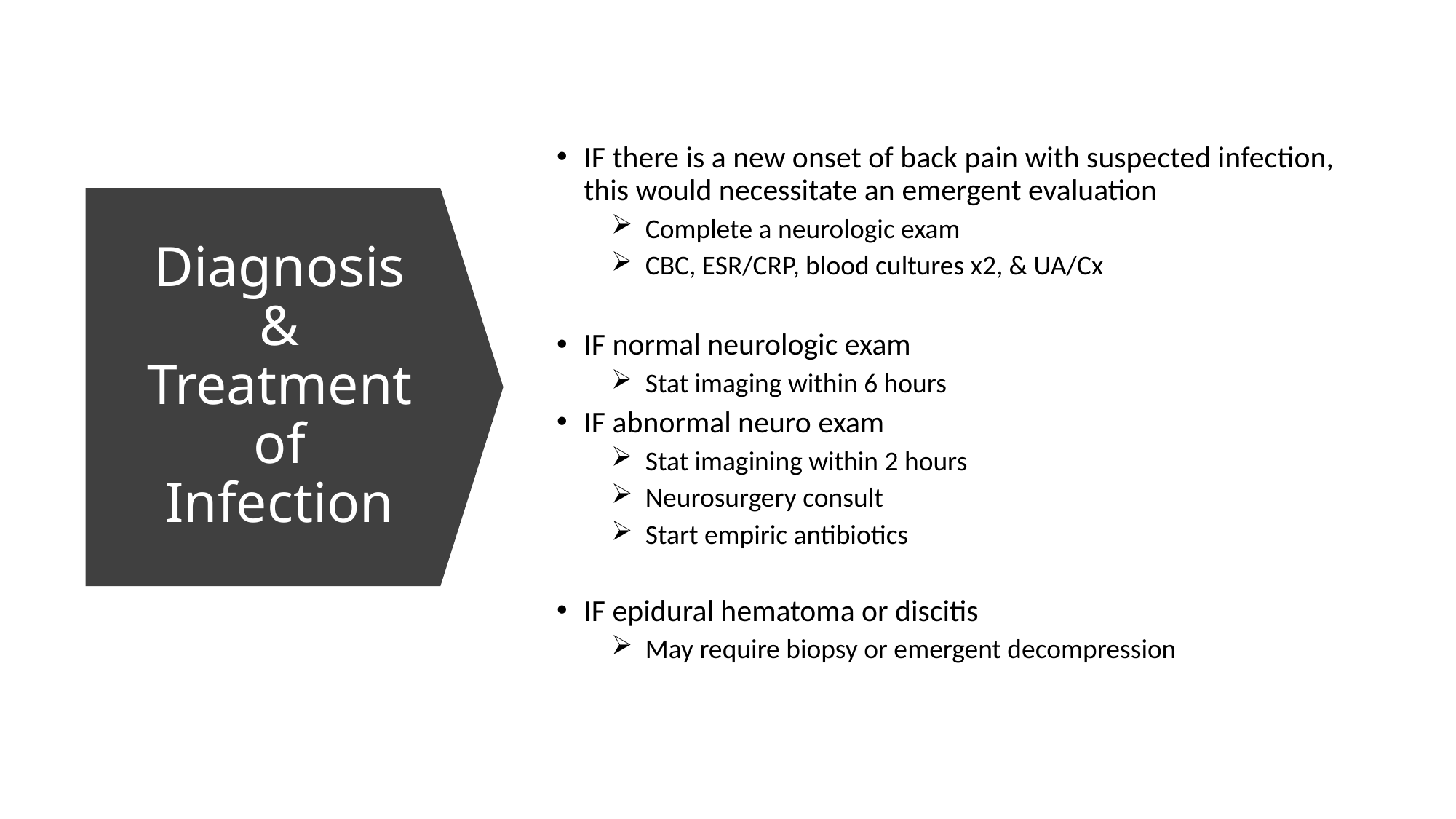

IF there is a new onset of back pain with suspected infection, this would necessitate an emergent evaluation
Complete a neurologic exam
CBC, ESR/CRP, blood cultures x2, & UA/Cx
IF normal neurologic exam
Stat imaging within 6 hours
IF abnormal neuro exam
Stat imagining within 2 hours
Neurosurgery consult
Start empiric antibiotics
IF epidural hematoma or discitis
May require biopsy or emergent decompression
# Diagnosis & Treatment of Infection

## Slide 26
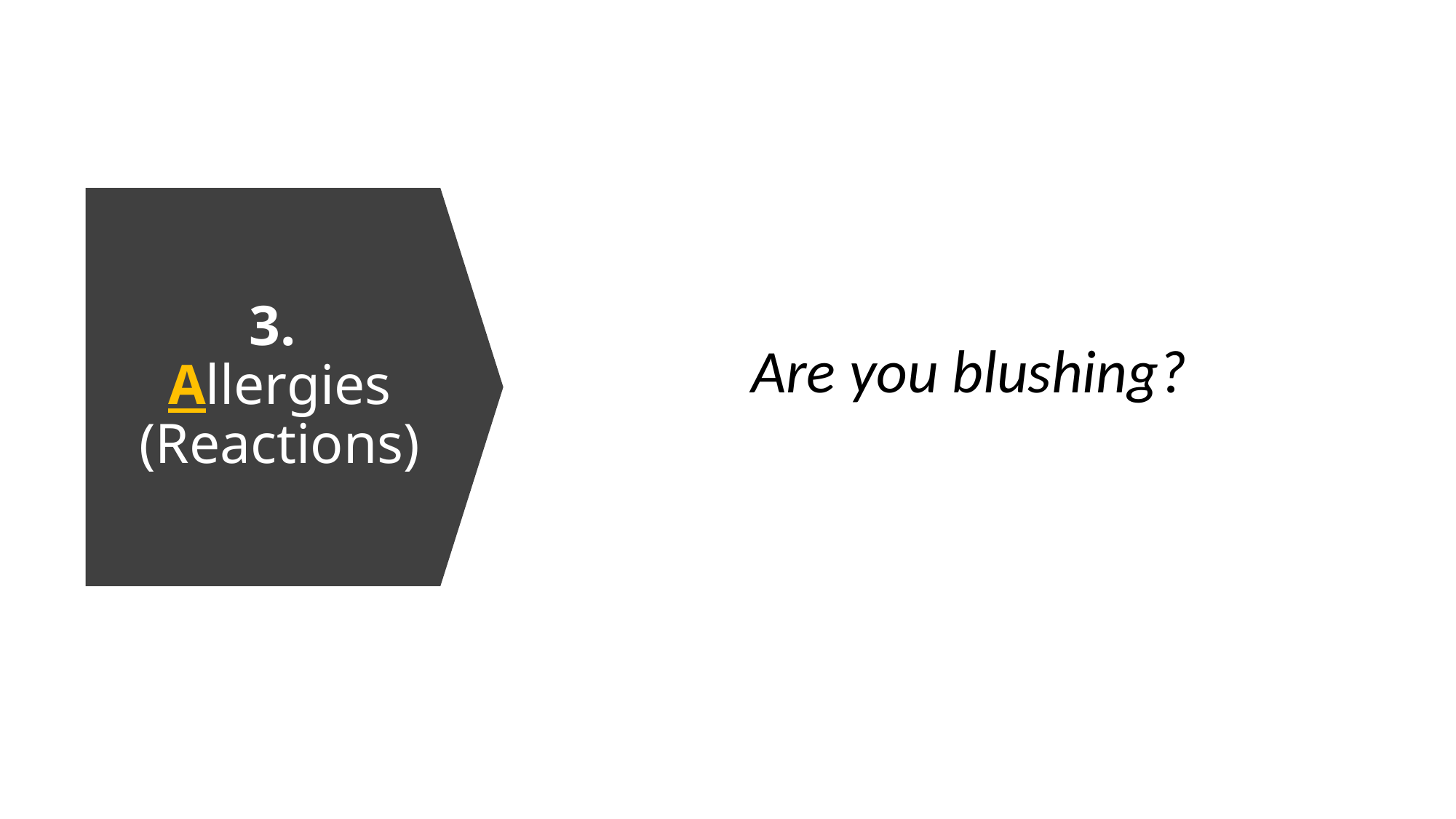

3.
Allergies (Reactions)
Are you blushing?

## Slide 27
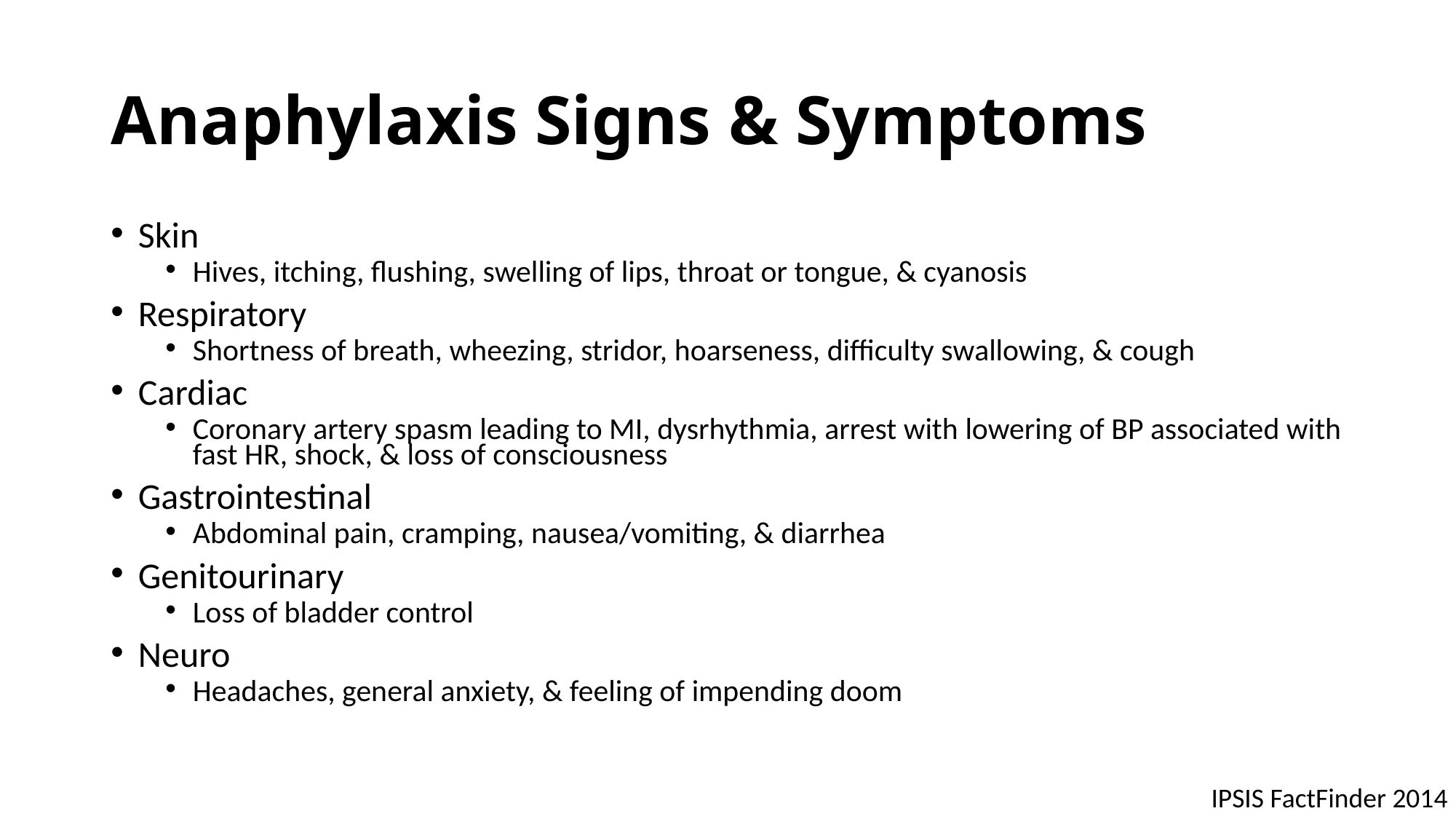

# Anaphylaxis Signs & Symptoms
Skin
Hives, itching, flushing, swelling of lips, throat or tongue, & cyanosis
Respiratory
Shortness of breath, wheezing, stridor, hoarseness, difficulty swallowing, & cough
Cardiac
Coronary artery spasm leading to MI, dysrhythmia, arrest with lowering of BP associated with fast HR, shock, & loss of consciousness
Gastrointestinal
Abdominal pain, cramping, nausea/vomiting, & diarrhea
Genitourinary
Loss of bladder control
Neuro
Headaches, general anxiety, & feeling of impending doom
IPSIS FactFinder 2014

## Slide 28
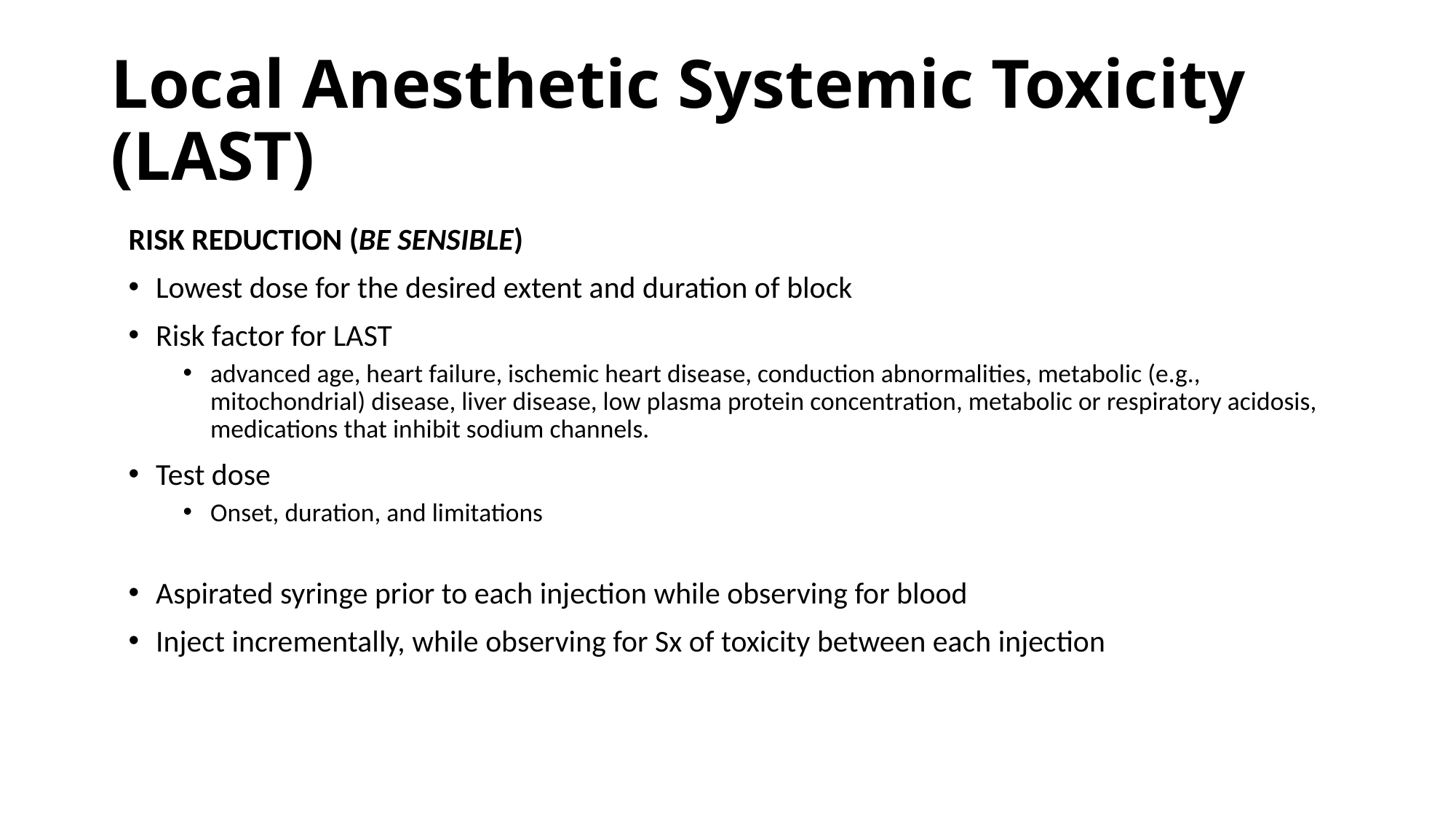

# Local Anesthetic Systemic Toxicity (LAST)
RISK REDUCTION (BE SENSIBLE)
Lowest dose for the desired extent and duration of block
Risk factor for LAST
advanced age, heart failure, ischemic heart disease, conduction abnormalities, metabolic (e.g., mitochondrial) disease, liver disease, low plasma protein concentration, metabolic or respiratory acidosis, medications that inhibit sodium channels.
Test dose
Onset, duration, and limitations
Aspirated syringe prior to each injection while observing for blood
Inject incrementally, while observing for Sx of toxicity between each injection

## Slide 29
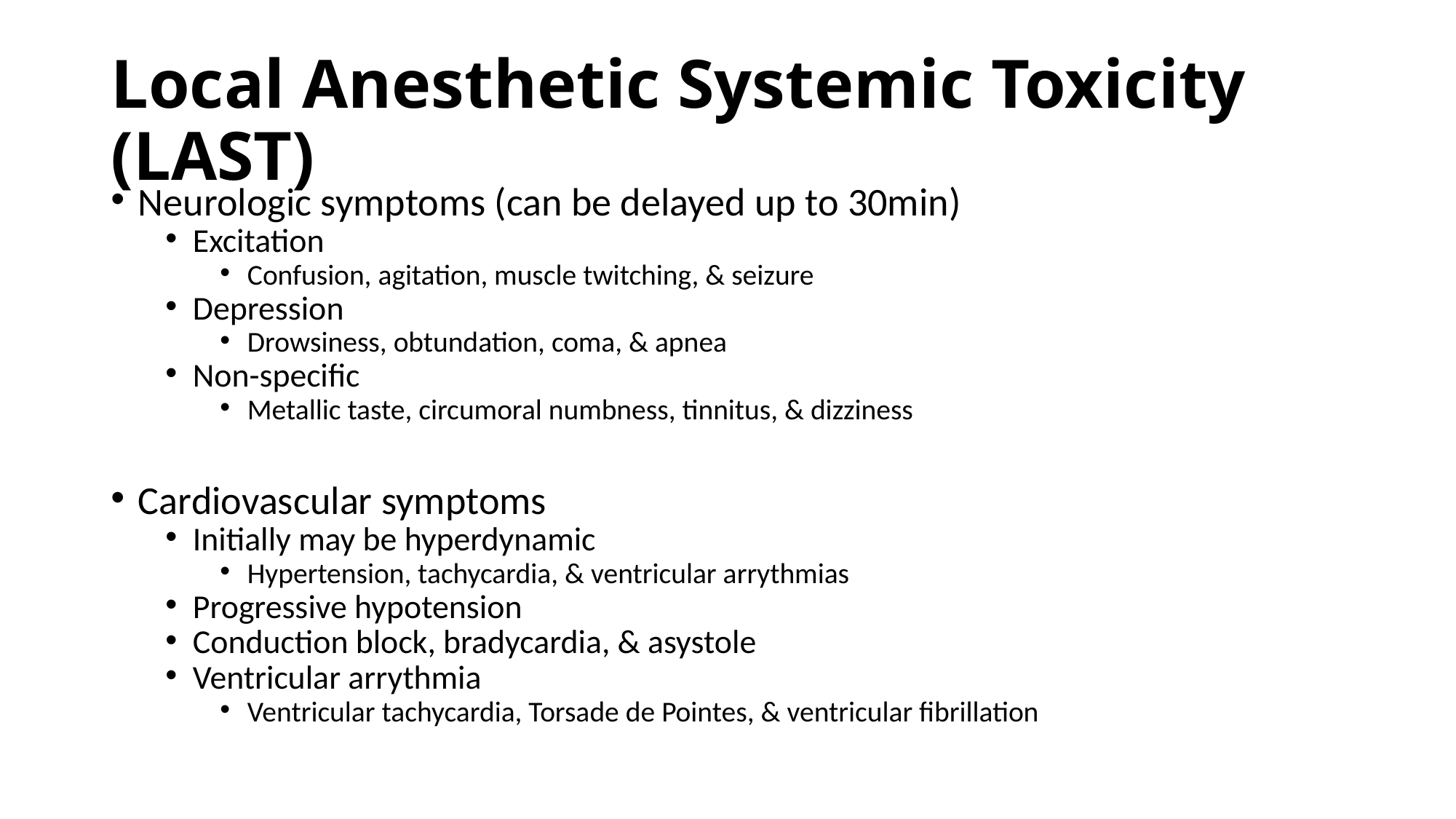

# Local Anesthetic Systemic Toxicity (LAST)
Neurologic symptoms (can be delayed up to 30min)
Excitation
Confusion, agitation, muscle twitching, & seizure
Depression
Drowsiness, obtundation, coma, & apnea
Non-specific
Metallic taste, circumoral numbness, tinnitus, & dizziness
Cardiovascular symptoms
Initially may be hyperdynamic
Hypertension, tachycardia, & ventricular arrythmias
Progressive hypotension
Conduction block, bradycardia, & asystole
Ventricular arrythmia
Ventricular tachycardia, Torsade de Pointes, & ventricular fibrillation

## Slide 30
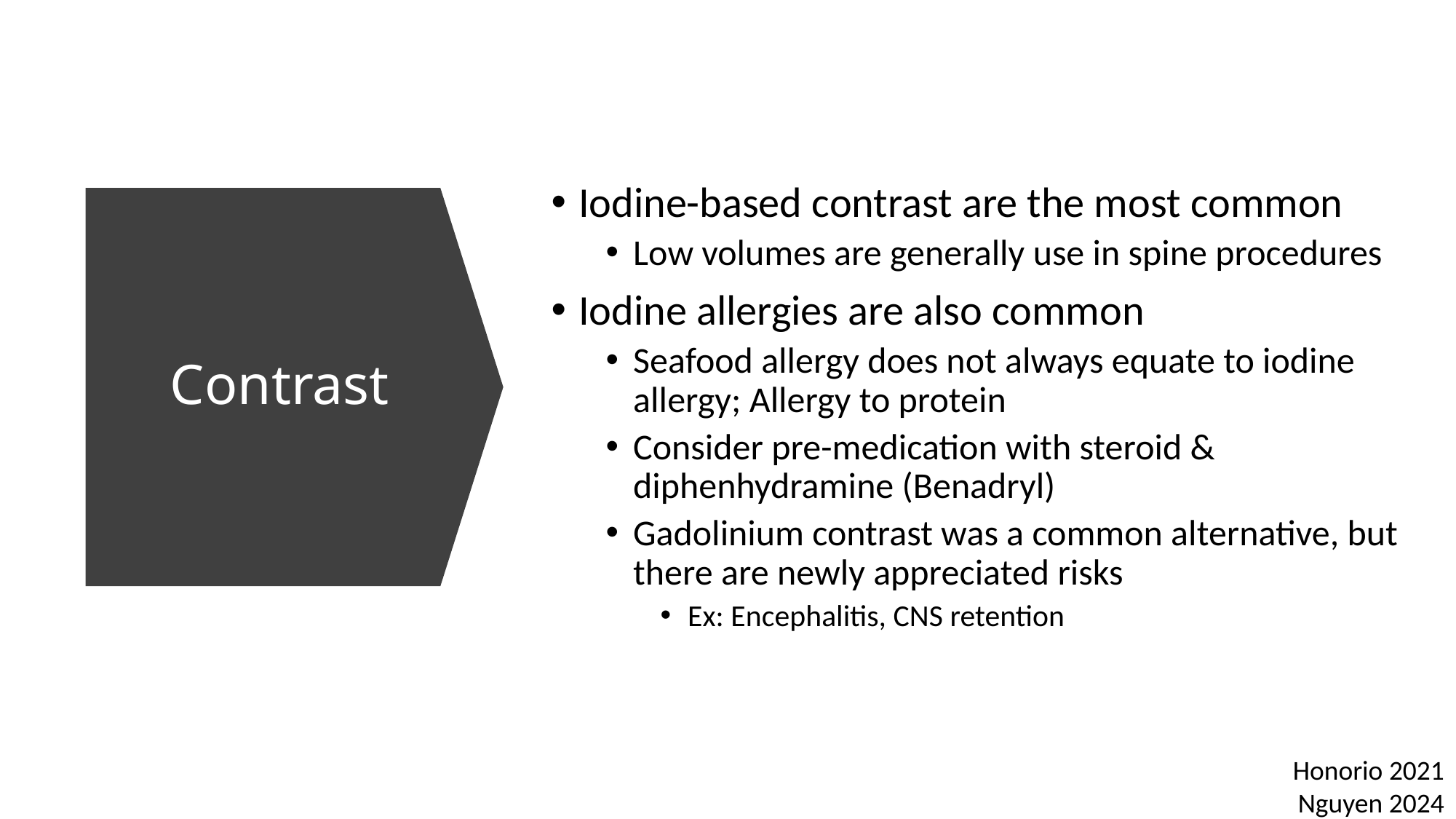

Iodine-based contrast are the most common
Low volumes are generally use in spine procedures
Iodine allergies are also common
Seafood allergy does not always equate to iodine allergy; Allergy to protein
Consider pre-medication with steroid & diphenhydramine (Benadryl)
Gadolinium contrast was a common alternative, but there are newly appreciated risks
Ex: Encephalitis, CNS retention
# Contrast
Honorio 2021
Nguyen 2024

## Slide 31
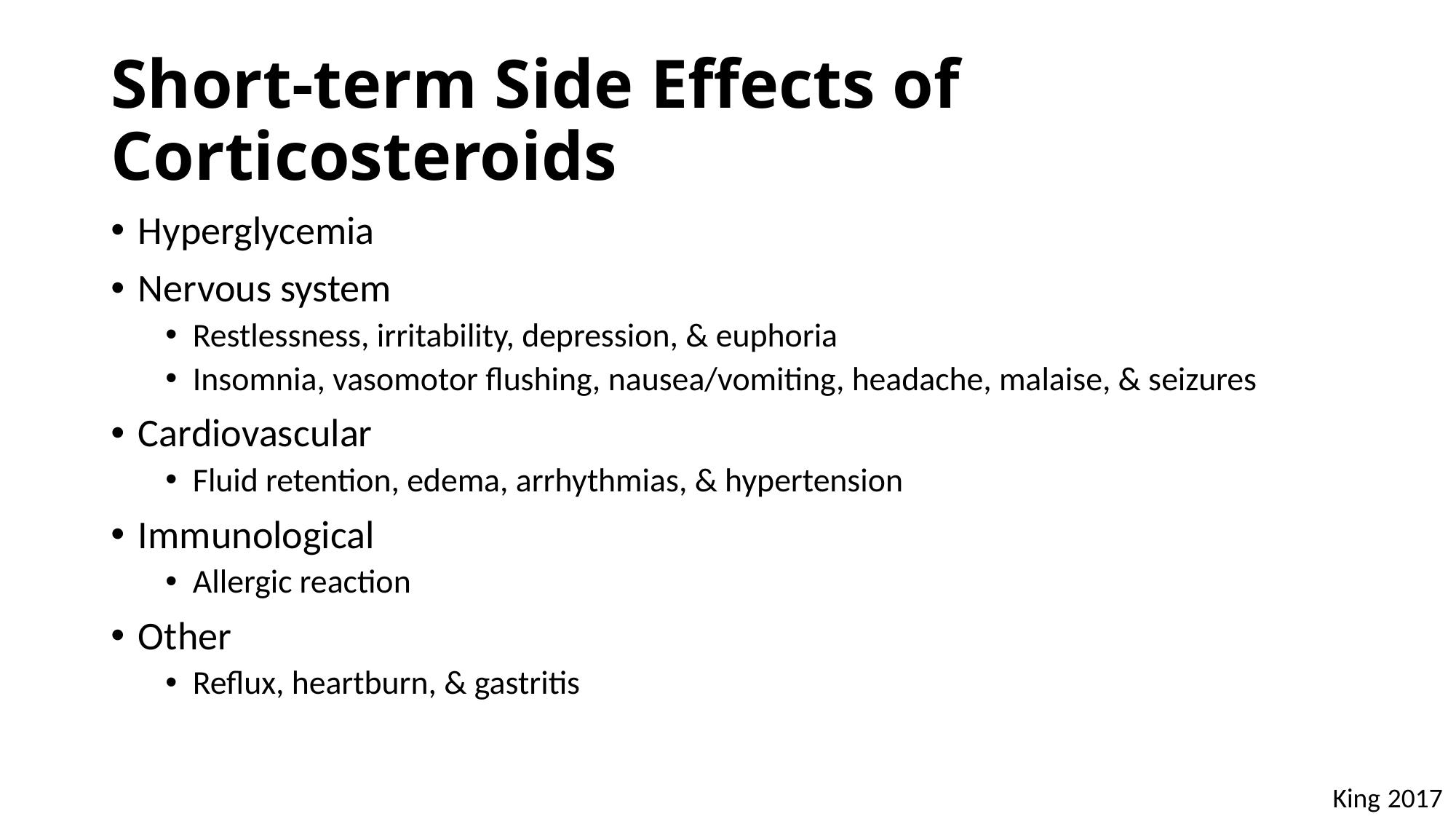

# Short-term Side Effects of Corticosteroids
Hyperglycemia
Nervous system
Restlessness, irritability, depression, & euphoria
Insomnia, vasomotor flushing, nausea/vomiting, headache, malaise, & seizures
Cardiovascular
Fluid retention, edema, arrhythmias, & hypertension
Immunological
Allergic reaction
Other
Reflux, heartburn, & gastritis
King 2017

## Slide 32
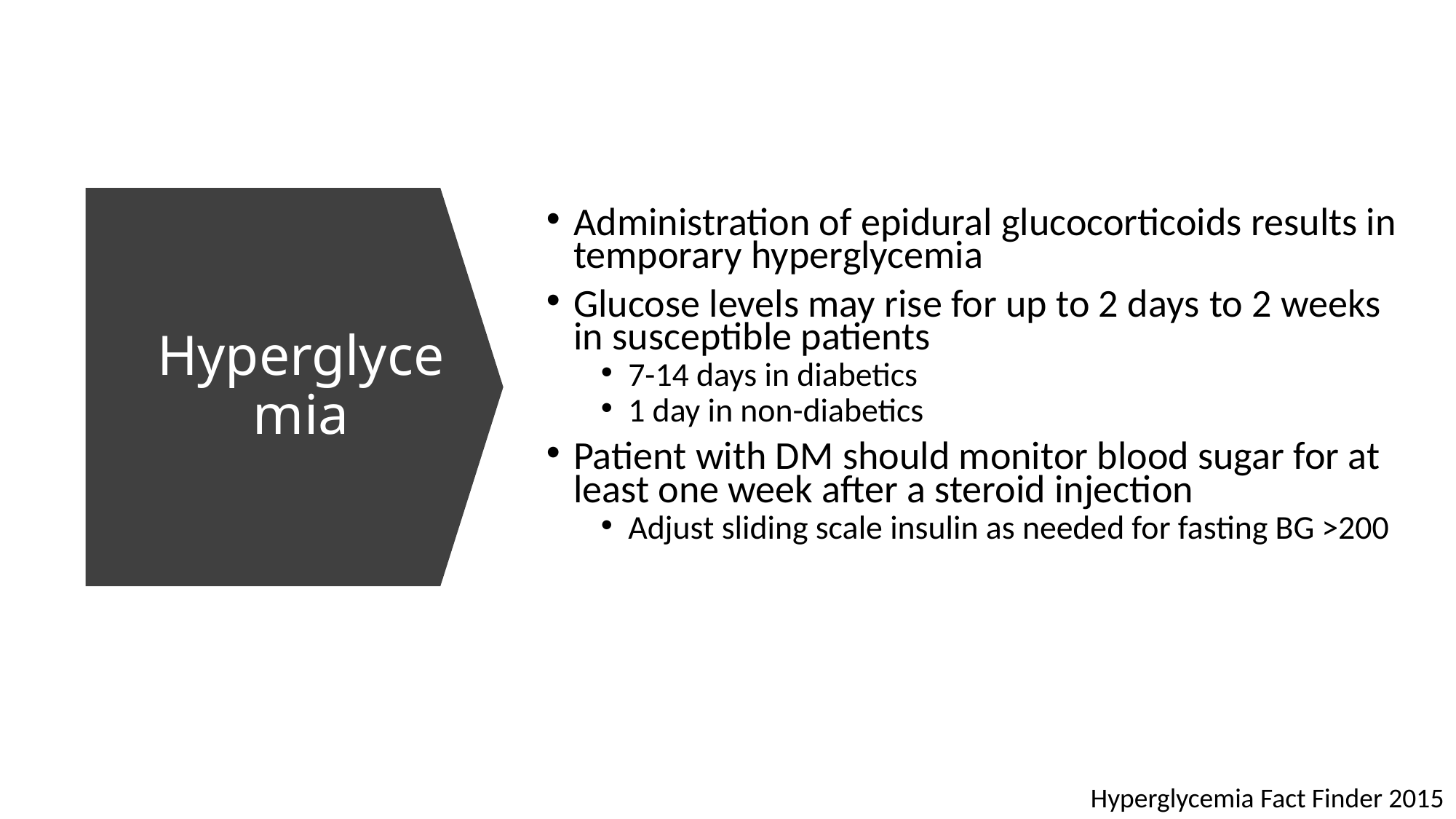

Administration of epidural glucocorticoids results in temporary hyperglycemia
Glucose levels may rise for up to 2 days to 2 weeks in susceptible patients
7-14 days in diabetics
1 day in non-diabetics
Patient with DM should monitor blood sugar for at least one week after a steroid injection
Adjust sliding scale insulin as needed for fasting BG >200
# Hyperglycemia
Hyperglycemia Fact Finder 2015

## Slide 33
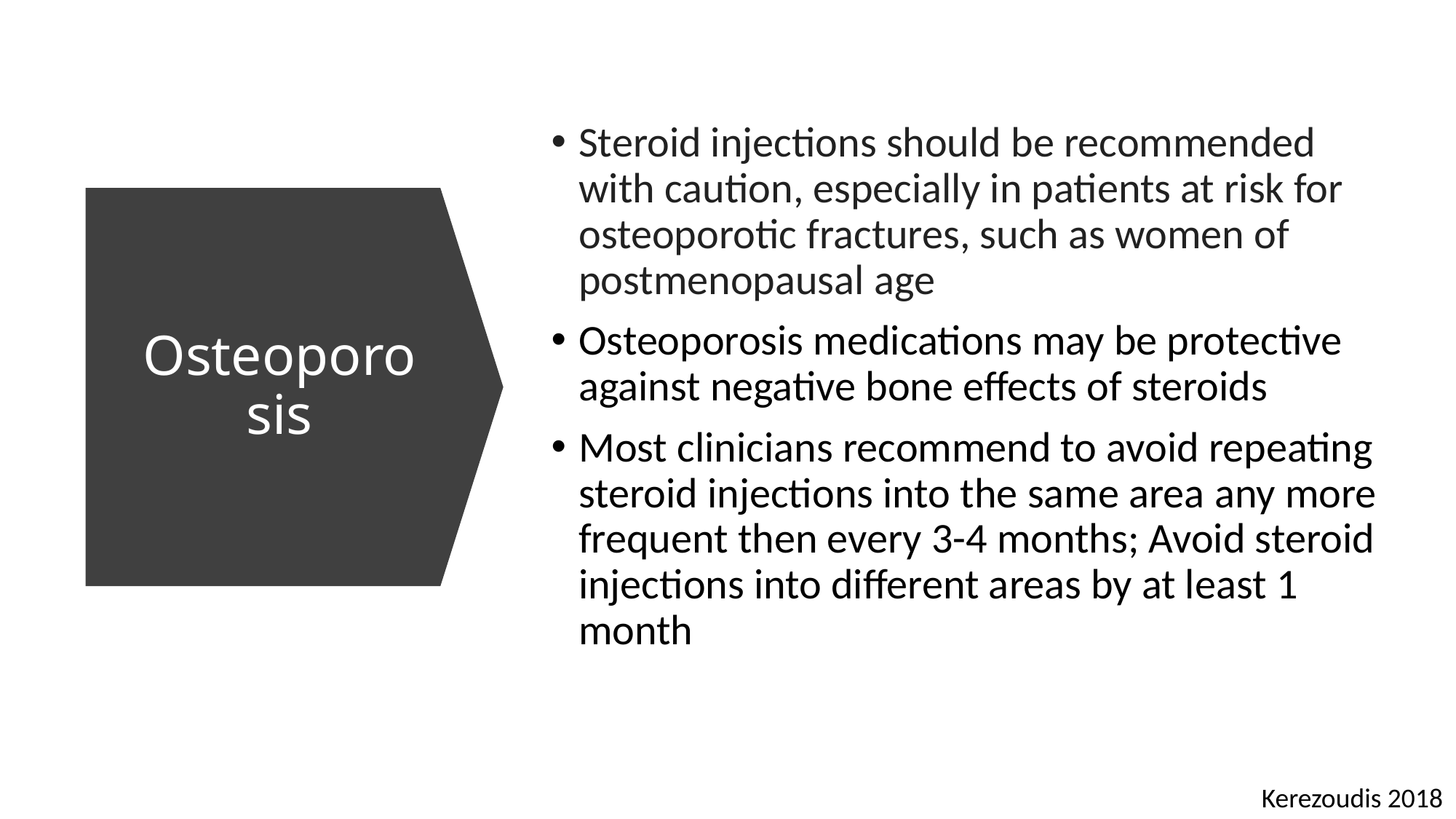

Steroid injections should be recommended with caution, especially in patients at risk for osteoporotic fractures, such as women of postmenopausal age
Osteoporosis medications may be protective against negative bone effects of steroids
Most clinicians recommend to avoid repeating steroid injections into the same area any more frequent then every 3-4 months; Avoid steroid injections into different areas by at least 1 month
# Osteoporosis
Kerezoudis 2018

## Slide 34
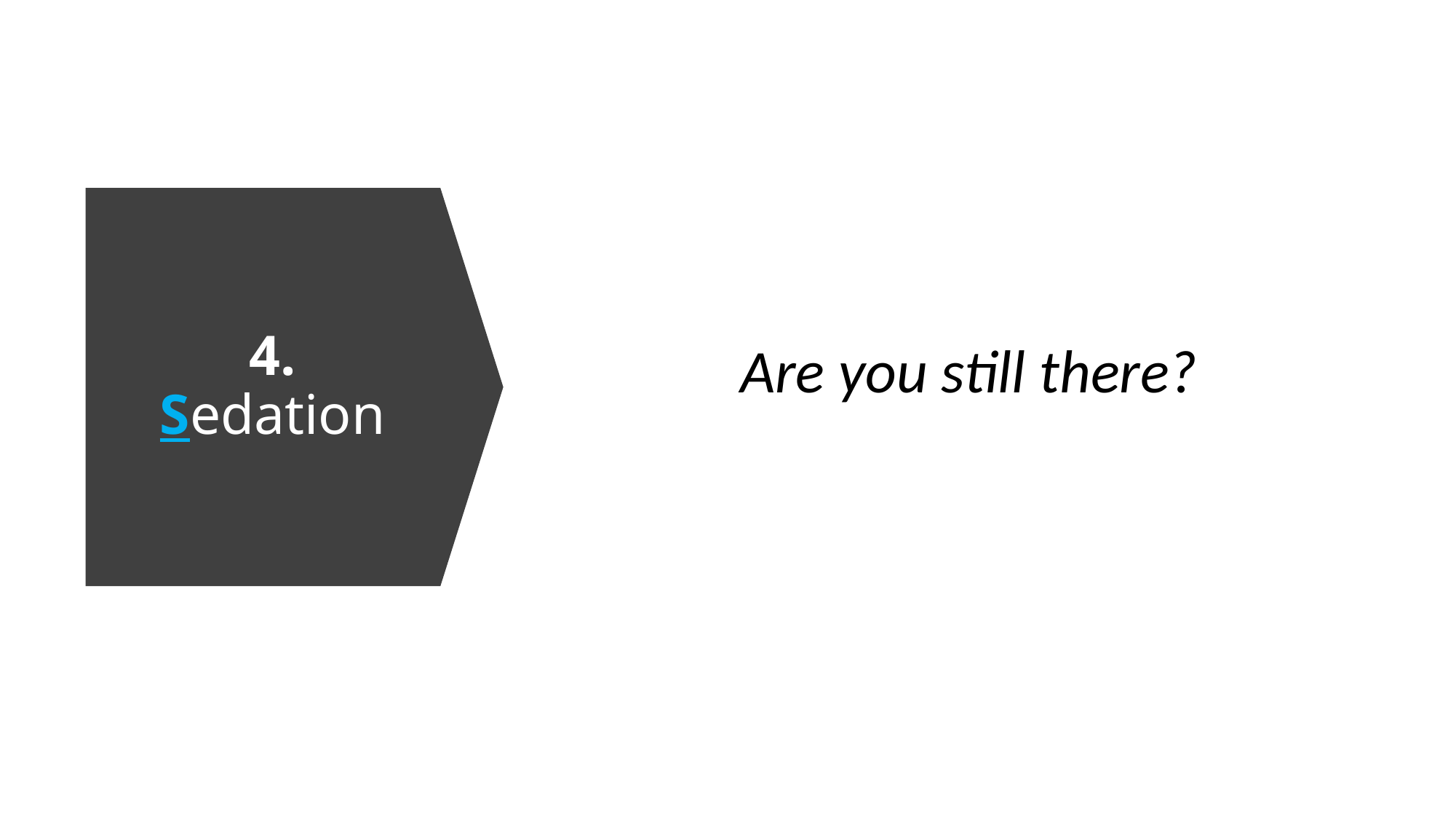

4.
Sedation
Are you still there?

## Slide 35
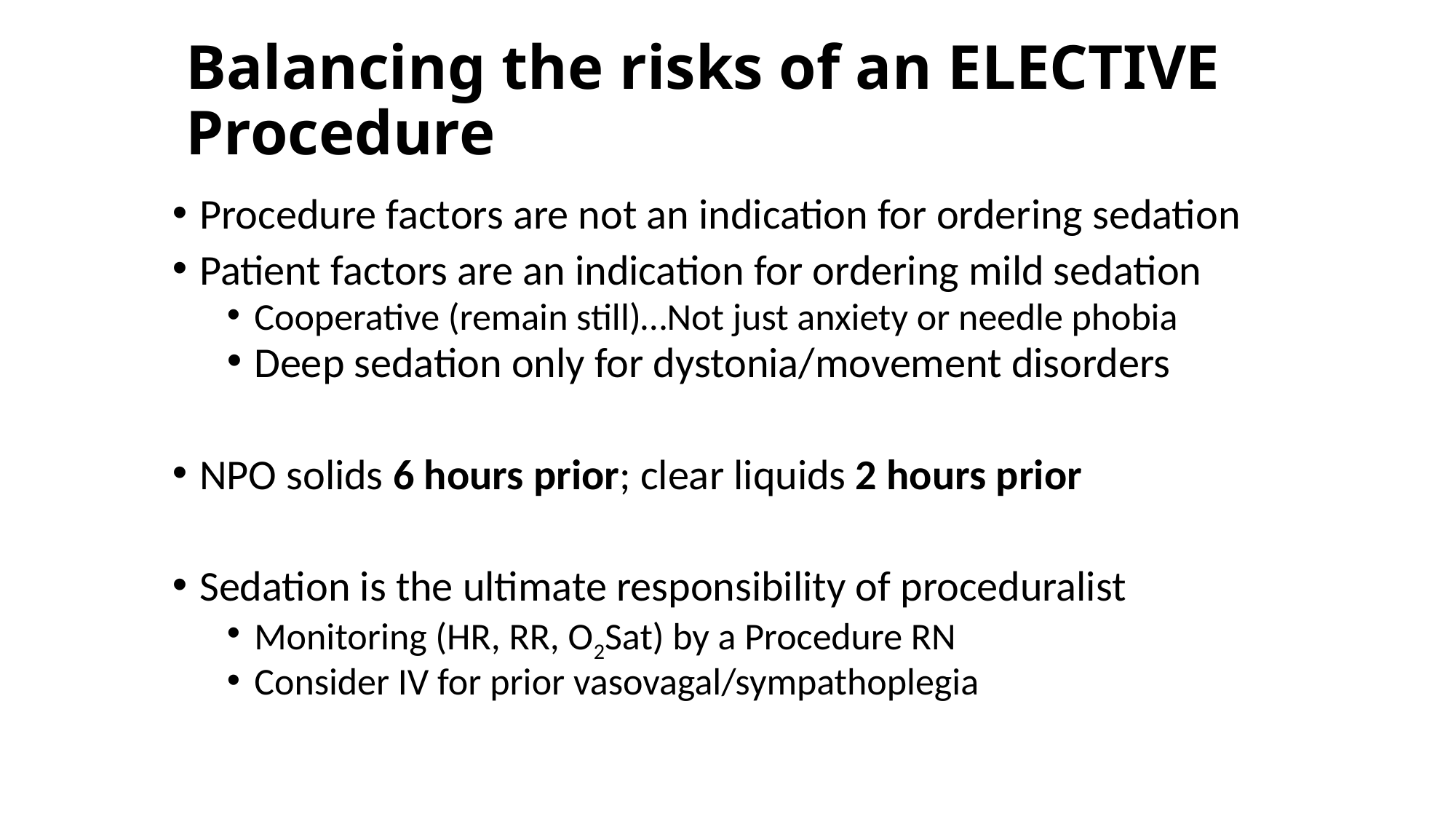

# Balancing the risks of an ELECTIVE Procedure
Procedure factors are not an indication for ordering sedation
Patient factors are an indication for ordering mild sedation
Cooperative (remain still)…Not just anxiety or needle phobia
Deep sedation only for dystonia/movement disorders
NPO solids 6 hours prior; clear liquids 2 hours prior
Sedation is the ultimate responsibility of proceduralist
Monitoring (HR, RR, O2Sat) by a Procedure RN
Consider IV for prior vasovagal/sympathoplegia

## Slide 36
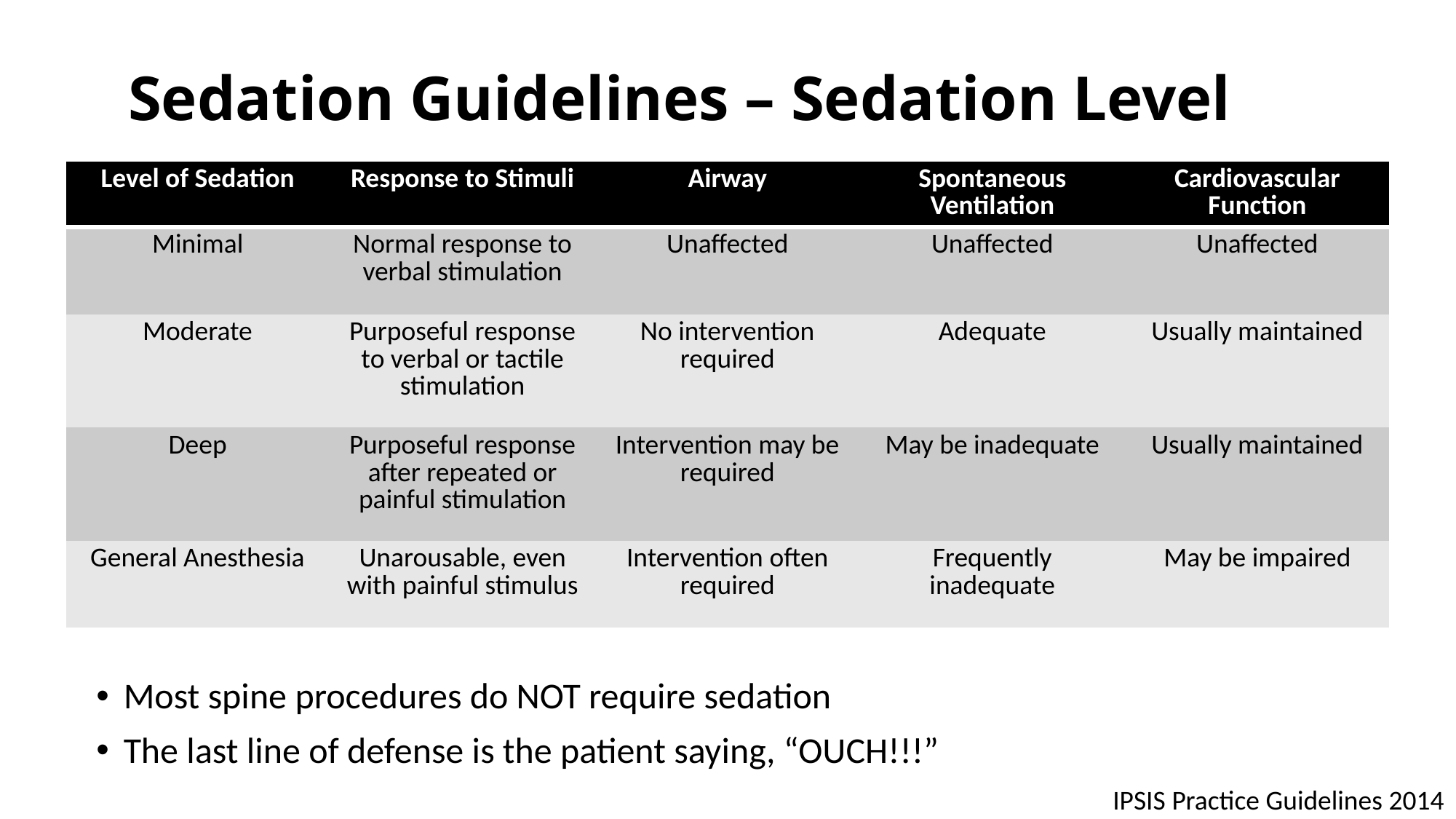

# Sedation Guidelines – Sedation Level
| Level of Sedation | Response to Stimuli | Airway | Spontaneous Ventilation | Cardiovascular Function |
| --- | --- | --- | --- | --- |
| Minimal | Normal response to verbal stimulation | Unaffected | Unaffected | Unaffected |
| Moderate | Purposeful response to verbal or tactile stimulation | No intervention required | Adequate | Usually maintained |
| Deep | Purposeful response after repeated or painful stimulation | Intervention may be required | May be inadequate | Usually maintained |
| General Anesthesia | Unarousable, even with painful stimulus | Intervention often required | Frequently inadequate | May be impaired |
Most spine procedures do NOT require sedation
The last line of defense is the patient saying, “OUCH!!!”
IPSIS Practice Guidelines 2014

## Slide 37
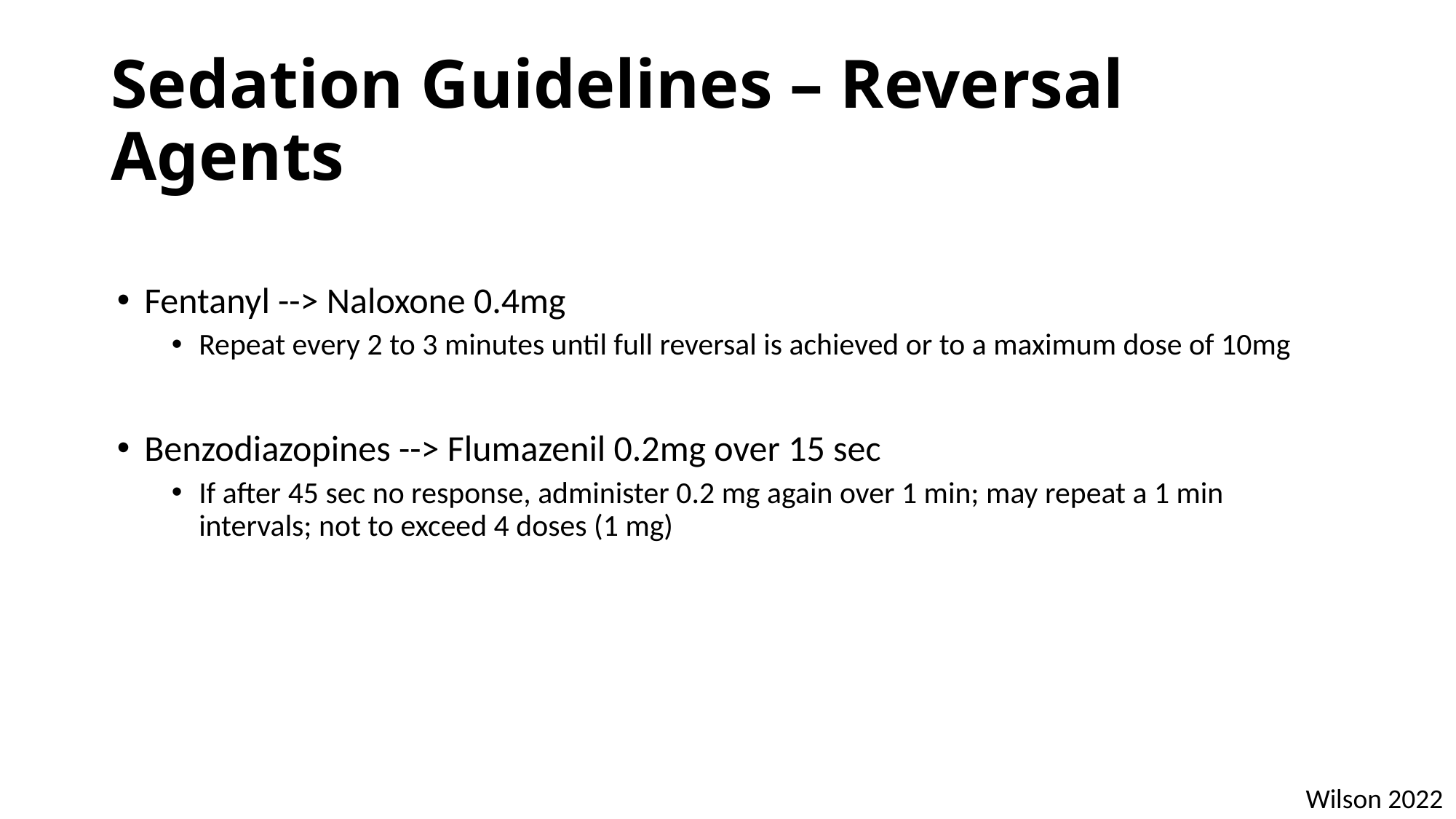

# Sedation Guidelines – Reversal Agents
Fentanyl --> Naloxone 0.4mg
Repeat every 2 to 3 minutes until full reversal is achieved or to a maximum dose of 10mg
Benzodiazopines --> Flumazenil 0.2mg over 15 sec
If after 45 sec no response, administer 0.2 mg again over 1 min; may repeat a 1 min intervals; not to exceed 4 doses (1 mg)
Wilson 2022

## Slide 38
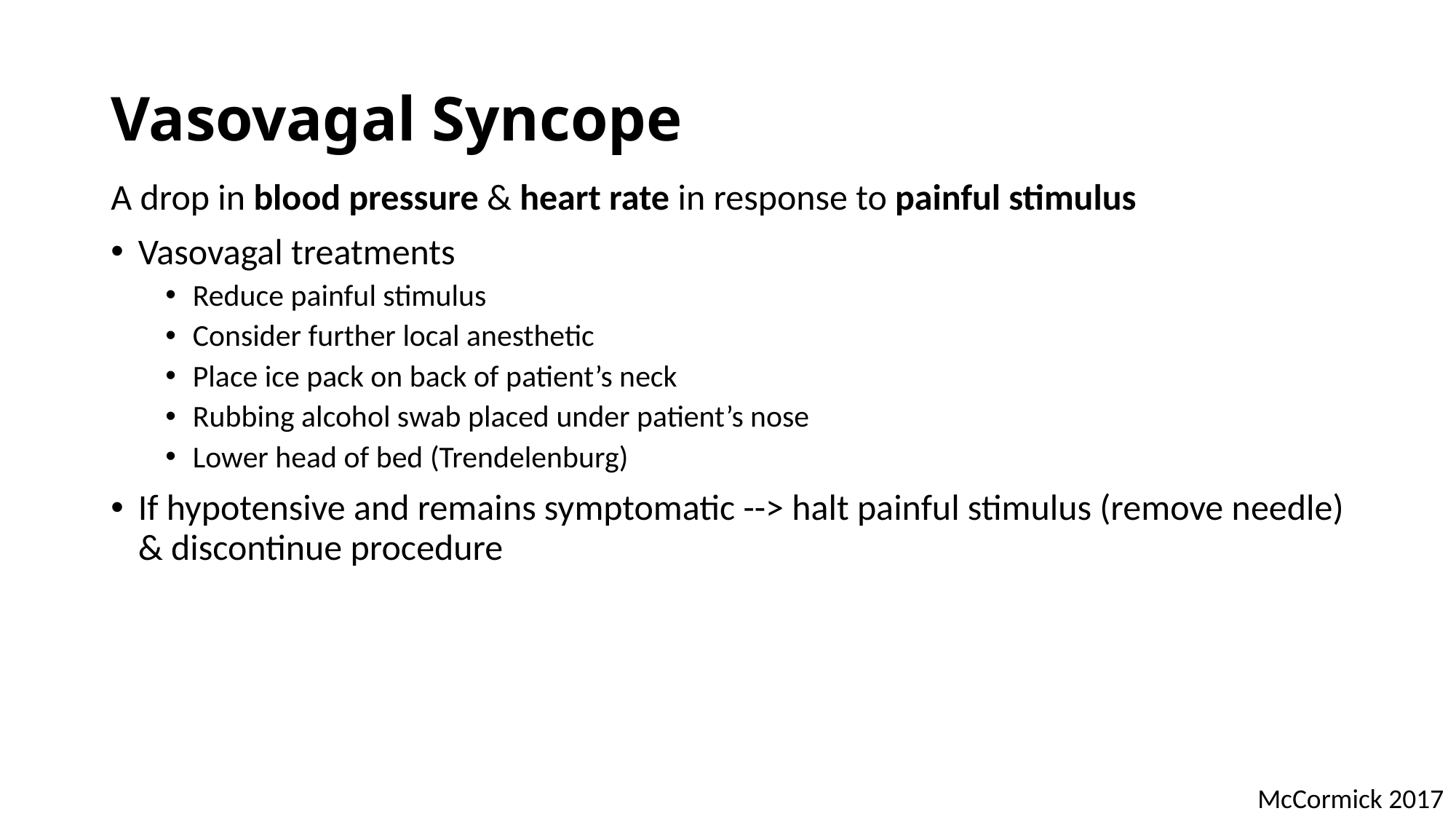

# Vasovagal Syncope
A drop in blood pressure & heart rate in response to painful stimulus
Vasovagal treatments
Reduce painful stimulus
Consider further local anesthetic
Place ice pack on back of patient’s neck
Rubbing alcohol swab placed under patient’s nose
Lower head of bed (Trendelenburg)
If hypotensive and remains symptomatic --> halt painful stimulus (remove needle) & discontinue procedure
McCormick 2017

## Slide 39
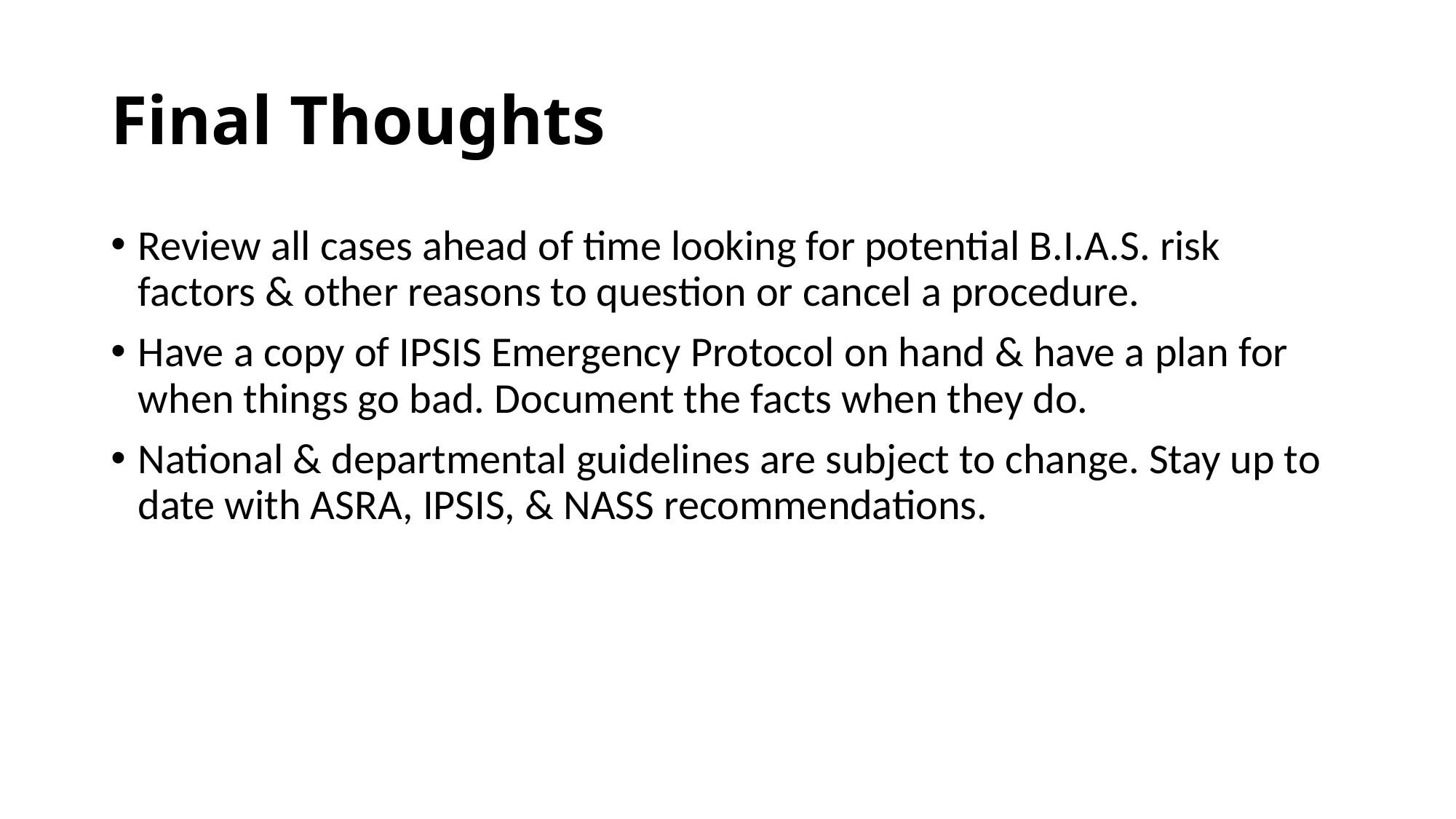

# Final Thoughts
Review all cases ahead of time looking for potential B.I.A.S. risk factors & other reasons to question or cancel a procedure.
Have a copy of IPSIS Emergency Protocol on hand & have a plan for when things go bad. Document the facts when they do.
National & departmental guidelines are subject to change. Stay up to date with ASRA, IPSIS, & NASS recommendations.

## Slide 40
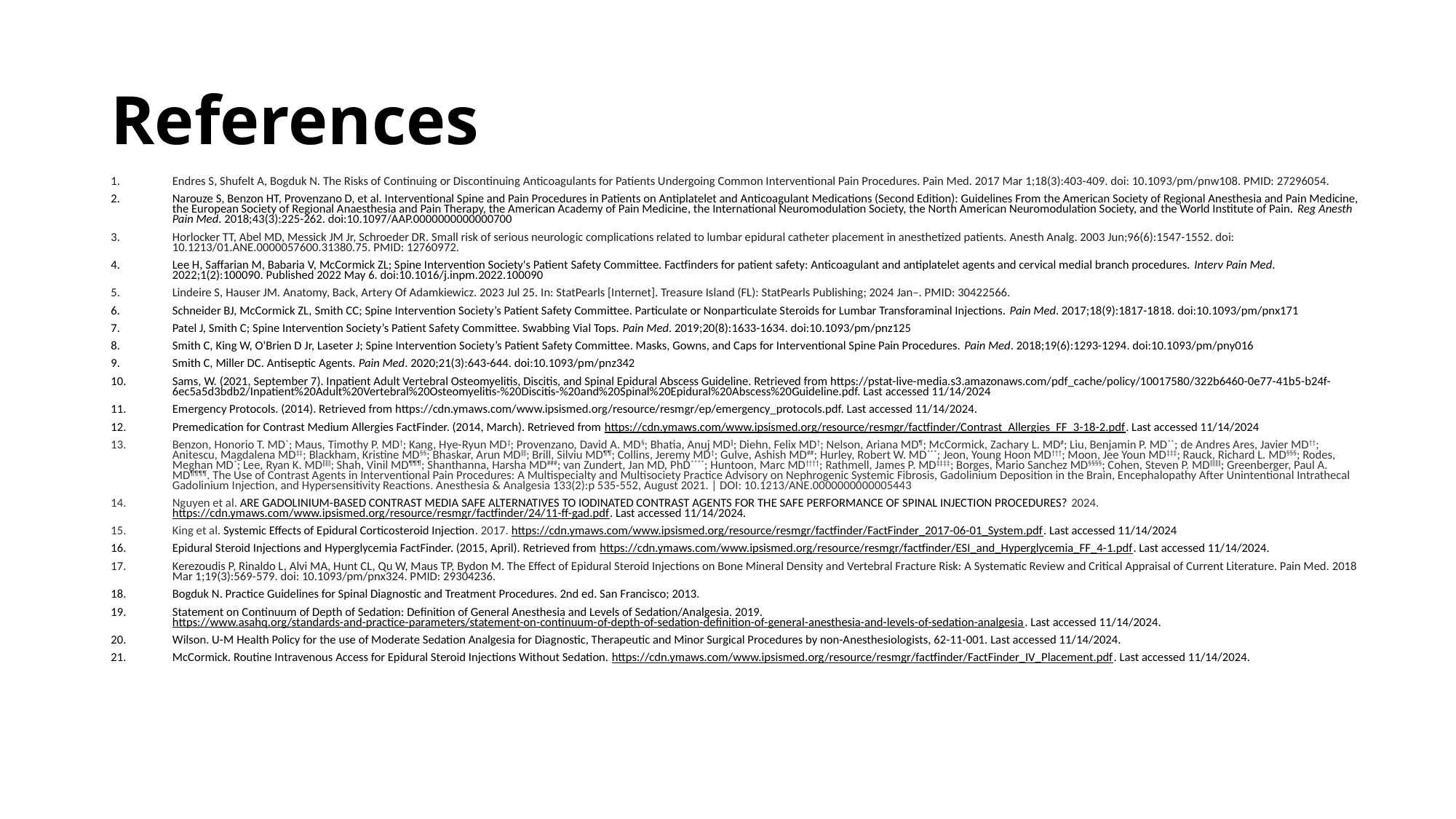

# References
Endres S, Shufelt A, Bogduk N. The Risks of Continuing or Discontinuing Anticoagulants for Patients Undergoing Common Interventional Pain Procedures. Pain Med. 2017 Mar 1;18(3):403-409. doi: 10.1093/pm/pnw108. PMID: 27296054.
Narouze S, Benzon HT, Provenzano D, et al. Interventional Spine and Pain Procedures in Patients on Antiplatelet and Anticoagulant Medications (Second Edition): Guidelines From the American Society of Regional Anesthesia and Pain Medicine, the European Society of Regional Anaesthesia and Pain Therapy, the American Academy of Pain Medicine, the International Neuromodulation Society, the North American Neuromodulation Society, and the World Institute of Pain. Reg Anesth Pain Med. 2018;43(3):225-262. doi:10.1097/AAP.0000000000000700
Horlocker TT, Abel MD, Messick JM Jr, Schroeder DR. Small risk of serious neurologic complications related to lumbar epidural catheter placement in anesthetized patients. Anesth Analg. 2003 Jun;96(6):1547-1552. doi: 10.1213/01.ANE.0000057600.31380.75. PMID: 12760972.
Lee H, Saffarian M, Babaria V, McCormick ZL; Spine Intervention Society's Patient Safety Committee. Factfinders for patient safety: Anticoagulant and antiplatelet agents and cervical medial branch procedures. Interv Pain Med. 2022;1(2):100090. Published 2022 May 6. doi:10.1016/j.inpm.2022.100090
Lindeire S, Hauser JM. Anatomy, Back, Artery Of Adamkiewicz. 2023 Jul 25. In: StatPearls [Internet]. Treasure Island (FL): StatPearls Publishing; 2024 Jan–. PMID: 30422566.
Schneider BJ, McCormick ZL, Smith CC; Spine Intervention Society’s Patient Safety Committee. Particulate or Nonparticulate Steroids for Lumbar Transforaminal Injections. Pain Med. 2017;18(9):1817-1818. doi:10.1093/pm/pnx171
Patel J, Smith C; Spine Intervention Society’s Patient Safety Committee. Swabbing Vial Tops. Pain Med. 2019;20(8):1633-1634. doi:10.1093/pm/pnz125
Smith C, King W, O'Brien D Jr, Laseter J; Spine Intervention Society’s Patient Safety Committee. Masks, Gowns, and Caps for Interventional Spine Pain Procedures. Pain Med. 2018;19(6):1293-1294. doi:10.1093/pm/pny016
Smith C, Miller DC. Antiseptic Agents. Pain Med. 2020;21(3):643-644. doi:10.1093/pm/pnz342
Sams, W. (2021, September 7). Inpatient Adult Vertebral Osteomyelitis, Discitis, and Spinal Epidural Abscess Guideline. Retrieved from https://pstat-live-media.s3.amazonaws.com/pdf_cache/policy/10017580/322b6460-0e77-41b5-b24f-6ec5a5d3bdb2/Inpatient%20Adult%20Vertebral%20Osteomyelitis-%20Discitis-%20and%20Spinal%20Epidural%20Abscess%20Guideline.pdf. Last accessed 11/14/2024
Emergency Protocols. (2014). Retrieved from https://cdn.ymaws.com/www.ipsismed.org/resource/resmgr/ep/emergency_protocols.pdf. Last accessed 11/14/2024.
Premedication for Contrast Medium Allergies FactFinder. (2014, March). Retrieved from https://cdn.ymaws.com/www.ipsismed.org/resource/resmgr/factfinder/Contrast_Allergies_FF_3-18-2.pdf. Last accessed 11/14/2024
Benzon, Honorio T. MD*; Maus, Timothy P. MD†; Kang, Hye-Ryun MD‡; Provenzano, David A. MD§; Bhatia, Anuj MD‖; Diehn, Felix MD†; Nelson, Ariana MD¶; McCormick, Zachary L. MD#; Liu, Benjamin P. MD**; de Andres Ares, Javier MD††; Anitescu, Magdalena MD‡‡; Blackham, Kristine MD§§; Bhaskar, Arun MD‖‖; Brill, Silviu MD¶¶; Collins, Jeremy MD†; Gulve, Ashish MD##; Hurley, Robert W. MD***; Jeon, Young Hoon MD†††; Moon, Jee Youn MD‡‡‡; Rauck, Richard L. MD§§§; Rodes, Meghan MD*; Lee, Ryan K. MD‖‖‖; Shah, Vinil MD¶¶¶; Shanthanna, Harsha MD###; van Zundert, Jan MD, PhD****; Huntoon, Marc MD††††; Rathmell, James P. MD‡‡‡‡; Borges, Mario Sanchez MD§§§§; Cohen, Steven P. MD‖‖‖‖; Greenberger, Paul A. MD¶¶¶¶. The Use of Contrast Agents in Interventional Pain Procedures: A Multispecialty and Multisociety Practice Advisory on Nephrogenic Systemic Fibrosis, Gadolinium Deposition in the Brain, Encephalopathy After Unintentional Intrathecal Gadolinium Injection, and Hypersensitivity Reactions. Anesthesia & Analgesia 133(2):p 535-552, August 2021. | DOI: 10.1213/ANE.0000000000005443
Nguyen et al. ARE GADOLINIUM-BASED CONTRAST MEDIA SAFE ALTERNATIVES TO IODINATED CONTRAST AGENTS FOR THE SAFE PERFORMANCE OF SPINAL INJECTION PROCEDURES? 2024. https://cdn.ymaws.com/www.ipsismed.org/resource/resmgr/factfinder/24/11-ff-gad.pdf. Last accessed 11/14/2024.
King et al. Systemic Effects of Epidural Corticosteroid Injection. 2017. https://cdn.ymaws.com/www.ipsismed.org/resource/resmgr/factfinder/FactFinder_2017-06-01_System.pdf. Last accessed 11/14/2024
Epidural Steroid Injections and Hyperglycemia FactFinder. (2015, April). Retrieved from https://cdn.ymaws.com/www.ipsismed.org/resource/resmgr/factfinder/ESI_and_Hyperglycemia_FF_4-1.pdf. Last accessed 11/14/2024.
Kerezoudis P, Rinaldo L, Alvi MA, Hunt CL, Qu W, Maus TP, Bydon M. The Effect of Epidural Steroid Injections on Bone Mineral Density and Vertebral Fracture Risk: A Systematic Review and Critical Appraisal of Current Literature. Pain Med. 2018 Mar 1;19(3):569-579. doi: 10.1093/pm/pnx324. PMID: 29304236.
Bogduk N. Practice Guidelines for Spinal Diagnostic and Treatment Procedures. 2nd ed. San Francisco; 2013.
Statement on ​Continuum of Depth of Sedation: Definition of General Anesthesia and Levels of Sedation/Analgesia. 2019. https://www.asahq.org/standards-and-practice-parameters/statement-on-continuum-of-depth-of-sedation-definition-of-general-anesthesia-and-levels-of-sedation-analgesia. Last accessed 11/14/2024.
Wilson. U-M Health Policy for the use of Moderate Sedation Analgesia for Diagnostic, Therapeutic and Minor Surgical Procedures by non-Anesthesiologists, 62-11-001. Last accessed 11/14/2024.
McCormick. Routine Intravenous Access for Epidural Steroid Injections Without Sedation. https://cdn.ymaws.com/www.ipsismed.org/resource/resmgr/factfinder/FactFinder_IV_Placement.pdf. Last accessed 11/14/2024.
