# Supplementary material for: Resident Interventional Spine Course with Didactics and Hands-On Skills Lab
Source: MedEdPORTAL. 2025 Oct 7;21:11551. doi: 10.15766/mep_2374-8265.11551 (PMC12502988; doi:10.15766/mep_2374-8265.11551)
Supplement: Supplementary file 1 — Overview - Spine.pptxPrep Kit Materials.docxBuilding a Low-Cost Spine Simulator.pptxFacilitators Guide.docxSpine Procedure - Guidelines Lecture.pptxSpine Procedure Guidelines Lecture Video.mp4Course Chart Review Guidelines.docxSpine Course - Cases.pptxChart Review Preprocedures Checklist.docxInformed Consent and Procedure Timeout Checklist.docxLumbar Procedure Table Checklist.docxProcedure Descriptions.docxFluoroscopic Spine Procedure Images.pptxSpine Course Pre-Post Survey - Updated.docxSpine Course Pre-Post Survey - Original.docx [file mep_2374-8265.11551-s001.zip › G. Course Chart Review Guidelines.docx]

**Interventional Spine Course: Chart Review, Procedure Guidelines, and Complications - Text Overview**

This presentation discusses the risks and guideline recommendations for interventional spine procedures. Potential risks may be related to a patient’s anatomy and current medications, specific aspects of the procedure, or even the experience level of the provider. Reviewing the patient charts ahead of procedure can help to identify potential risks and minimize procedure cancellations. This presentation will have four categories of potential risk using the acronym B.I.A.S. for Bleeding, Infection, Allergy, and Sedation. Ultimately, clinical decisions should be based on individual patient risk factors on a case-by-case basis. National guidelines and department policies are subject to change over time.

Part 1 – Bleeding: *Not all bleeding can be stopped*

An epidural hematoma is the primary bleeding concern with spine procedures as it is difficult to stop bleeding within the spinal column. There are potential bleeding risks associated with continuing anticoagulation medication during elective spine procedures, but this must be weighed against the risk of holding these important medications and putting the patient in potential danger of clotting with subsequent stroke, myocardial infarction, or pulmonary embolism. The 2015 and 2018 ASRA guidelines for anticoagulation management combine both patient and procedure risk factors in their recommendations. Patient factors include age, diabetes, clotting history, hypercoagulable states, and liver or renal disease. Procedure factors may vary as some procedures have closer proximity to vascular structure (stellate ganglion blocks), while a bleed from other procedures could result in catastrophic nerve injury (interlaminar epidural steroid injections). It is highly recommended that interventional spine providers familiarize themselves with the ASRA anticoagulation guidelines.

Somewhat related to bleeding is the topic of particulate versus non-particulate steroid selection. The concern arises with the potential for the inadvertant injection of a particulate steroid directly into the artery of Adamkiewicz, which could cause clotting in the anterior spinal artery leading to anterior cord syndrome. When performing transforaminal epidural steroid injections (TFESI) in the thoracic or lumbar spine it is important to have clear fluoroscopic imaging of the midline of the spinal column to identify the vascular spread of contrast in the hairpin turn of the artery of Adamkiewicz. While the artery of Adamkiewicz most commonly rises at T10 on the left, it can exist anywhere from T7 to L4 on the right as well. The use of a non-particulate steroid, such as dexamethasone, is recommended as the first line of steroid selection for TFESI’s (or other procedures closer to vasculature) due to its lower risk of triggering clot formation. Methylprednisolone or triamcinolone could be considered as a second line if the patient has not responded well to dexamethasone and there were no indications of vascular structures in the area with prior procedures.

Part 2 – Infection: *There is nothing so bad that it can’t be made worse*

The primary infection concerns following an interventional spine procedure are the potential for vertebral osteomyelitis, discitis, or an epidural abscess. It is important to recognize patient risk factors for infection, including diabetes, immune-compromised patients, and recent spinal procedures. Following a procedure, providers should be aware of clinical features that should raise suspicion for a potential infection, including focal neurologic changes and systemic infection symptoms. If suspected, providers should obtain urgent labs and perform a neurologic examination. MRI imaging with and without contrast should be complete within 2 hours if the neurologic exam is abnormal; within 6 hours if the neurologic exam is normal.

Additional measures to reduce potential infection risks include hand washing with antimicrobial soap, use of chlorhexidine as the preferred skin antiseptic, and meticulous sterile technique.

Part 3 – Allergy (Adverse Reactions) *Are you blushing?*

During chart review, providers must become aware of a patient’s known allergic reactions and potential risks posed by the medications, substances, and materials used during the procedure. Providers should be able to identify signs of anaphylaxis, particularly skin and respiratory symptoms, before they progress to more severe systemic, cardiac, and neurological symptoms.

There is an emerging concern about the use of gadolinium-based contrasts, particularly in relation to unintentional subdural injection. There have been rare cases of encephalitis and gadolinium retention within the central nervous system, the consequences of which remain unknown. It has been recommended that gadolinium-based contrast is not used for epidural steroid injections, especially interlaminar approaches. For patients with iodine allergies, the current recommendation is the use of pre-medication with steroids and diphenhydramine before using iodine-based contrast.

While there are potential risks of local anesthetic systemic toxicity (LAST), the low volumes used, and location of most spine procedures makes LAST rather unlikely. It remains the recommendation to use the lowest dose of local anesthetic for the desired effect.

The amount of steroid exposure must also be considered in the setting of patient-specific factors. Patients with diabetes should monitor fasting blood glucose for at least a week after any steroid injection and communicate with their primary care doctor or endocrinologist if needed. Additionally, steroids should be used with caution for patients at risk of developing osteoporosis.

4. Sedation – *Are you still there?*

Interventional spine physicians should balance the risks and benefits when considering sedation for elective spine procedures. There are patient-specific factors that should be considered before ordering sedation, during the pre-sedation planning, and while monitoring the patient pre-/post-procedure. The goal for mild to moderate sedation for spine procedures is to have a cooperative and relatively still patient. Deep sedation is generally not recommended for most spine procedures as the last line of defense before a potential injury is the patient being able to defend themselves by saying the word “ouch”.

For patients with a history of vasovagal syncope, IV sedation may be helpful, but it doesn't guarantee this will prevent similar vasovagal episodes.

In conclusion, all interventional spine physicians should remain up to date with the ever-changing guidelines and recommendations from their local departments and national organizations such as ASRA, IPSIS, and NASS.
