# Supplementary material for: Resident Interventional Spine Course with Didactics and Hands-On Skills Lab
Source: MedEdPORTAL. 2025 Oct 7;21:11551. doi: 10.15766/mep_2374-8265.11551 (PMC12502988; doi:10.15766/mep_2374-8265.11551)
Supplement: Supplementary file 1 — Overview - Spine.pptxPrep Kit Materials.docxBuilding a Low-Cost Spine Simulator.pptxFacilitators Guide.docxSpine Procedure - Guidelines Lecture.pptxSpine Procedure Guidelines Lecture Video.mp4Course Chart Review Guidelines.docxSpine Course - Cases.pptxChart Review Preprocedures Checklist.docxInformed Consent and Procedure Timeout Checklist.docxLumbar Procedure Table Checklist.docxProcedure Descriptions.docxFluoroscopic Spine Procedure Images.pptxSpine Course Pre-Post Survey - Updated.docxSpine Course Pre-Post Survey - Original.docx [file mep_2374-8265.11551-s001.zip › H. Spine Course - Cases.pptx]

## Slide 1
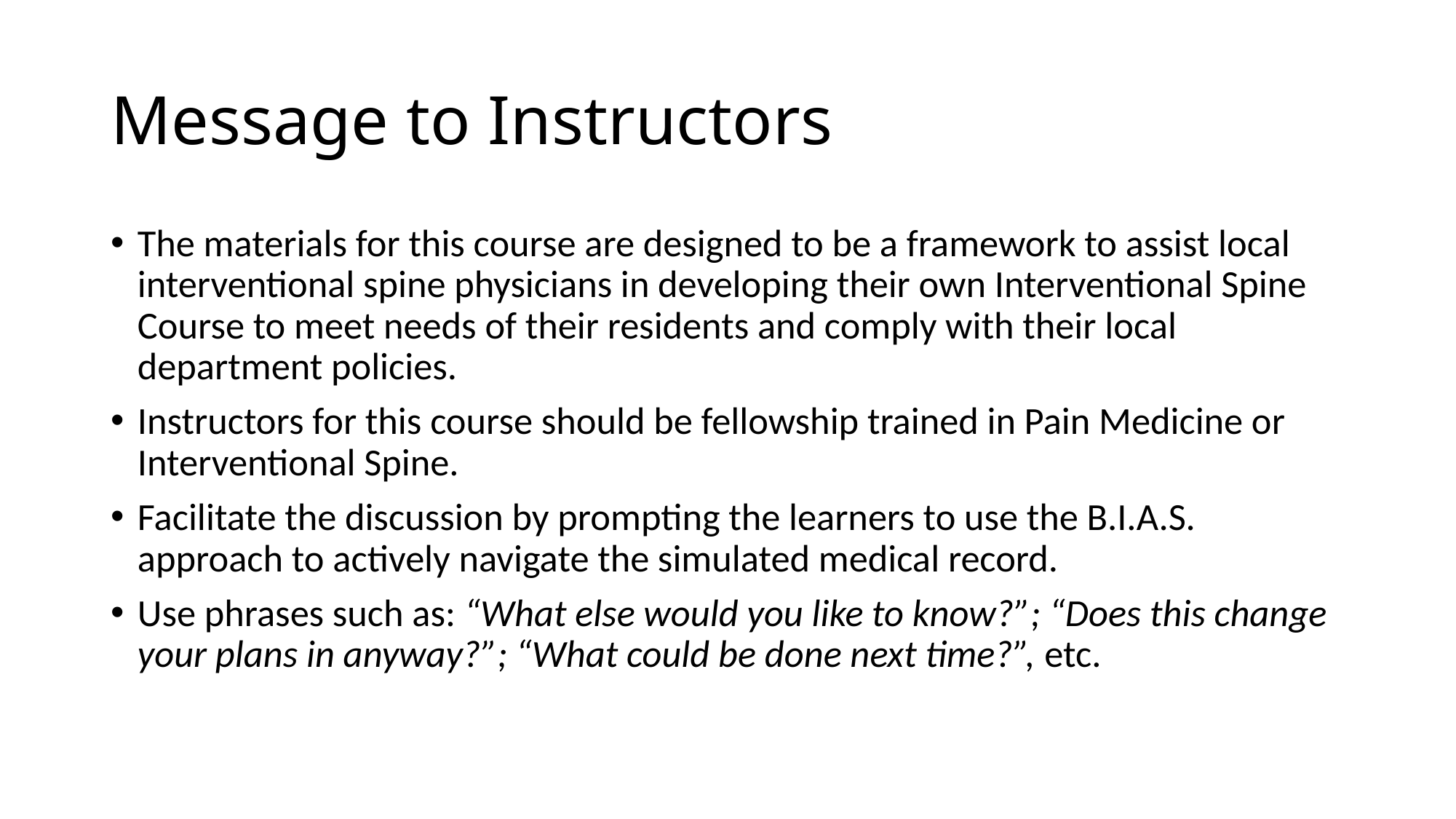

# Message to Instructors
The materials for this course are designed to be a framework to assist local interventional spine physicians in developing their own Interventional Spine Course to meet needs of their residents and comply with their local department policies.
Instructors for this course should be fellowship trained in Pain Medicine or Interventional Spine.
Facilitate the discussion by prompting the learners to use the B.I.A.S. approach to actively navigate the simulated medical record.
Use phrases such as: “What else would you like to know?”; “Does this change your plans in anyway?”; “What could be done next time?”, etc.

## Slide 2
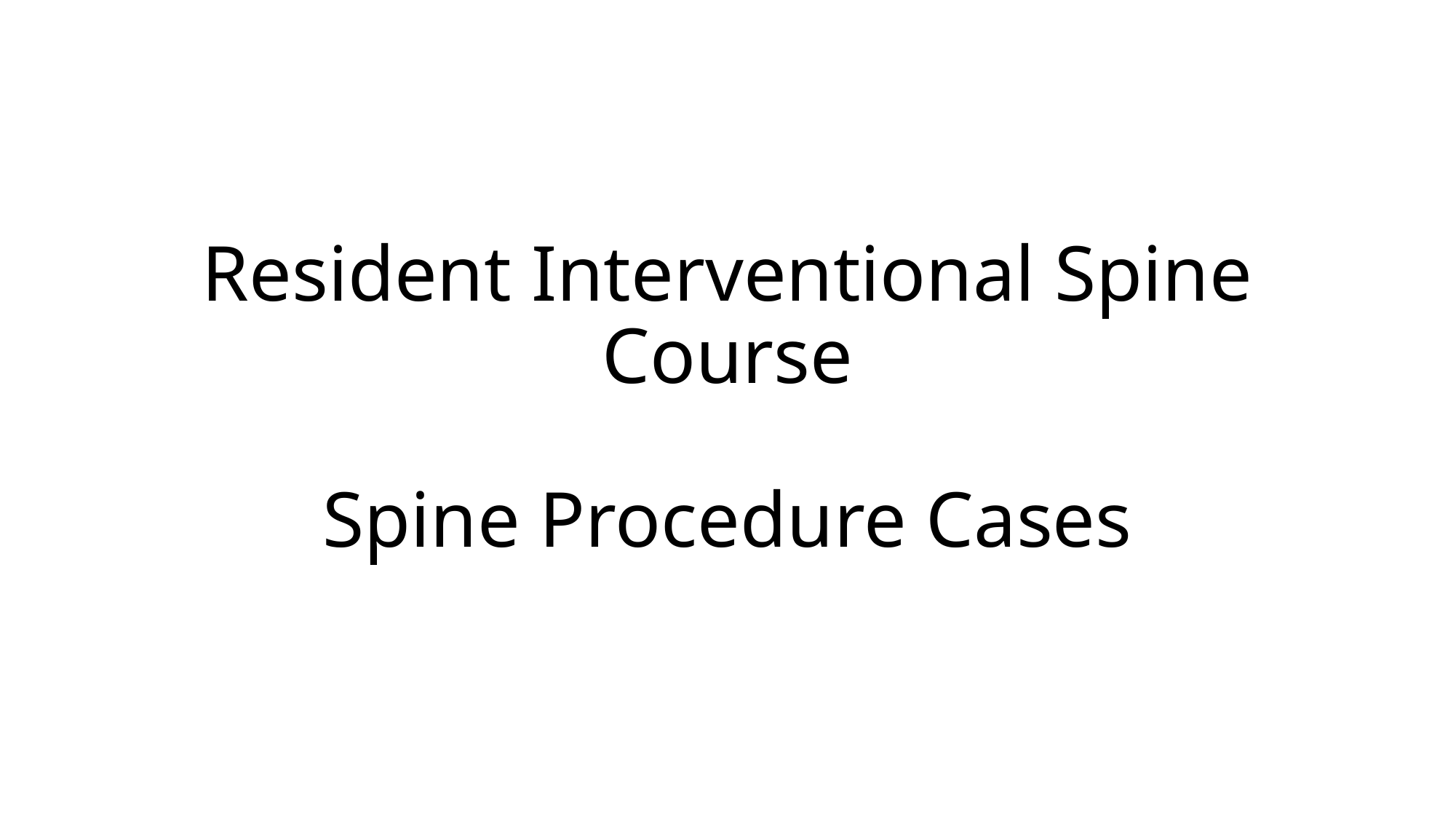

# Resident Interventional Spine Course Spine Procedure Cases

## Slide 3
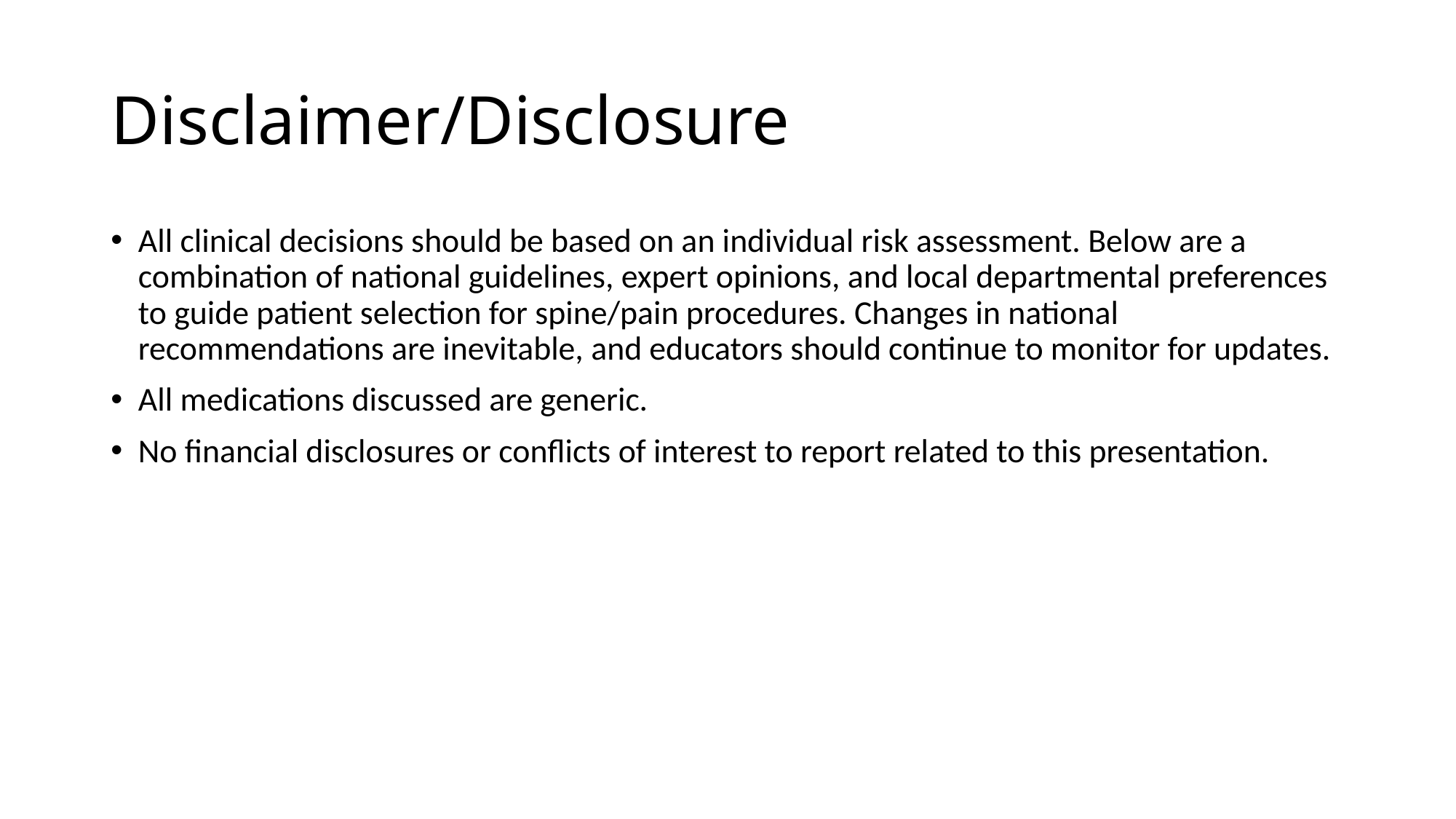

# Disclaimer/Disclosure
All clinical decisions should be based on an individual risk assessment. Below are a combination of national guidelines, expert opinions, and local departmental preferences to guide patient selection for spine/pain procedures. Changes in national recommendations are inevitable, and educators should continue to monitor for updates.
All medications discussed are generic.
No financial disclosures or conflicts of interest to report related to this presentation.

## Slide 4
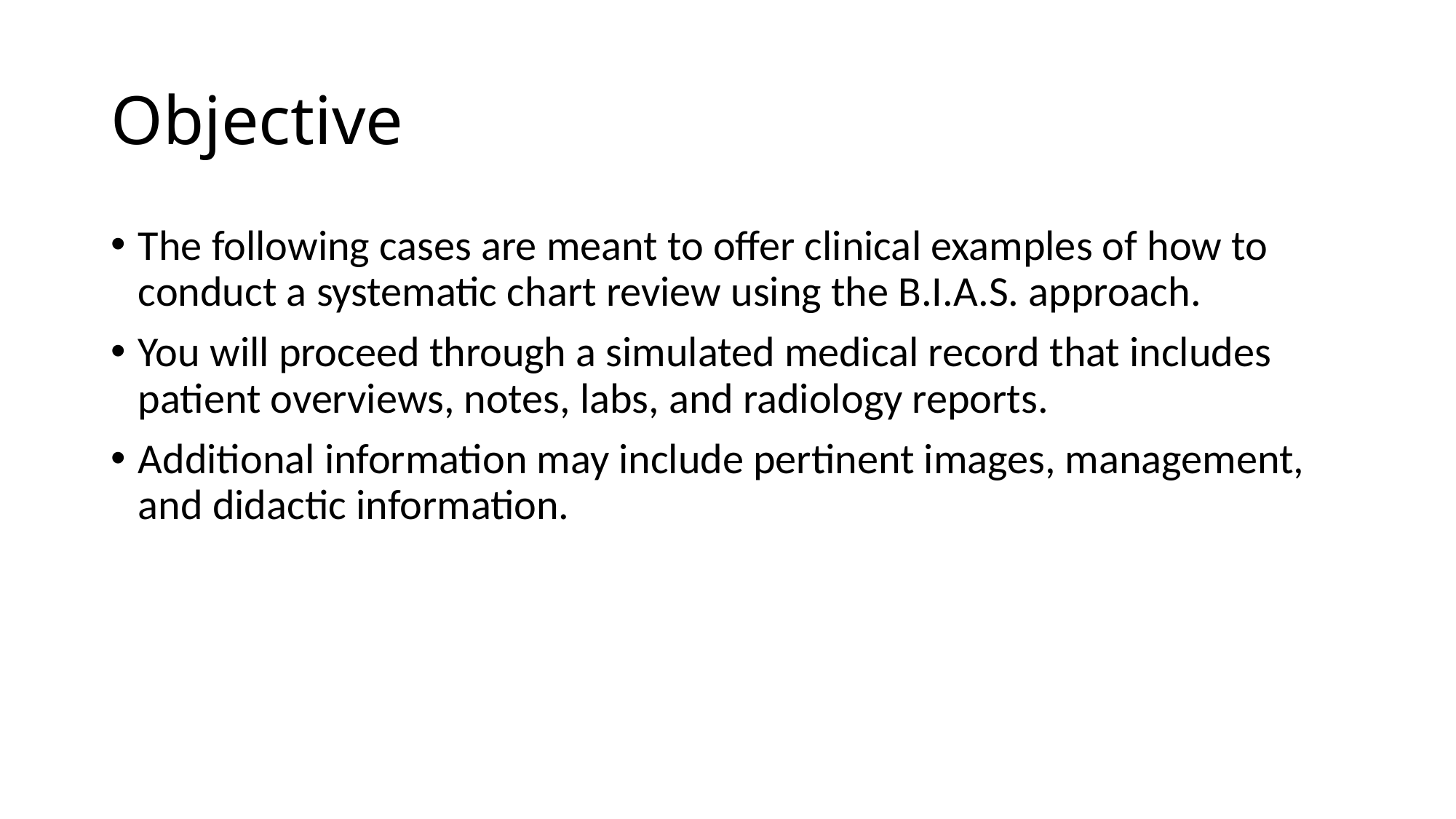

# Objective
The following cases are meant to offer clinical examples of how to conduct a systematic chart review using the B.I.A.S. approach.
You will proceed through a simulated medical record that includes patient overviews, notes, labs, and radiology reports.
Additional information may include pertinent images, management, and didactic information.

## Slide 5
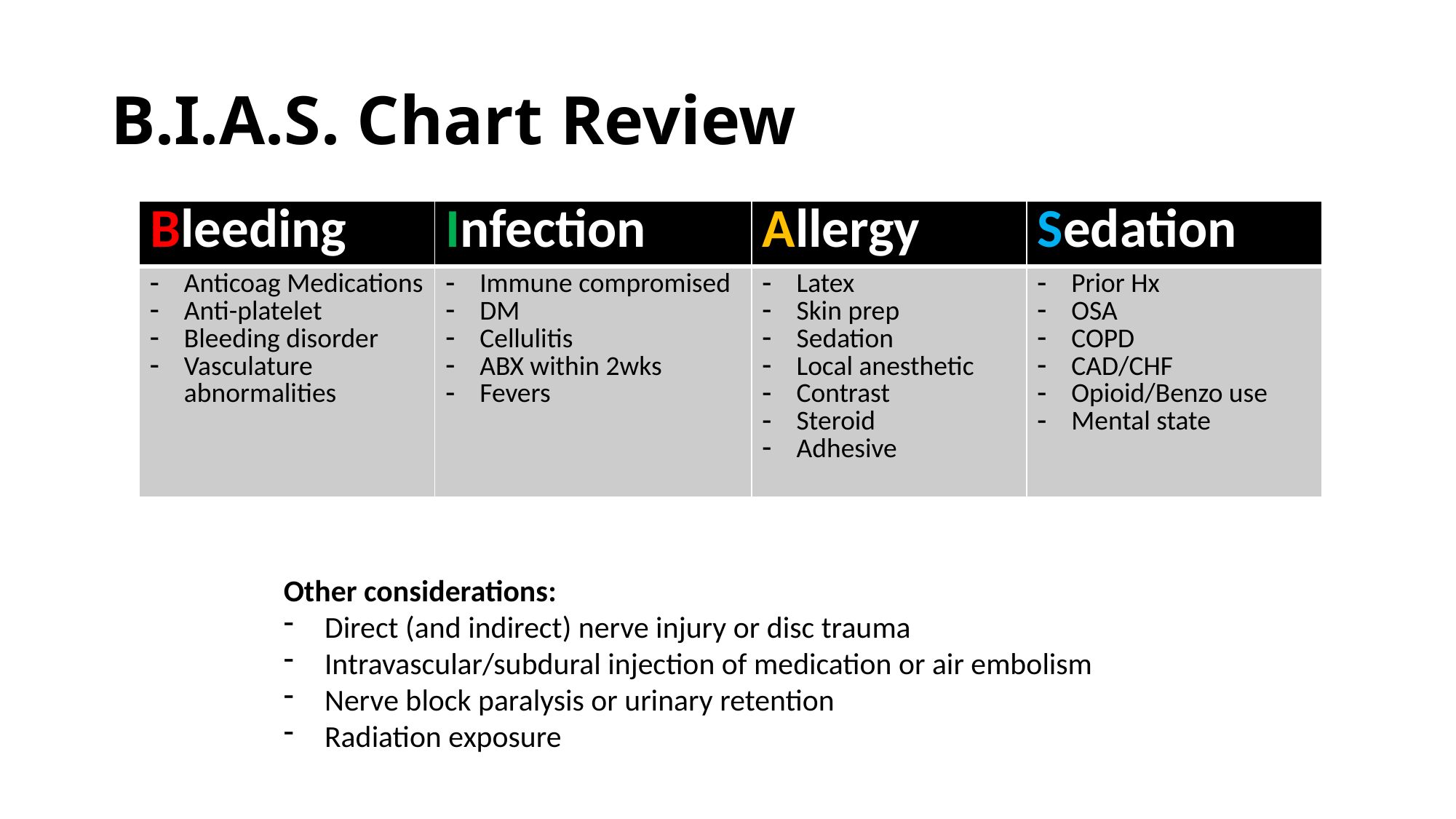

# B.I.A.S. Chart Review
| Bleeding | Infection | Allergy | Sedation |
| --- | --- | --- | --- |
| Anticoag Medications Anti-platelet Bleeding disorder Vasculature abnormalities | Immune compromised DM Cellulitis ABX within 2wks Fevers | Latex Skin prep Sedation Local anesthetic Contrast Steroid Adhesive | Prior Hx OSA COPD CAD/CHF Opioid/Benzo use Mental state |
Other considerations:
Direct (and indirect) nerve injury or disc trauma
Intravascular/subdural injection of medication or air embolism
Nerve block paralysis or urinary retention
Radiation exposure

## Slide 6
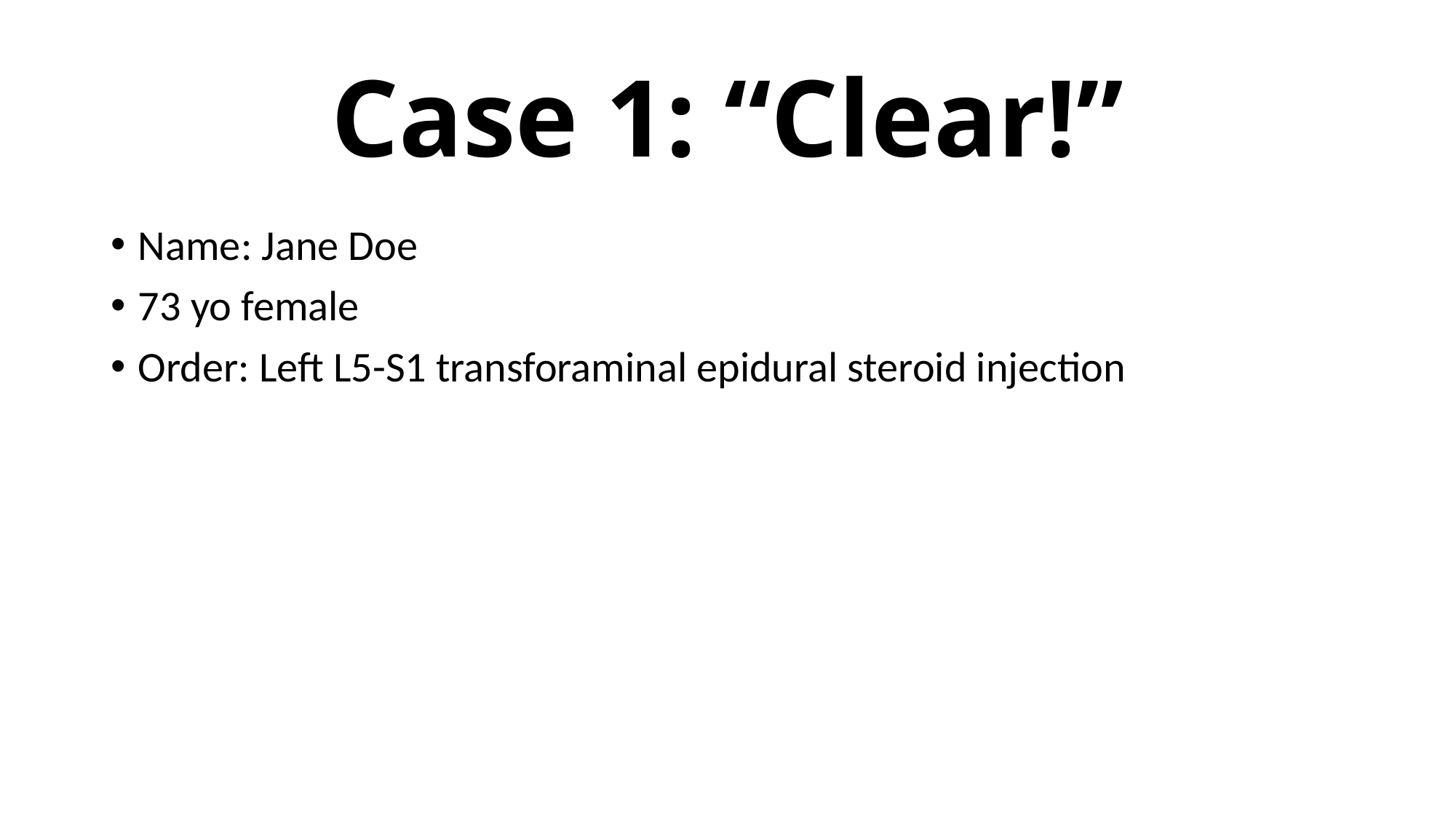

# Case 1: “Clear!”
Name: Jane Doe
73 yo female
Order: Left L5-S1 transforaminal epidural steroid injection

## Slide 7
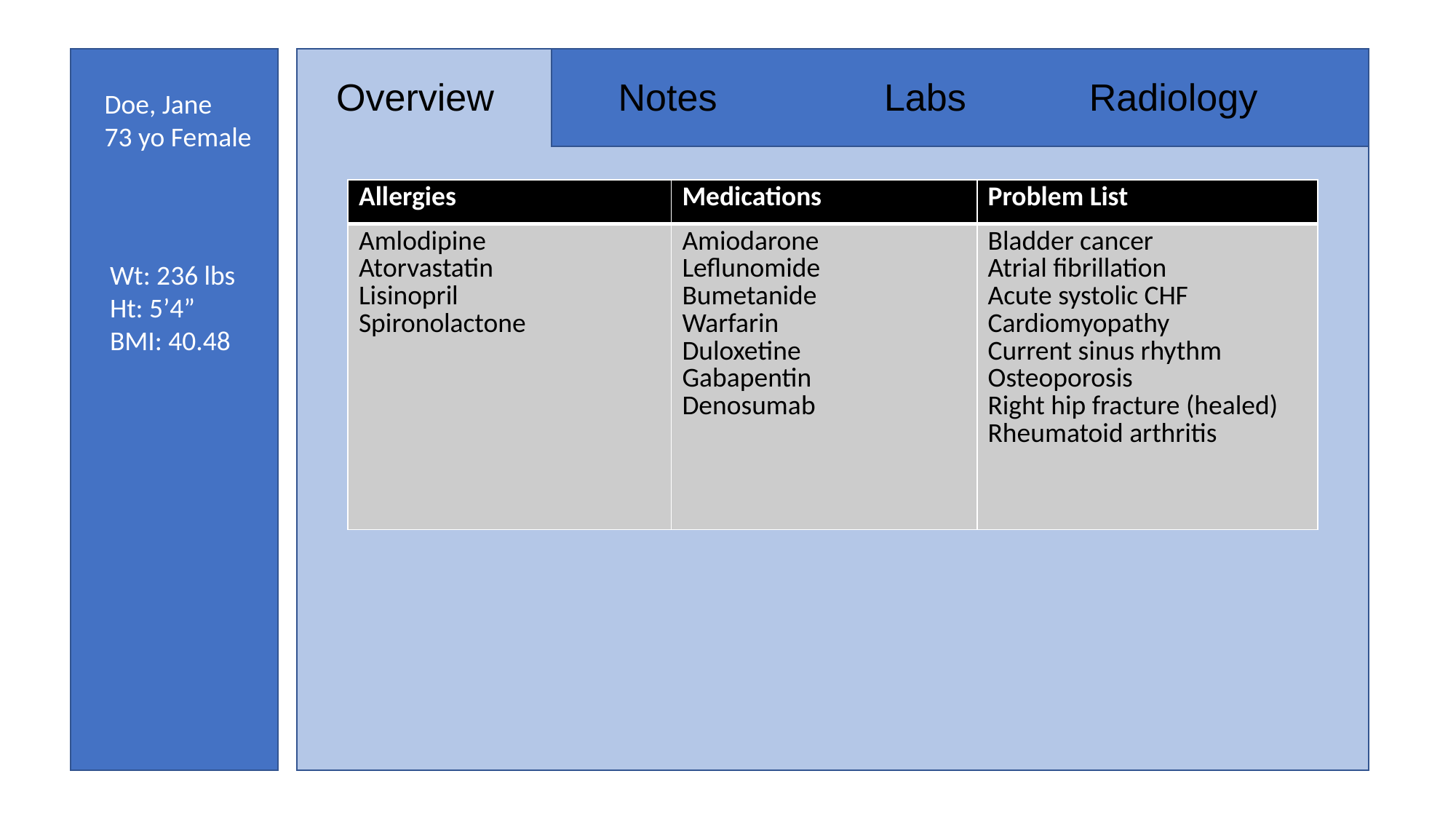

Overview
Notes
Labs
Radiology
Doe, Jane
73 yo Female
| Allergies | Medications | Problem List |
| --- | --- | --- |
| Amlodipine Atorvastatin Lisinopril Spironolactone | Amiodarone Leflunomide  Bumetanide  Warfarin Duloxetine Gabapentin Denosumab | Bladder cancer Atrial fibrillation Acute systolic CHF Cardiomyopathy Current sinus rhythm Osteoporosis Right hip fracture (healed) Rheumatoid arthritis |
Wt: 236 lbs
Ht: 5’4”
BMI: 40.48

## Slide 8
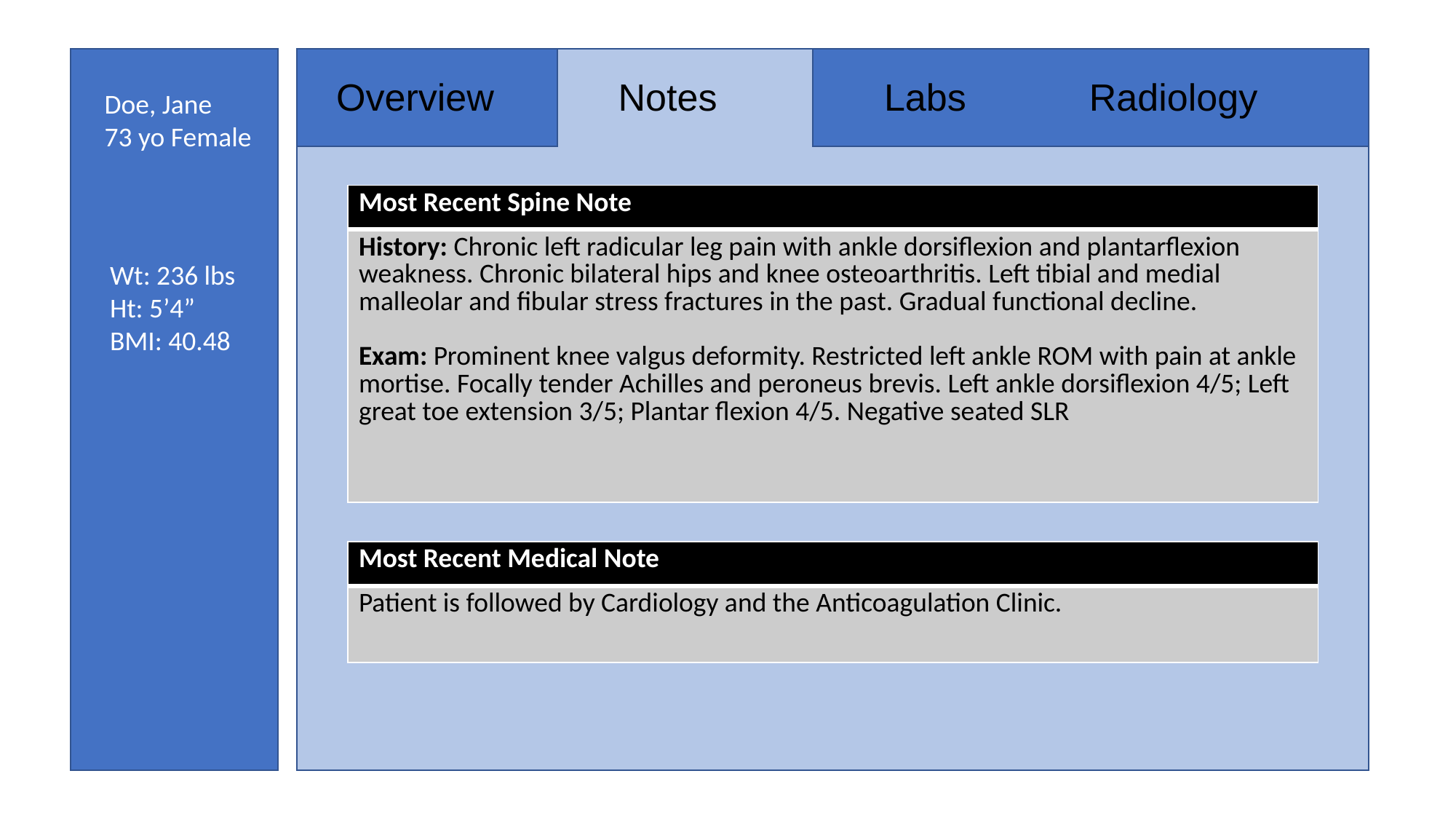

Overview
Notes
Labs
Radiology
Doe, Jane
73 yo Female
| Most Recent Spine Note |
| --- |
| History: Chronic left radicular leg pain with ankle dorsiflexion and plantarflexion weakness. Chronic bilateral hips and knee osteoarthritis. Left tibial and medial malleolar and fibular stress fractures in the past. Gradual functional decline. Exam: Prominent knee valgus deformity. Restricted left ankle ROM with pain at ankle mortise. Focally tender Achilles and peroneus brevis. Left ankle dorsiflexion 4/5; Left great toe extension 3/5; Plantar flexion 4/5. Negative seated SLR |
Wt: 236 lbs
Ht: 5’4”
BMI: 40.48
| Most Recent Medical Note |
| --- |
| Patient is followed by Cardiology and the Anticoagulation Clinic. |

## Slide 9
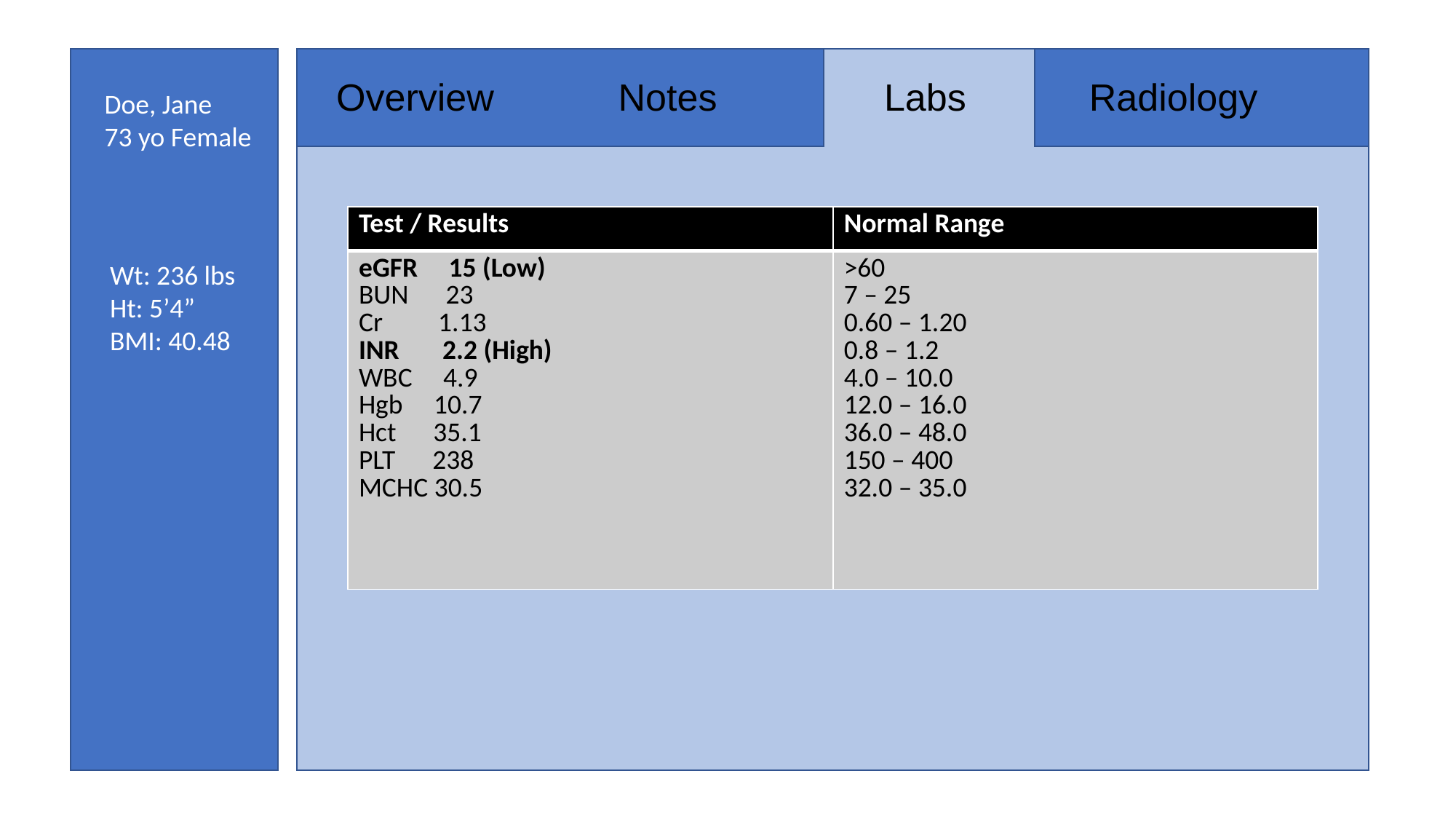

Overview
Notes
Labs
Radiology
Doe, Jane
73 yo Female
| Test / Results | Normal Range |
| --- | --- |
| eGFR 15 (Low) BUN 23 Cr 1.13 INR 2.2 (High) WBC 4.9 Hgb 10.7 Hct 35.1 PLT 238 MCHC 30.5 | >60 7 – 25 0.60 – 1.20 0.8 – 1.2 4.0 – 10.0 12.0 – 16.0 36.0 – 48.0 150 – 400 32.0 – 35.0 |
Wt: 236 lbs
Ht: 5’4”
BMI: 40.48

## Slide 10
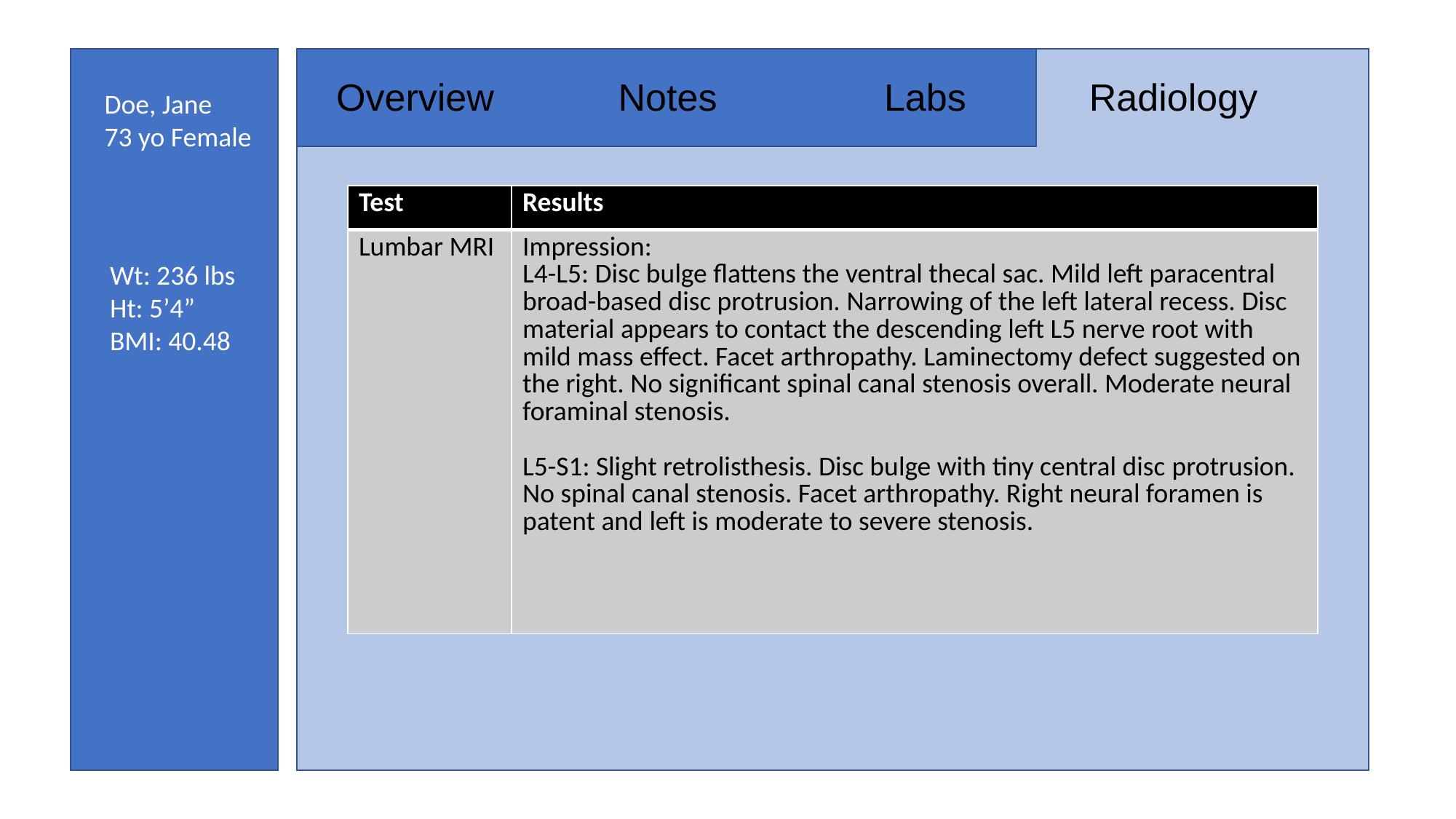

Overview
Notes
Labs
Radiology
Doe, Jane
73 yo Female
| Test | Results |
| --- | --- |
| Lumbar MRI | Impression: L4-L5: Disc bulge flattens the ventral thecal sac. Mild left paracentral broad-based disc protrusion. Narrowing of the left lateral recess. Disc material appears to contact the descending left L5 nerve root with mild mass effect. Facet arthropathy. Laminectomy defect suggested on the right. No significant spinal canal stenosis overall. Moderate neural foraminal stenosis. L5-S1: Slight retrolisthesis. Disc bulge with tiny central disc protrusion. No spinal canal stenosis. Facet arthropathy. Right neural foramen is patent and left is moderate to severe stenosis. |
Wt: 236 lbs
Ht: 5’4”
BMI: 40.48

## Slide 11
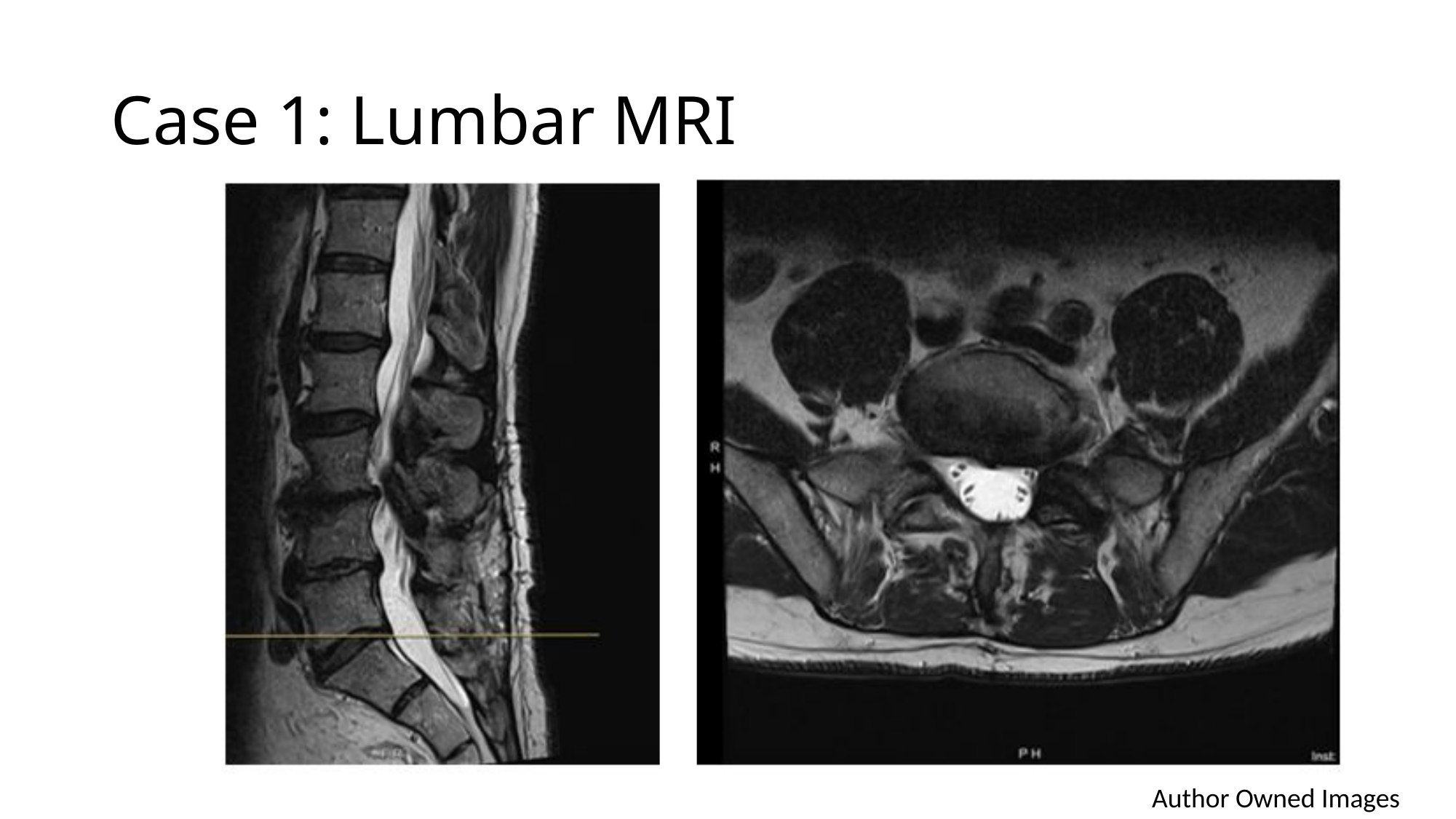

# Case 1: Lumbar MRI
Author Owned Images

## Slide 12
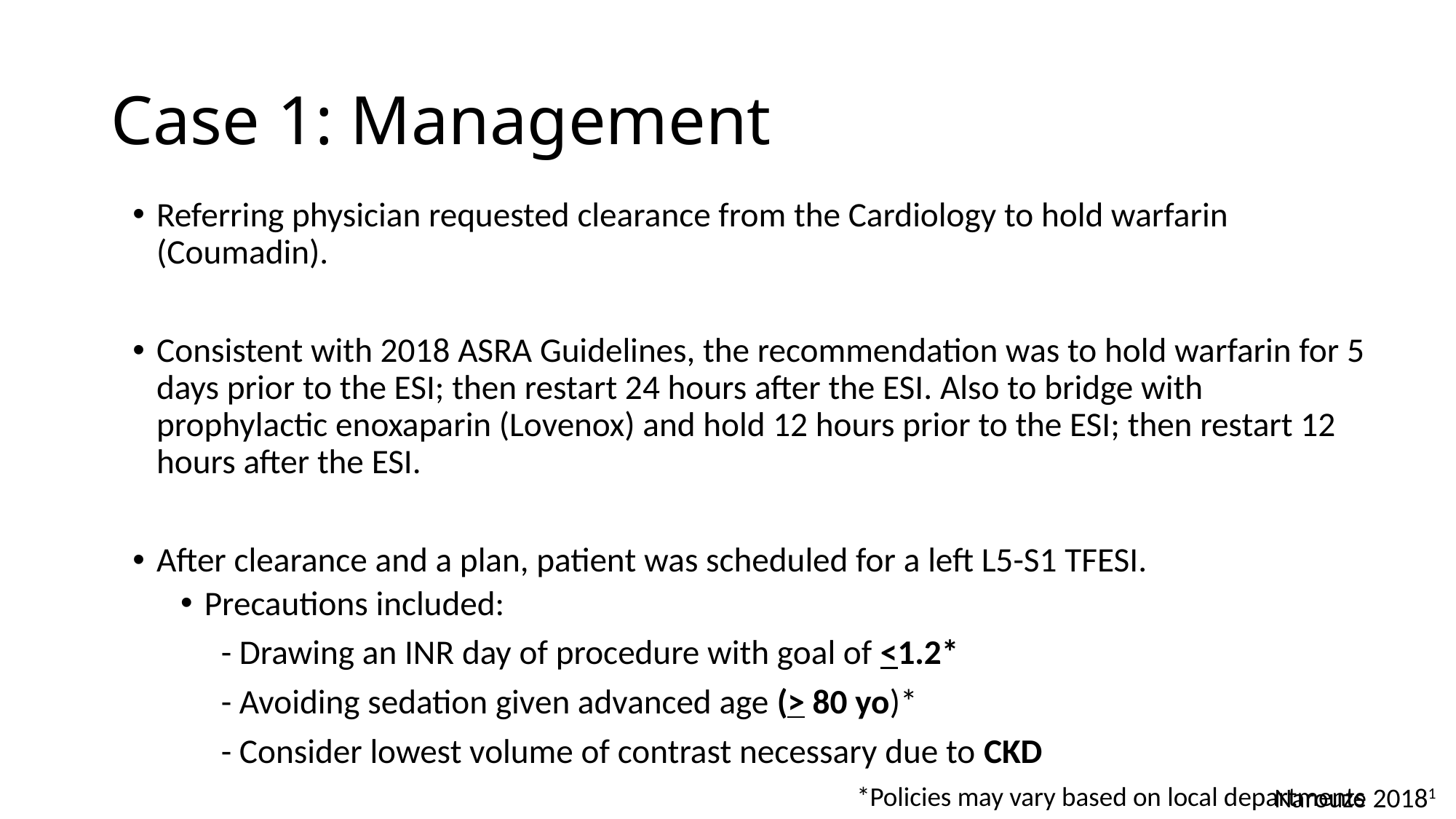

# Case 1: Management
Referring physician requested clearance from the Cardiology to hold warfarin (Coumadin).
Consistent with 2018 ASRA Guidelines, the recommendation was to hold warfarin for 5 days prior to the ESI; then restart 24 hours after the ESI. Also to bridge with prophylactic enoxaparin (Lovenox) and hold 12 hours prior to the ESI; then restart 12 hours after the ESI.
After clearance and a plan, patient was scheduled for a left L5-S1 TFESI.
Precautions included:
	- Drawing an INR day of procedure with goal of <1.2*
	- Avoiding sedation given advanced age (> 80 yo)*
	- Consider lowest volume of contrast necessary due to CKD
*Policies may vary based on local departments
Narouze 20181

## Slide 13
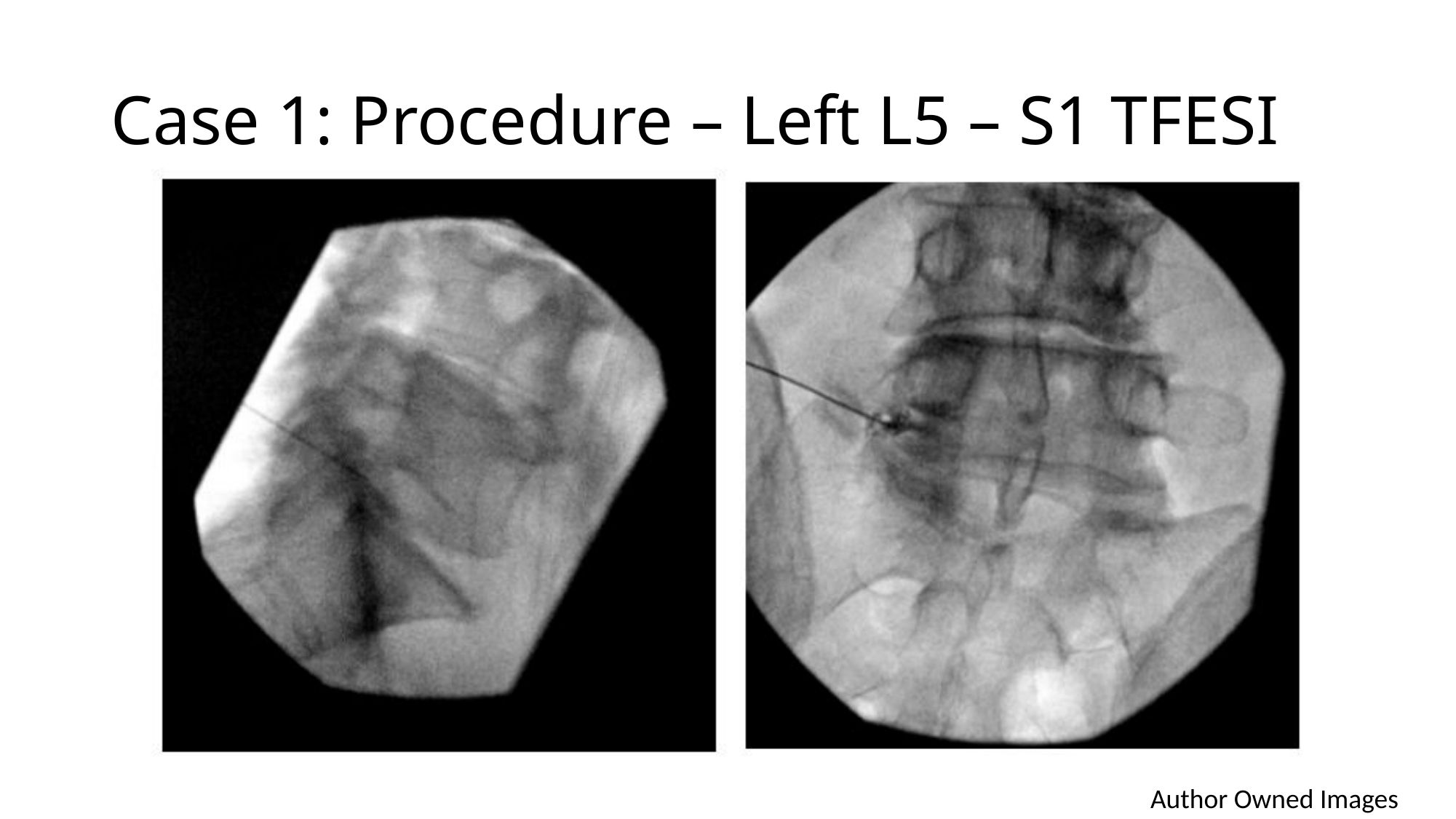

# Case 1: Procedure – Left L5 – S1 TFESI
Author Owned Images

## Slide 14
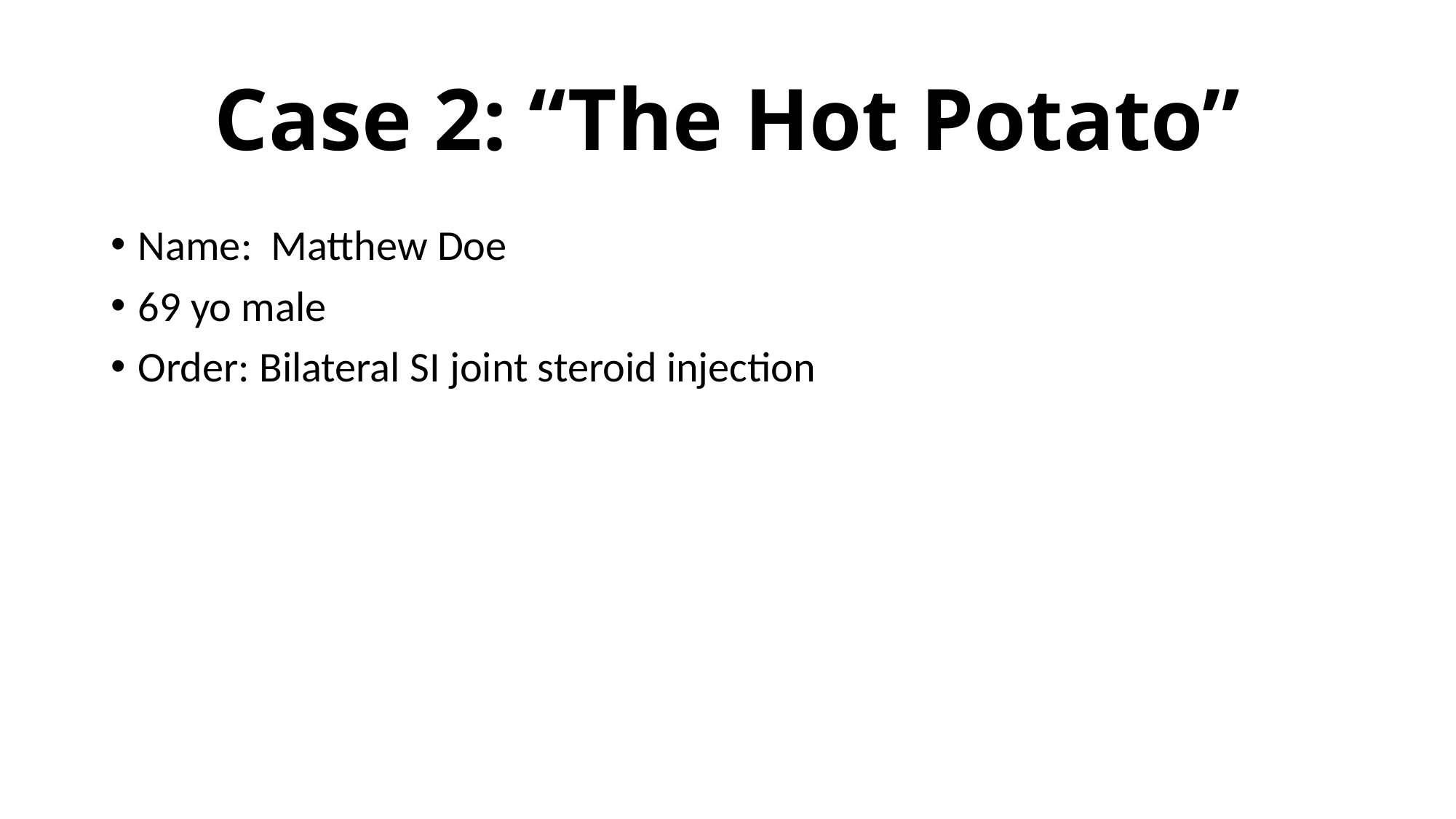

# Case 2: “The Hot Potato”
Name: Matthew Doe
69 yo male
Order: Bilateral SI joint steroid injection

## Slide 15
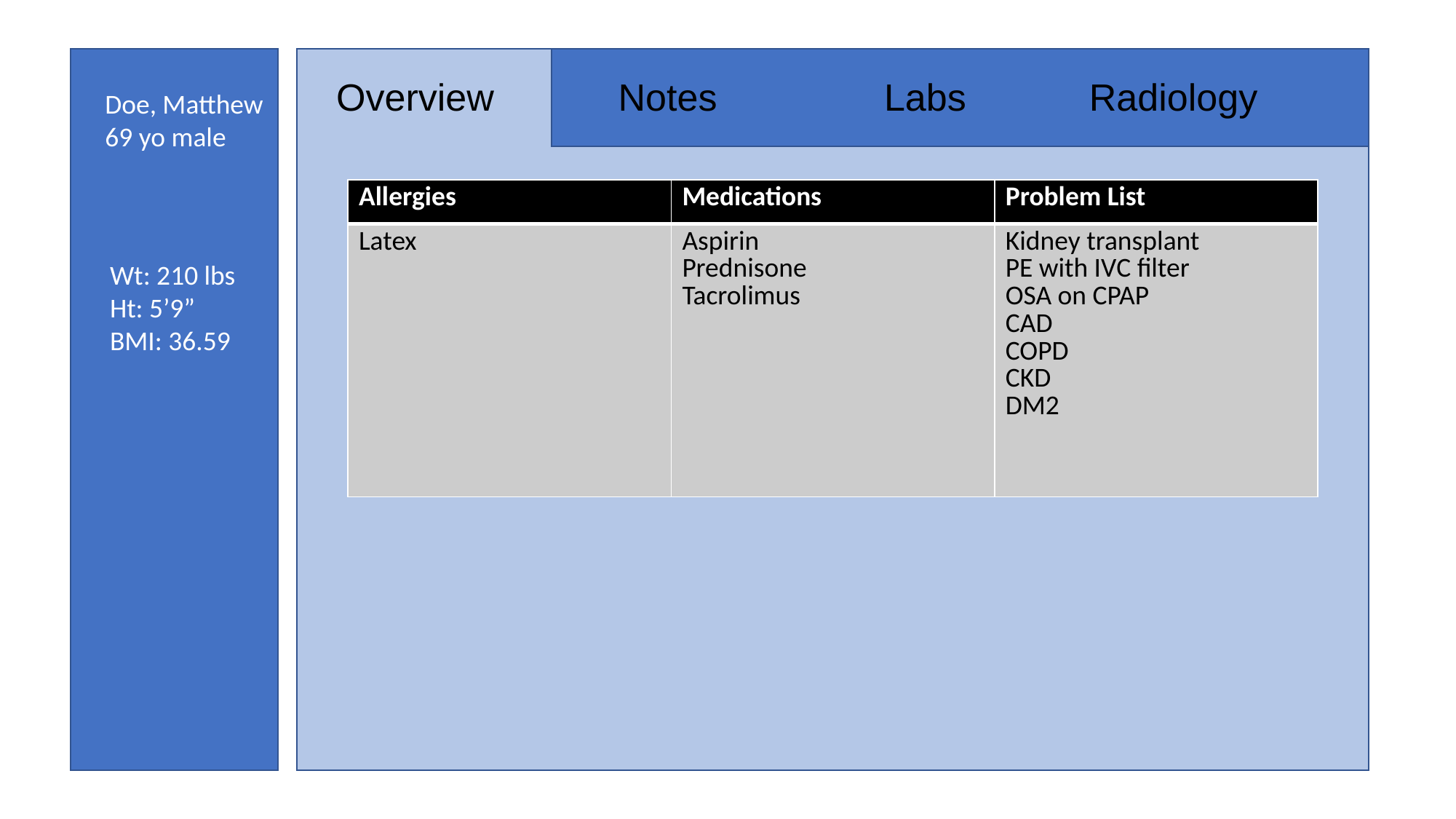

Overview
Notes
Labs
Radiology
Doe, Matthew
69 yo male
| Allergies | Medications | Problem List |
| --- | --- | --- |
| Latex | Aspirin Prednisone Tacrolimus | Kidney transplant PE with IVC filter OSA on CPAP CAD COPD CKD DM2 |
Wt: 210 lbs
Ht: 5’9”
BMI: 36.59

## Slide 16
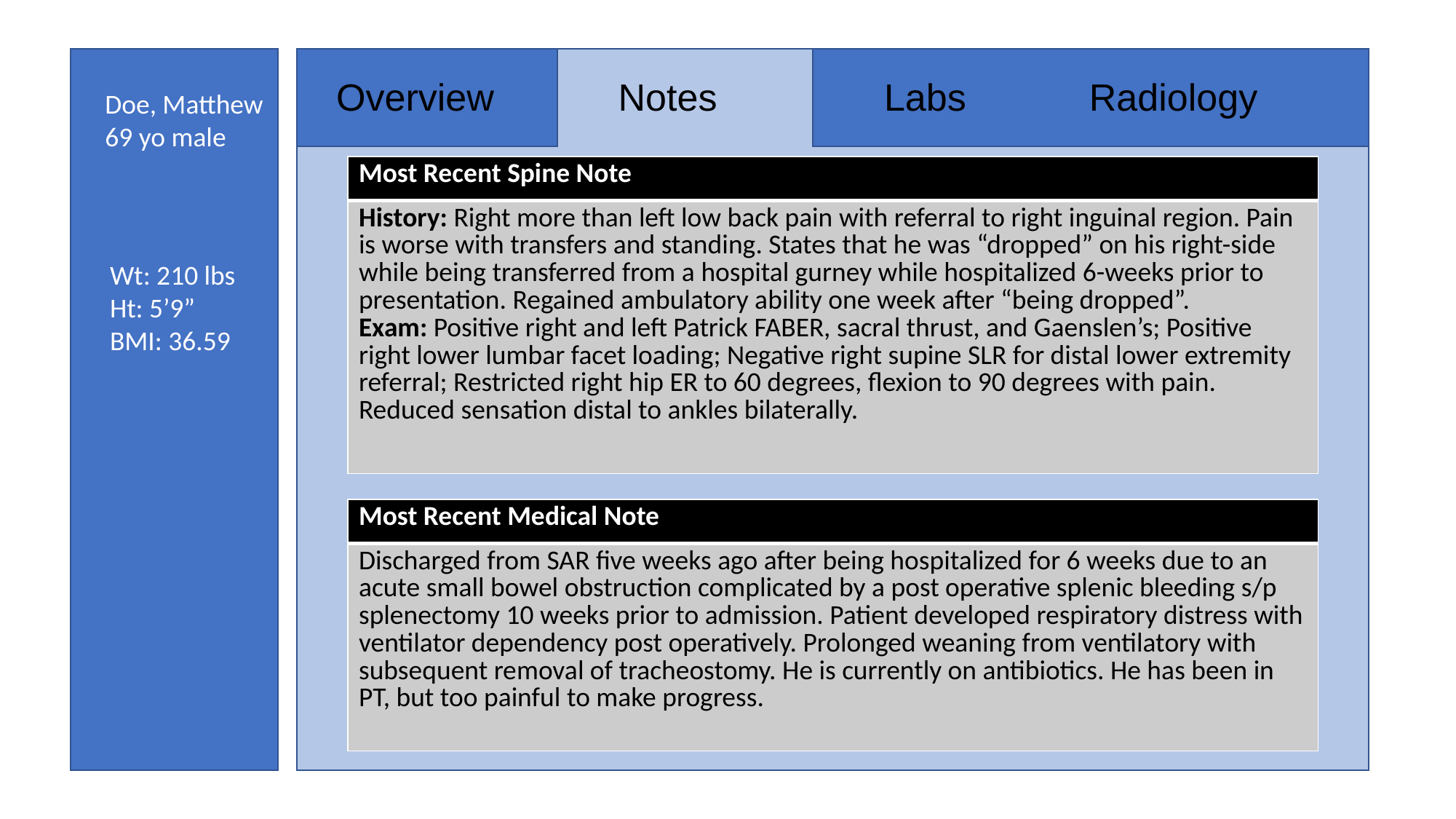

Overview
Notes
Labs
Radiology
Doe, Matthew
69 yo male
| Most Recent Spine Note |
| --- |
| History: Right more than left low back pain with referral to right inguinal region. Pain is worse with transfers and standing. States that he was “dropped” on his right-side while being transferred from a hospital gurney while hospitalized 6-weeks prior to presentation. Regained ambulatory ability one week after “being dropped”. Exam: Positive right and left Patrick FABER, sacral thrust, and Gaenslen’s; Positive right lower lumbar facet loading; Negative right supine SLR for distal lower extremity referral; Restricted right hip ER to 60 degrees, flexion to 90 degrees with pain. Reduced sensation distal to ankles bilaterally. |
Wt: 210 lbs
Ht: 5’9”
BMI: 36.59
| Most Recent Medical Note |
| --- |
| Discharged from SAR five weeks ago after being hospitalized for 6 weeks due to an acute small bowel obstruction complicated by a post operative splenic bleeding s/p splenectomy 10 weeks prior to admission. Patient developed respiratory distress with ventilator dependency post operatively. Prolonged weaning from ventilatory with subsequent removal of tracheostomy. He is currently on antibiotics. He has been in PT, but too painful to make progress. |

## Slide 17
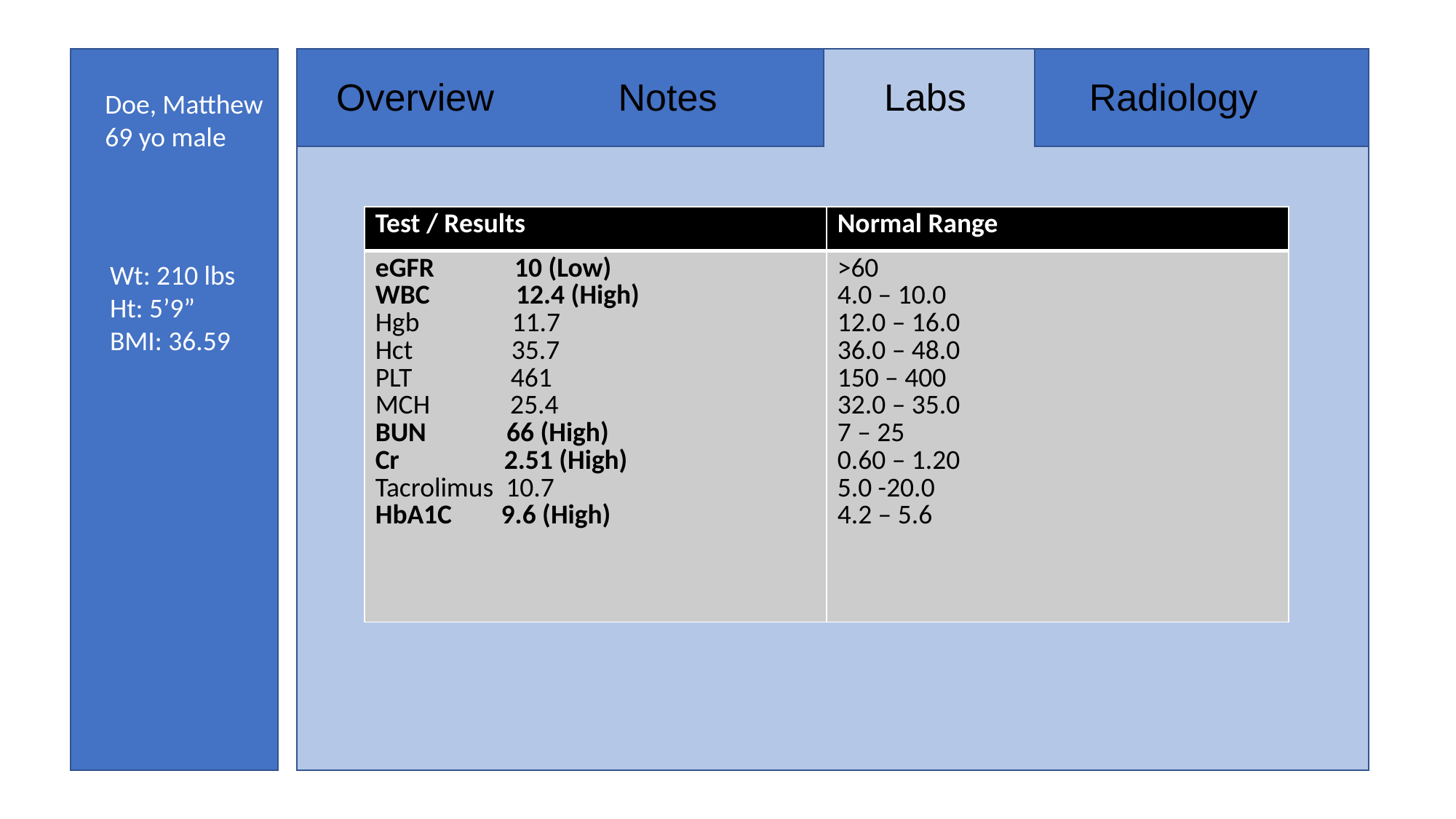

Overview
Notes
Labs
Radiology
Doe, Matthew
69 yo male
| Test / Results | Normal Range |
| --- | --- |
| eGFR 10 (Low) WBC 12.4 (High) Hgb 11.7 Hct 35.7 PLT 461 MCH 25.4 BUN 66 (High) Cr 2.51 (High) Tacrolimus 10.7 HbA1C 9.6 (High) | >60 4.0 – 10.0 12.0 – 16.0 36.0 – 48.0 150 – 400 32.0 – 35.0 7 – 25 0.60 – 1.20 5.0 -20.0 4.2 – 5.6 |
Wt: 210 lbs
Ht: 5’9”
BMI: 36.59

## Slide 18
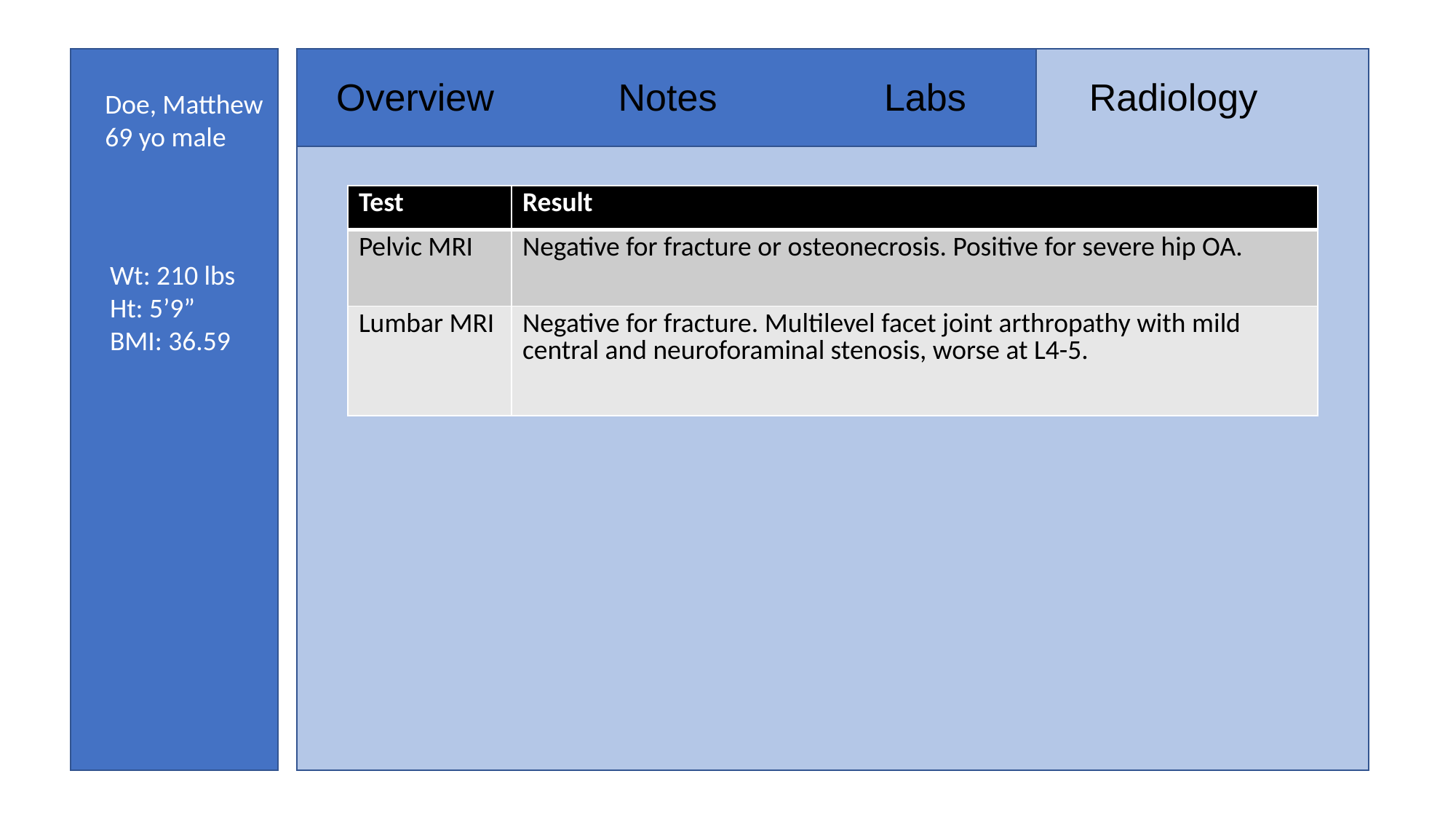

Overview
Notes
Labs
Radiology
Doe, Matthew
69 yo male
| Test | Result |
| --- | --- |
| Pelvic MRI | Negative for fracture or osteonecrosis. Positive for severe hip OA. |
| Lumbar MRI | Negative for fracture. Multilevel facet joint arthropathy with mild central and neuroforaminal stenosis, worse at L4-5. |
Wt: 210 lbs
Ht: 5’9”
BMI: 36.59

## Slide 19
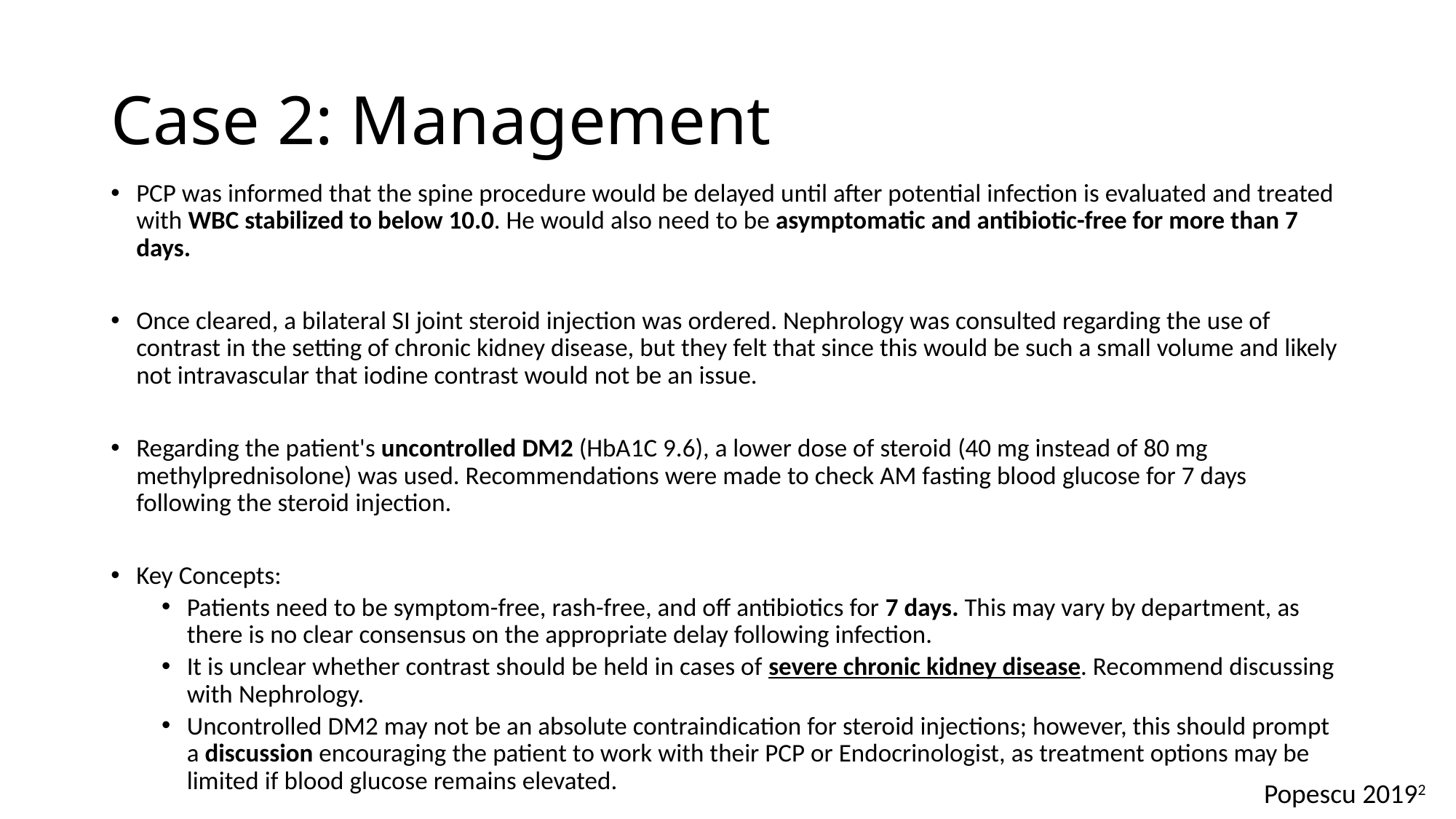

# Case 2: Management
PCP was informed that the spine procedure would be delayed until after potential infection is evaluated and treated with WBC stabilized to below 10.0. He would also need to be asymptomatic and antibiotic-free for more than 7 days.
Once cleared, a bilateral SI joint steroid injection was ordered. Nephrology was consulted regarding the use of contrast in the setting of chronic kidney disease, but they felt that since this would be such a small volume and likely not intravascular that iodine contrast would not be an issue.
Regarding the patient's uncontrolled DM2 (HbA1C 9.6), a lower dose of steroid (40 mg instead of 80 mg methylprednisolone) was used. Recommendations were made to check AM fasting blood glucose for 7 days following the steroid injection.
Key Concepts:
Patients need to be symptom-free, rash-free, and off antibiotics for 7 days. This may vary by department, as there is no clear consensus on the appropriate delay following infection.
It is unclear whether contrast should be held in cases of severe chronic kidney disease. Recommend discussing with Nephrology.
Uncontrolled DM2 may not be an absolute contraindication for steroid injections; however, this should prompt a discussion encouraging the patient to work with their PCP or Endocrinologist, as treatment options may be limited if blood glucose remains elevated.
Popescu 20192

## Slide 20
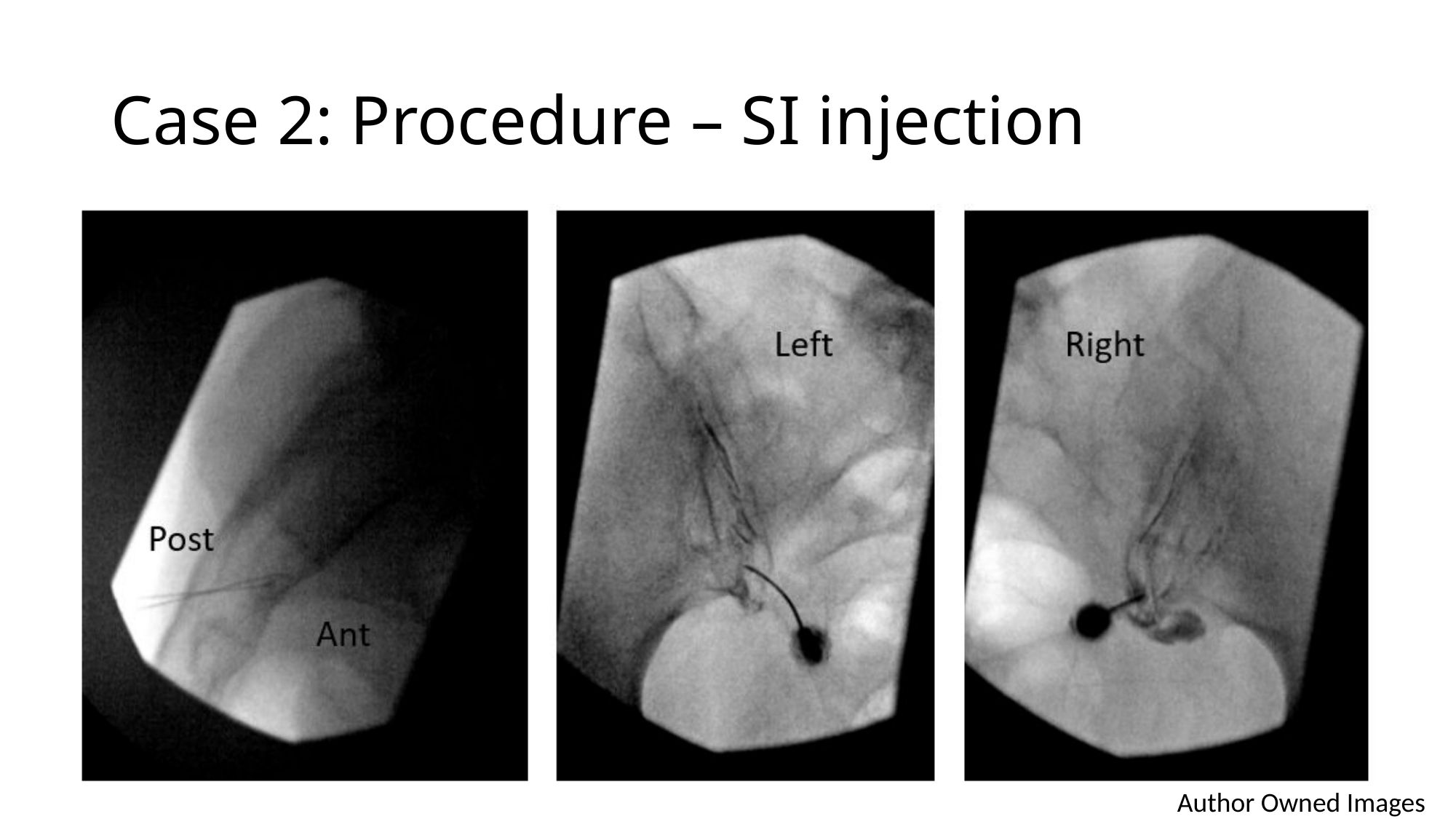

# Case 2: Procedure – SI injection
Author Owned Images

## Slide 21
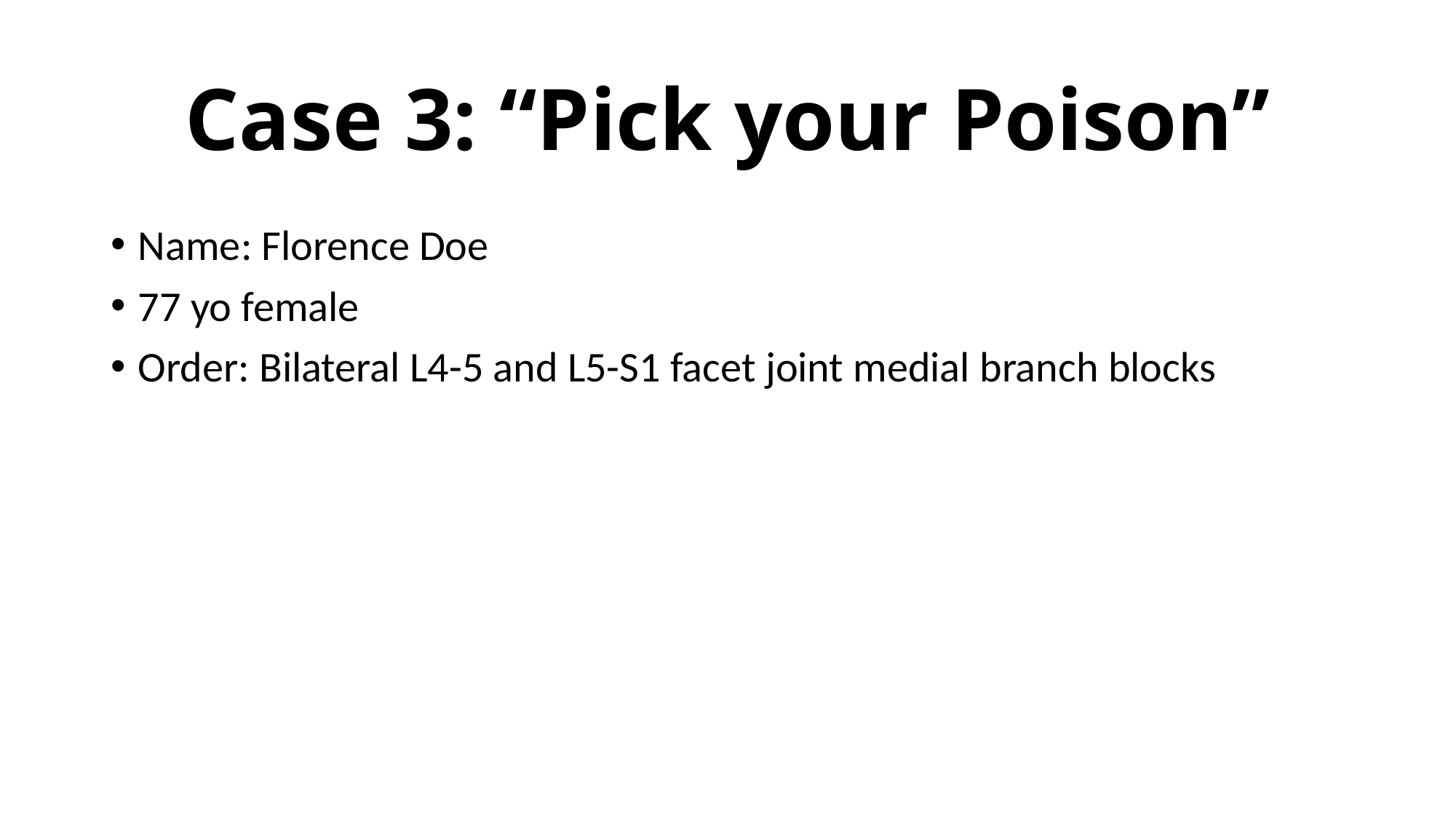

# Case 3: “Pick your Poison”
Name: Florence Doe
77 yo female
Order: Bilateral L4-5 and L5-S1 facet joint medial branch blocks

## Slide 22
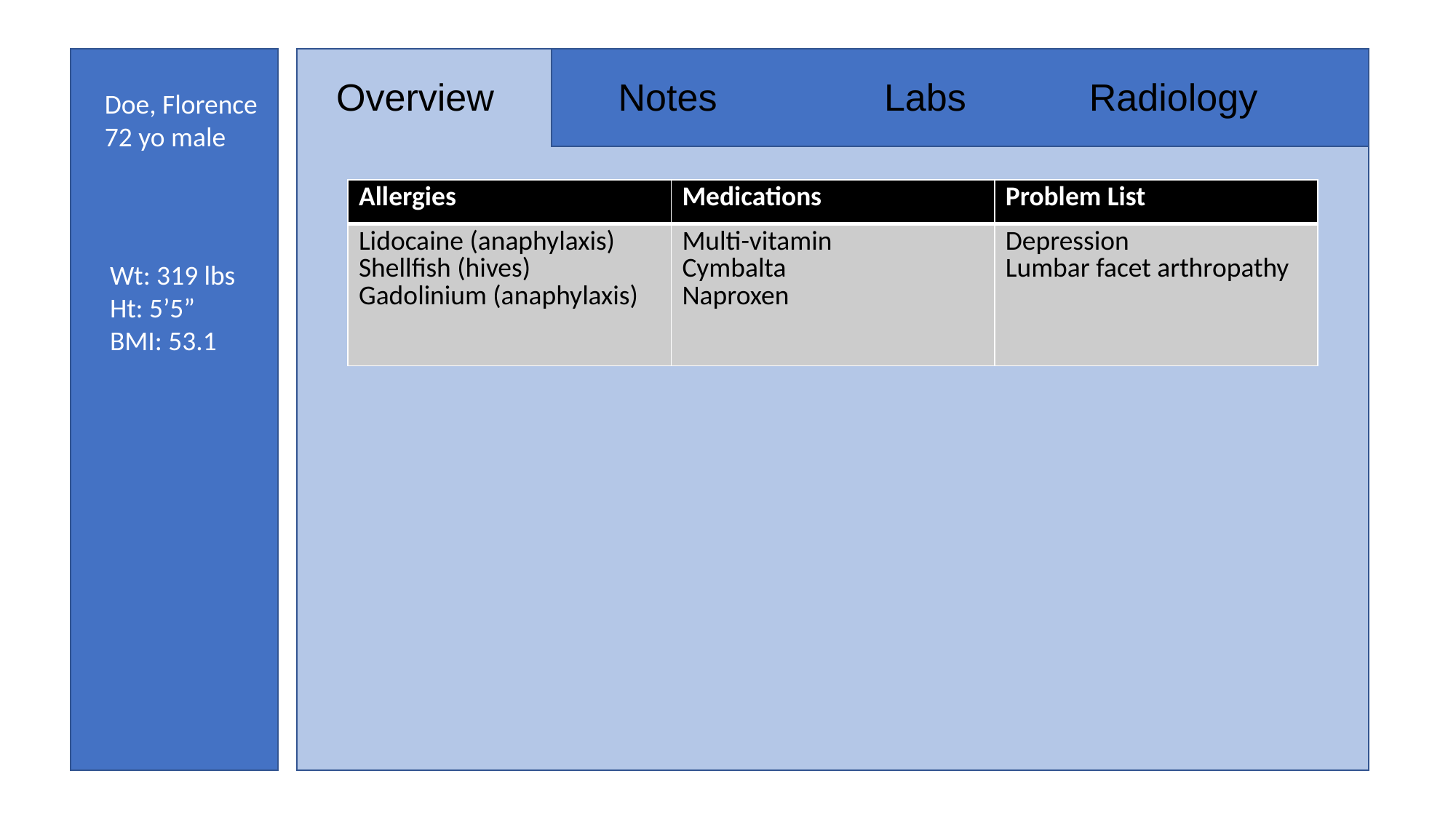

Overview
Notes
Labs
Radiology
Doe, Florence
72 yo male
| Allergies | Medications | Problem List |
| --- | --- | --- |
| Lidocaine (anaphylaxis) Shellfish (hives) Gadolinium (anaphylaxis) | Multi-vitamin Cymbalta Naproxen | Depression Lumbar facet arthropathy |
Wt: 319 lbs
Ht: 5’5”
BMI: 53.1

## Slide 23
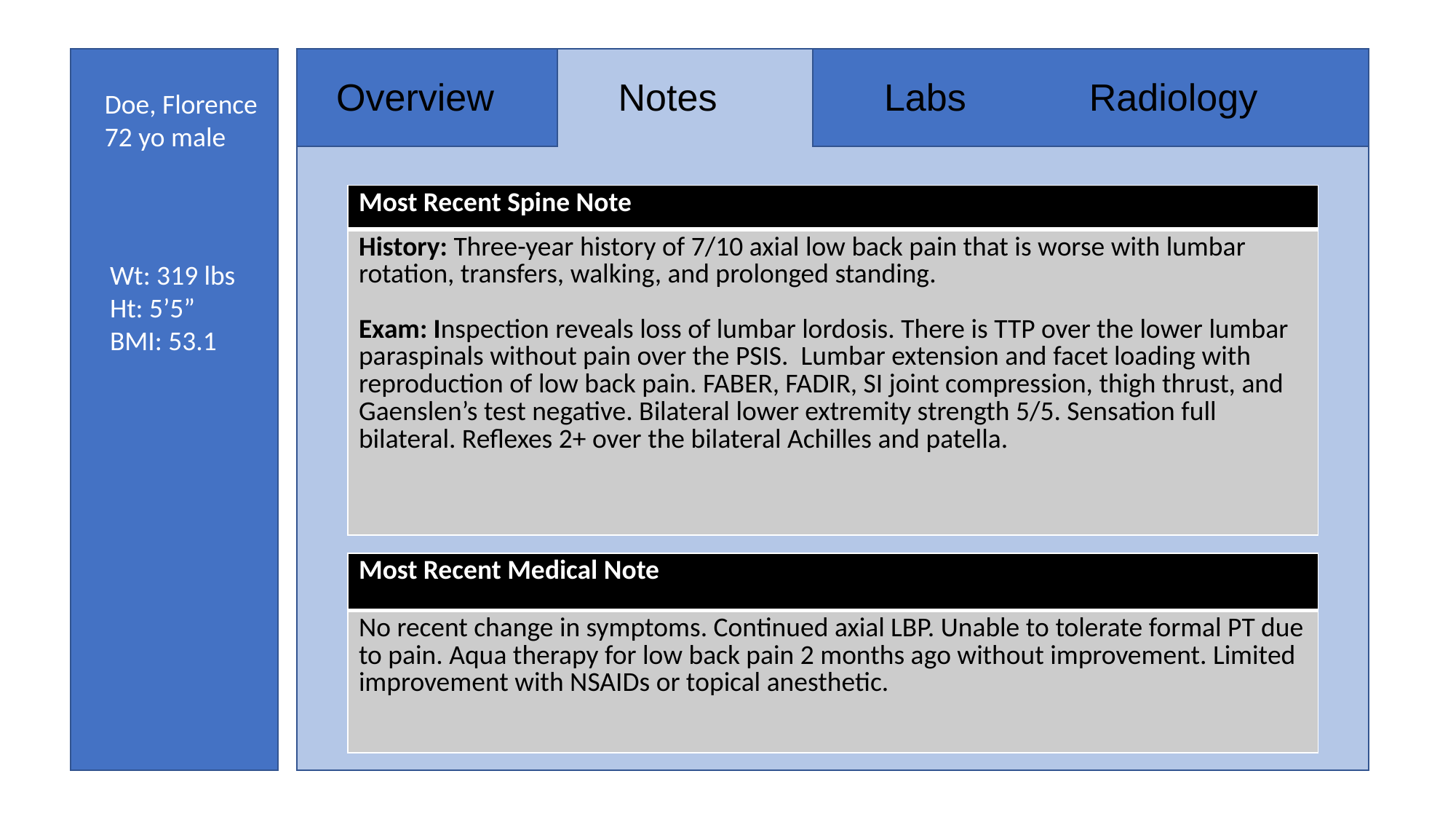

Overview
Notes
Labs
Radiology
Doe, Florence
72 yo male
| Most Recent Spine Note |
| --- |
| History: Three-year history of 7/10 axial low back pain that is worse with lumbar rotation, transfers, walking, and prolonged standing. Exam: Inspection reveals loss of lumbar lordosis. There is TTP over the lower lumbar paraspinals without pain over the PSIS. Lumbar extension and facet loading with reproduction of low back pain. FABER, FADIR, SI joint compression, thigh thrust, and Gaenslen’s test negative. Bilateral lower extremity strength 5/5. Sensation full bilateral. Reflexes 2+ over the bilateral Achilles and patella. |
Wt: 319 lbs
Ht: 5’5”
BMI: 53.1
| Most Recent Medical Note |
| --- |
| No recent change in symptoms. Continued axial LBP. Unable to tolerate formal PT due to pain. Aqua therapy for low back pain 2 months ago without improvement. Limited improvement with NSAIDs or topical anesthetic. |

## Slide 24
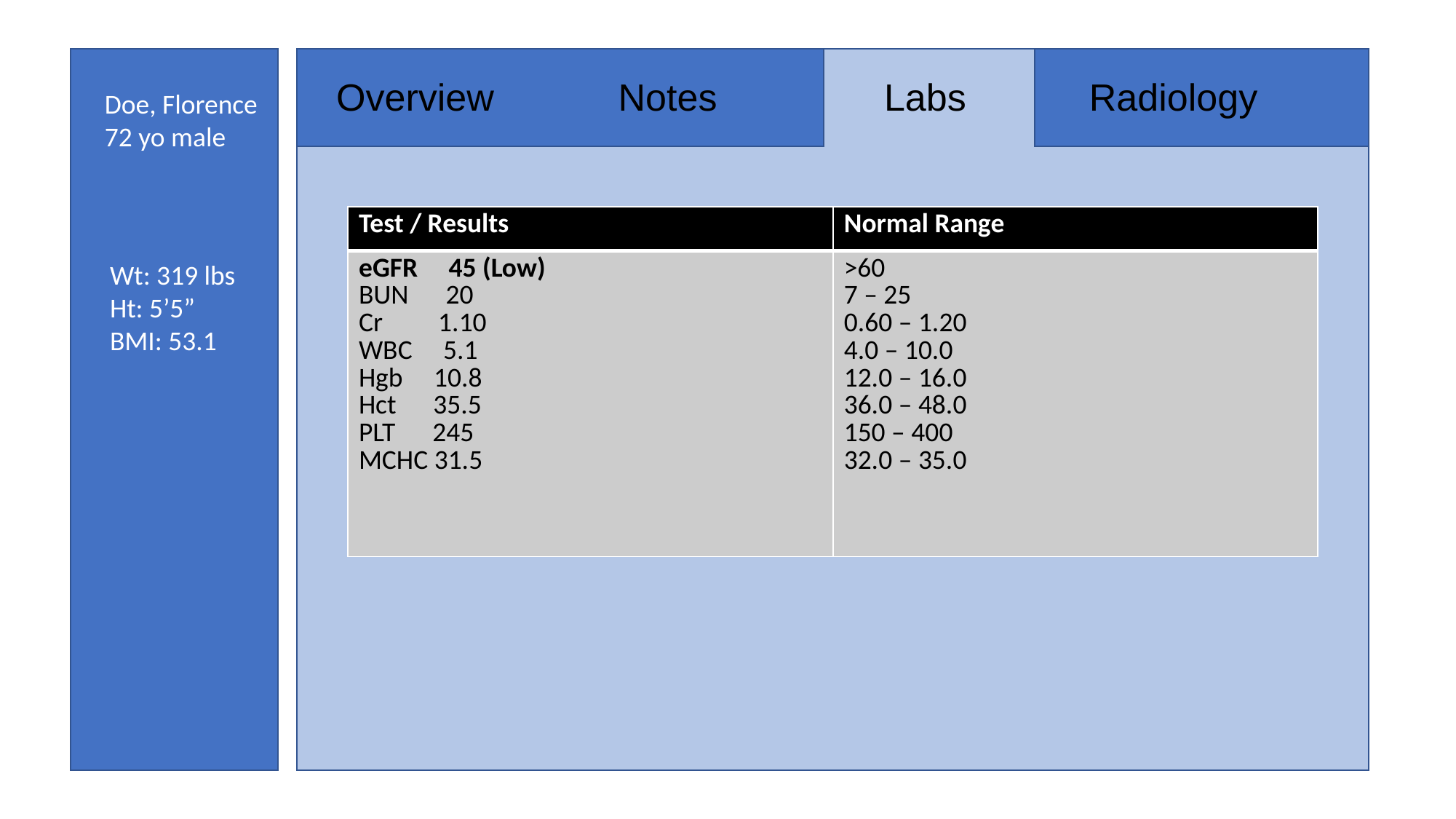

Overview
Notes
Labs
Radiology
Doe, Florence
72 yo male
| Test / Results | Normal Range |
| --- | --- |
| eGFR 45 (Low) BUN 20 Cr 1.10 WBC 5.1 Hgb 10.8 Hct 35.5 PLT 245 MCHC 31.5 | >60 7 – 25 0.60 – 1.20 4.0 – 10.0 12.0 – 16.0 36.0 – 48.0 150 – 400 32.0 – 35.0 |
Wt: 319 lbs
Ht: 5’5”
BMI: 53.1

## Slide 25
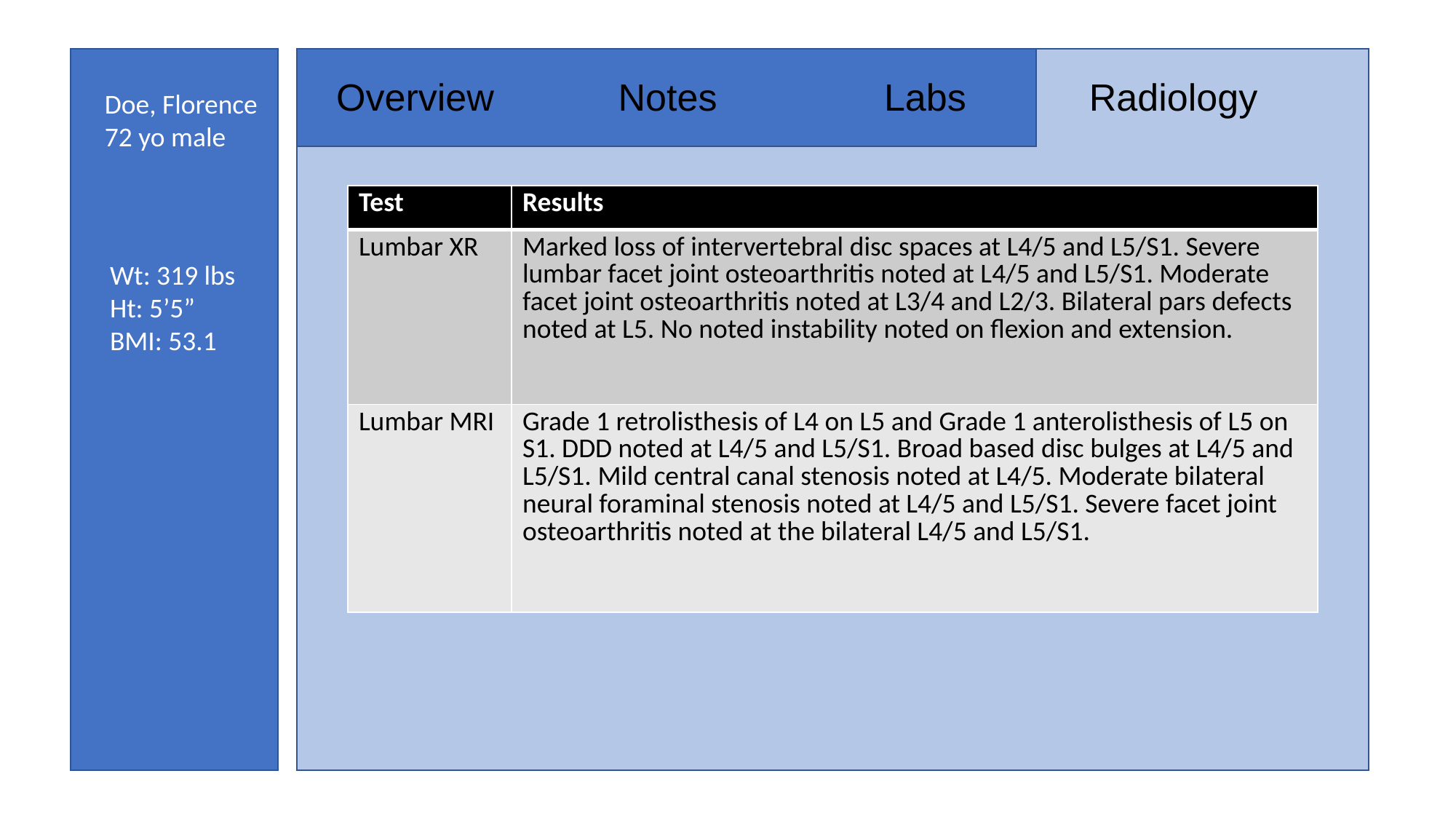

Overview
Notes
Labs
Radiology
Doe, Florence
72 yo male
| Test | Results |
| --- | --- |
| Lumbar XR | Marked loss of intervertebral disc spaces at L4/5 and L5/S1. Severe lumbar facet joint osteoarthritis noted at L4/5 and L5/S1. Moderate facet joint osteoarthritis noted at L3/4 and L2/3. Bilateral pars defects noted at L5. No noted instability noted on flexion and extension. |
| Lumbar MRI | Grade 1 retrolisthesis of L4 on L5 and Grade 1 anterolisthesis of L5 on S1. DDD noted at L4/5 and L5/S1. Broad based disc bulges at L4/5 and L5/S1. Mild central canal stenosis noted at L4/5. Moderate bilateral neural foraminal stenosis noted at L4/5 and L5/S1. Severe facet joint osteoarthritis noted at the bilateral L4/5 and L5/S1. |
Wt: 319 lbs
Ht: 5’5”
BMI: 53.1

## Slide 26
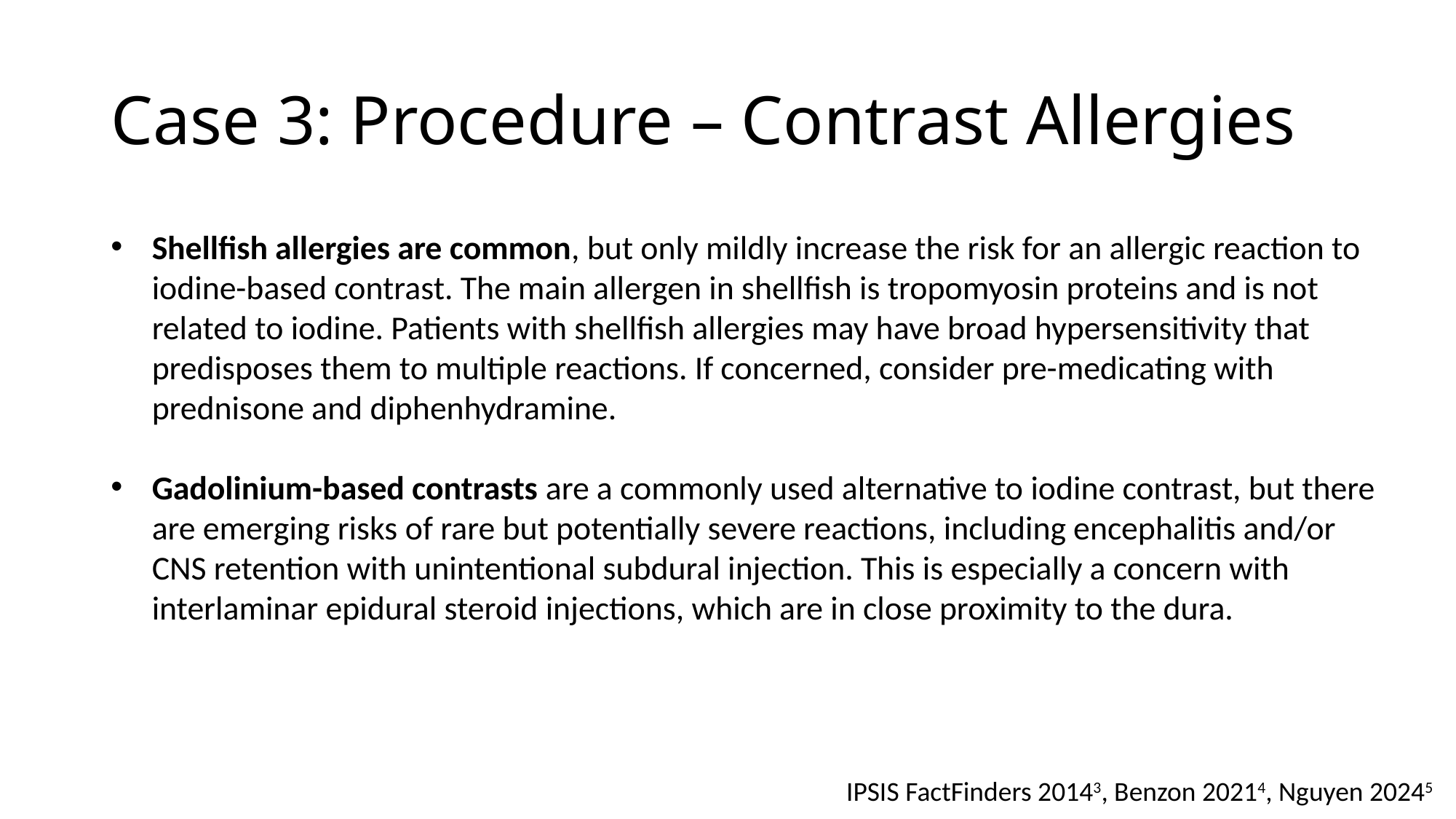

# Case 3: Procedure – Contrast Allergies
Shellfish allergies are common​, but only mildly increase the risk for an allergic reaction to iodine-based contrast. The main allergen in shellfish is tropomyosin proteins and is not related to iodine. Patients with shellfish allergies may have broad hypersensitivity that predisposes them to multiple reactions. If concerned, consider pre-medicating with prednisone and diphenhydramine.
Gadolinium-based contrasts are a commonly used alternative to iodine contrast, but there are emerging risks​ of rare but potentially severe reactions, including encephalitis and/or CNS retention with unintentional subdural injection. This is especially a concern with interlaminar epidural steroid injections, which are in close proximity to the dura. ​
IPSIS FactFinders 20143, Benzon 20214, Nguyen 20245

## Slide 27
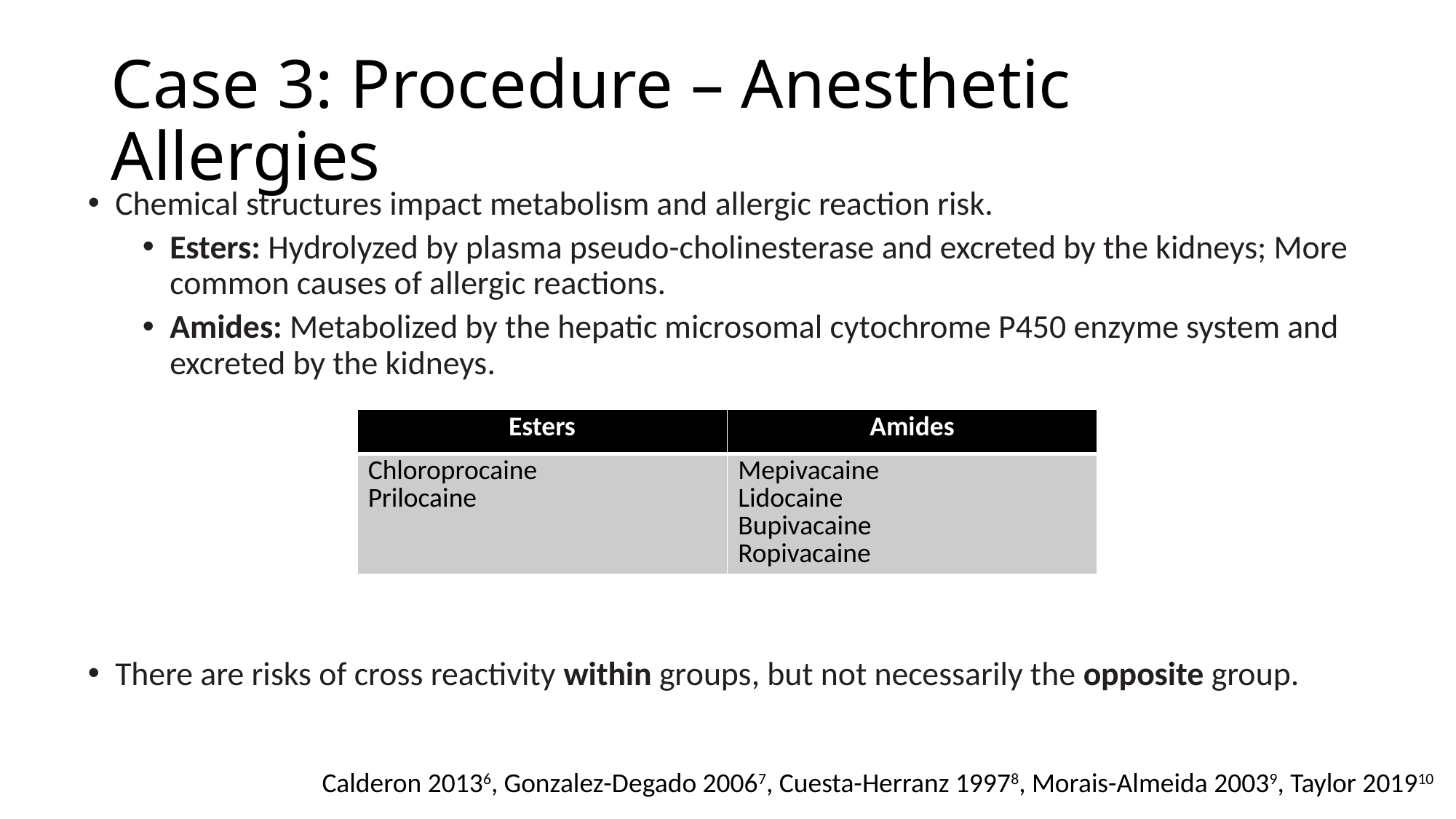

# Case 3: Procedure – Anesthetic Allergies
Chemical structures impact metabolism and allergic reaction risk.
Esters: Hydrolyzed by plasma pseudo-cholinesterase and excreted by the kidneys; More common causes of allergic reactions.
Amides: Metabolized by the hepatic microsomal cytochrome P450 enzyme system and excreted by the kidneys.
There are risks of cross reactivity within groups, but not necessarily the opposite group.
| Esters | Amides |
| --- | --- |
| Chloroprocaine Prilocaine | Mepivacaine Lidocaine Bupivacaine Ropivacaine |
Calderon 20136, Gonzalez-Degado 20067, Cuesta-Herranz 19978, Morais-Almeida 20039, Taylor 201910

## Slide 28
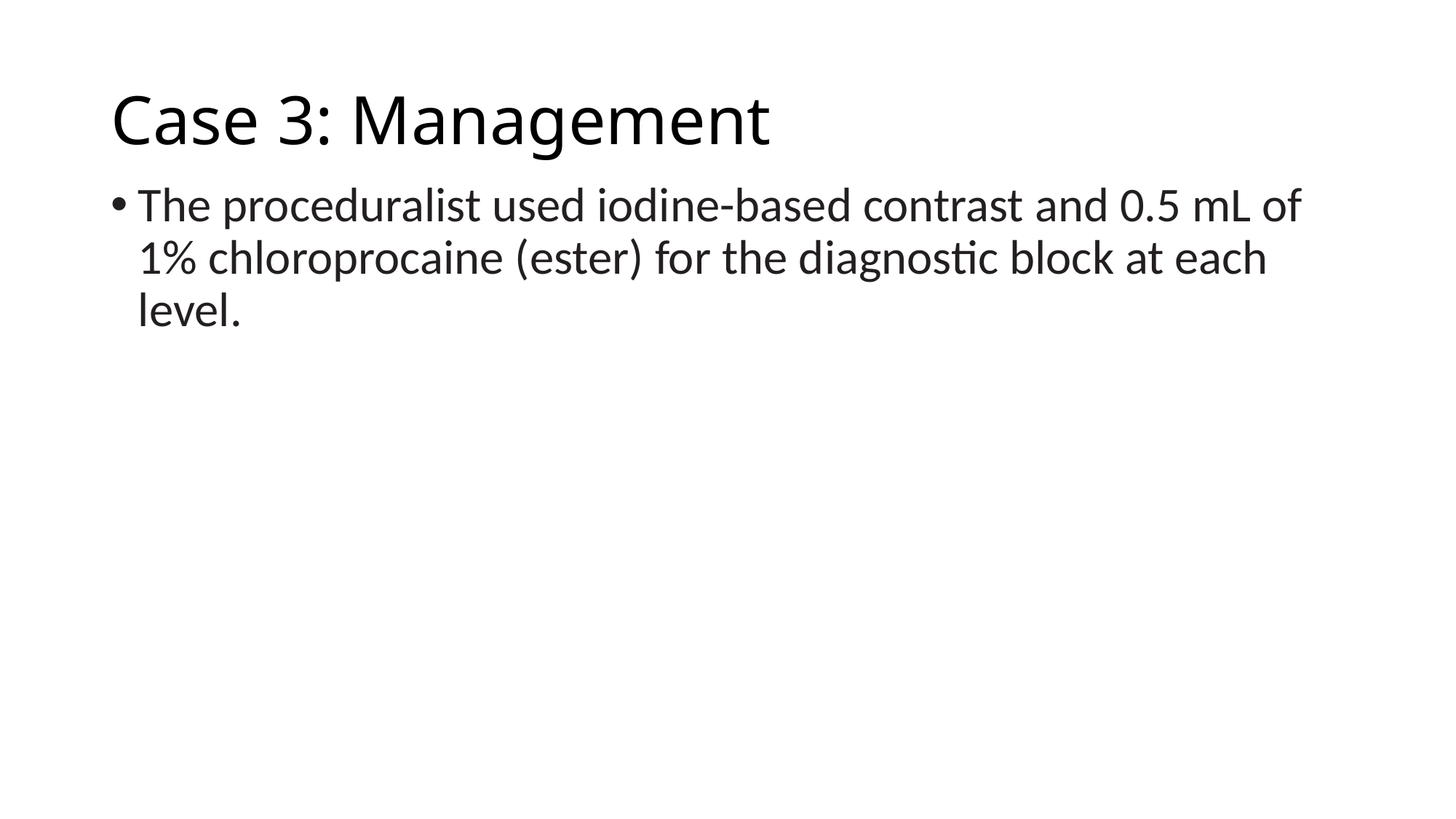

# Case 3: Management
The proceduralist used iodine-based contrast and 0.5 mL of 1% chloroprocaine (ester) for the diagnostic block at each level.

## Slide 29
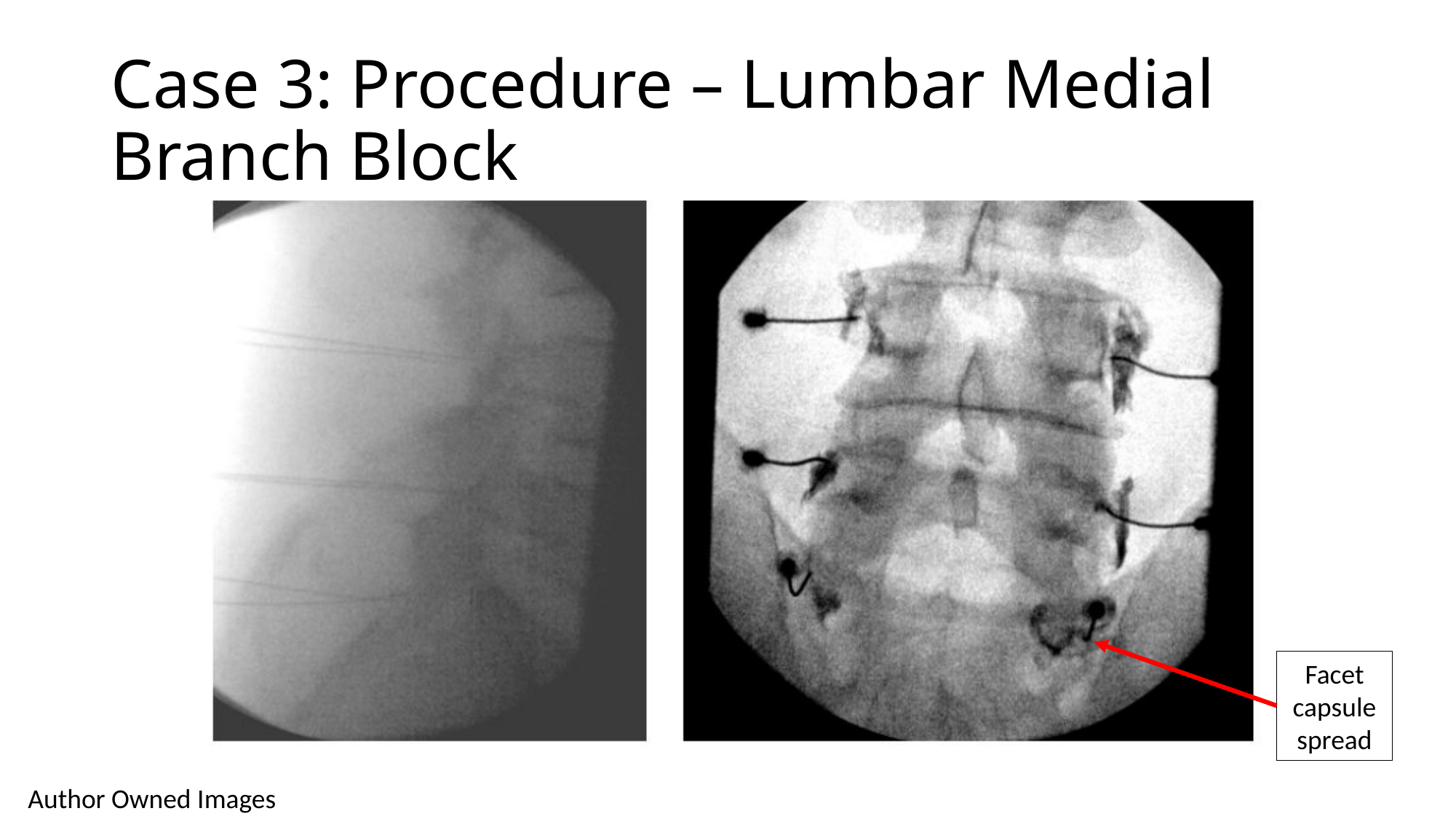

# Case 3: Procedure – Lumbar Medial Branch Block
Facet capsule spread
Author Owned Images

## Slide 30
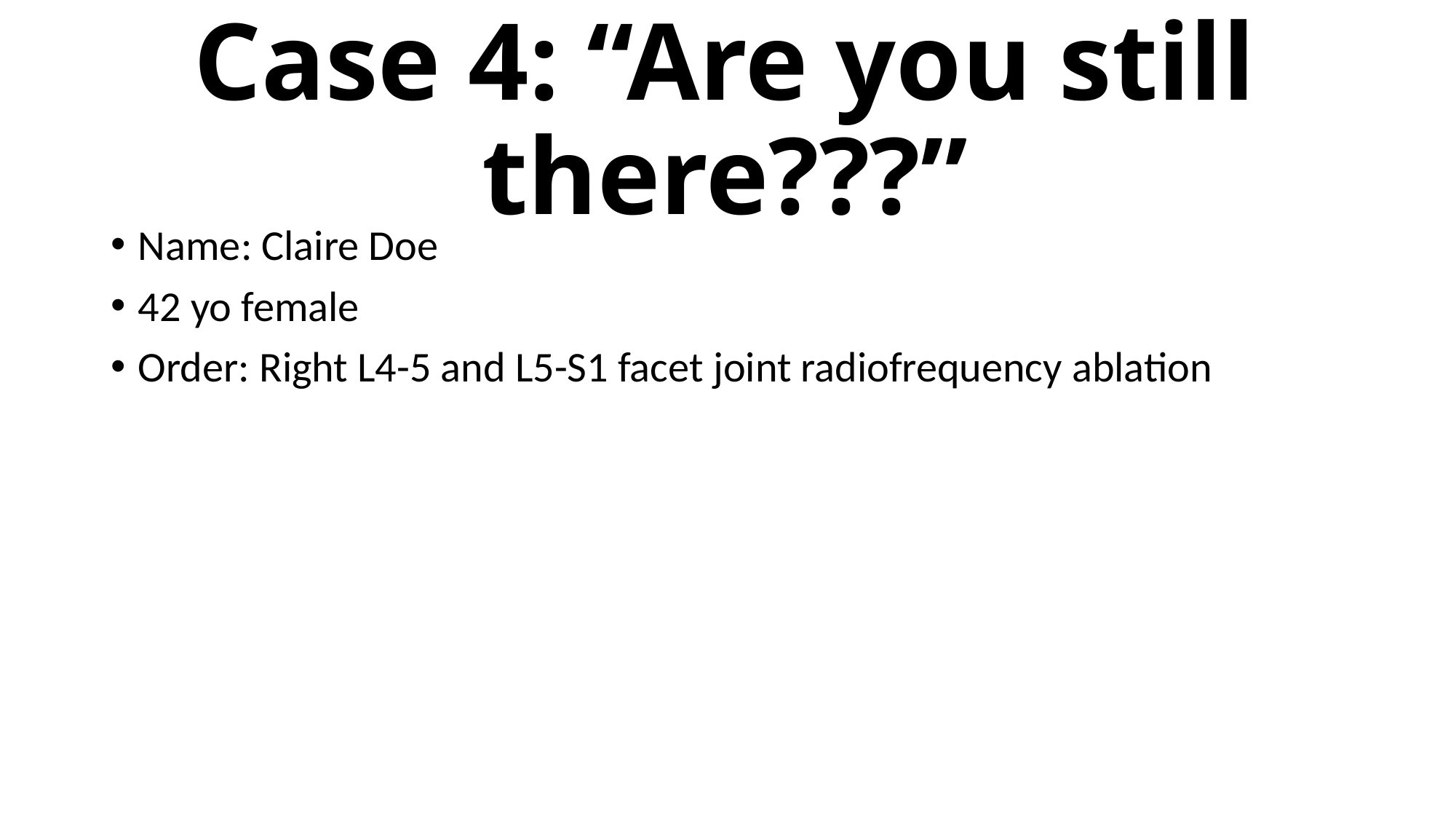

# Case 4: “Are you still there???”
Name: Claire Doe
42 yo female
Order: Right L4-5 and L5-S1 facet joint radiofrequency ablation

## Slide 31
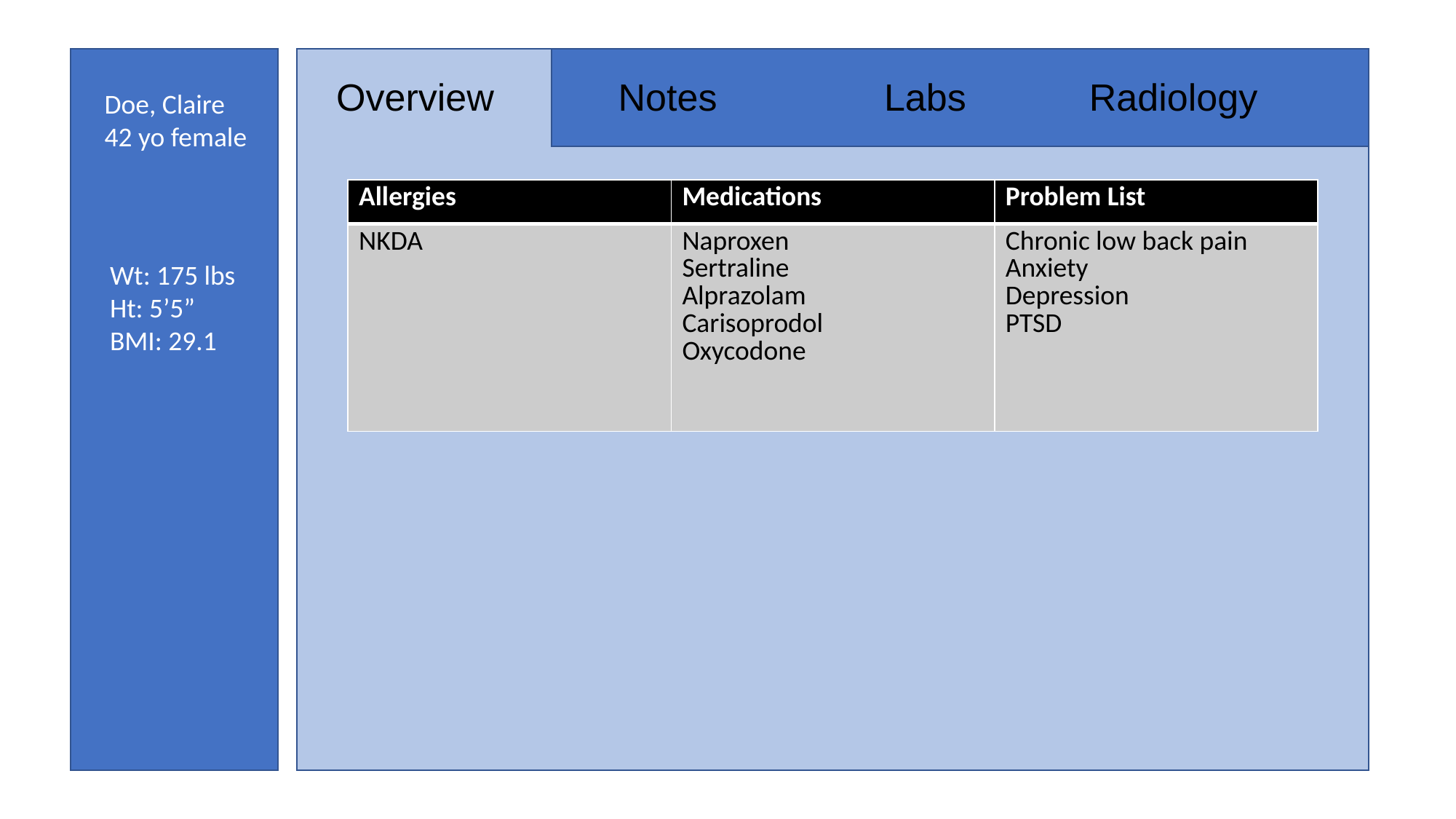

Overview
Notes
Labs
Radiology
Doe, Claire
42 yo female
| Allergies | Medications | Problem List |
| --- | --- | --- |
| NKDA | Naproxen Sertraline Alprazolam Carisoprodol Oxycodone | Chronic low back pain Anxiety Depression PTSD |
Wt: 175 lbs
Ht: 5’5”
BMI: 29.1

## Slide 32
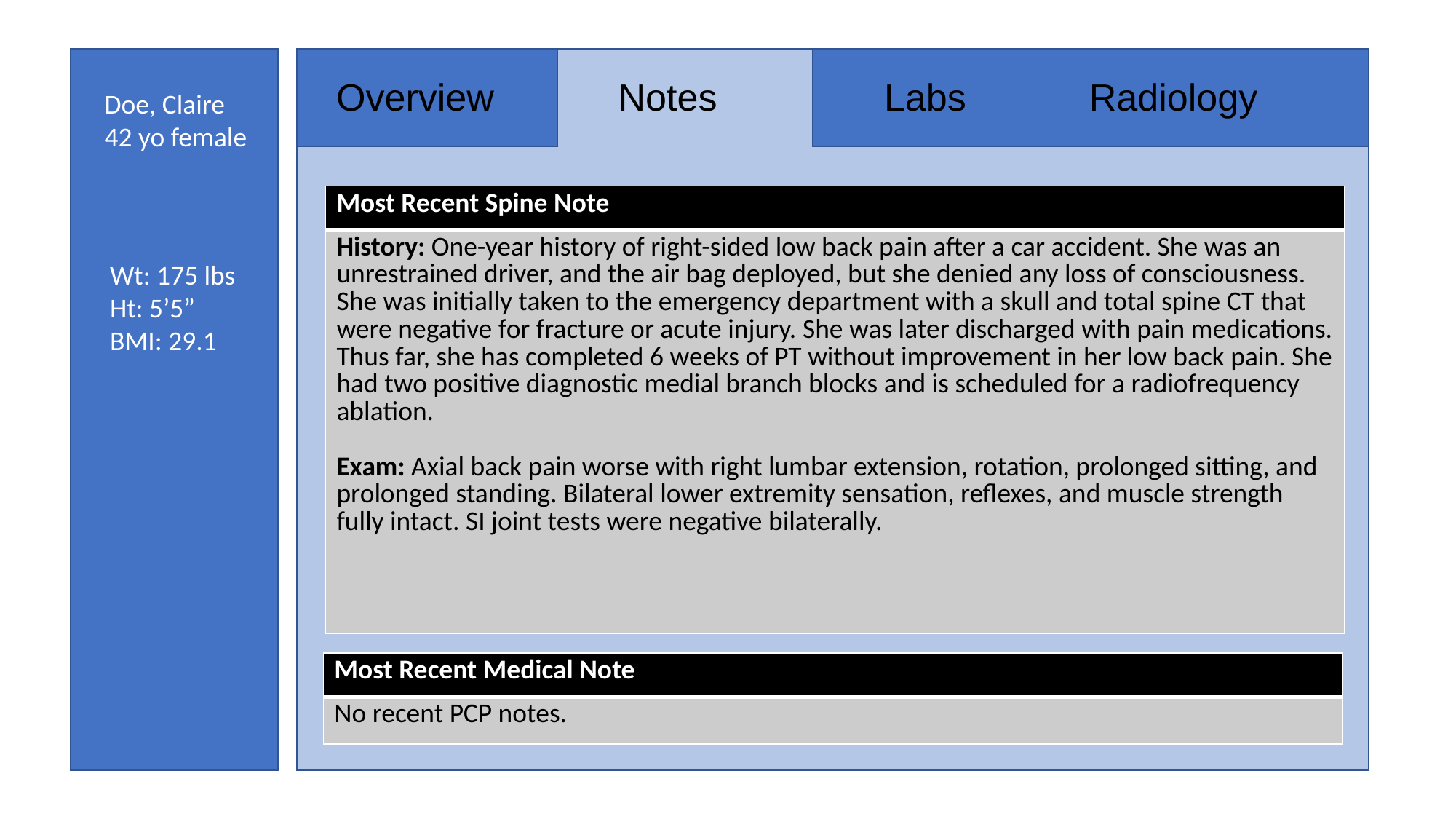

Overview
Notes
Labs
Radiology
Doe, Claire
42 yo female
| Most Recent Spine Note |
| --- |
| History: One-year history of right-sided low back pain after a car accident. She was an unrestrained driver, and the air bag deployed, but she denied any loss of consciousness. She was initially taken to the emergency department with a skull and total spine CT that were negative for fracture or acute injury. She was later discharged with pain medications. Thus far, she has completed 6 weeks of PT without improvement in her low back pain. She had two positive diagnostic medial branch blocks and is scheduled for a radiofrequency ablation. Exam: Axial back pain worse with right lumbar extension, rotation, prolonged sitting, and prolonged standing. Bilateral lower extremity sensation, reflexes, and muscle strength fully intact. SI joint tests were negative bilaterally. |
Wt: 175 lbs
Ht: 5’5”
BMI: 29.1
| Most Recent Medical Note |
| --- |
| No recent PCP notes. |

## Slide 33
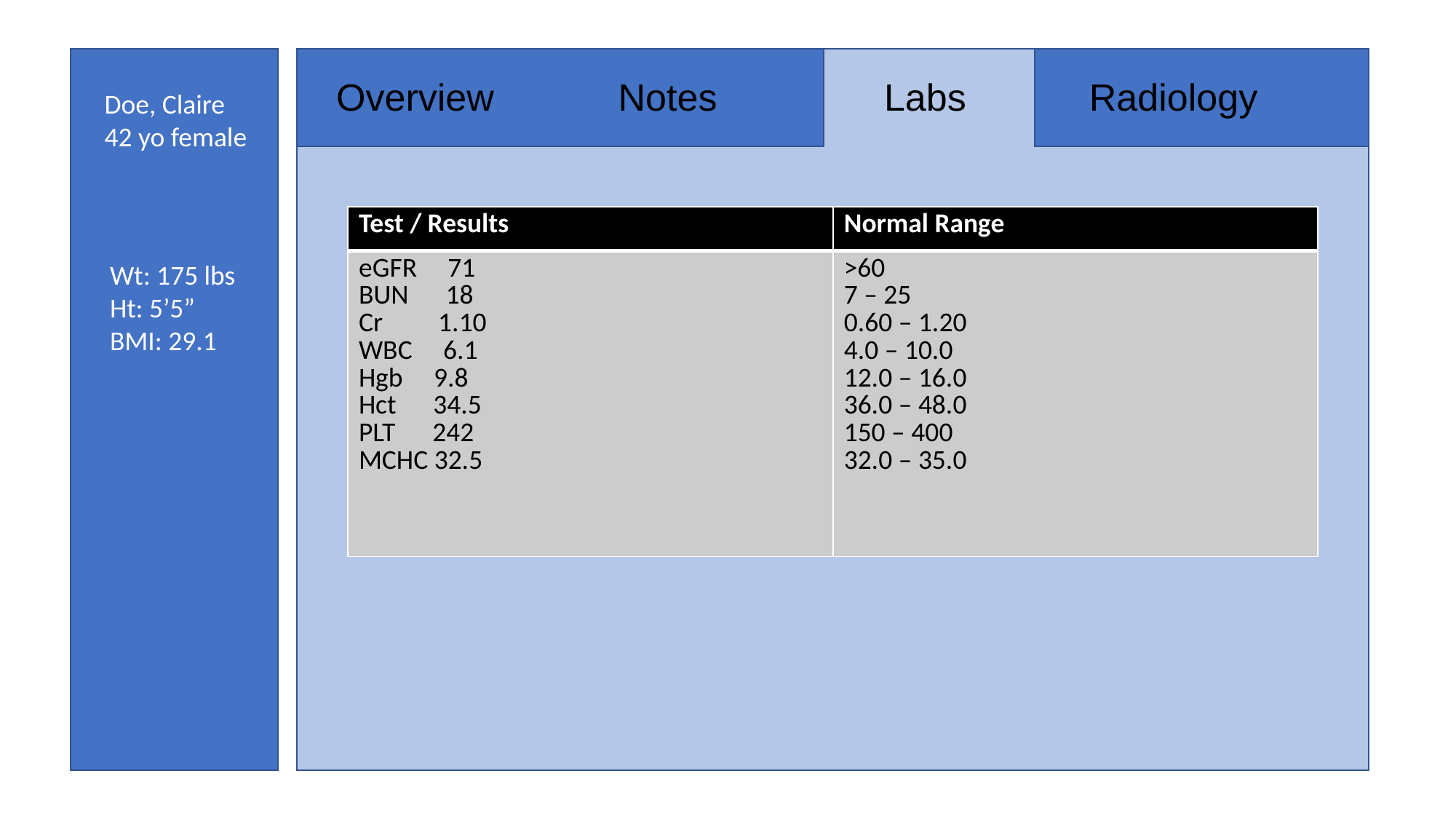

Overview
Notes
Labs
Radiology
Doe, Claire
42 yo female
| Test / Results | Normal Range |
| --- | --- |
| eGFR 71 BUN 18 Cr 1.10 WBC 6.1 Hgb 9.8 Hct 34.5 PLT 242 MCHC 32.5 | >60 7 – 25 0.60 – 1.20 4.0 – 10.0 12.0 – 16.0 36.0 – 48.0 150 – 400 32.0 – 35.0 |
Wt: 175 lbs
Ht: 5’5”
BMI: 29.1

## Slide 34
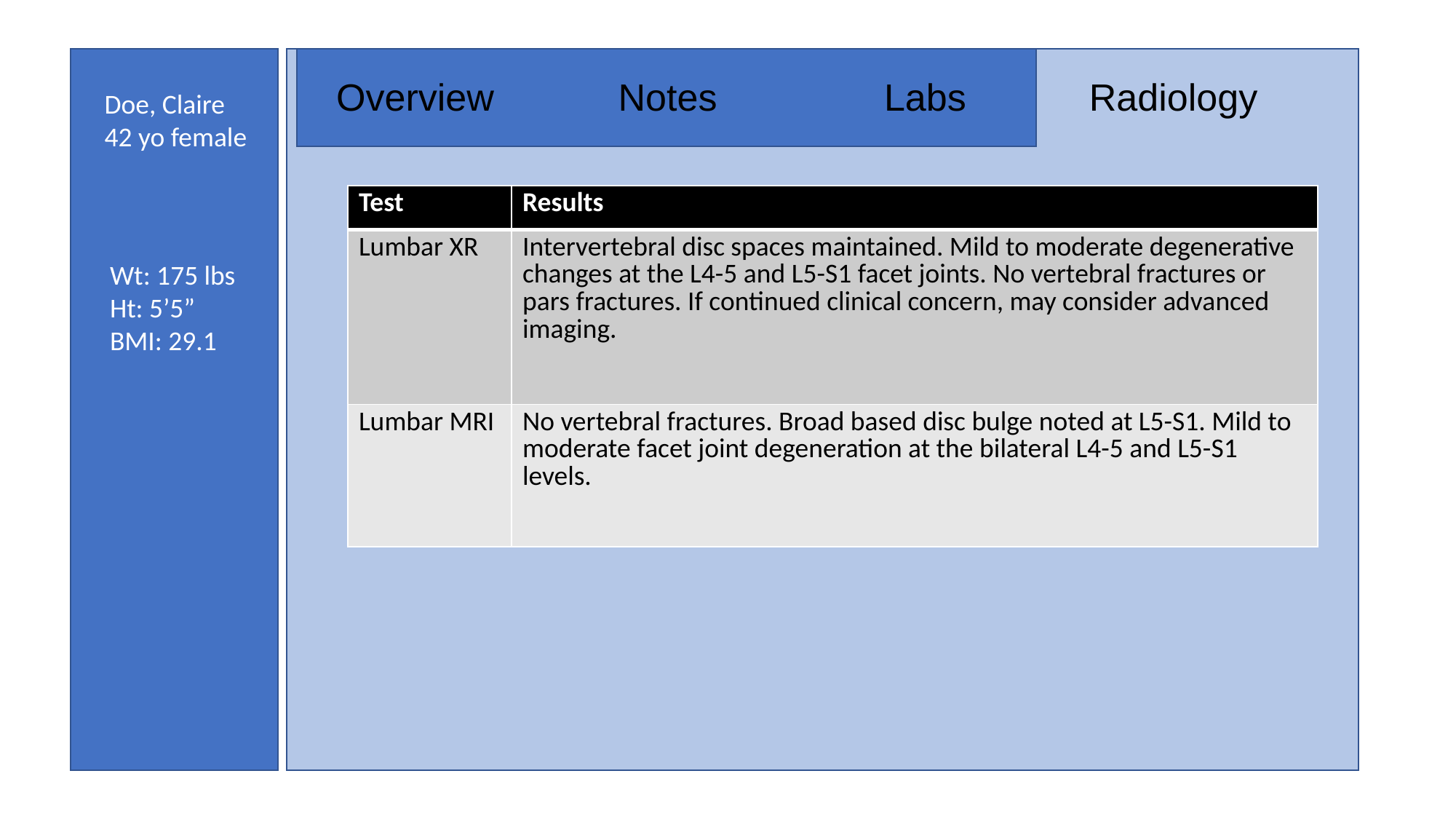

Overview
Notes
Labs
Radiology
Doe, Claire
42 yo female
| Test | Results |
| --- | --- |
| Lumbar XR | Intervertebral disc spaces maintained. Mild to moderate degenerative changes at the L4-5 and L5-S1 facet joints. No vertebral fractures or pars fractures. If continued clinical concern, may consider advanced imaging. |
| Lumbar MRI | No vertebral fractures. Broad based disc bulge noted at L5-S1. Mild to moderate facet joint degeneration at the bilateral L4-5 and L5-S1 levels. |
Wt: 175 lbs
Ht: 5’5”
BMI: 29.1

## Slide 35
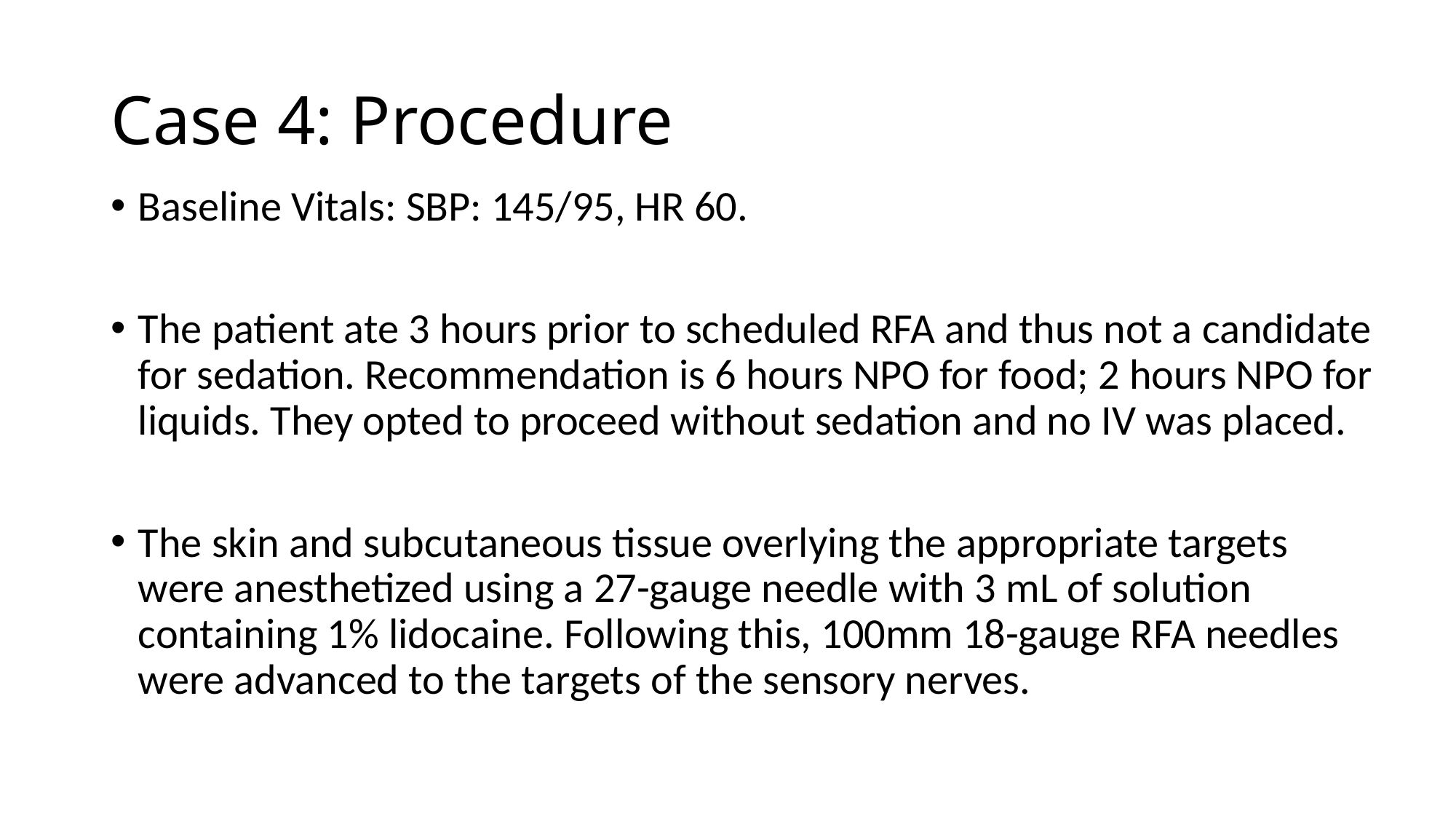

# Case 4: Procedure
Baseline Vitals: SBP: 145/95, HR 60.
The patient ate 3 hours prior to scheduled RFA and thus not a candidate for sedation. Recommendation is 6 hours NPO for food; 2 hours NPO for liquids. They opted to proceed without sedation and no IV was placed.
The skin and subcutaneous tissue overlying the appropriate targets were anesthetized using a 27-gauge needle with 3 mL of solution containing 1% lidocaine. Following this, 100mm 18-gauge RFA needles were advanced to the targets of the sensory nerves.

## Slide 36
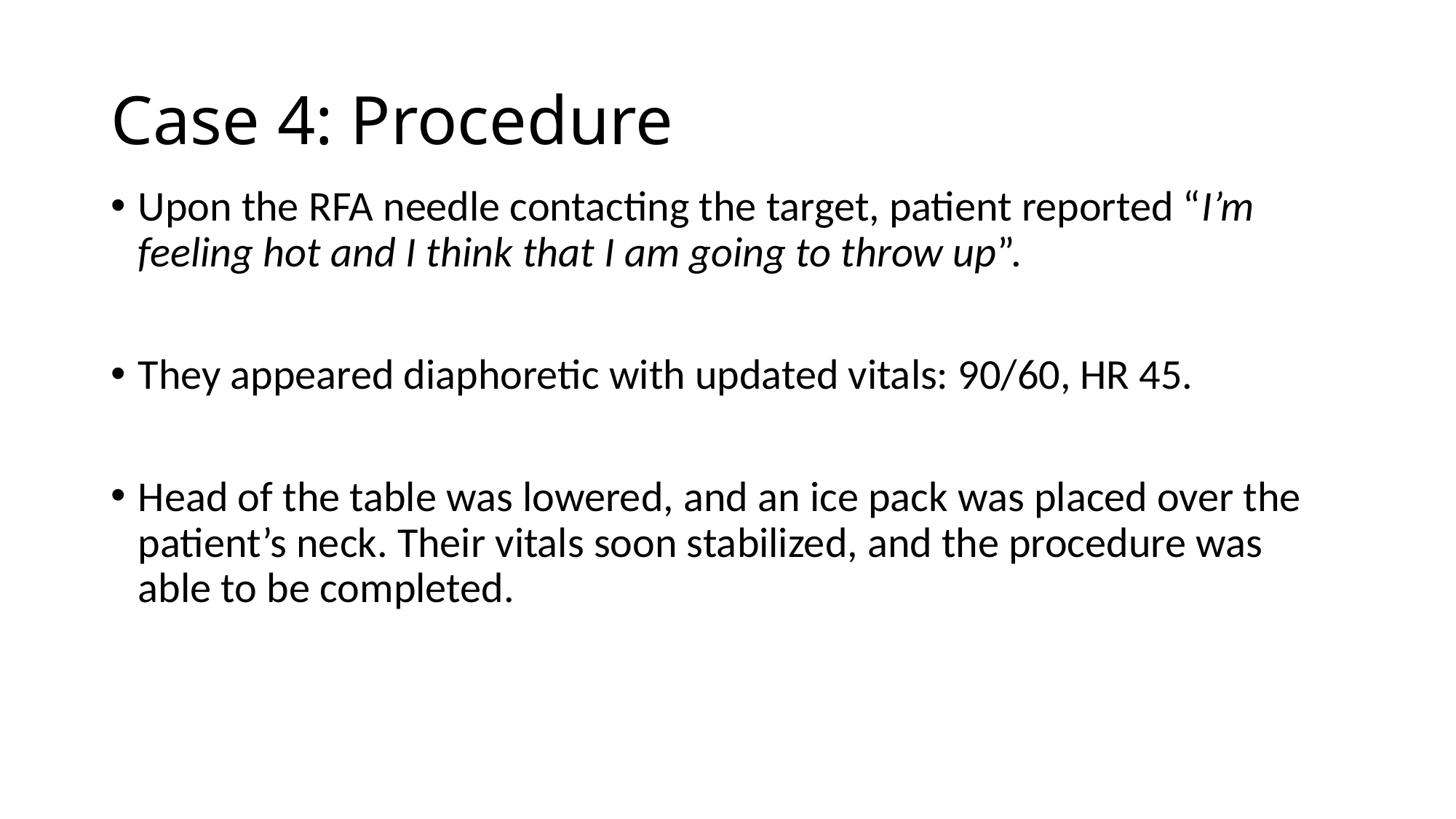

# Case 4: Procedure
Upon the RFA needle contacting the target, patient reported “I’m feeling hot and I think that I am going to throw up”.
They appeared diaphoretic with updated vitals: 90/60, HR 45.
Head of the table was lowered, and an ice pack was placed over the patient’s neck. Their vitals soon stabilized, and the procedure was able to be completed.

## Slide 37
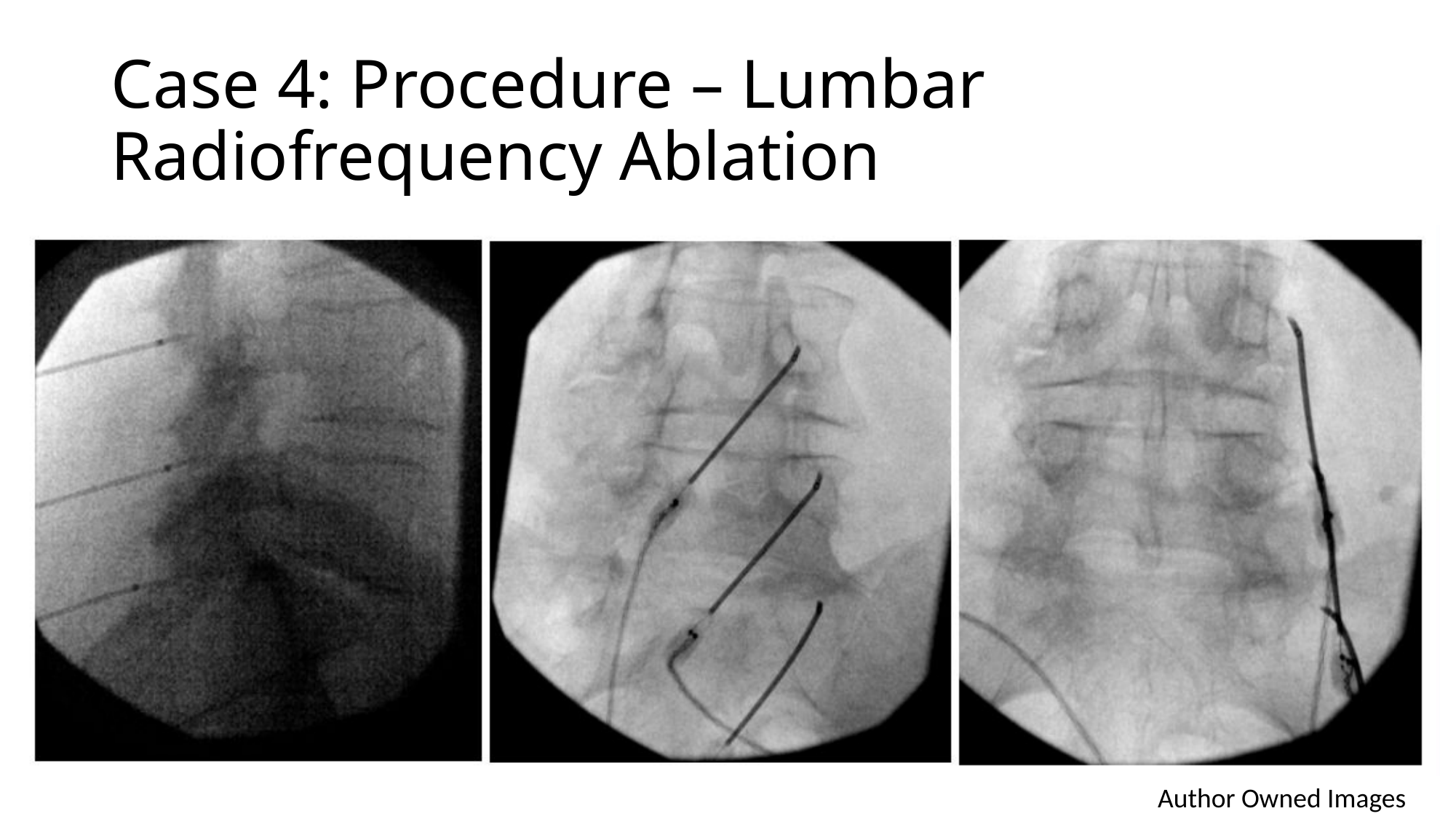

# Case 4: Procedure – Lumbar Radiofrequency Ablation
Author Owned Images

## Slide 38
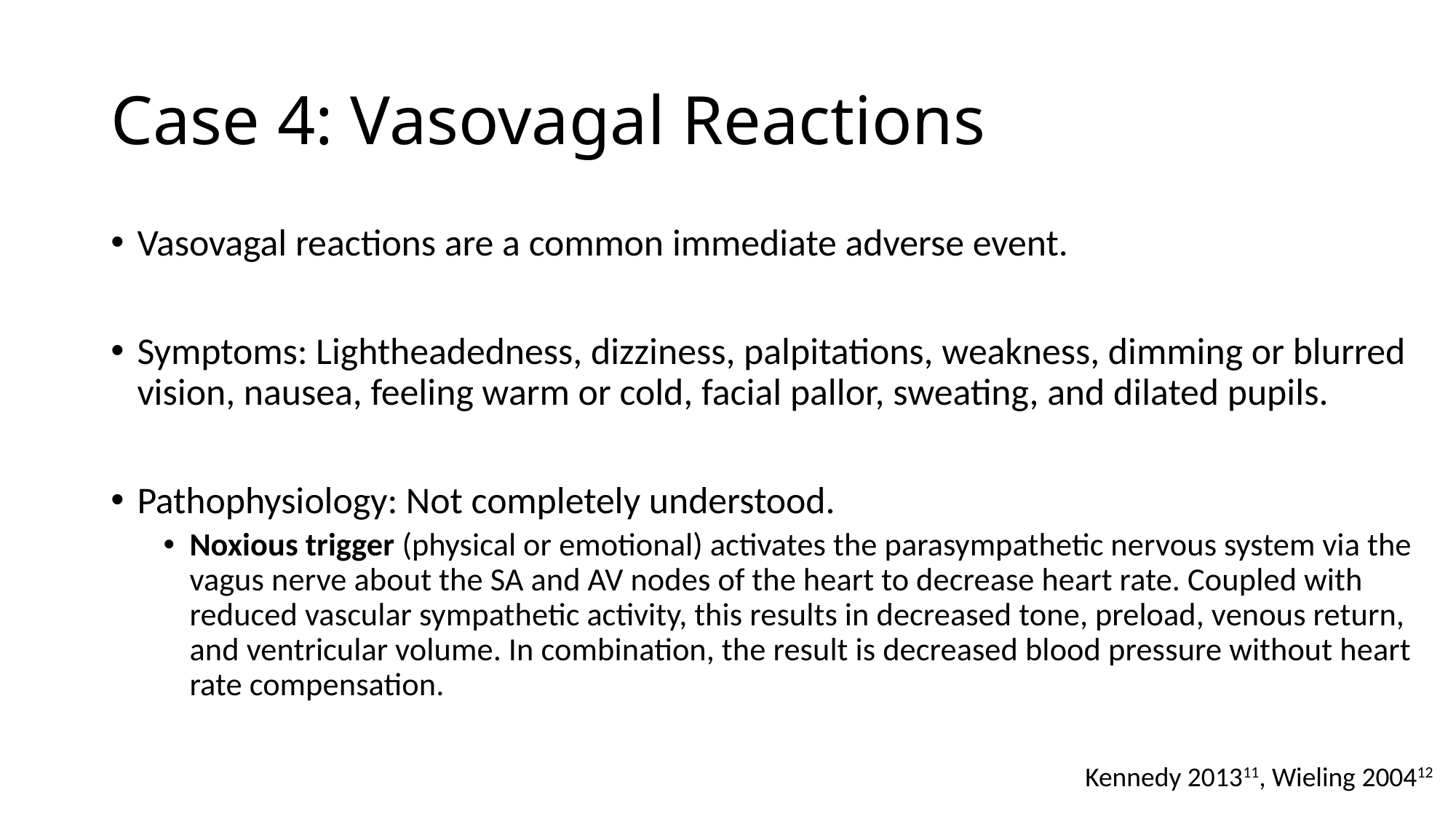

# Case 4: Vasovagal Reactions
Vasovagal reactions are a common immediate adverse event.
Symptoms: Lightheadedness, dizziness, palpitations, weakness, dimming or blurred vision, nausea, feeling warm or cold, facial pallor, sweating, and dilated pupils.
Pathophysiology: Not completely understood.
Noxious trigger (physical or emotional) activates the parasympathetic nervous system via the vagus nerve about the SA and AV nodes of the heart to decrease heart rate. Coupled with reduced vascular sympathetic activity, this results in decreased tone, preload, venous return, and ventricular volume. In combination, the result is decreased blood pressure without heart rate compensation.
Kennedy 201311, Wieling 200412

## Slide 39
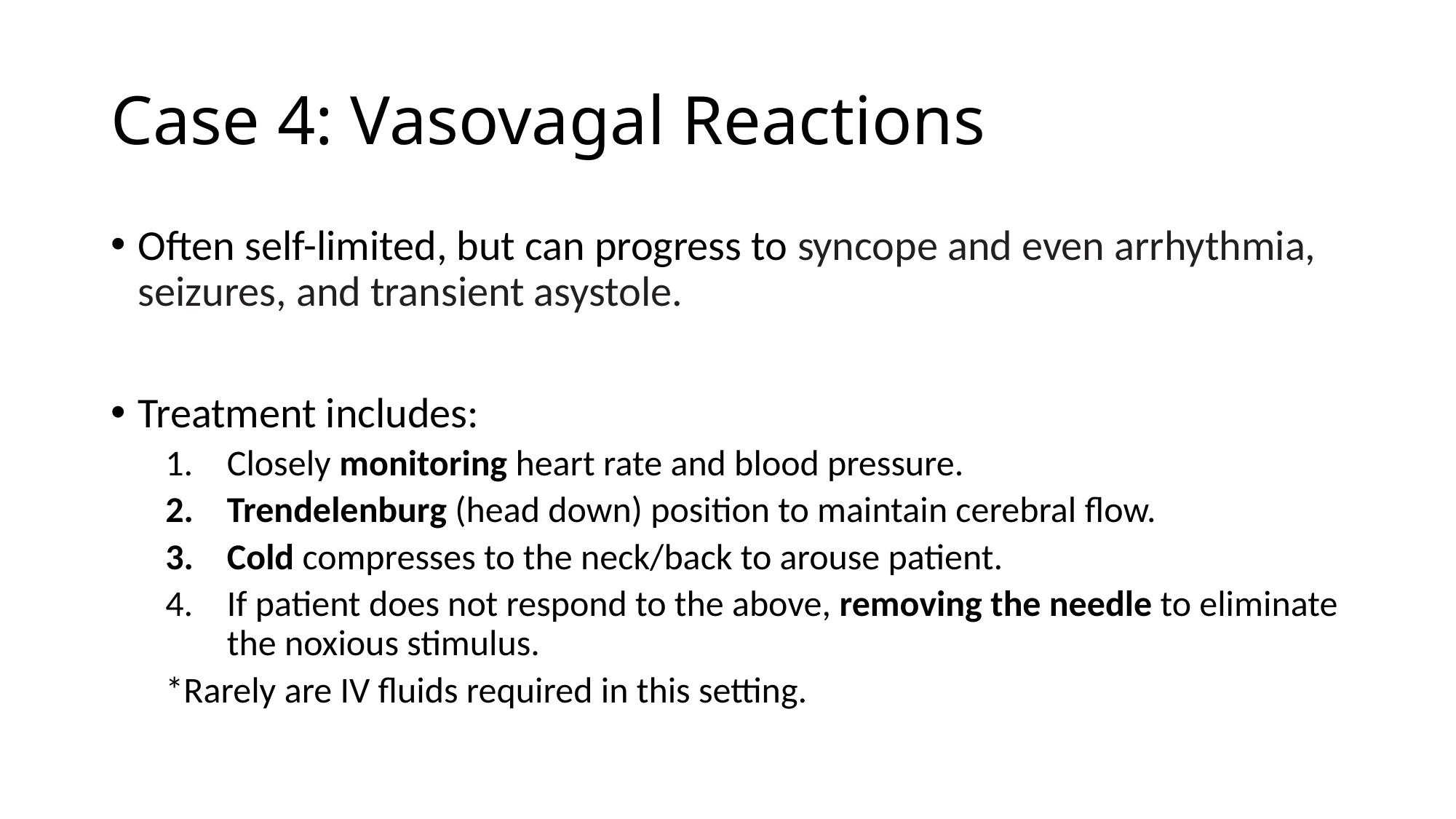

# Case 4: Vasovagal Reactions
Often self-limited, but can progress to syncope and even arrhythmia, seizures, and transient asystole.
Treatment includes:
Closely monitoring heart rate and blood pressure.
Trendelenburg (head down) position to maintain cerebral flow.
Cold compresses to the neck/back to arouse patient.
If patient does not respond to the above, removing the needle to eliminate the noxious stimulus.
*Rarely are IV fluids required in this setting.

## Slide 40
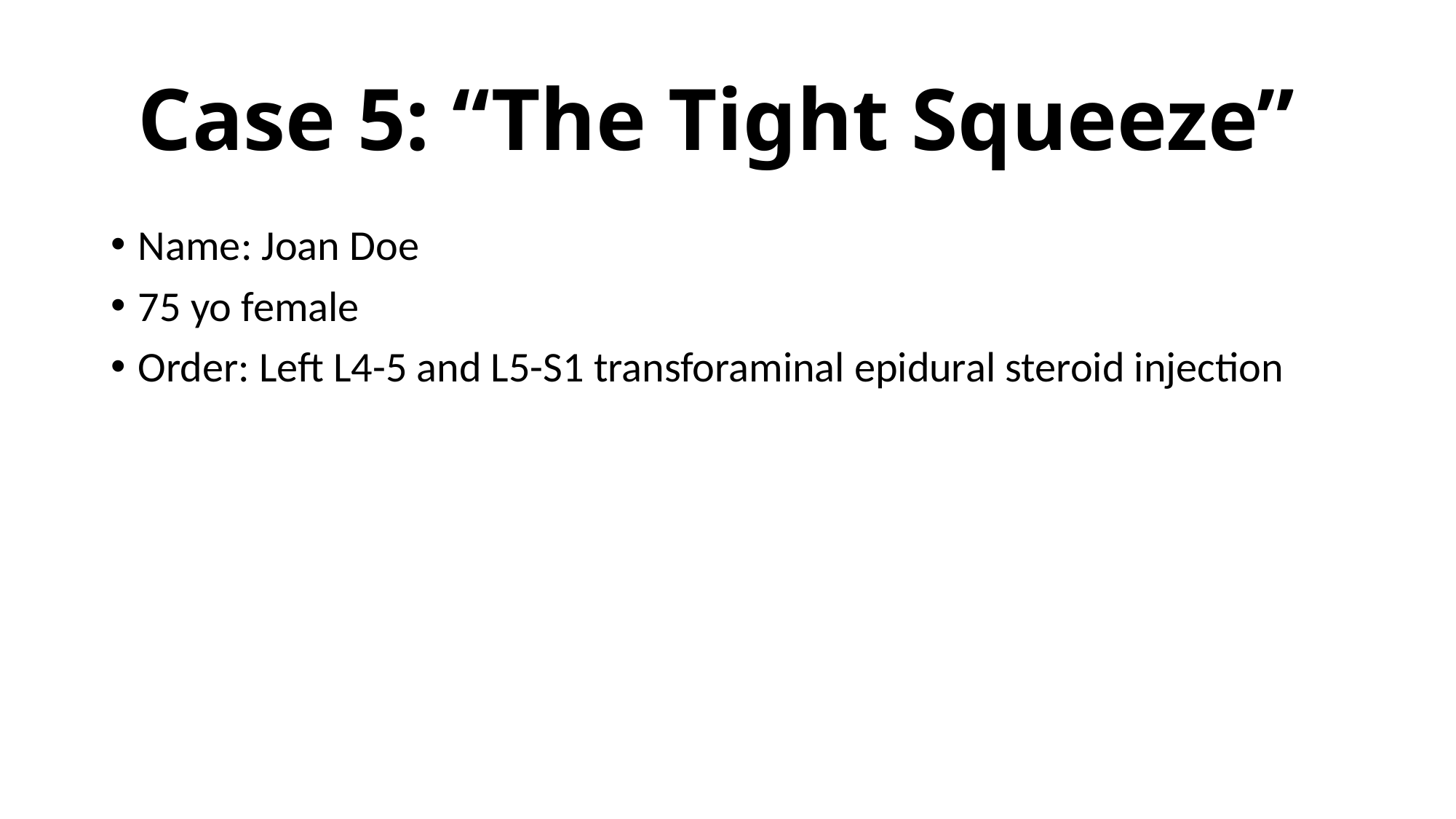

# Case 5: “The Tight Squeeze”
Name: Joan Doe
75 yo female
Order: Left L4-5 and L5-S1 transforaminal epidural steroid injection

## Slide 41
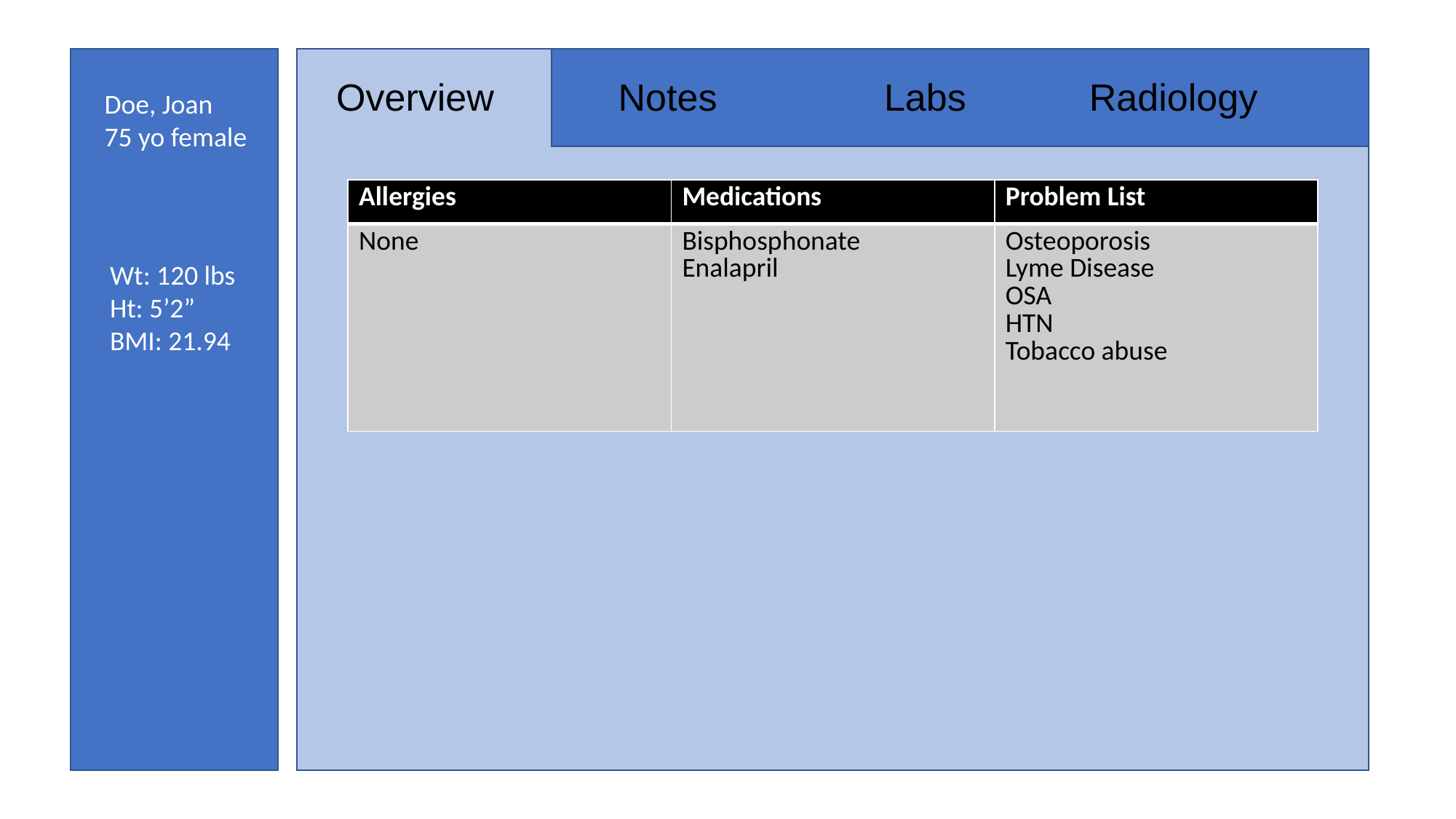

Overview
Notes
Labs
Radiology
Doe, Joan
75 yo female
| Allergies | Medications | Problem List |
| --- | --- | --- |
| None | Bisphosphonate Enalapril | Osteoporosis Lyme Disease OSA HTN Tobacco abuse |
Wt: 120 lbs
Ht: 5’2”
BMI: 21.94

## Slide 42
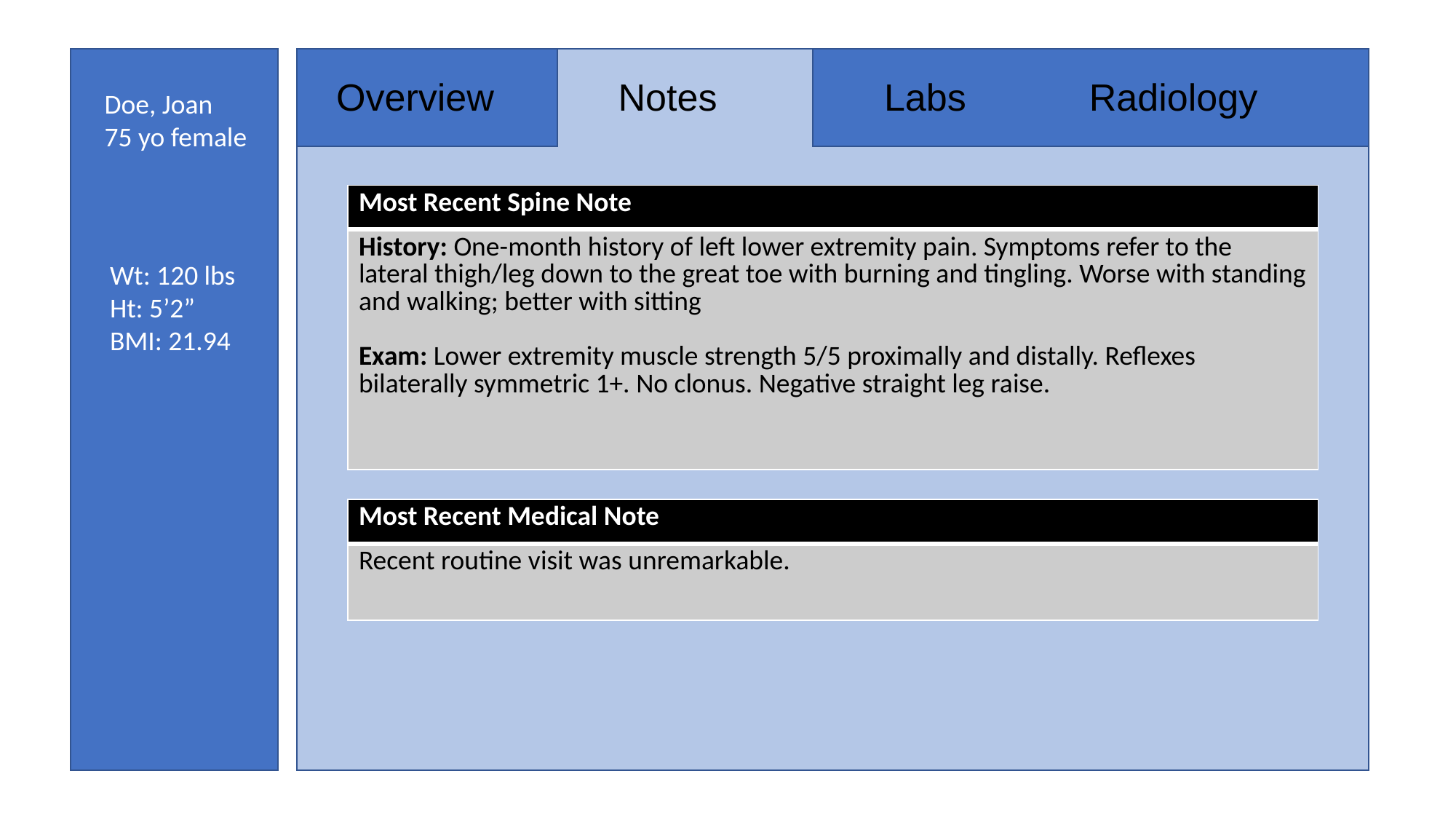

Overview
Notes
Labs
Radiology
Doe, Joan
75 yo female
| Most Recent Spine Note |
| --- |
| History: One-month history of left lower extremity pain. Symptoms refer to the lateral thigh/leg down to the great toe with burning and tingling. Worse with standing and walking; better with sitting Exam: Lower extremity muscle strength 5/5 proximally and distally. Reflexes bilaterally symmetric 1+. No clonus. Negative straight leg raise. |
Wt: 120 lbs
Ht: 5’2”
BMI: 21.94
| Most Recent Medical Note |
| --- |
| Recent routine visit was unremarkable. |

## Slide 43
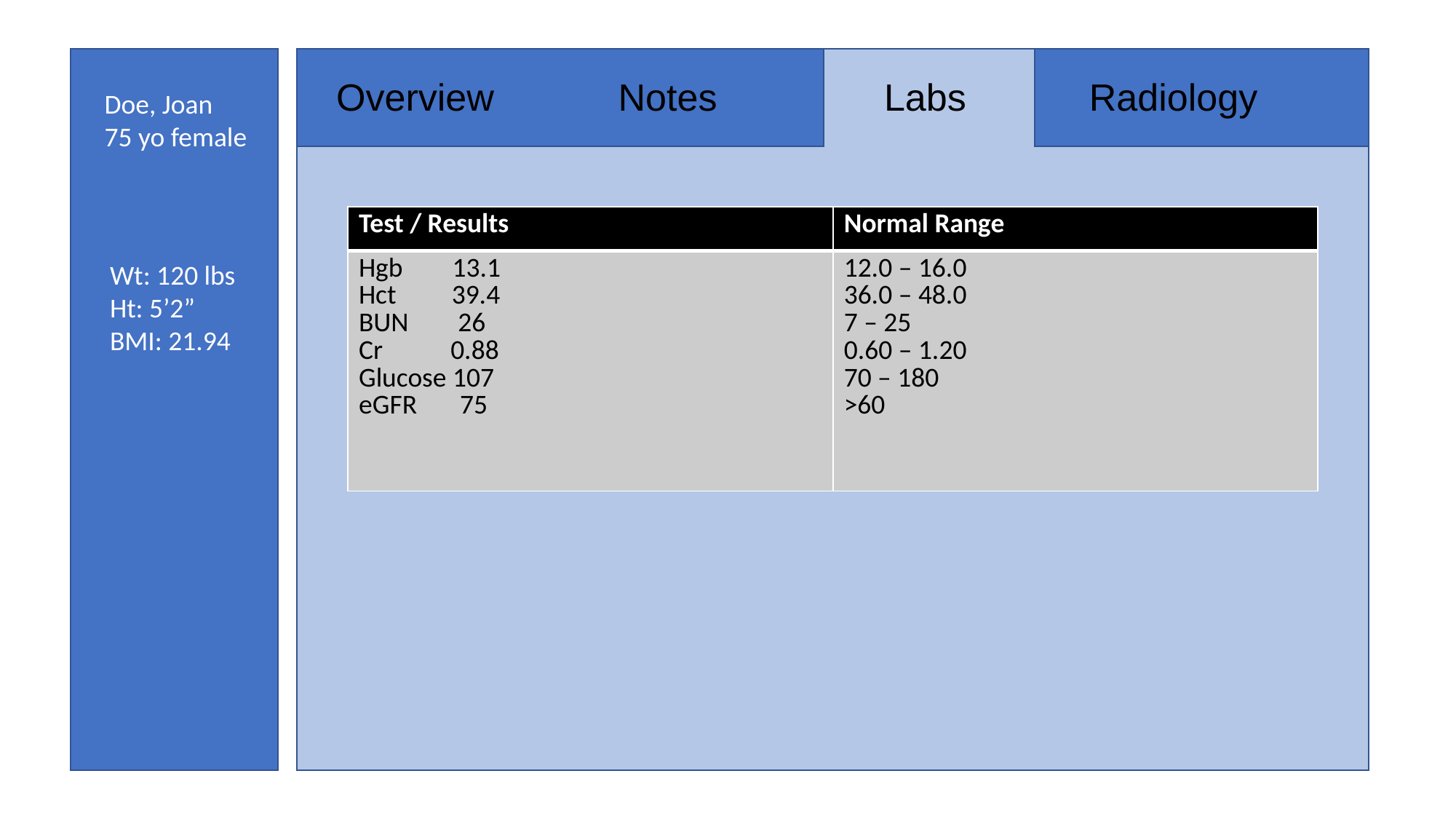

Overview
Notes
Labs
Radiology
Doe, Joan
75 yo female
| Test / Results | Normal Range |
| --- | --- |
| Hgb 13.1 Hct 39.4 BUN 26 Cr 0.88 Glucose 107 eGFR 75 | 12.0 – 16.0 36.0 – 48.0 7 – 25 0.60 – 1.20 70 – 180 >60 |
Wt: 120 lbs
Ht: 5’2”
BMI: 21.94

## Slide 44
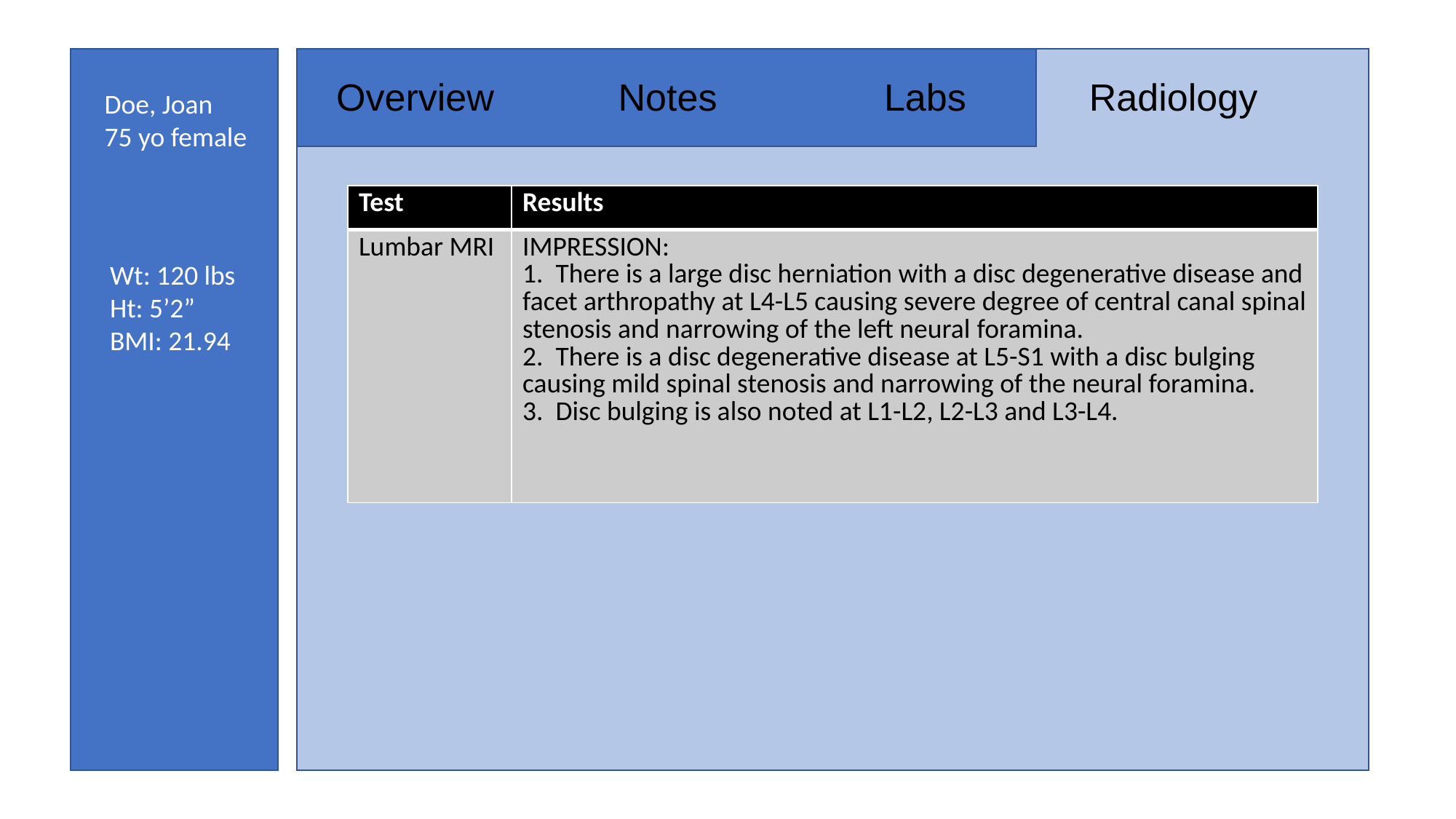

Overview
Notes
Labs
Radiology
Doe, Joan
75 yo female
| Test | Results |
| --- | --- |
| Lumbar MRI | IMPRESSION: 1.  There is a large disc herniation with a disc degenerative disease and facet arthropathy at L4-L5 causing severe degree of central canal spinal stenosis and narrowing of the left neural foramina. 2.  There is a disc degenerative disease at L5-S1 with a disc bulging causing mild spinal stenosis and narrowing of the neural foramina. 3.  Disc bulging is also noted at L1-L2, L2-L3 and L3-L4. |
Wt: 120 lbs
Ht: 5’2”
BMI: 21.94

## Slide 45
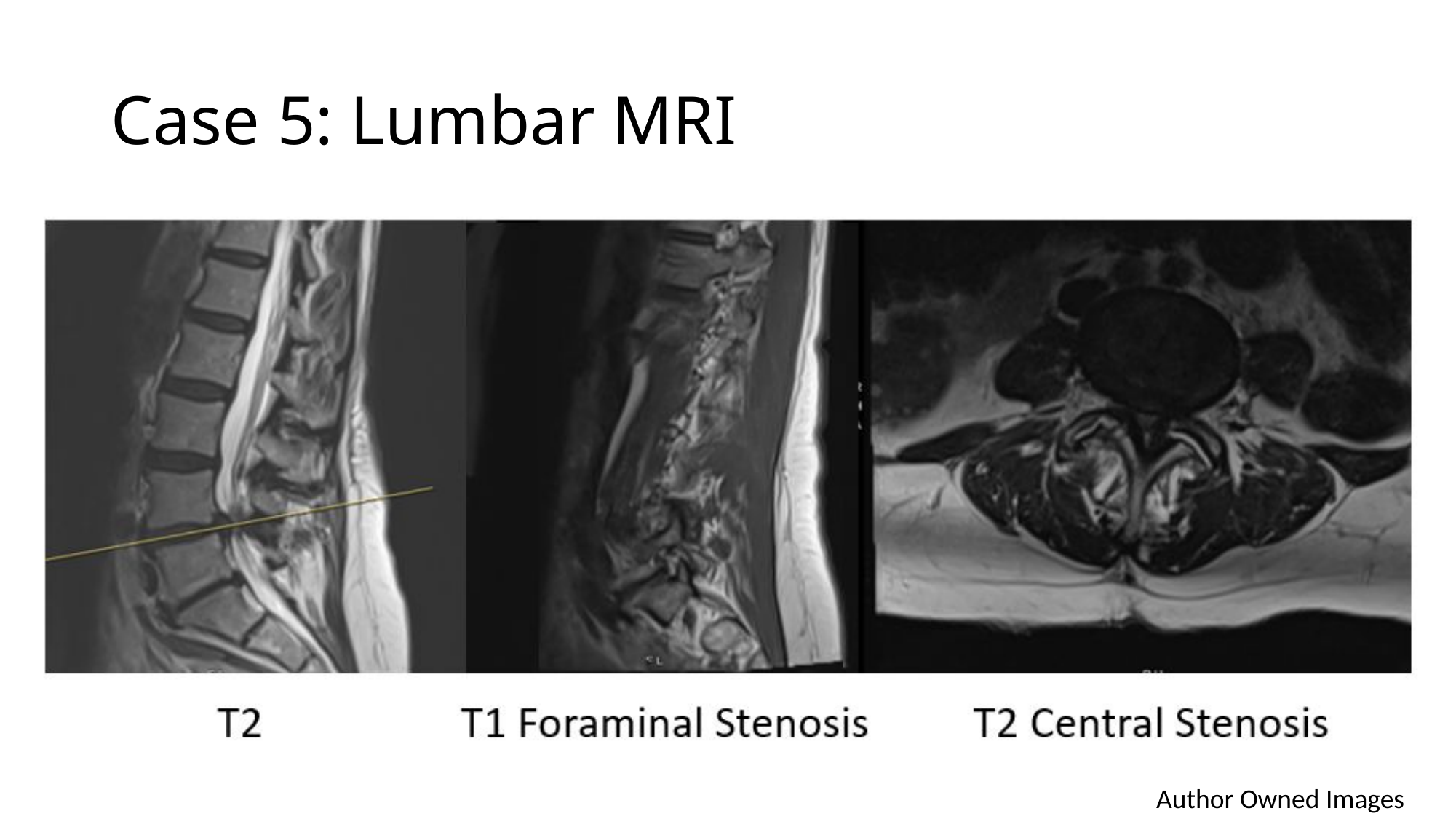

# Case 5: Lumbar MRI
Author Owned Images

## Slide 46
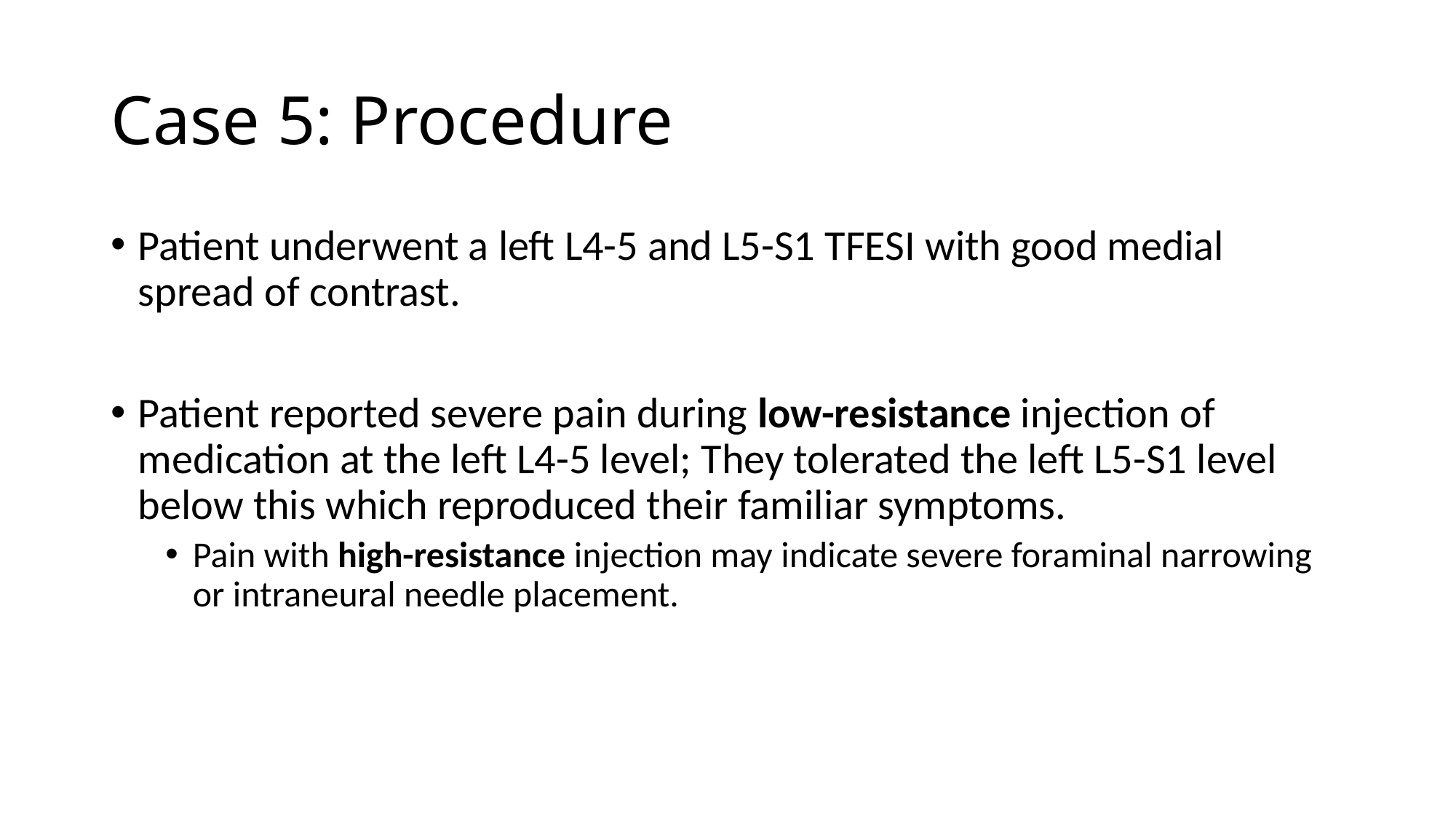

# Case 5: Procedure
Patient underwent a left L4-5 and L5-S1 TFESI with good medial spread of contrast.
Patient reported severe pain during low-resistance injection of medication at the left L4-5 level; They tolerated the left L5-S1 level below this which reproduced their familiar symptoms.
Pain with high-resistance injection may indicate severe foraminal narrowing or intraneural needle placement.

## Slide 47
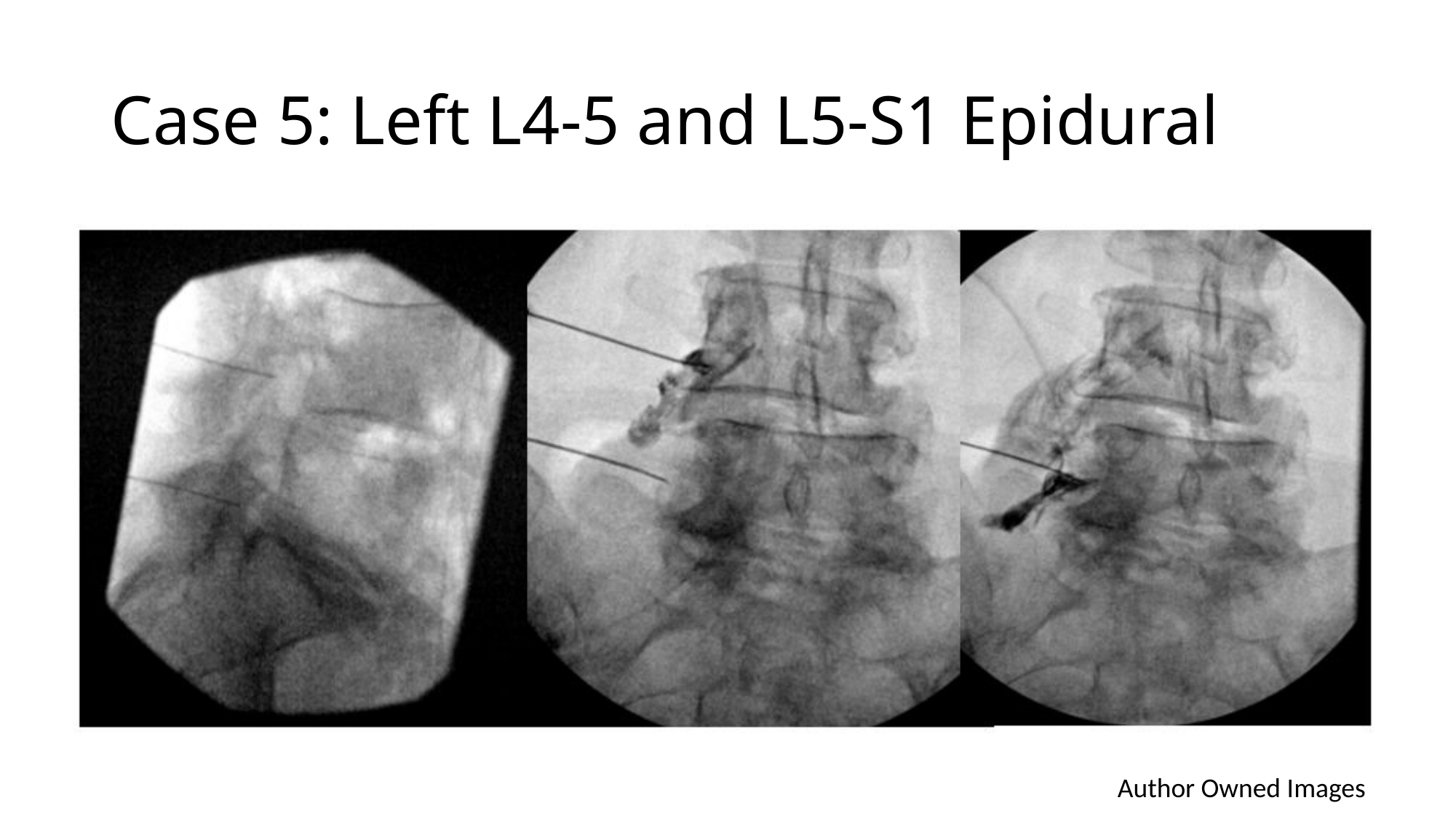

# Case 5: Left L4-5 and L5-S1 Epidural
Author Owned Images

## Slide 48
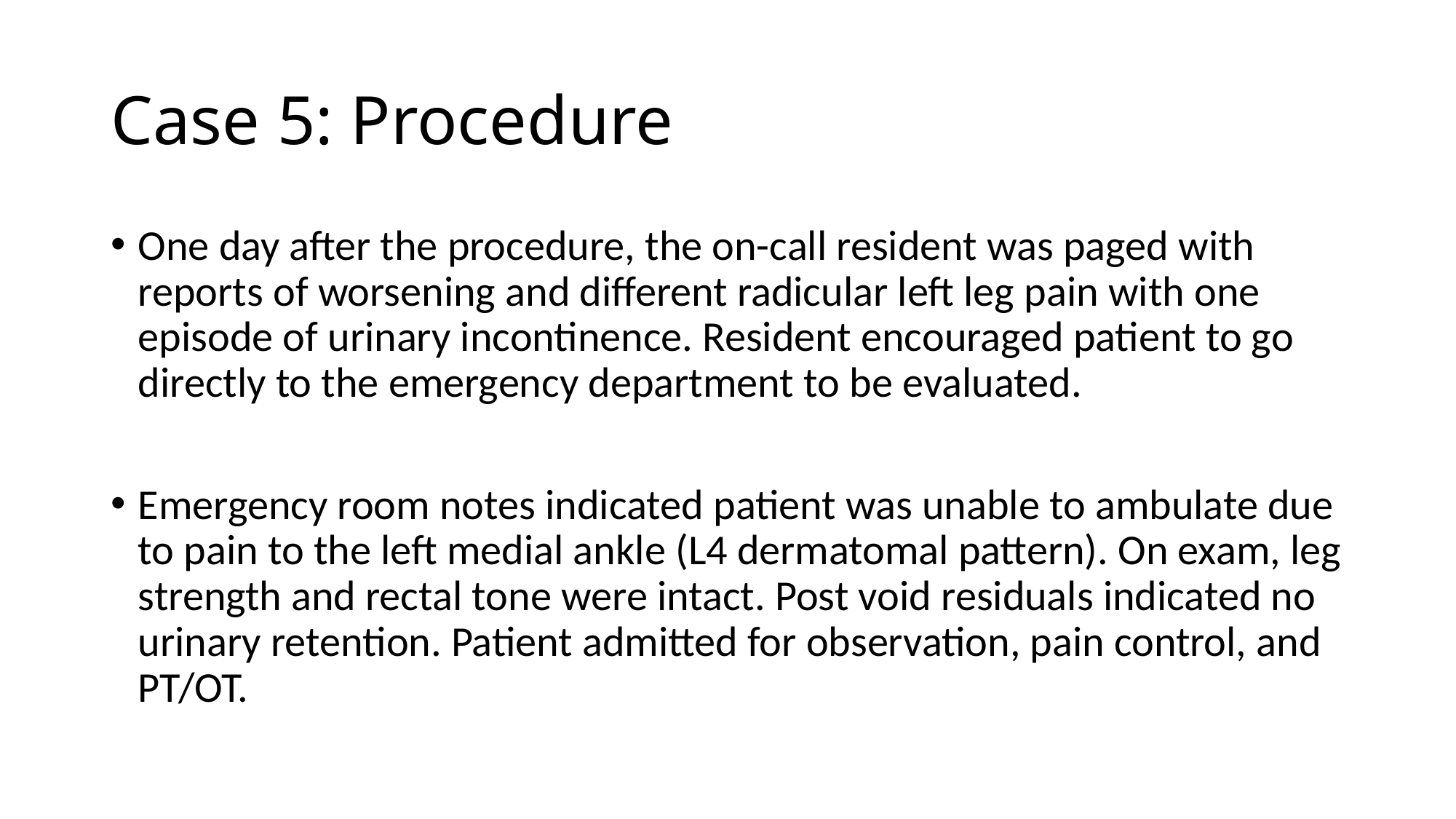

# Case 5: Procedure
One day after the procedure, the on-call resident was paged with reports of worsening and different radicular left leg pain with one episode of urinary incontinence. Resident encouraged patient to go directly to the emergency department to be evaluated.
Emergency room notes indicated patient was unable to ambulate due to pain to the left medial ankle (L4 dermatomal pattern). On exam, leg strength and rectal tone were intact. Post void residuals indicated no urinary retention. Patient admitted for observation, pain control, and PT/OT.

## Slide 49
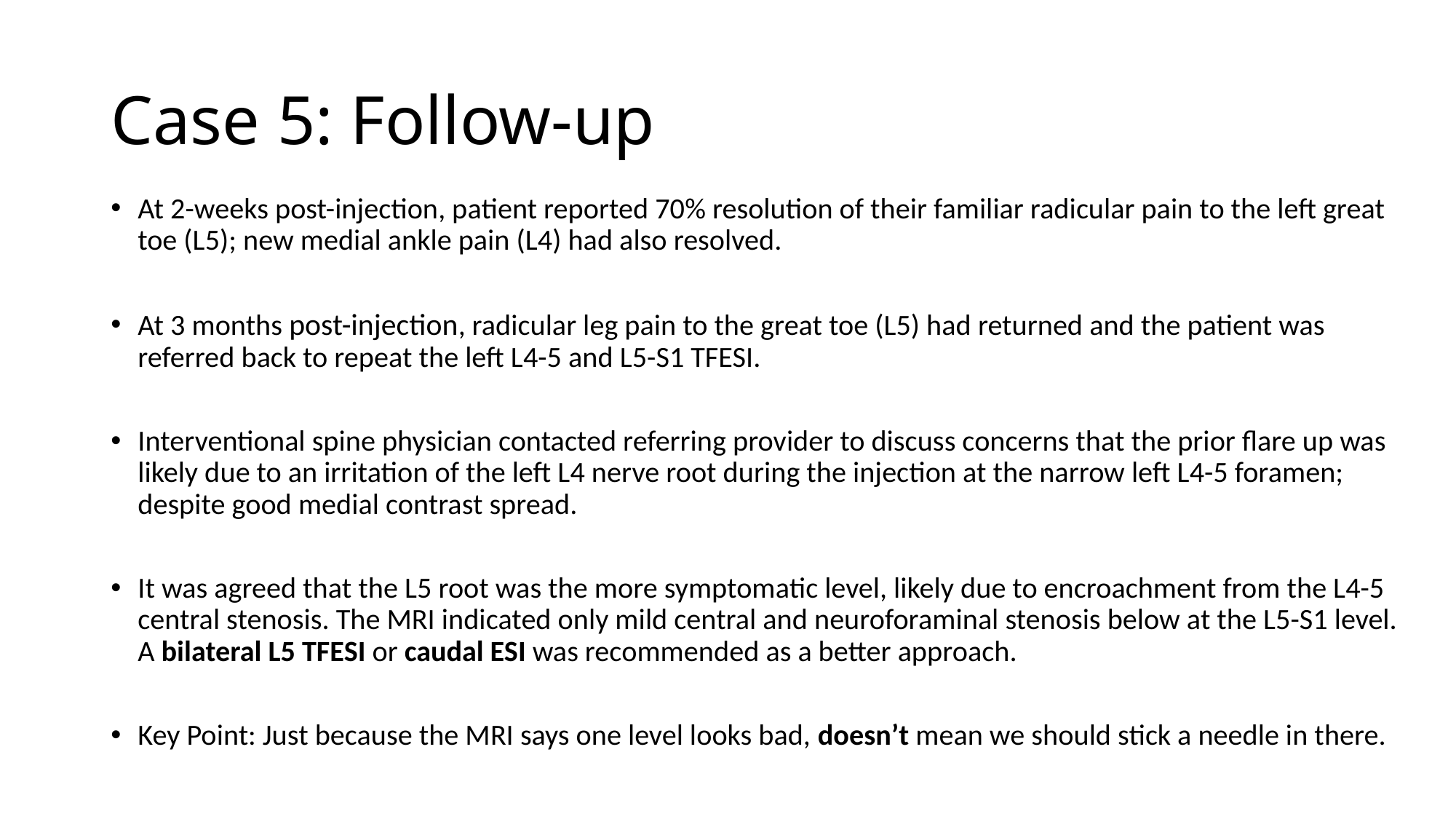

# Case 5: Follow-up
At 2-weeks post-injection, patient reported 70% resolution of their familiar radicular pain to the left great toe (L5); new medial ankle pain (L4) had also resolved.
At 3 months post-injection, radicular leg pain to the great toe (L5) had returned and the patient was referred back to repeat the left L4-5 and L5-S1 TFESI.
Interventional spine physician contacted referring provider to discuss concerns that the prior flare up was likely due to an irritation of the left L4 nerve root during the injection at the narrow left L4-5 foramen; despite good medial contrast spread.
It was agreed that the L5 root was the more symptomatic level, likely due to encroachment from the L4-5 central stenosis. The MRI indicated only mild central and neuroforaminal stenosis below at the L5-S1 level. A bilateral L5 TFESI or caudal ESI was recommended as a better approach.
Key Point: Just because the MRI says one level looks bad, doesn’t mean we should stick a needle in there.

## Slide 50
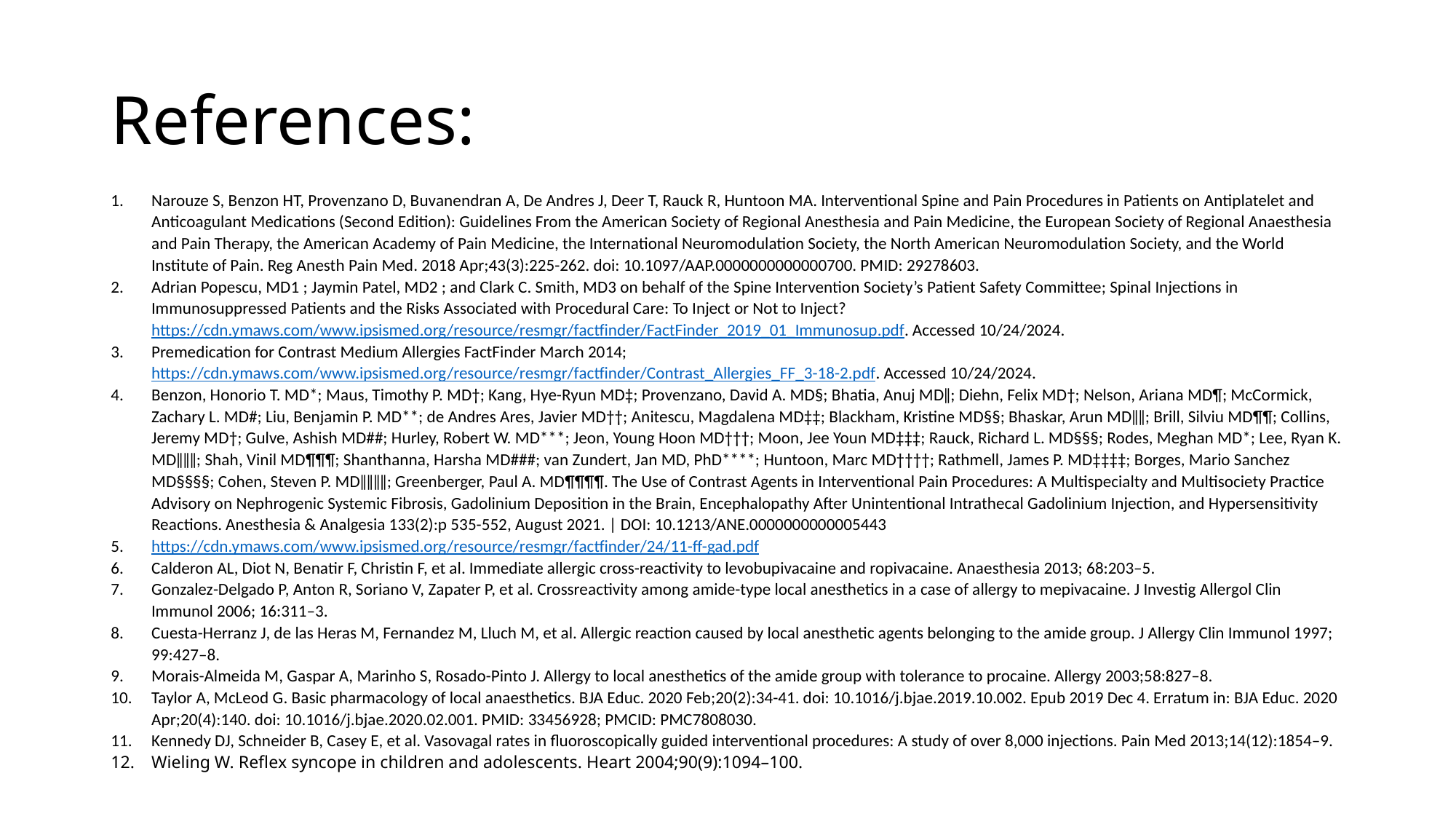

# References:
Narouze S, Benzon HT, Provenzano D, Buvanendran A, De Andres J, Deer T, Rauck R, Huntoon MA. Interventional Spine and Pain Procedures in Patients on Antiplatelet and Anticoagulant Medications (Second Edition): Guidelines From the American Society of Regional Anesthesia and Pain Medicine, the European Society of Regional Anaesthesia and Pain Therapy, the American Academy of Pain Medicine, the International Neuromodulation Society, the North American Neuromodulation Society, and the World Institute of Pain. Reg Anesth Pain Med. 2018 Apr;43(3):225-262. doi: 10.1097/AAP.0000000000000700. PMID: 29278603.
Adrian Popescu, MD1 ; Jaymin Patel, MD2 ; and Clark C. Smith, MD3 on behalf of the Spine Intervention Society’s Patient Safety Committee; Spinal Injections in Immunosuppressed Patients and the Risks Associated with Procedural Care: To Inject or Not to Inject? https://cdn.ymaws.com/www.ipsismed.org/resource/resmgr/factfinder/FactFinder_2019_01_Immunosup.pdf. Accessed 10/24/2024.
Premedication for Contrast Medium Allergies FactFinder March 2014; https://cdn.ymaws.com/www.ipsismed.org/resource/resmgr/factfinder/Contrast_Allergies_FF_3-18-2.pdf. Accessed 10/24/2024.
Benzon, Honorio T. MD*; Maus, Timothy P. MD†; Kang, Hye-Ryun MD‡; Provenzano, David A. MD§; Bhatia, Anuj MD‖; Diehn, Felix MD†; Nelson, Ariana MD¶; McCormick, Zachary L. MD#; Liu, Benjamin P. MD**; de Andres Ares, Javier MD††; Anitescu, Magdalena MD‡‡; Blackham, Kristine MD§§; Bhaskar, Arun MD‖‖; Brill, Silviu MD¶¶; Collins, Jeremy MD†; Gulve, Ashish MD##; Hurley, Robert W. MD***; Jeon, Young Hoon MD†††; Moon, Jee Youn MD‡‡‡; Rauck, Richard L. MD§§§; Rodes, Meghan MD*; Lee, Ryan K. MD‖‖‖; Shah, Vinil MD¶¶¶; Shanthanna, Harsha MD###; van Zundert, Jan MD, PhD****; Huntoon, Marc MD††††; Rathmell, James P. MD‡‡‡‡; Borges, Mario Sanchez MD§§§§; Cohen, Steven P. MD‖‖‖‖; Greenberger, Paul A. MD¶¶¶¶. The Use of Contrast Agents in Interventional Pain Procedures: A Multispecialty and Multisociety Practice Advisory on Nephrogenic Systemic Fibrosis, Gadolinium Deposition in the Brain, Encephalopathy After Unintentional Intrathecal Gadolinium Injection, and Hypersensitivity Reactions. Anesthesia & Analgesia 133(2):p 535-552, August 2021. | DOI: 10.1213/ANE.0000000000005443
https://cdn.ymaws.com/www.ipsismed.org/resource/resmgr/factfinder/24/11-ff-gad.pdf
Calderon AL, Diot N, Benatir F, Christin F, et al. Immediate allergic cross-reactivity to levobupivacaine and ropivacaine. Anaesthesia 2013; 68:203–5.
Gonzalez-Delgado P, Anton R, Soriano V, Zapater P, et al. Crossreactivity among amide-type local anesthetics in a case of allergy to mepivacaine. J Investig Allergol Clin Immunol 2006; 16:311–3.
Cuesta-Herranz J, de las Heras M, Fernandez M, Lluch M, et al. Allergic reaction caused by local anesthetic agents belonging to the amide group. J Allergy Clin Immunol 1997; 99:427–8.
Morais-Almeida M, Gaspar A, Marinho S, Rosado-Pinto J. Allergy to local anesthetics of the amide group with tolerance to procaine. Allergy 2003;58:827–8.
Taylor A, McLeod G. Basic pharmacology of local anaesthetics. BJA Educ. 2020 Feb;20(2):34-41. doi: 10.1016/j.bjae.2019.10.002. Epub 2019 Dec 4. Erratum in: BJA Educ. 2020 Apr;20(4):140. doi: 10.1016/j.bjae.2020.02.001. PMID: 33456928; PMCID: PMC7808030.
Kennedy DJ, Schneider B, Casey E, et al. Vasovagal rates in fluoroscopically guided interventional procedures: A study of over 8,000 injections. Pain Med 2013;14(12):1854–9.
Wieling W. Reflex syncope in children and adolescents. Heart 2004;90(9):1094–100.
