# Supplementary material for: Resident Interventional Spine Course with Didactics and Hands-On Skills Lab
Source: MedEdPORTAL. 2025 Oct 7;21:11551. doi: 10.15766/mep_2374-8265.11551 (PMC12502988; doi:10.15766/mep_2374-8265.11551)
Supplement: Supplementary file 1 — Overview - Spine.pptxPrep Kit Materials.docxBuilding a Low-Cost Spine Simulator.pptxFacilitators Guide.docxSpine Procedure - Guidelines Lecture.pptxSpine Procedure Guidelines Lecture Video.mp4Course Chart Review Guidelines.docxSpine Course - Cases.pptxChart Review Preprocedures Checklist.docxInformed Consent and Procedure Timeout Checklist.docxLumbar Procedure Table Checklist.docxProcedure Descriptions.docxFluoroscopic Spine Procedure Images.pptxSpine Course Pre-Post Survey - Updated.docxSpine Course Pre-Post Survey - Original.docx [file mep_2374-8265.11551-s001.zip › I. Chart Review Preprocedures Checklist.docx]

Chart Review Checklist Resident Name: Date of Test:

| **Review risks in the following categories:** | 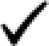 **If reviewed** |
| --- | --- |
| **Bleeding**   - Anticoagulation/Antiplatelet medication - Aspirin and non-aspirin NSAID utilization - Review images for vascular, neurologic, or other risks - Liver or renal disease - Personal or family history of bleeding disorder |  |
| **Infection**   - Recent illnesses or infection - Recent Antibiotics - Diabetes Mellitus - Immune compromised (malignancy, HIV/AIDS) - Immunosuppressive medication |  |
| **Allergy**   - Latex - Skin prep - Sedation - Local anesthetic - Contrast - Steroid - Adhesive |  |
| **Sedation**   - Prior History with sedation - Obstructive Sleep Apnea - Chronic Obstructive Pulmonary Disease - Coronary Artery Disease/Congestive Heart Failure - Opioid/Benzodiazepine use - Mental state |  |
| **Entrustment Scale** | **(1-5)** |
| 1. “I would have to do”—i.e., Requires complete hands-on guidance 2. “I had to talk them through”—i.e., Able to perform tasks but requires constant direction 3. “I had to prompt them from time to time”—i.e., Demonstrates some independence, but requires intermittent direction 4. “I would need to be in the room just in case”—i.e., Independence but unaware of risks 5. “I would not need to be there”—i.e., Complete independence, understands risks and performs safely |  |
| **Comments:** | |
