# Supplementary material for: Resident Interventional Spine Course with Didactics and Hands-On Skills Lab
Source: MedEdPORTAL. 2025 Oct 7;21:11551. doi: 10.15766/mep_2374-8265.11551 (PMC12502988; doi:10.15766/mep_2374-8265.11551)
Supplement: Supplementary file 1 — Overview - Spine.pptxPrep Kit Materials.docxBuilding a Low-Cost Spine Simulator.pptxFacilitators Guide.docxSpine Procedure - Guidelines Lecture.pptxSpine Procedure Guidelines Lecture Video.mp4Course Chart Review Guidelines.docxSpine Course - Cases.pptxChart Review Preprocedures Checklist.docxInformed Consent and Procedure Timeout Checklist.docxLumbar Procedure Table Checklist.docxProcedure Descriptions.docxFluoroscopic Spine Procedure Images.pptxSpine Course Pre-Post Survey - Updated.docxSpine Course Pre-Post Survey - Original.docx [file mep_2374-8265.11551-s001.zip › J. Informed Consent and Procedure Timeout Checklist.docx]

Resident Name: Date of test:

|  | **Performance Steps** | 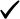**If done**  **correctly** |
| --- | --- | --- |
| **1.** | **Confirm patient full name and date of birth** |  |
| **2.** | **Confirm with the patient what procedure is planned** |  |
| **3.** | **Review risks of interventional spine procedure:** |  |
|  | **Bleeding**  - Confirm whether patient is taking blood thinning medication and when last dose was taken |  |
|  | **Infection**   - Introducing needle through skin poses infection risk, we take special care to clean the area - Has the patient been ill or on antibiotics in the past two weeks? |  |
|  | **Allergies**  - Confirm whether allergies to chlorhexidine, iodine contrast, lidocaine, steroids, adhesives, latex, or any others |  |
|  | **Structures**  - Introducing needle through skin and multiple tissue planes poses risk to damaging surrounding structures (fluoroscopic imaging guidance minimizes this risk) |  |
| **4.** | **Confirm whether sedation will be used during procedure**   - **If YES:**   - Cardiac and Respiratory exam   - Mallampati score |  |
| **5.** | **Confirm laterality of the procedure**  - **MUST** initial appropriate side, both if bilateral |  |
| **6.** | **Obtain patient signature that risks have been reviewed**  **and they would like to proceed with the procedure**  - Answer any questions the patient may have |  |
| **Entrustment Scale** | | **(1-5)** |
| 1. “I would have to do”—i.e., Requires complete hands-on guidance 2. “I had to talk them through”—i.e., Able to perform tasks but requires constant direction 3. “I had to prompt them from time to time”—i.e., Demonstrates some independence, but requires intermittent direction 4. “I would need to be in the room just in case”—i.e., Independence but unaware of risks 5. “I would not need to be there”—i.e., Complete independence, understands risks and performs safely | |  |
| **Comments:** | | |

Resident Name: Date of test:

|  | **Performance Steps** | 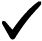 **If done correctly** |
| --- | --- | --- |
| **1.** | **Time-out.**  - All staff stop, including the patient |  |
| **2.** | **Confirm patient’s full name and date of birth** |  |
| **3.** | **Confirm the procedure the patient will be receiving** |  |
| **4.** | **Confirm the location(s) and side(s) of the procedure.**  - Confirm site(s) are marked with provider initials |  |
| **5.** | **Discuss patient allergies to chlorhexidine, iodine contrast,**  **lidocaine, steroids, adhesives, latex, or any others?**  - Adjust based on procedure requirements |  |
| **6.** | **There is low risk of fire from with this procedure**  - Possible heat source (RFA?); oxygen source (nasal canula O_2_?) |  |
| **7.** | **Additional considerations include:**  - Confirm driver, sedation, not pregnant, etc. Adjust  based on procedure requirements |  |
| **8.** | **Does the patient or team have any concerns before the**  **procedure begins?** |  |
| **Entrustment Scale** | | **(1-5)** |
| 1. “I would have to do”—i.e., Requires complete hands-on guidance 2. “I had to talk them through”—i.e., Able to perform tasks but requires constant direction 3. “I had to prompt them from time to time”—i.e., Demonstrates some independence, but requires intermittent direction 4. “I would need to be in the room just in case”—i.e., Independence but unaware of risks 5. “I would not need to be there”—i.e., Complete independence, understands risks and performs safely | |  |
| **Comments:** | | |
