# Supplementary material for: Resident Interventional Spine Course with Didactics and Hands-On Skills Lab
Source: MedEdPORTAL. 2025 Oct 7;21:11551. doi: 10.15766/mep_2374-8265.11551 (PMC12502988; doi:10.15766/mep_2374-8265.11551)
Supplement: Supplementary file 1 — Overview - Spine.pptxPrep Kit Materials.docxBuilding a Low-Cost Spine Simulator.pptxFacilitators Guide.docxSpine Procedure - Guidelines Lecture.pptxSpine Procedure Guidelines Lecture Video.mp4Course Chart Review Guidelines.docxSpine Course - Cases.pptxChart Review Preprocedures Checklist.docxInformed Consent and Procedure Timeout Checklist.docxLumbar Procedure Table Checklist.docxProcedure Descriptions.docxFluoroscopic Spine Procedure Images.pptxSpine Course Pre-Post Survey - Updated.docxSpine Course Pre-Post Survey - Original.docx [file mep_2374-8265.11551-s001.zip › K. Lumbar Procedure Table Checklist.docx]

| **Procedure** | **Type of needle** | **Fluoroscopic C-arm positioning** |
| --- | --- | --- |
| **Lumbar TFESI** | 22- or 25-gauge Quincke spinal needle | Cephalad or caudal tilt to line up the superior end plate  Ipsilateral oblique to visualize the “Scotty Dog” |
| **Lumbar ILESI** | 18- or 20-gauge Tuohy needle  Loss of resistance syringe | Cephalad or caudal tilt to open the interlaminar space  Slight ipsilateral oblique for paramedian approach |
| **Lumbar MBB** | 25-gauge Quincke spinal needle | Cephalad or caudal tilt to line up the superior end plate  Ipsilateral oblique to visualize the junction of the superior articular process & the transverse process  Alternate: Anterior/Posterior |
| **Lumbar RFA** | 18- or 20-guage RFA needle | Cephalad or caudal tilt to line up the superior end plate (SEP)  Then, caudal tilt and oblique to optimize inferior view of the junction of the SAP and the TP |
| **SI Joint Steroid Injection** | 22-gauge Quincke spinal needle | Cephalad tilt to square off S1  Contralateral oblique to view inferior SI access  Alternate: Caudal tilt |

Abbreviations: TFESI- Transforaminal Epidural Steroid Injections. ILESI- Interlaminar Epidural Steroid Injection. MBB- Medial Branch Block. RFA- Radiofrequency Ablation. SI- Sacroiliac.

*Above are quick references for simulating fluoroscopic guided spinal procedures. Preferences may vary amongst interventional spine physicians.
