# Supplementary material for: Resident Interventional Spine Course with Didactics and Hands-On Skills Lab
Source: MedEdPORTAL. 2025 Oct 7;21:11551. doi: 10.15766/mep_2374-8265.11551 (PMC12502988; doi:10.15766/mep_2374-8265.11551)
Supplement: Supplementary file 1 — Overview - Spine.pptxPrep Kit Materials.docxBuilding a Low-Cost Spine Simulator.pptxFacilitators Guide.docxSpine Procedure - Guidelines Lecture.pptxSpine Procedure Guidelines Lecture Video.mp4Course Chart Review Guidelines.docxSpine Course - Cases.pptxChart Review Preprocedures Checklist.docxInformed Consent and Procedure Timeout Checklist.docxLumbar Procedure Table Checklist.docxProcedure Descriptions.docxFluoroscopic Spine Procedure Images.pptxSpine Course Pre-Post Survey - Updated.docxSpine Course Pre-Post Survey - Original.docx [file mep_2374-8265.11551-s001.zip › L. Procedure Descriptions.docx]

**Overview of Fluoroscopic Guided Spine Procedures**

**Interlaminar Epidural Steroid Injection**
Interventional pain and spine providers utilize fluoroscopic guidance to ensure accurate needle placement and minimize the risk of inadvertent intravascular, subdural, or intraneural injection. A midline or paramedian approach with an 18- or 20-gauge (G) 3.5-inch Touhy needle is traditionally used for interlaminar epidural steroid injections. After a local anesthetic is applied, the needle is inserted and advanced towards the target. When close to the ligamentum flavum, a glass or plastic loss of resistance (LOR) syringe is attached. The needle is then slowly advanced with repeated checks for LOR. Once the ligamentum flavum is breached, there will be a distinct “pop” felt with LOR indicating entry into the epidural space. Contrast is then injected under live fluoroscopy to confirm flow in the posterior epidural space. Once the placement is verified, a mixture of corticosteroid (dexamethasone), normal saline, with (or without) local anesthetic is injected slowly.

**Lumbar Transforaminal Epidural Steroid Injection**
Lumbar transforaminal epidural steroid injections (TFESI) are used to alleviate neuropathic pain from irritated spinal nerve roots. With fluoroscopic imaging, oblique views allow for visualization of the classic “Scotty dog” view. The target is generally under the “chin of the dog” in the intervertebral foramen for the traditional subpedicular technique. Depending on patient anatomy, however, a retroneural or infraneural approach may be considered for better access to the epidural space. Regardless of the approach, images should be taken at multiple angles to ensure the needle is safely away from the spinal root. Once in position, contrast is injected under live fluoroscopy to confirm flow along the nerve root in the epidural space. Once the placement is verified, a mixture of corticosteroid (dexamethasone as first line) and local anesthetic is injected slowly.

**Medial Bundle Branch Block and Lumbar Radiofrequency Ablation**
Radiofrequency ablation (RFA) is used in the treatment of facet-mediated back pain by ablating (heat lesion) the sensory medial bundle branch (MBB) nerves that transmit signal from the symptomatic joints. Prior to considering RFA, diagnostic MBB blocks are used to ensure that the patient is a good candidate.

The lumbar facet joints are innervated by the medial branches of two separate spinal nerve root dorsal rami; one at the level of the facet and the other from the level above. For example, the L3-4 facet joint is supplied by the L2 and L3 dorsal ramus medial branches. These medial branches lie near the junction of the superior articular process (SAP) and the transverse process (TP). Using fluoroscopic imaging, a slight oblique image allows for visualization of this junction in the classic “Scotty dog” view. The medial branches generally lay over the eye of the dog”. Once the needle is in place, a small amount of contrast is used to confirm spread at the junction of the SAP and the TP. When spread is appropriate, a small volume (0.5 ml) of local anesthetic is injected. Patients are then asked to complete a pain diary to assess their response to the diagnostic block. Typically, two positive medial branch blocks are required before the patient is considered a good candidate for an RFA.

The fluoroscopic approach for the RFA is similar to the MBB approach; however, many providers recommend starting with more of a caudal (foot) tilt to place the RFA needle tip more parallel to the medial branches, and thus, a more proximal ablation to the nerves. This would be opposed to a perpendicular angle to the nerve with a more anterior-posterior approach. Needle placement is confirmed using fluoroscopic imaging in both antero-posterior and lateral views, which ensures the needle tip does not encroach upon the neural foramen, and therefore, avoids damaging the exiting spinal nerve root. Next, stimulation testing is conducted to ensure the RFA needle is near the intended medial branches, but not in close proximity to the more anterior spinal nerve root. After an additional local anesthetic is applied, the ablation is started typically at least 60 to 80°C for at least 60 to 90 seconds. After a small needle adjustment to increase the size of the ablation, a second ablation is typically done.

**Sacroiliac Joint Steroid Injection** Sacroiliac joint (SIJ) pain is often in the lower lateral back or gluteal area; however, it has been known to refer to the groin or down the leg. Accessing the SIJ even with fluoroscopic guidance can be technically challenging due to its irregular articular surface of the joint. The most common approach for an intra-articular injection is to target the lower third of the joint. To start, it is recommended to square off the S1 vertebra. Then, a slight contralateral oblique view will often help open the view of the lower SIJ space by aligning the posterior and anterior joint lines. Depending on the patient's anatomy, a caudal tilt may be helpful to visualize access to the SIJ space. After local anesthetic, the needle is advanced down to the joint space. A lateral view is used to confirm the needle depth and placement within the joint. Once placement appears appropriate, a small amount of contrast is injected to further confirm intraarticular spread. Finally, a small amount of steroid and local anesthetic (2 ml total) is injected.

**Resources:**

1. Bogduk N. *International Spine Intervention Society: Practice Guidelines for Spinal Diagnostic and Treatment Procedures*. Second Edition. International Spine Intervention Society; 2014.
