# Supplementary material for: Resident Interventional Spine Course with Didactics and Hands-On Skills Lab
Source: MedEdPORTAL. 2025 Oct 7;21:11551. doi: 10.15766/mep_2374-8265.11551 (PMC12502988; doi:10.15766/mep_2374-8265.11551)
Supplement: Supplementary file 1 — Overview - Spine.pptxPrep Kit Materials.docxBuilding a Low-Cost Spine Simulator.pptxFacilitators Guide.docxSpine Procedure - Guidelines Lecture.pptxSpine Procedure Guidelines Lecture Video.mp4Course Chart Review Guidelines.docxSpine Course - Cases.pptxChart Review Preprocedures Checklist.docxInformed Consent and Procedure Timeout Checklist.docxLumbar Procedure Table Checklist.docxProcedure Descriptions.docxFluoroscopic Spine Procedure Images.pptxSpine Course Pre-Post Survey - Updated.docxSpine Course Pre-Post Survey - Original.docx [file mep_2374-8265.11551-s001.zip › M. Fluoroscopic Spine Procedure Images.pptx]

## Slide 1
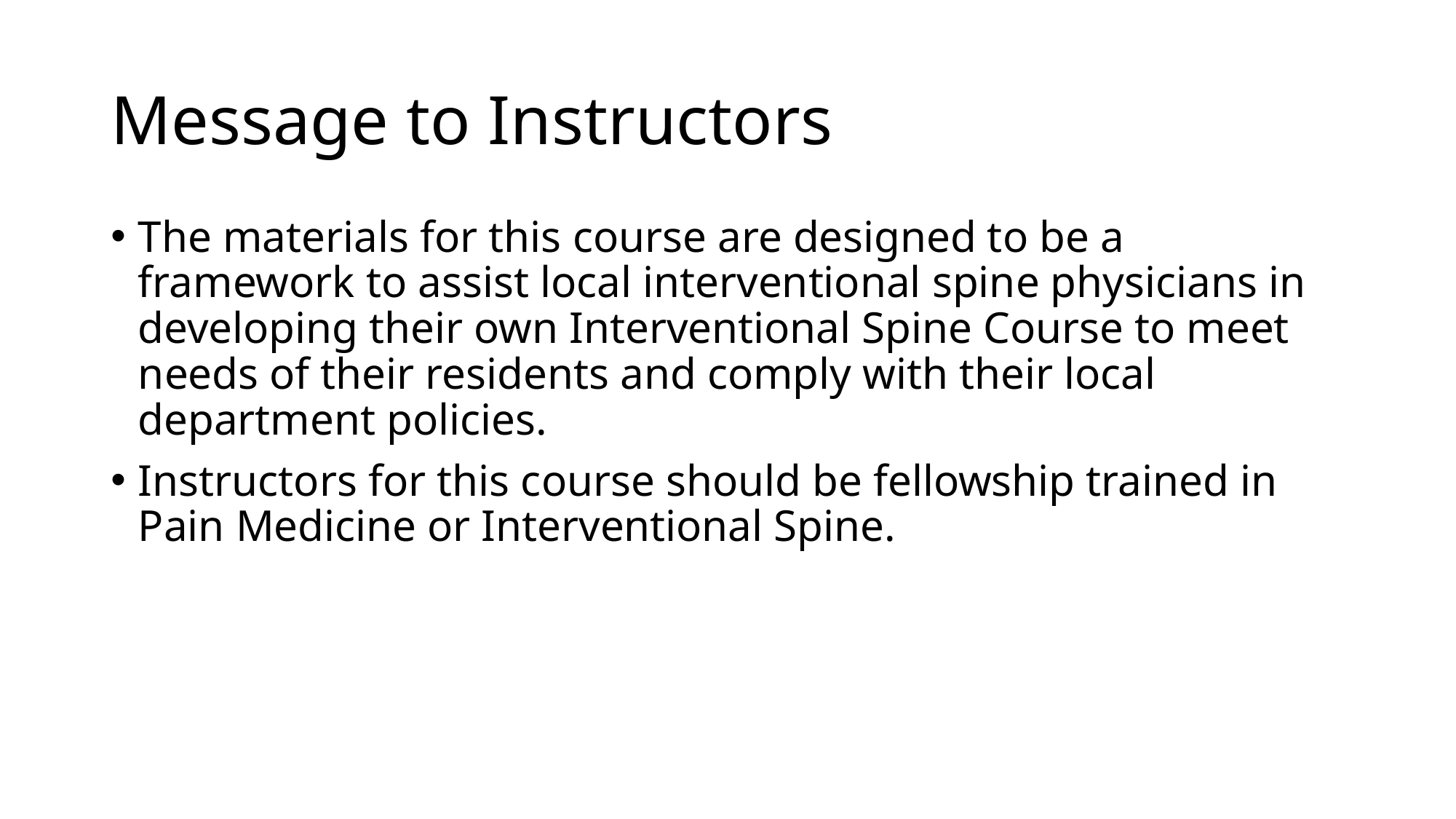

# Message to Instructors
The materials for this course are designed to be a framework to assist local interventional spine physicians in developing their own Interventional Spine Course to meet needs of their residents and comply with their local department policies.
Instructors for this course should be fellowship trained in Pain Medicine or Interventional Spine.

## Slide 2
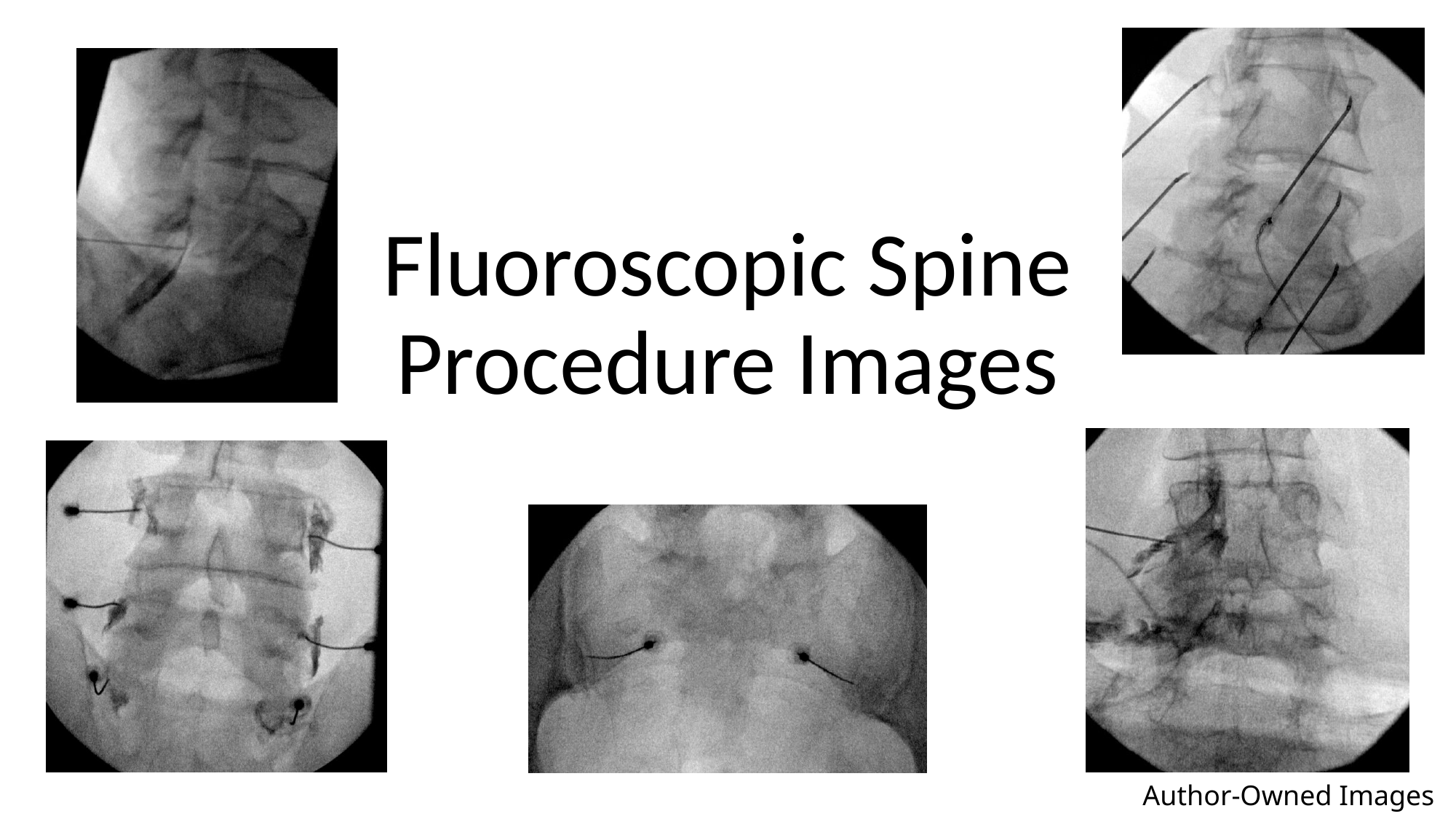

# Fluoroscopic Spine Procedure Images
Author-Owned Images

## Slide 3
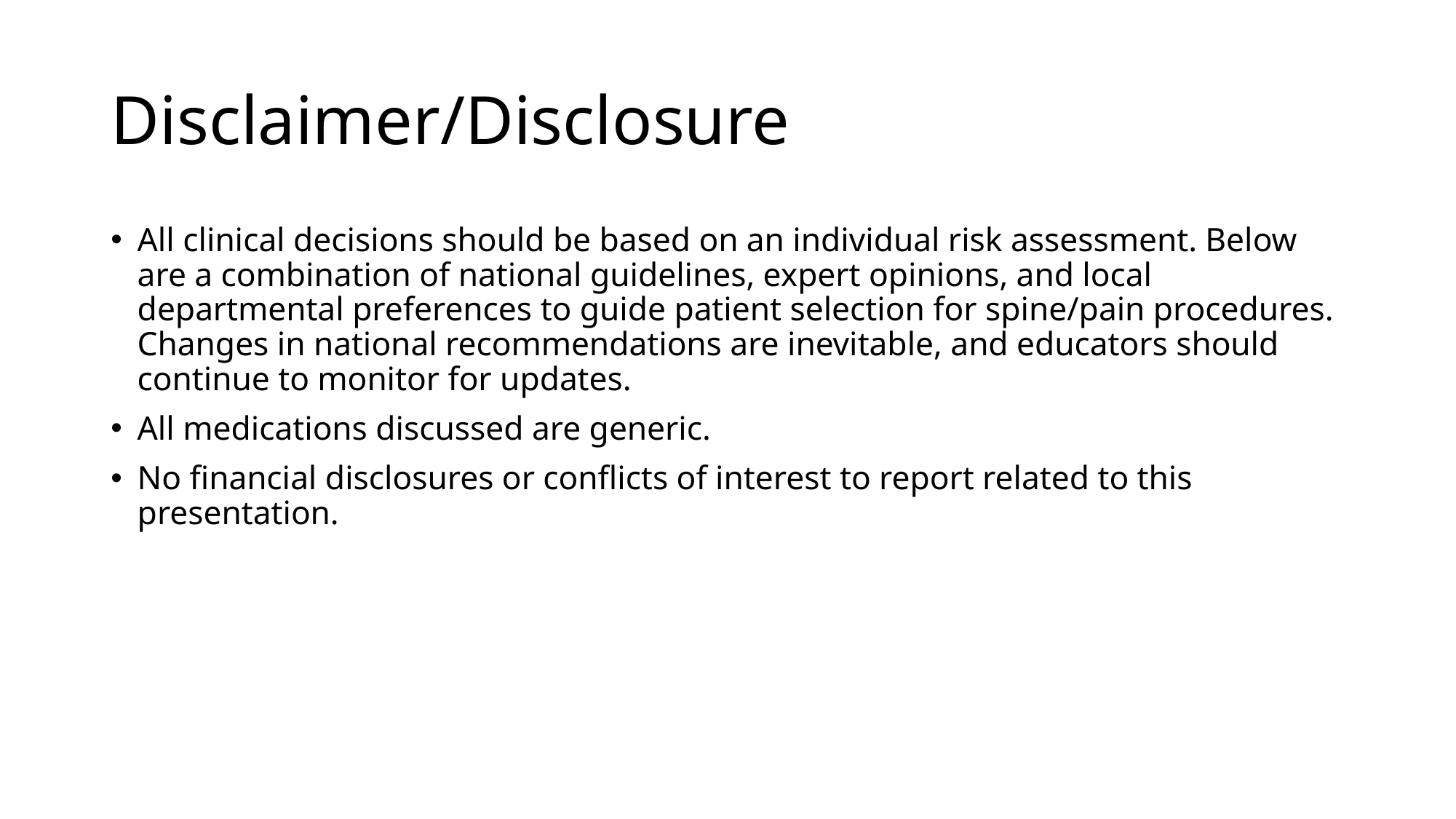

# Disclaimer/Disclosure
All clinical decisions should be based on an individual risk assessment. Below are a combination of national guidelines, expert opinions, and local departmental preferences to guide patient selection for spine/pain procedures. Changes in national recommendations are inevitable, and educators should continue to monitor for updates.
All medications discussed are generic.
No financial disclosures or conflicts of interest to report related to this presentation.

## Slide 4
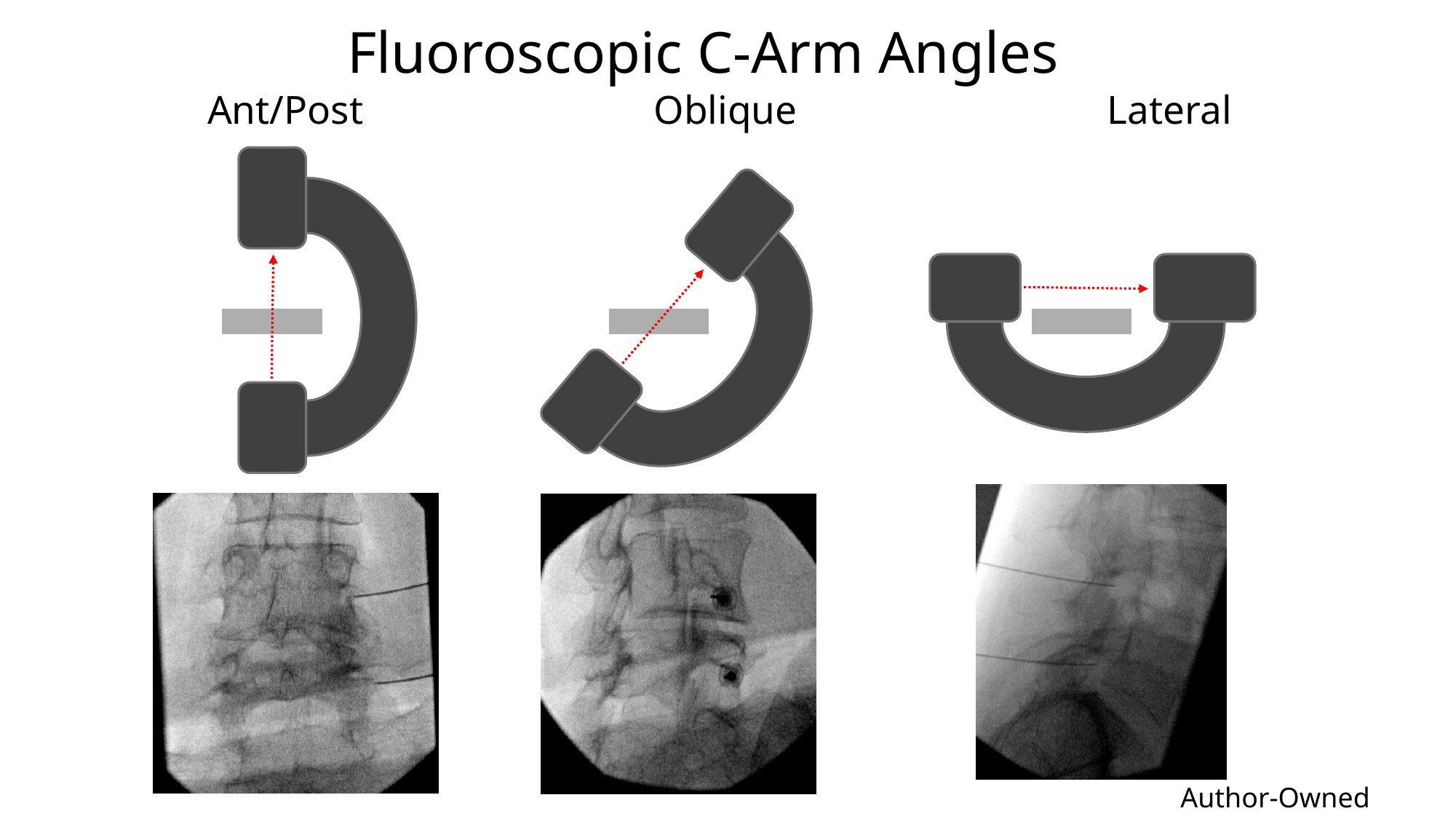

# Fluoroscopic C-Arm Angles Ant/Post Oblique Lateral
Author-Owned Images

## Slide 5
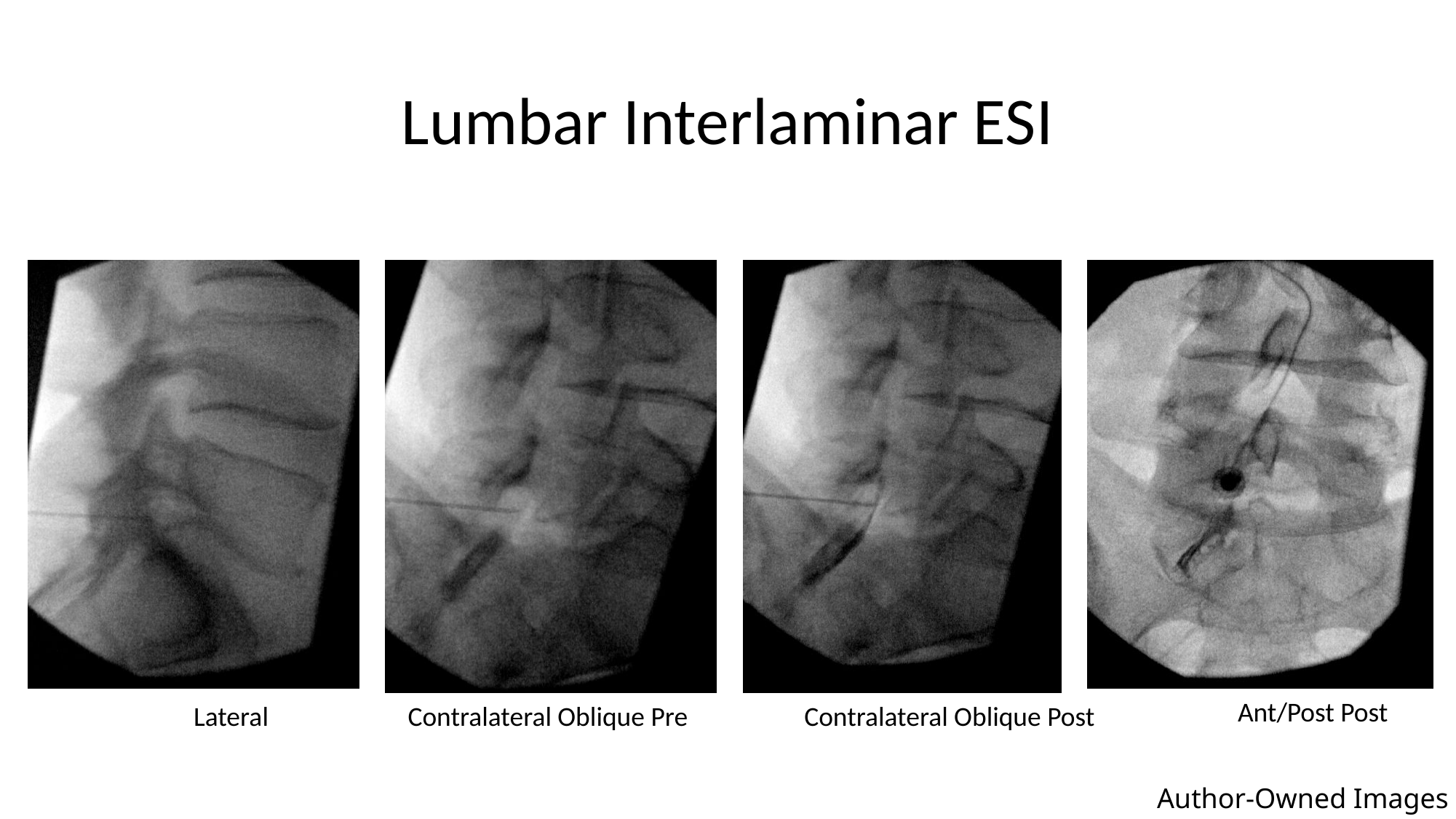

Oblique left Lateral Pre Lateral Post Ant/Post Pre Ant/Post Post L5 Ant/Post Post L4
Oblique left Lateral Pre Lateral Post Ant/Post Pre Ant/Post Post L5 Ant/Post Post L4
Oblique left Lateral Pre Lateral Post Ant/Post Pre Ant/Post Post L5 Ant/Post Post L4
# Lumbar Interlaminar ESI
 Ant/Post Post
 Lateral
Contralateral Oblique Pre
   Contralateral Oblique Post
Author-Owned Images

## Slide 6
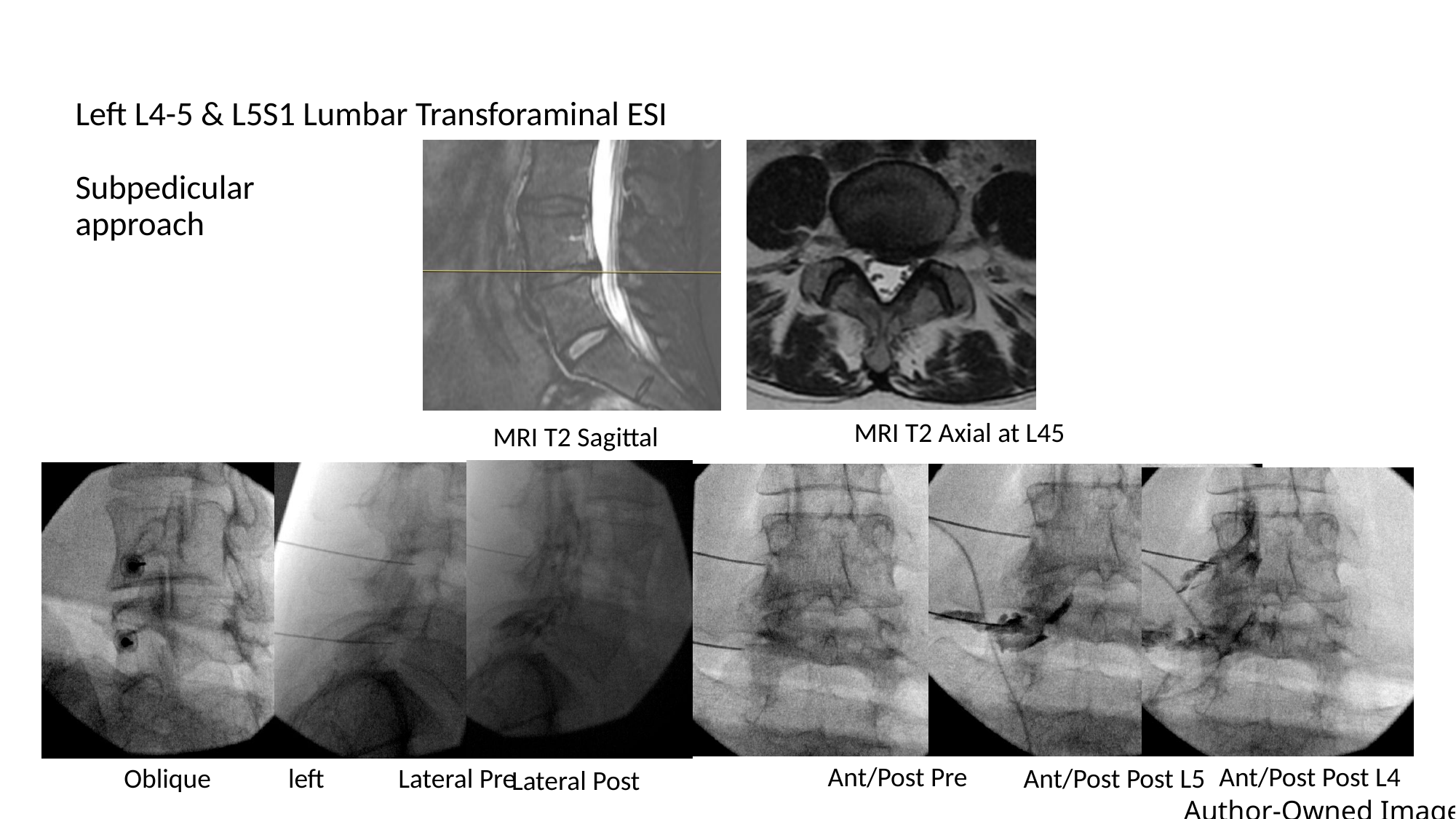

# Left L4-5 & L5S1 Lumbar Transforaminal ESI Subpedicular approach
 MRI T2 Axial at L45
MRI T2 Sagittal
 Ant/Post Pre
 Ant/Post Post L4
left Lateral Pre
Oblique
 Ant/Post Post L5
Lateral Post
Author-Owned Images

## Slide 7
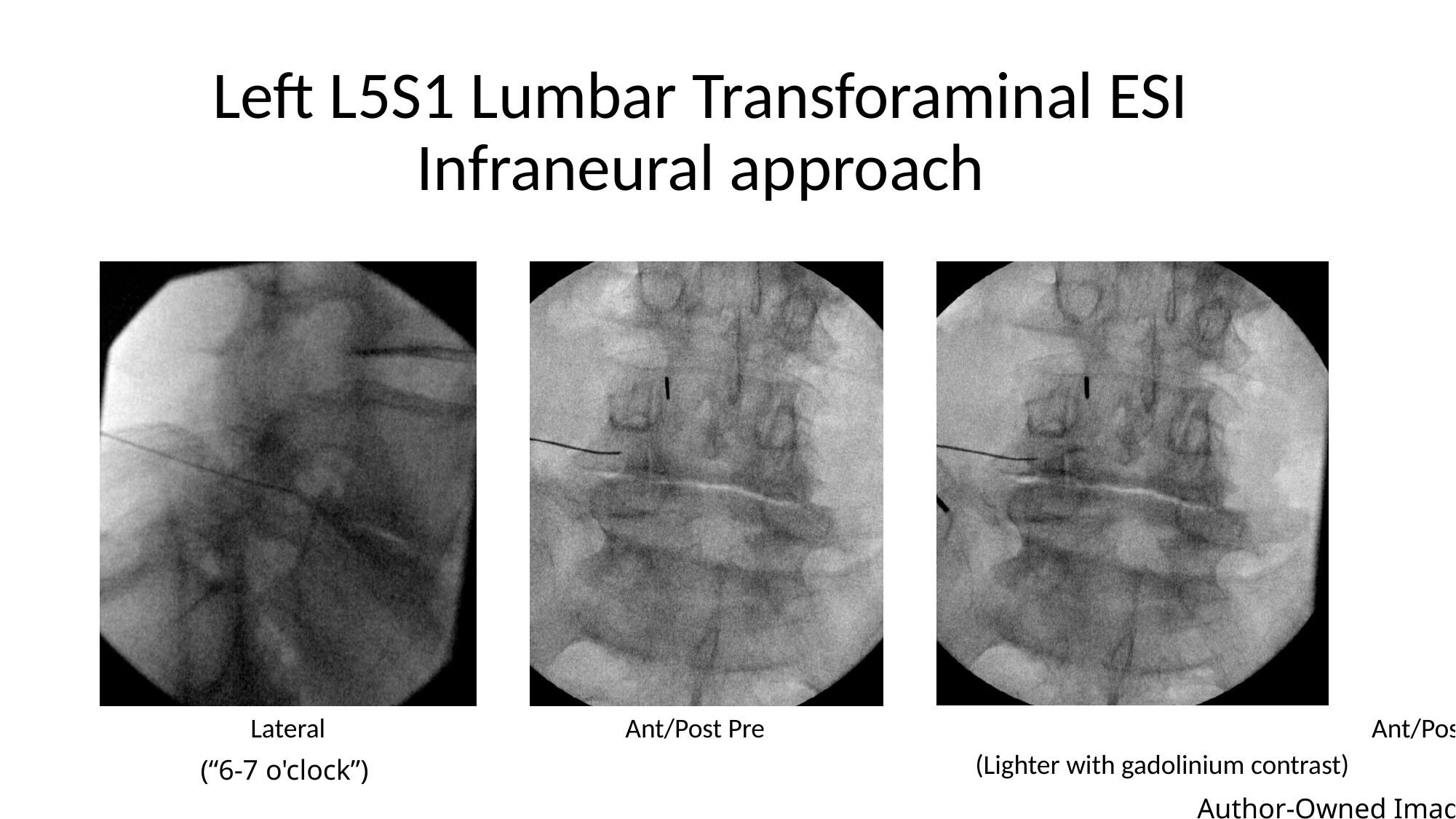

# Left L5S1 Lumbar Transforaminal ESI Infraneural approach
Lateral
Ant/Post Pre
 Ant/Post Post
  (Lighter with gadolinium contrast)
(“6-7 o'clock”)
Author-Owned Images

## Slide 8
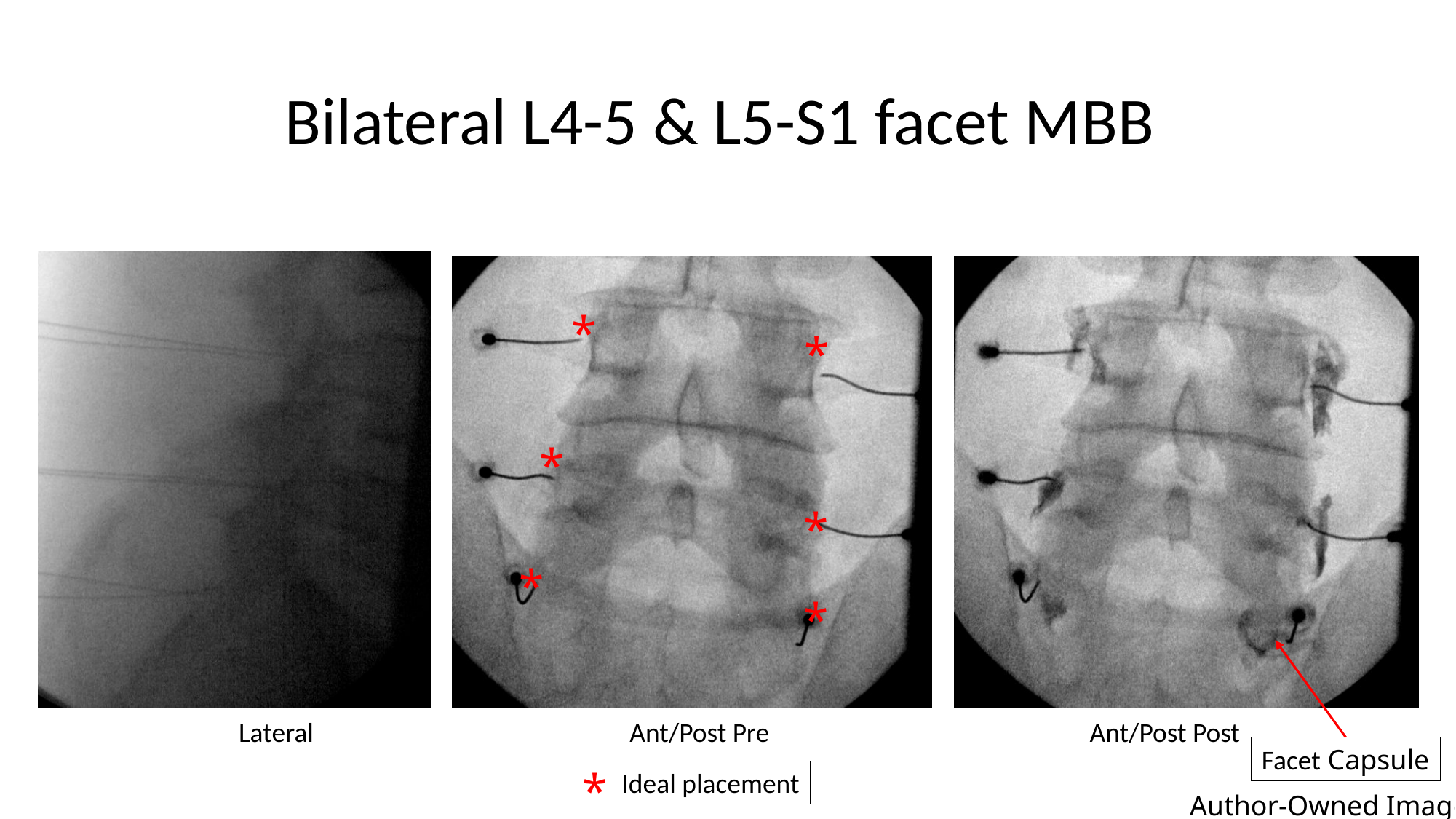

# Bilateral L4-5 & L5-S1 facet MBB
*
*
*
*
*
*
Ant/Post Pre
 Lateral
 Ant/Post Post
Facet Capsule
*
 Ideal placement
Author-Owned Images

## Slide 9
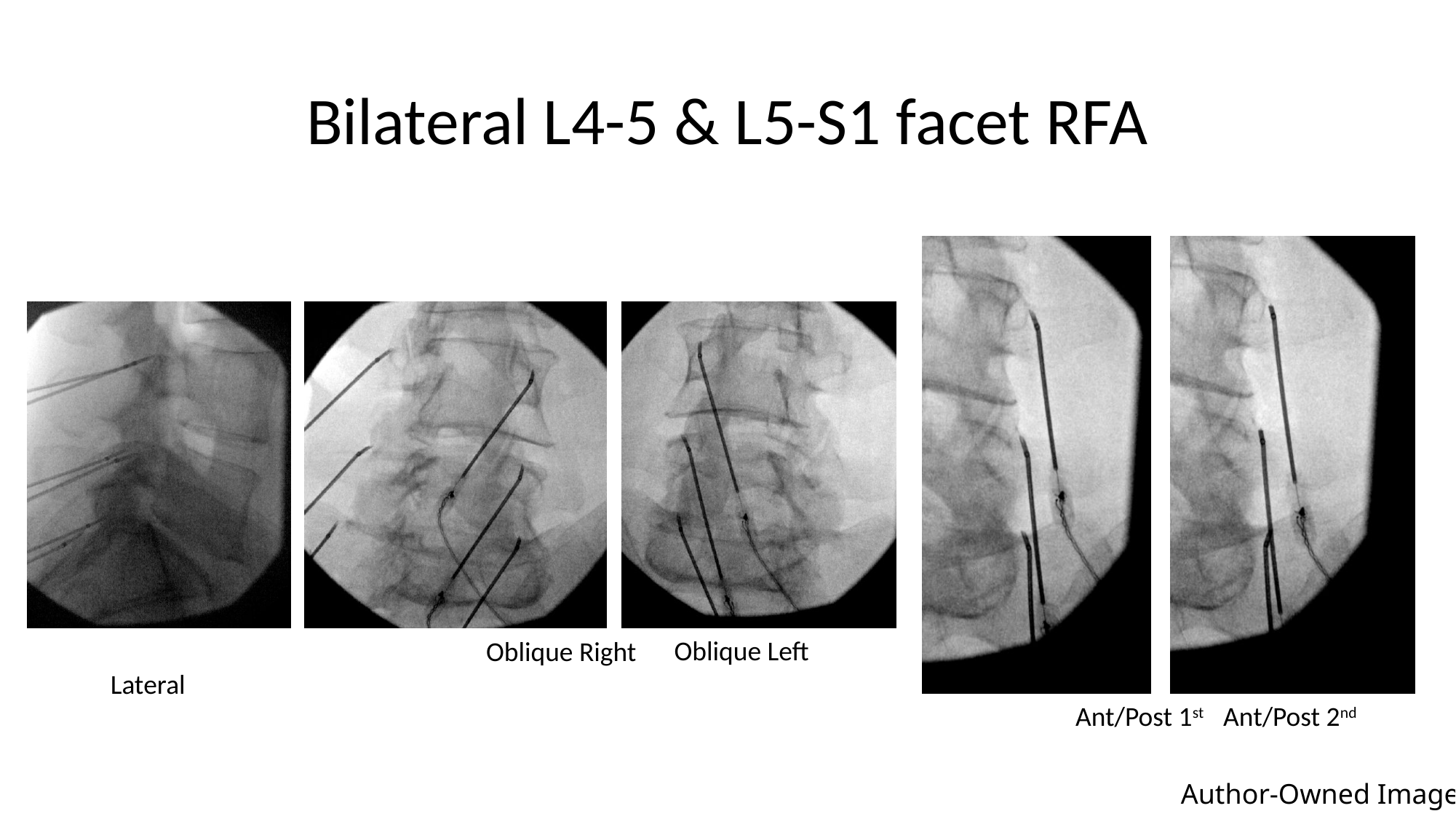

# Bilateral L4-5 & L5-S1 facet RFA
 Oblique Left
 Lateral
  Oblique Right
   Ant/Post 1st
Ant/Post 2nd
Author-Owned Images

## Slide 10
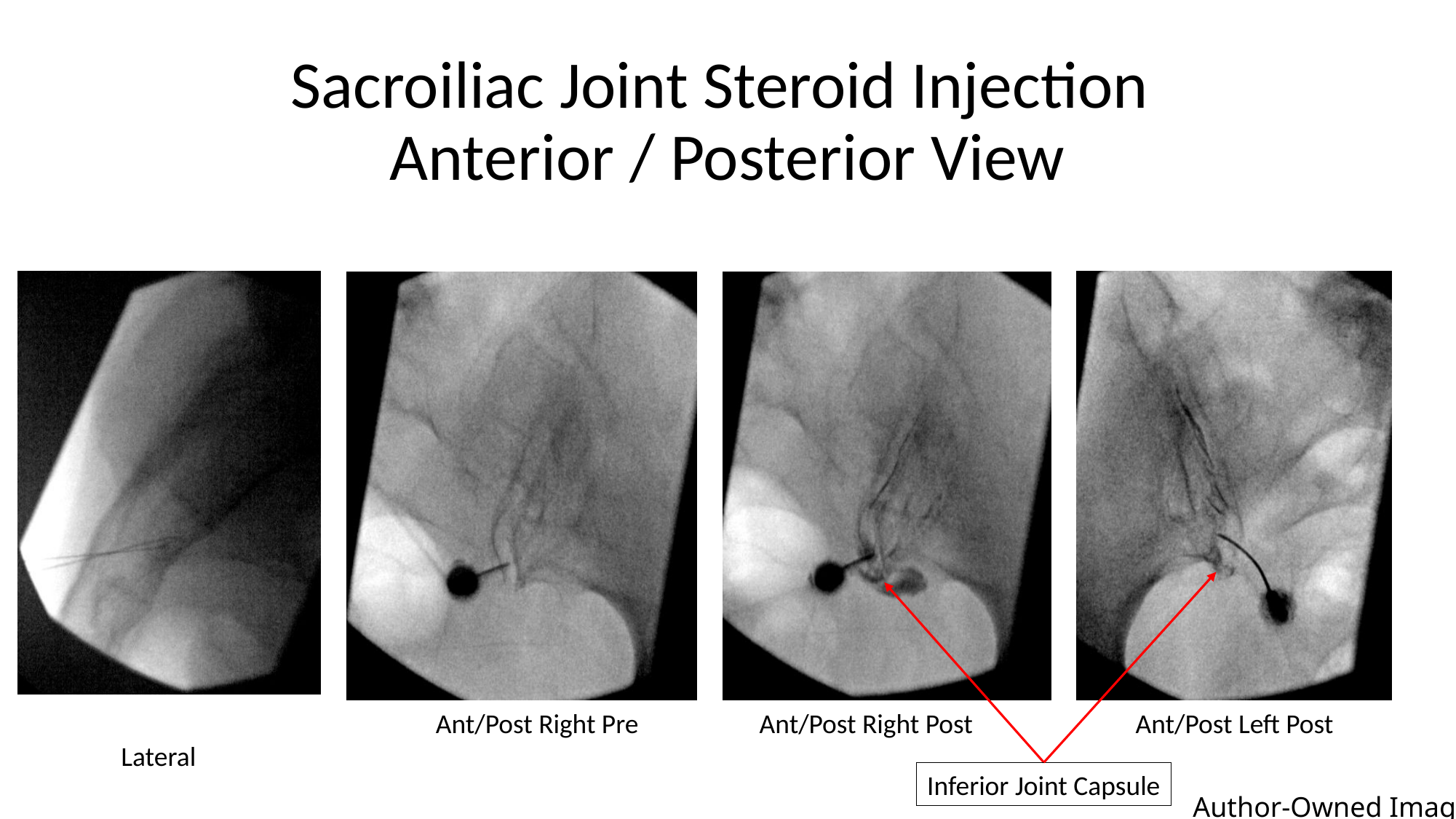

# Sacroiliac Joint Steroid Injection Anterior / Posterior View
Ant/Post Right Pre
  Ant/Post Right Post
Ant/Post Left Post
 Lateral
Inferior Joint Capsule
Author-Owned Images

## Slide 11
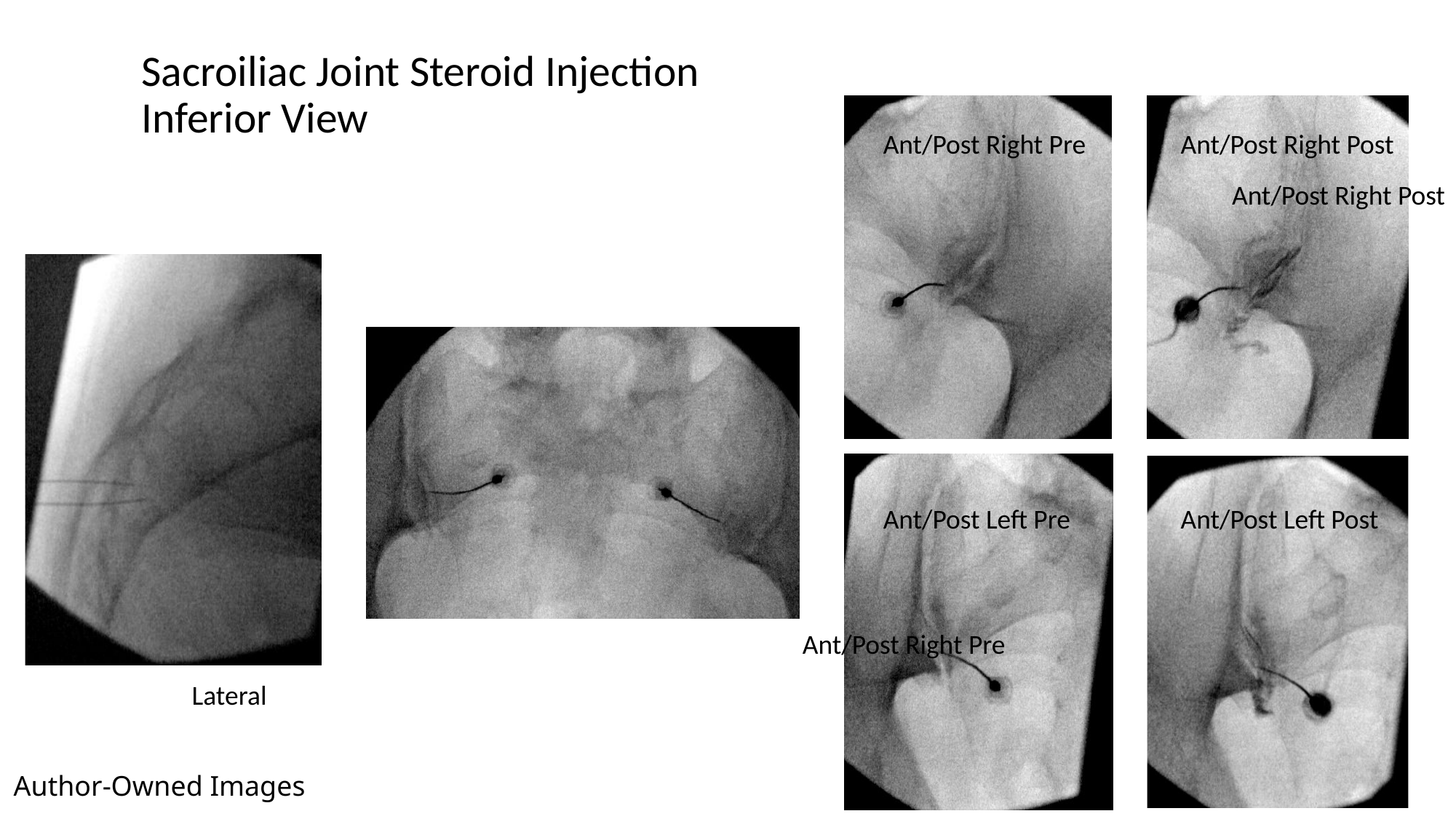

# Sacroiliac Joint Steroid Injection Inferior View
Ant/Post Right Pre
Ant/Post Right Post
Ant/Post Right Post
Ant/Post Left Pre
Ant/Post Left Post
  Ant/Post Right Pre
 Lateral
Author-Owned Images
