# Supplementary material for: Resident Interventional Spine Course with Didactics and Hands-On Skills Lab
Source: MedEdPORTAL. 2025 Oct 7;21:11551. doi: 10.15766/mep_2374-8265.11551 (PMC12502988; doi:10.15766/mep_2374-8265.11551)
Supplement: Supplementary file 1 — Overview - Spine.pptxPrep Kit Materials.docxBuilding a Low-Cost Spine Simulator.pptxFacilitators Guide.docxSpine Procedure - Guidelines Lecture.pptxSpine Procedure Guidelines Lecture Video.mp4Course Chart Review Guidelines.docxSpine Course - Cases.pptxChart Review Preprocedures Checklist.docxInformed Consent and Procedure Timeout Checklist.docxLumbar Procedure Table Checklist.docxProcedure Descriptions.docxFluoroscopic Spine Procedure Images.pptxSpine Course Pre-Post Survey - Updated.docxSpine Course Pre-Post Survey - Original.docx [file mep_2374-8265.11551-s001.zip › N. Spine Course Pre-Post Survey - Updated.docx]

| **Name:** | | **PGY:** | **Date:** | | | | | |
| --- | --- | --- | --- | --- | --- | --- | --- | --- |
| **Resident Interventional Spine Course – Pre**  ***1 = Not at all, 2 = Slightly, 3 = Moderately, 4 = Quite, 5 = Extremely*** | | | | **1** | **2** | **3** | **4** | **5** |
| **Overall Confidence:** | | | | | | | | |
| 1 | How confident are you in describing the specific indications/contraindications for spine procedures? | | |  |  |  |  |  |
| 2 | How confident are you in performing the informed consent process for spine procedures? | | |  |  |  |  |  |
| 3 | How confident are you in performing the set up and preparation for a spine procedure? | | |  |  |  |  |  |
| 4 | How confident are you in describing the targets and areas to avoid in basics lumbar procedures? | | |  |  |  |  |  |
| 5 | How confident are you in performing needle driving skills of basic lumbar procedures? | | |  |  |  |  |  |
| 6 | How confident are you in describing means to reduce and manage complications of basic lumbar procedures? | | |  |  |  |  |  |
| **Course Content:** | | | | | | | | |
| 1 | How helpful was the on-demand Spine Procedure Guidelines video? | | |  |  |  |  |  |
| 2 | How helpful were the pre-course articles/chapters/links? | | |  |  |  |  |  |
| **Comments:** | | | | | | | | |
| **I am looking to get these three things accomplished through this course:** | | | | | | | | |
| **I feel the best way for me to learn these above three things are through _________:** | | | | | | | | |
| **Other thoughts or recommendations:** | | | | | | | | |

| **Name:** | | **PGY:** | **Date:** | | | | | | |
| --- | --- | --- | --- | --- | --- | --- | --- | --- | --- |
| **Resident Interventional Spine Course – Post**  ***1 = Not at all, 2 = Slightly, 3 = Moderately, 4 = Quite, 5 = Extremely*** | | | | **1** | **2** | **3** | **4** | **5** |  |
| **Confidence:** | | | | | | | | |  |
| 1 | How confident are you in describing the specific indications/contraindications for spine procedures? | | |  |  |  |  |  |  |
| 2 | How confident are you in performing the informed consent process for spine procedures? | | |  |  |  |  |  |  |
| 3 | How confident are you in performing the set up and preparation for a spine procedure? | | |  |  |  |  |  |  |
| 4 | How confident are you in describing the targets and areas to avoid in basics lumbar procedures? | | |  |  |  |  |  |  |
| 5 | How confident are you in performing needle driving skills of basic lumbar procedures? | | |  |  |  |  |  |  |
| 6 | How confident are you in describing means to reduce and manage complications of basic lumbar procedures? | | |  |  |  |  |  |  |
| **Course Content:** | | | | | | | | |  |
| 1 | How helpful were the case reviews? | | |  |  |  |  |  |  |
| 2 | How helpful was the patient selection station? | | |  |  |  |  |  |  |
| 3 | How helpful was the procedure kit/informed consent preparation station? | | |  |  |  |  |  |  |
| 4 | How helpful was the procedure simulation station? | | |  |  |  |  |  |  |
| 5 | How helpful was the overall course? | | |  |  |  |  |  |  |
| **Comments:** | | | | | | | | |  |
| **Course Strengths:** | | | | | | | | |  |
| **Course Weaknesses:** | | | | | | | | |  |
| **Course Suggestions:** | | | | | | | | |  |
