# Supplementary material for: Resident Interventional Spine Course with Didactics and Hands-On Skills Lab
Source: MedEdPORTAL. 2025 Oct 7;21:11551. doi: 10.15766/mep_2374-8265.11551 (PMC12502988; doi:10.15766/mep_2374-8265.11551)
Supplement: Supplementary file 1 — Overview - Spine.pptxPrep Kit Materials.docxBuilding a Low-Cost Spine Simulator.pptxFacilitators Guide.docxSpine Procedure - Guidelines Lecture.pptxSpine Procedure Guidelines Lecture Video.mp4Course Chart Review Guidelines.docxSpine Course - Cases.pptxChart Review Preprocedures Checklist.docxInformed Consent and Procedure Timeout Checklist.docxLumbar Procedure Table Checklist.docxProcedure Descriptions.docxFluoroscopic Spine Procedure Images.pptxSpine Course Pre-Post Survey - Updated.docxSpine Course Pre-Post Survey - Original.docx [file mep_2374-8265.11551-s001.zip › O. Spine Course Pre-Post Survey - Original.docx]

| **Name:** | | **PGY:** | **Date:** | | | | | |
| --- | --- | --- | --- | --- | --- | --- | --- | --- |
| **Resident Interventional Spine Course – Pre**  ***(1 = Low to 5 = High)*** | | | | **1** | **2** | **3** | **4** | **5** |
| **Confidence:** | | | | | | | | |
| 1 | I feel confident in selecting patients for lumbar spine interventions. | | |  |  |  |  |  |
| 2 | I feel confident in reviewing the chart to identify risk factors | | |  |  |  |  |  |
| 3 | I feel confident in preparing a sterile procedure kit | | |  |  |  |  |  |
| 4 | I feel confident in basic lumbar procedures | | |  |  |  |  |  |
| 5 | I feel confident in addressing this course’s fluoroscopic procedure complications | | |  |  |  |  |  |
| **Knowledge:** | | | | | | | | |
| 1 | I can describe the specific indications/contraindications for spine interventions | | |  |  |  |  |  |
| 2 | I can discuss the informed consent process with a patient | | |  |  |  |  |  |
| 3 | I can describe the set up and preparation for a spine procedure | | |  |  |  |  |  |
| 4 | I can describe the targets and areas to avoid in basics lumbar procedures | | |  |  |  |  |  |
| 5 | I can describe means to reduce and manage complications from this course’s procedures | | |  |  |  |  |  |
| **Simulated Skills:** | | | | | | | | |
| 1 | I can do a full informed consent for basic lumbar procedures | | |  |  |  |  |  |
| 2 | I can set up a sterile procedure kit in preparation for a patient | | |  |  |  |  |  |
| 3 | I can do basic lumbar spine procedures | | |  |  |  |  |  |
| **Course Content:** | | | | | | | | |
| 1 | How helpful was the on-demand Spine Procedure Guidelines video (if utilized)? | | |  |  |  |  |  |
| 2 | How helpful were the pre-course articles/chapters/links? | | |  |  |  |  |  |
| 3 | How helpful were the supplementary materials? | | |  |  |  |  |  |
| **Comments:** | | | | | | | | |
| **I am looking to get these 3 things accomplished through this course:** | | | | | | | | |
| **I feel the best way for me to learn these above 3 things are though _________:** | | | | | | | | |
| **Other thoughts or recommendations:** | | | | | | | | |

| **Resident Interventional Spine Course – Post**  ***(1 = Low to 5 = High)*** | | **1** | **2** | **3** | **4** | **5** |
| --- | --- | --- | --- | --- | --- | --- |
| **Confidence:** | | | | | | |
| 1 | I feel confident in selecting patients for lumbar spine interventions. |  |  |  |  |  |
| 2 | I feel confident in reviewing the chart to identify risk factors |  |  |  |  |  |
| 3 | I feel confident in preparing a sterile procedure kit |  |  |  |  |  |
| 4 | I feel confident in basic lumbar fluoroscopic procedures |  |  |  |  |  |
| 5 | I feel confident in addressing the discussed fluoroscopic procedure complications |  |  |  |  |  |
| **Knowledge:** | | | | | | |
| 1 | I can describe the specific indications/contraindications for spine interventions |  |  |  |  |  |
| 2 | I can discuss the informed consent process with a patient |  |  |  |  |  |
| 3 | I can describe the set up and preparation for a spine procedure |  |  |  |  |  |
| 4 | I can describe the targets and areas to avoid in basic lumbar procedures |  |  |  |  |  |
| 5 | I can describe means to reduce and manage complications from this course’s procedures |  |  |  |  |  |
| **Simulated Skills:** | | | | | | |
| 1 | I can do a full informed consent for basic lumbar procedures |  |  |  |  |  |
| 2 | I can set up a sterile procedure kit in preparation for a patient |  |  |  |  |  |
| 3 | I can do basic lumbar spine procedures |  |  |  |  |  |
| **Course Content:** | | | | | | |
| 1 | How helpful were the case reviews? |  |  |  |  |  |
| 2 | How helpful was the patient selection station? |  |  |  |  |  |
| 3 | How helpful was the procedure kit/informed consent preparation station? |  |  |  |  |  |
| 4 | How helpful was the procedure simulation station? |  |  |  |  |  |
| 5 | How helpful was the overall course? |  |  |  |  |  |
| **Comments:** | | | | | | |
| **Course Strengths (3):** | | | | | | |
| **Course Weaknesses (3):** | | | | | | |
| **Course Suggestions (3):** | | | | | | |
